# Proteomic analysis upon peach fruit infection with *Monilinia fructicola* and *M. laxa* identify responses contributing to brown rot resistance

**DOI:** 10.1038/s41598-020-64864-x

**Published:** 2020-05-08

**Authors:** Antonios Papavasileiou, Georgia Tanou, Anastasios Samaras, Martina Samiotaki, Athanassios Molassiotis, George Karaoglanidis

**Affiliations:** 1 Laboratory of Plant Pathology, Faculty of Agriculture, Forestry and Natural Environment, Aristotle University, POB 269, 54124 Thessaloniki, Greece; 2 Institute of Soil and Water Resources, ELGO-Demeter Thermi, Thessaloniki, Greece; 3 Biomedical Sciences Research Center “Alexander Fleming”, Vari, 16672 Greece; 4 Laboratory of Pomology, Faculty of Agriculture, Forestry and Natural Environment, Aristotle University, 570 01 Thessaloniki-Thermi, Greece

**Keywords:** Biological techniques, Microbiology, Plant sciences

## Abstract

Brown rot, caused by *Monilinia* spp., is a major peach disease worldwide. In this study, the response of peach cultivars Royal Glory (RG) and Rich Lady (RL) to infection by *Monilinia fructicola* or *Monilinia laxa*, was characterized. Phenotypic data, after artificial inoculations, revealed that ‘RL’ was relatively susceptible whereas ‘RG’ was moderately resistant to *Monilinia* spp. Comparative proteomic analysis identified mesocarp proteins of the 2 cultivars whose accumulation were altered by the 2 *Monilinia* species. Functional analysis indicated that pathogen-affected proteins in ‘RG’ were mainly involved in energy and metabolism, while, differentially accumulated proteins by the pathogen presence in ‘RL’ were involved in disease/defense and metabolism. A higher number of proteins was differentiated in ‘RG’ fruit compared to ‘RL’. Upon *Monilinia* spp. infection, various proteins were-down accumulated in ‘RL’ fruit. Protein identification by mass spectrometric analysis revealed that several defense-related proteins including thaumatin, formate dehydrogenase, *S*-formylglutathione hydrolase, CBS domain-containing protein, HSP70, and glutathione *S*-transferase were up-accumulated in ‘RG’ fruit following inoculation. The expression profile of selected defense-related genes, such as major latex allergen, 1-aminocyclopropane-1-carboxylate deaminase and UDP-glycoltransferase was assessed by RT-PCR. This is the first study deciphering differential regulations of peach fruit proteome upon *Monilinia* infection elucidating resistance responses.

## Introduction

Brown rot is one of the most important and common diseases of peach (*Prunus persica* (L) Batsch) causing devastating yield losses that can exceed 50% or even reach 100% under environmental conditions favorable for the development of the disease^1,2^. The disease occurs wherever stone fruits are cultivated and causes blossom, twig or fruit blight and pre-or post- harvest fruit rots. The latter are associated with the most severe yield losses^2^. On stone fruit the disease is caused by several *Monilinia* species, with *Monilinia fructicola* and *Monilinia laxa* being the predominant^3^. *M. laxa* is widely distributed around the world and considered to be the most common brown rot pathogen in Europe^3^. In addition, *M. fructicola* occurs mainly in America, Australia, China and Japan. However, several reports of the last 2 decades have confirmed the presence of *M. fructicola* in South and Central European countries^4–7^.

The control of the disease relies mainly on the use of synthetic fungicides and thus, they are essential for providing stone fruits of high quality at affordable prices^8–10^. However, the increased public concern about food safety and the potential impact on the environment, combined with concerns of the peach industry regarding fungicide resistance and the emergence of new resistant strains have led to extensive research for the development of disease management methods alternative to conventional fungicides^11,12^. Such alternatives include biological control with microbial antagonists or use of resistance inducers, but none of these approaches has gained until now a commercial feasibility^12–14^.

Host resistance to brown rot could be an attractive, cost effective and environmentally friendly alternative to chemical control. However, most of the commercial peach cultivars are susceptible to brown rot, although some differences exist among them in the level of susceptibility to the disease^15–17^. The susceptibility of peach fruit is also associated with the stages of their development and appear more susceptible in the very early stages, during the development of the pericarp and later at the stage of ripeness of the fruit, after the endocarp and the mesocarp have been fully developed^8,11^. The mechanisms behind the different sensitivity levels at different stages are allegedly due to physical and chemical reasons^18^. For instance, the high levels of resistance in the peach cultivar Bolinha are associated with the presence of higher concentrations of phenolic compounds, such as caffeic acid and its quinate ester, chlorogenic acid, located in the epidermis and mesocarp^8,11–21^. While these substances have no direct effect on mycelial growth and spore germination, they inhibit the production of the cell wall degrading enzymes polygalactorunase and cutinase, by down regulating their respective genes^8^. Additional factors, also playing an important role in resistance to the disease, are associated with different levels of the environmental pH of the host, regulated by the pathogen and affecting the expression of genes, responsible for pathogenesis^22^. Despite intensive investigation, the mechanism of peach fruit brown rot resistance remains unknown.

In recent years, proteomics has gained popularity in understanding host–pathogen interactions^23,24^. Comparative proteomics have been successfully used to study interactions of plants with a wide range of pathogens^25^. However, no proteomic studies have been published to date investigating the interactions of peach fruit with *Monilinia* species. To address this issue, the responses of two peach cultivars, ‘RL’ and ‘RG’, differing in their level of resistance to infection by *M. fructicola* or *M. laxa*, was characterized. Using two-dimensional gel electrophoresis (2-DE) analysis, we compared the proteomic changes from the fruit mesocarp of the two cultivars following inoculation with either *M. fructicola* or *M. laxa* to identify proteins potentially involved in defense mechanisms.

## Results

### Disease incidence

**Figure 1:**
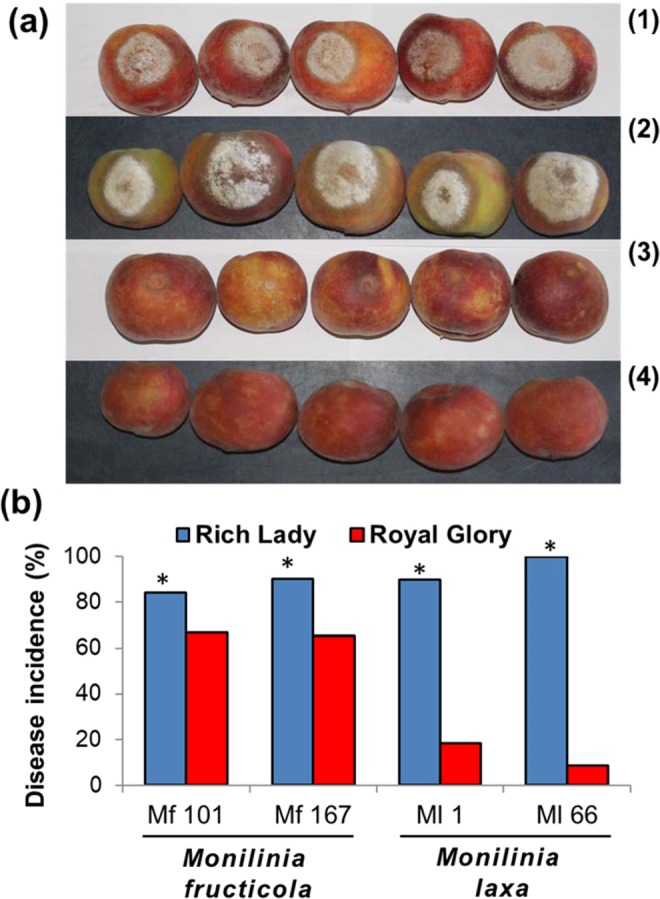
(**a**) Brown rot symptoms on peach fruit of Rich Lady (1, 2) and Royal Glory (3, 4) cultivars 6 days after the inoculation with isolates of *Monilinia fructicola* (1, 3) and *M. laxa* (2, 4). (**b**) Disease incidence on fruits of Rich Lady and Royal Glory cultivars artificially inoculated with *M. fructicola* (Mf 101, Mf 167) and *M. laxa* (Ml 1, Ml 66) isolates. Asterisks on the bars indicate significant differences between the disease incidence values in the 2 cultivars, according to a chi square analysis at P = 0.05.

The artificial inoculations of the fruit of the 2 different peach cultivars revealed a higher rate of disease incidence at ‘RL’ fruit compared to that observed at ‘RG’ fruit, either inoculated with *M. fructicola* or *M. laxa* isolates (Fig. 1a,b). However, the difference in the disease incidence between the 2 cultivars was lower, when the fruits were inoculated with *M. fructicola* isolates Mf 101 and Mf 167, with disease incidence values of 84.2 and 90.5% in ‘RL’ and 66.7 and 65.2% in ‘RG’, respectively (Fig. 1b). Moreover, a higher difference in disease incidence was observed, when fruits were inoculated with *M. laxa* isolates Ml 1 and Ml 66, with infected fruit rates reaching 90 and 100% in ‘RL’ and only 18.2 and 8.7% in ‘RG’, respectively (Fig. 1b).

### Characterization of the peach fruit proteome

**Figure 2:**
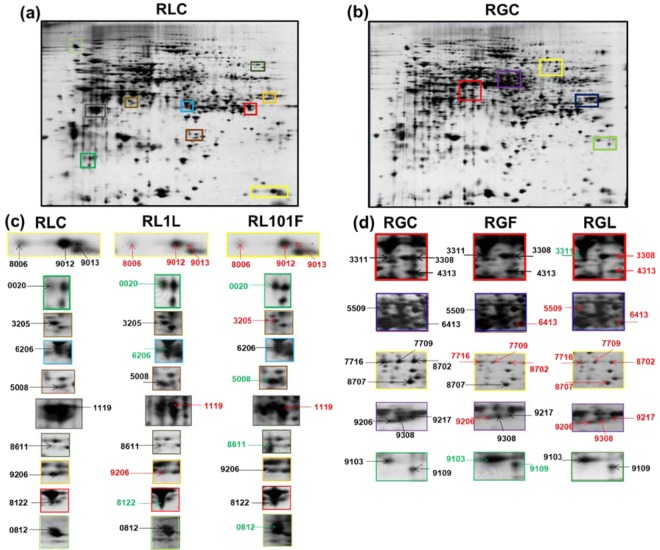
Representative silver-stained 2-DE map of total proteins from (**a**) Rich Lady (RLC) and (**b**) Royal Glory (RGC) control peach cultivars. (**c)** Zoomed in views of selected areas from RLC in relation to ‘Rich Lady’ fruits inoculated with *M. laxa* (RL1L) or *M. fructicola* (RL101F), showing differences in the protein spots intensity. (**d**) Enlarged views of RGC *versus* ‘Royal Glory’ fruits inoculated with *M. laxa* (RGL) or *M. fructicola* (RGF). Black, green and red arrows indicate protein spots whose abundance remained unchanged, increased, or decreased, respectively in comparison to control. Full-length gels of which zoomed in views of selected areas are given in Supplementary Data Fig. S2.

To obtain a better understanding of the mechanisms behind the disease incidence data, a two-dimensional gel electrophoresis (2-DE) analysis was conducted to detect the protein changes in both cultivars ‘RL’ and ‘RG’ (Fig. 2a,b). In both cultivars a total of 250 protein spots, were detected, while 120 of them showed significant differences in their volume in the presence of either *M. fructicola* or *M. laxa* (Fig. 2c,d, Supplementary Data Figs. S1 and S2), according to the Student’s t-test for 95% confidence further validated by the two-fold change threshold. Following mass spectrum analysis, 126 different peach proteins were identified, while 11 of them were detected in more than one spots. These proteins were aconitate hydratase (spots no.: 4828, 4829), adenylyl cyclase-associated protein (spots no: 8510, 8520), aminoacylase (spots no.: 2311, 3316), aspartate aminotransferase (spots no: 9214, 9216), HSP70 (spots no: 1705, 1821, 2701, 2704), malic enzyme (spots no: 3616, 5615, 8604), peptidyl-prolyl cis-trans isomerase (spots no: 1705, 9013), phosphoenolpyruvate carboxykinase [ATP] (spots no: 6708, 7707, 7709, 8711), phosphoglycerate kinase (spots no: 6206, 7301), *S*-adenosylmethionine synthase (spots no: 4208, 4313), thaumatin (spots no: 5008, 9103). Detailed information regarding the identified proteins are provided in Supplementary Data Table S1. Subsequently, proteins based on gene ontology and literature, were grouped into functional categories^26^.

### Functional analysis of proteins modulated by the presence of the pathogen relative to the control fruit, within the same peach cultivar

**Figure 3:**
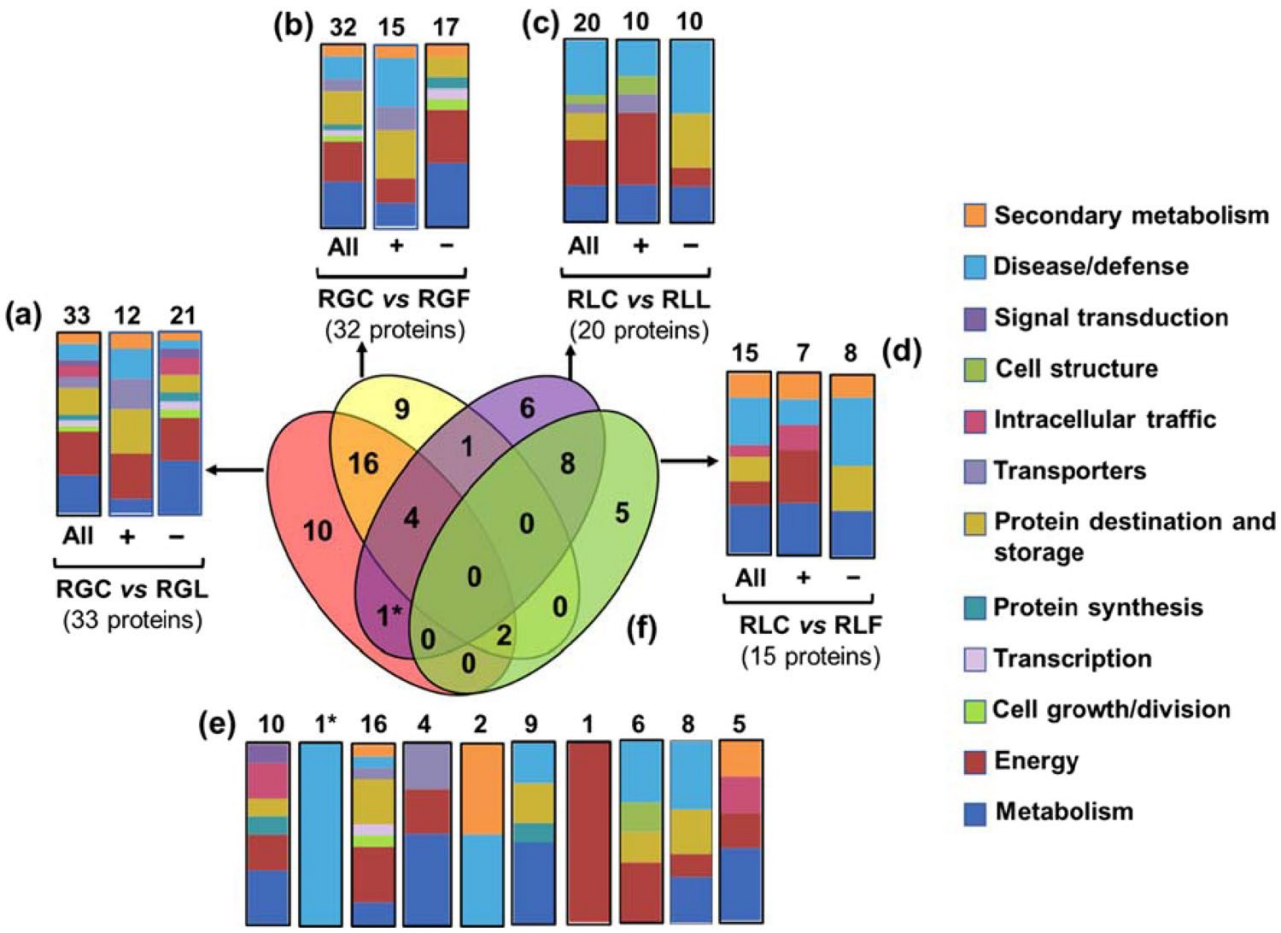
Venn diagram presenting the common and distinct differentially expressed proteins in peach cultivars after inoculation with *M. fructicola* and *M. laxa*, respectively. Graphical representation of the functional classification of the differentially expressed proteins in: (**a**) ‘Royal Glory’ inoculated with *M. laxa*, (**b**) ‘Royal Glory’ inoculated with *M. fructicola*, (**c**) ‘Rich Lady’ inoculated with *M. laxa*, (**d**) ‘Rich Lady’ inoculated with *M. fructicola*. (**e**) Functional classification of the unique and overlapping proteins presented in the Venn diagram (**f**). Symbols (+) and (−) indicate up-regulated and down-regulated proteins in each treatment. (RGC: ‘Royal Glory’ control fruits, RGL: ‘Royal Glory’ fruits inoculated with *M. laxa*, RGF: ‘Royal Glory’ fruits inoculated with *M. fructicola*, RLC: ‘Rich Lady’ control fruits, RLL: ‘Rich Lady’ fruits inoculated with *M. laxa*, RLF: ‘Rich Lady’ fruits inoculated with *M. fructicola*).

**Figure 4:**
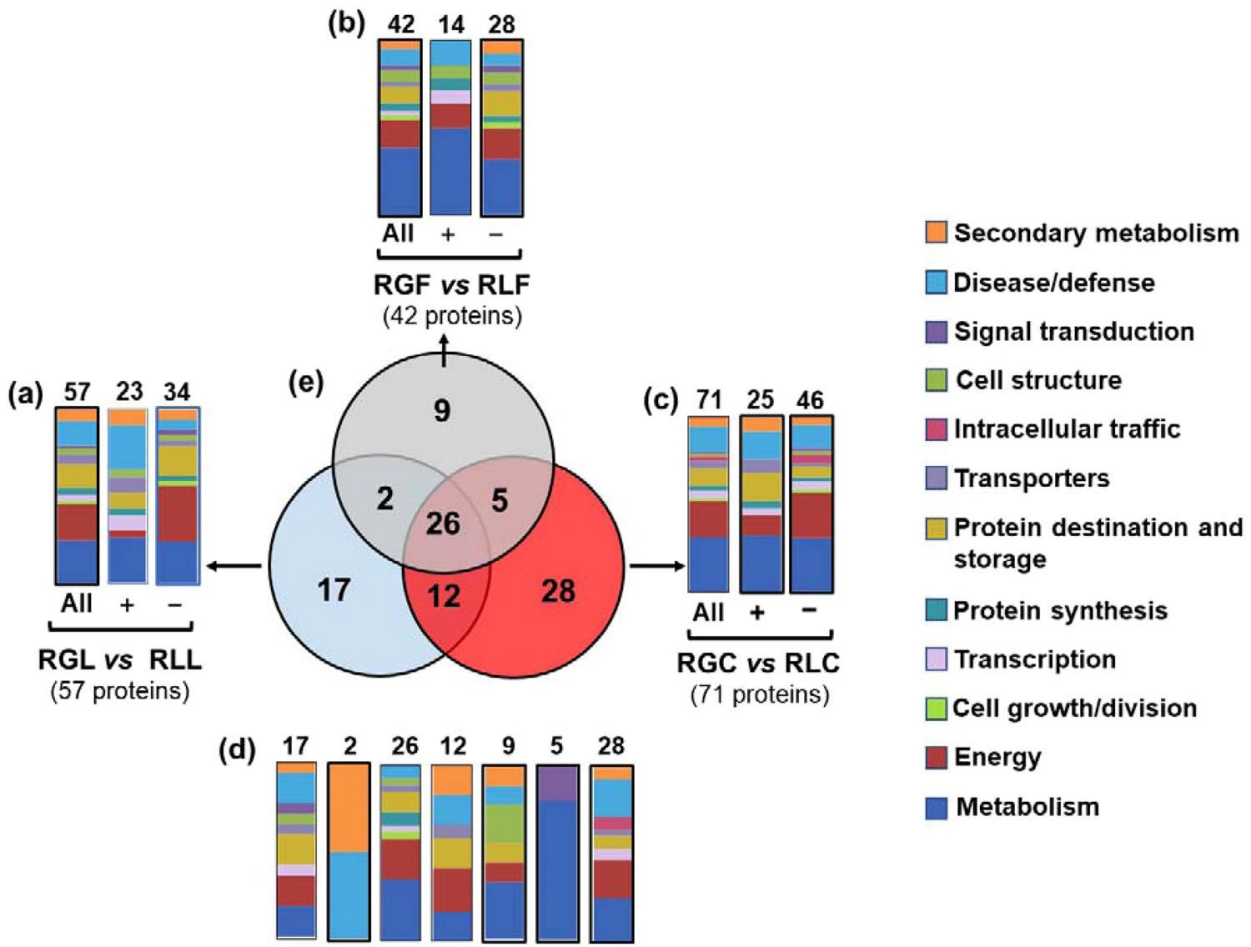
Venn diagram presenting the common and unique differentially expressed proteins among Royal Glory and Rich Lady cultivars inoculated with the same pathogen. Graphical representation of the functional classification of the differentially expressed proteins among the 2 varieties, (**a**) inoculated with *M. laxa*, (**b**) inoculated with *M. fructicola*, c) control treatments. (**d**) Functional classification of the unique and overlapping proteins presented in the Venn diagram **(e**). Symbols (+) and (−) indicate up-regulated and down-regulated proteins in each treatment. (RGC: ‘Royal Glory’ control fruits, RGL: ‘Royal Glory’ fruits inoculated with *M. laxa*, RGF: ‘Royal Glory’ fruits inoculated with *M. fructicola*, RLC: ‘Rich Lady’ control fruits, RLL: ‘Rich Lady’ fruits inoculated with *M. laxa*, RLF: ‘Rich Lady’ fruits inoculated with *M. fructicola*).

In order to identify the peach proteins whose abundance was significantly changed (increased or decreased) by the presence of the pathogen in relation to the control, within the same peach cultivar, proteins were grouped into four sets: (1) a set of 33 proteins modulated in ‘RG’ by the presence of *M. laxa* (RGC *vs* RGL) (Fig. 3a), (2) a set of 32 proteins modulated in ‘RG’ by the presence of *M. fructicola* (RGC *vs* RGF) (Fig. 3b), (3) a set of 20 proteins modulated by the presence of *M. laxa*, in ‘RL’ (RLC *vs* RLL) (Fig. 3c) and (4) a set of 15 proteins modulated by the presence of *M. fructicola*, in ‘RL’ (RLC *vs* RLF) (Fig. 3d). Functional analysis revealed that the *M. laxa* affected several proteins (n = 33) in ‘RG’ that were mainly involved in energy (24.2%), metabolism (21.2%) and protein destination and storage (15.1%) (Fig. 3a). Similarly, the *M. fructicola* affected proteins in the same cultivar were mainly involved in metabolism (25%), energy (21.8%) and protein destination and storage (18.7%) (Fig. 3b). In contrast, *M. laxa* affected proteins in ‘RL’ participated mainly in disease/defense (30%), energy (25%) and metabolism (20%) (RLC *vs* RLL) (Fig. 3c) while, the *M. fructicola* affected proteins in the same cultivar participated in disease/defense (26.7%), metabolism (26.7%), energy (13.3%), protein destination and storage (13.3%) and secondary metabolism (13.3%) (RLC *vs* RLF) (Fig. 3d).

### Proteins functional analysis: ‘Royal Glory’ and ‘Rich Lady’ inoculated with *M. laxa* or *M. fructicola*, versus not inoculated cultivars

Information concerning distinct or common proteins affected by *M. fructicola* or *M. laxa* in the 2 peach cultivars, in relation to control fruits, are given in Fig. 3f. Among the 2 sets of proteins modulated in ‘RG’ by the presence of *M. fructicola* and *M. laxa*, 16 proteins were common, while 10 proteins were specifically modulated in response to *M. laxa* and 9 proteins were specifically modulated in response to *M. fructicola* (Fig. 3f). In ‘RL’ fruits, 6 and 5 proteins were individually changed by the presence of *M. laxa* and *M. fructicola*, respectively, while 8 proteins overlapped between the 2 sets of proteins (Fig. 3f).

Venn diagrams were further analyzed to determine the functional distribution of distinct or overlapping proteins. Proteins that were specifically modulated by *M. laxa* (*n* = 10) in ‘RG’ in relation with the control treatment were mainly involved in metabolism (30%), energy (20%) and intracellular traffic (20%) (Fig. 3e,f), while proteins specifically modulated by *M. fructicola* (*n* = 9), in ‘RG’ (RGC *vs* RGF) were mainly involved in metabolism (44.4%), protein destination and storage (22.2%), disease/defense (22.2%) (Fig. 3e,f). Proteins commonly affected by *M. laxa* and *M. fructicola* (n = 16) in ‘RG’ were involved in protein destination and storage (25%), energy (31.2%) and metabolism (12.5%) (Fig. 3e,f).

Proteins identified exclusively in ‘RL’ after inoculation with *M. laxa* (n = 6) were involved in energy (33.3%), disease/defense (33.3%), protein destination and storage (16.7%) and cell structure (16.7%) (Fig. 3e,f), while proteins (n = 5) exclusively identified in ‘RL’ after inoculation with *M. fructicola* were involved in metabolism (40%), energy (20%), intracellular traffic (20%) and secondary metabolism (20%) (Fig. 3e,f). Common targeted proteins among *M. laxa* and *M. fructictola* infected fruits of ‘RL’ (n = 8) were involved in disease/defense (37.5%), metabolism (25%) and protein destination and storage (25%) (Fig. 3e,f).

Comparing the proteins modulated by the pathogen in ‘RG’ (RGC *vs* RGL) 12 of the 33 proteins were up-regulated by *M. laxa* and they were involved mainly in energy (25%), cell growth/division (25%), disease/defense (16.7%) and transporters (16.7%), while, 21 proteins were down-regulated and participated mainly in metabolism (28%) and energy (24%) (Fig. 3a). In the presence of *M. fructicola* in the same cultivar (RGC *vs* RGF), 15 proteins were up-regulated, involved mainly in disease/defense (26%) and protein destination and storage (26%) whereas, 17 proteins were down-regulated mainly involved in metabolism (35%) and energy (29%) (Fig. 3b). Furthermore, in ‘RL’ (RLC *vs* RLL) out of the 20 proteins modulated by the presence of *M. laxa*, 10 of them were increased in abundance and involved mainly in energy (40%), disease/defense (20%) and metabolism (20%), while the remaining 10 down-regulated proteins were involved in disease/defense (40%) and protein destination and storage (30%) (Fig. 3c). Seven out of 15 proteins in the same cultivar (RLC *vs* RLF) were up-regulated by the presence of *M. fructicola* and involved in metabolism (28%), energy (28%), intracellular traffic (14%), disease/defense (14%), secondary metabolism (14%) while 8 of them were down regulated and involved in disease/defense (37%), metabolism (25%) and protein destination and storage (25%) (Fig. 3d).

### Proteins functional analysis: comparison between ‘Royal Glory’ and ‘Rich Lady’ inoculated with the same *Monilinia* species

Comparison of proteins that differed between the 2 cultivars due to the presence of the same pathogen, *M. fructicola* or *M. laxa*, 3 sets of proteins were defined: a set of 57 proteins varied due to the presence of *M. laxa* (RGL *vs* RLL), (Fig. 4a), a set of 42 proteins changed in response to *M. fructicola* (RGF *vs* RLF), (Fig. 4b) and a set of 71 proteins differed between ‘RL’ and ‘RG’ in the absence of any pathogen (RGC *vs* RLC) (Fig. 4c).

The *M. laxa* modulated proteins, which were differentiated between the inoculated ‘RG’ and ‘RL’ fruits (n = 57) were mostly involved in metabolism (24.6%), energy (21%), protein destination and storage (14%) and disease/defense (14%) (Fig. 4a). Of the 57 modulated proteins, 23 of them were up-regulated and involved in metabolism (26%) and disease/defense (26%), while 34 were down-regulated and participated in energy (32%) and metabolism (23.5%). In addition, proteins affected by *M. fructicola* differentially in the inoculated ‘RG’ and ‘RL’ fruits (n = 42) were involved in metabolism (38.1%) and energy (16.6%) (Fig. 4b). Of them 14 proteins were up-regulated and 28 down-regulated accounting majorly for the same functional categories. The proteomic maps of control fruits of ‘RL’ and ‘RG’ were varied in proteins (n = 71) mostly involved in metabolism (31%), energy (21.1%) and disease/defence (14.1%) (Fig. 4c). Of them, 25 proteins were up-regulated and participate mainly in metabolism (32%), protein destination and storage (16%) and disease/defense (16%), whereas the major categories for the 46 down-regulated proteins are metabolism (30%) and energy (26%).

Information on common and distinct proteins between the ‘RG’ and ‘RL’ peach cultivars inoculated with the same pathogen are provided in Fig. 4e. The commonly affected proteins (n = 26) between the 3 sets of proteins modified by the presence of the same pathogen or in the absence of pathogen, between ‘RL’ and ‘RG’ respectively, were mainly involved in metabolism (34.6%), energy (23.1%) and protein destination and storage (11.5%) (Fig. 4d). Proteins targeted specifically by the presence of *M. laxa* (n = 17) between ‘RL’ and ‘RG’ were mainly involved in metabolism (17.6%), energy (17.6%), protein destination and storage (17.6%) and disease/defense (17.6%) (Fig. 4a). Proteins related with metabolism (33.3%) and cell structure (22.2%) were predominant in the set of proteins (n = 9) exclusively responsive to the inoculation with *M. fructicola* between ‘RL’ and ‘RG’ (Fig. 4e). Moreover, proteins individually identified between control conditions (n = 28) of the 2 peach cultivars were involved in metabolism (25%), energy (21.4%) and disease/defense (21.4%) (Fig. 4e).

### Proteins functional analysis: comparison between ‘Royal Glory’ and ‘Rich Lady’ inoculated with different *Monilinia* species

**Figure 5:**
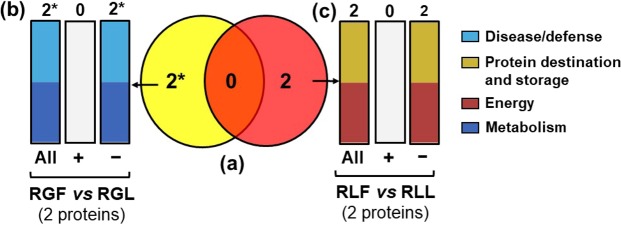
Venn diagram (**a**) presenting unique and common proteins within the same cultivar after inoculation with *M. fructicola* and *M. laxa* individually. Functional classification of differentially expressed proteins in ‘Royal Glory’ (**b**) and ‘Rich Lady’ (**c**), inoculated with *M. fructicola* and *M. laxa*, respectively. Symbols (+) and (−) indicate up-regulated and down-regulated proteins in each treatment. (RGL: ‘Royal Glory’ fruits inoculated with *M. laxa*, RGF: ‘Royal Glory’ fruits inoculated with *M. fructicola*, RLL: ‘Rich Lady’ fruits inoculated with *M. laxa*, RLF: ‘Rich Lady’ fruits inoculated with *M. fructicola*).

Comparisons performed to identify proteins modulated within the same peach cultivar in response to the 2 different pathogens, *M. fructicola* and *M. laxa*, revealed a set of 2 proteins in ‘RG’ differed between the fruits which were inoculated by *M. fructicola* and *M. laxa* (RGF *vs* RGL) (Fig. 5b) and a set of another 2 proteins in ‘RL’ differed between the fruits which were exposed to *M. fructicola* and *M. laxa*, respectively (RLF *vs* RLL) (Fig. 5c).

Protein differences within ‘RG’ inoculated with either *M. fructicola* or *M. laxa*, were involved in energy (50%) and protein destination and storage (50%) (RGF *vs* RGL) (Fig. 5b) while differentiated proteins within ‘RL’ were participate in metabolism (50%) and disease/defense (50%) (RLF *vs* RLL) (Fig. 5c).

There were no common proteins among the 2 group of proteins modulated within the same cultivar by the presence of a different pathogen, *M. fructicola* and *M. laxa*, respectively (Fig. 5a). Only 2 proteins were specifically differentiated within ‘RG’ and ‘RL’, respectively (Fig. 5b,c).

**Figure 6:**
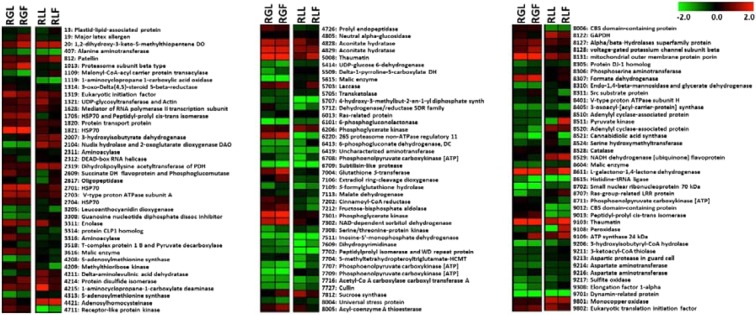
Protein abundance changes in ‘Royal Glory’ and ‘Rich Lady’ peach fruit inoculated with *Monilinia fructicola* or *M. laxa*. Heat map of proteins that demonstrated statistically significant differences among the different experimental conditions (RGC: ‘Royal Glory’ control fruits, RGL: ‘Royal Glory’ fruits inoculated with *M. laxa*, RGF: ‘Royal Glory’ fruits inoculated with *M. fructicola*, RLC: ‘Rich Lady’ control fruits, RLL: ‘Rich Lady’ fruits inoculated with *M. laxa*, RLF: ‘Rich Lady’ fruits inoculated with *M. fructicola*). Color scale shows the relative abundance of each protein across the experimental conditions as it has been calculated as log2 of the ratio of the protein abundance of the RGL or RGF or RLL or RLF to the protein abundance of the corresponding control; red and green indicate respectively enhanced and reduced abundance in RGL or RGF or RLL or RLF samples in comparison to the corresponding control. Proteins correspond to that listed in Supplementary Table 1.

Peach proteins of the mesocarp of ‘RL’ and ‘RG’ cultivars differentiated (up-accumulated or down-accumulated) after inoculation with *M. fructicola* or M*. laxa* are given as heat map profile in Fig. 6 and discussed below.

### Quantitative RT-PCR assays

**Figure 7:**
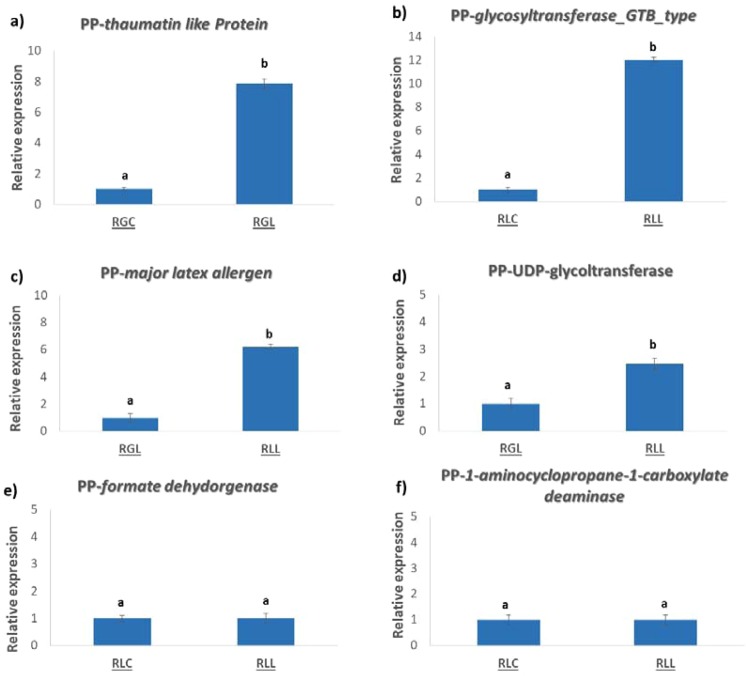
Transcript levels of *Prunus persicae* (PP) defense-related genes (**a**) thaumatin like protein, (**b**) glycotransferase_GTB_type, (**c**) major latex allergen, (**d**) UDP-glycotransferase, (**e**) formate dehydrogenase and (**f**) 1-aminocyclopropane-1-carboxylate deaminase in fruits of ‘Royal Glory’ and ‘Rich Lady’ inoculated with *Monilinia fructicola* or *M. laxa*. Actin and ubiquinone were used as reference genes. (RGC: ‘Royal Glory’ control fruits, RGL: ‘Royal Glory’ fruits inoculated with *M. laxa*, RGF: ‘Royal Glory’ fruits inoculated with *M. fructicola*, RLC: ‘Rich Lady’ control fruits, RLL: ‘Rich Lady’ fruits inoculated with *M. laxa*, RLF: ‘Rich Lady’ fruits inoculated with *M. fructicola*). Different letters on the columns indicate significant differences according to analysis of variance (ANOVA) at *P* = 0.05.

To investigate the correlation between proteins accumulation level and mRNA expression, a RT-qPCR analysis was performed for 6 representative defense-related genes, selected based on the data derived from the proteome analysis. Using this approach, we found that 4 of these 6 genes, namely thaumatin (Fig. 7a), glycosyltransferase_GTB_type (Fig. 7b), major latex allergen (Fig. 7c) and UDP-glycoltransferase (Fig. 7d) were in agreement with the corresponding protein levels as analyzed by proteomic analysis (Fig. 7; Supplementary Data Table S1). However, no correlation between mRNA expression and protein abundance of formate dehydrogenase (Fig. 7e) and 1-aminocyclopropane-1-carboxylate deaminase (Fig. 7f) was observed.

## Discussion

This study represents a first attempt to investigate the response of peach fruit at proteomic level following infection by either *M. laxa* or *M. fructicola*, the two most common brown rot agents, and may constitute a groundwork for future studies. The phenotypical data acquired by artificial inoculations, revealed significant differences in the levels of resistance to brown rot between the two different peach cultivars used making them an interesting experimental model to characterize proteome-based responses to the disease.

In this work, the comparison of the proteomic profile of artificially-inoculated and non-inoculated control fruit of ‘RG’ and ‘RL’ cultivars, showed that a higher number of proteins was changed in the ‘RG’ fruit exposed to either *M. fructicola* or *M. laxa*. Such results suggest that the relatively resistant cultivar displayed a stronger proteome-associated response to brown rot infection in relation to the more susceptible cultivar. Despite the differences in the disease incidence between *M. fructicola* and *M. laxa* observed in both cultivars, the artificial inoculations with both *Monilinia* species induced a similar proteomic response in the two cultivars. Thus, it is tempting to speculate that the peach fruit’s response to infection is presumably differentiated in terms of cultivar-specific protein changes that are involved in the resistance. On this basis, an interesting finding that emerged from this work is the overall accumulation of various defense-related proteins such as thaumatin, formate dehydrogenase, *S*-formylglutathione hydrolase, UDP-glycosyltransferase, 1-aminocyclopropane-1-carboxylate deaminase, CBS domain-containing protein, HSP70, L-galactono-1,4-lactone dehydrogenase, phosphoglycerate kinase, mitochondrial outer membrane protein porin, major latex allergen and glutathione *S*-transferase that observed in the relatively resistant ‘RG’ fruit following inoculation with either *M. fructicola* or *M. laxa*. For several of these proteins (thaumatin, major latex allergen, glutathione *S*-transferase or UDP-glycosyltransferase) their up-accumulation was confirmed by the over-expression of the respective genes using qPCR. However, there were also some of these proteins (1-aminocyclopropane-1-carboxylate deaminase, formate dehydrogenase) for which their over-accumulation was not confirmed by the qPCR data. This mismatch between the protein and gene expression can be due to various reasons such as post-transcriptional processes that may modify the active protein levels, such as synthesis, degradation, processing and post-translational modification, as shown in many studies^23,27^. In the following, we discuss the changes of several of these defense-related proteins and their respective encoding genes that presented in Fig. 6, in an effort, to determine their biological functions and their contribution in the defense of peach fruits against brown rot.

Plant cells have displayed specific defense‐related protein reprograming to combat pathogen infection^23^. One of the acquired modes in response to pathogen attack is the production of the pathogenesis-related (PR) proteins, which play key roles in plant disease-resistance responses^28^. For example, thaumatins and thaumatin-like proteins with similar nucleotide sequences are considered pathogenesis-related (PR) proteins and are mainly involved in the induction of resistance against pathogens^29^. Members of the thaumatin family have been reported to exhibit strong antifungal activity, participating in inhibition of mycelial or germ tube growth and in reduction of sporulation of many fungal pathogens in several fruits^30^. The results of this study indicated that thaumatin was strongly over-accumulated in ‘RG’ fruits exposed to either *M. fructicola* or *M. laxa* as well as in ‘RL’ fruit treated with *M. fructicola*. In support to this observation we found that *thaumatin expression* was strongly induced in ‘RG’ fruits inoculated with *M. laxa* compared to control, suggesting that thaumatin plays an important anti-disease role in peach fruit against brown rot. Meanwhile, thaumatin proteins have been reported to be up-accumulated in chilled-injury resistant cultivars^31^. Thus, the accumulation of thaumatin might be a common response cascade during both abiotc and biotic stimuli in peach fruit.

Heat shock proteins (HSP) are a multi-gene family of stress proteins that protect the cell’s functions in response to stress conditions^32^. In this study, the abundance of HSP70 was increased in ‘RG’ fruit that had been inoculated with either *M. fructicola* or *M. laxa*, providing evidence of their involvement in resistance to these pathogens. In plants, HSP70 exhibit a protective role against these stresses acting as a molecular chaperone and playing an important role in protein folding, intracellular trafficking of proteins and helping to refold proteins that were denatured due to abiotc cell stress^33^. Overwhelming data support that HSP70 is also an important molecular chaperone during biotic stress responses. Cytoplasmic *Capsicum annuum* HSP70 (CaHSP70) significantly accumulates in pepper leaves, inducing the hypersensitive response (HR) by *Xanthomonas campestris* pv. *vesicatoria* (*Xcv*) infection. CaHSP70 silencing in pepper was shown to increase susceptibility to *Xcv* as well as alter the cell death response to *Xcv* infection^34^.

Another interesting finding that emerged from this work is the fact that the ‘RG’ proteome status was differently modified by the two *Monilinia* species. For example, several proteins, such as major latex allergen and glutathione *S*-transferases (GST) were up-accumulated only in ‘RG’ fruits inoculated with *M. fructicola*, signifying their involvement in the defense of this cultivar to brown rot. In support to the role of GST in brown rot, our data disclosed that ‘RG’ fruit exposed to *M. fructicola* induced *GST* expression. GST’s are a large group of conserved enzymes, known for their ability to convert xenobiotic compounds to less reactive and more soluble substances, by facilitating their conjugation with glutathione and assisting to their discharge from the cells, via membrane-based glutathione conjugate pumps^35^. Several studies reviewed by Gullner *et al*.^36^, have shown that GSTs play a crucial role in plant-pathogen interactions, by suppressing ROS accumulation in the infected tissues of the host. In detail, it has been shown that infection of plant tissue by necrotrophic fungi leads to an up-regulation of GSTs that in subsequence, enhance the antioxidant plant system contributing to disease resistance to necrotrophic pathogens such as *Botrytis cinerea*, *Alternaria brassisicola* or *Sclerotinia sclerotiorum*^36,37^.

The present proteomic study also identified several proteins, such ascystathionine-β-synthase (CBS) domains containing protein (CDCP), *S*-formylglutathione hydrolase, subtilisin-like protease,4-hydroxy-3-methylbut-2-en-1-yl diphosphate synthase, inosine-5′-monophosphate dehydrogenase, peptidylprolyl isomerase, peptidylprolyl isomerase and dynamin-related protein that are specifically decreased by brown rot in the relatively susceptible ‘RL’ fruit. Particularly, the observation that CDCPs abundance was decreased in ‘RL’ fruits inoculated with both *M. fructicola* and *M. laxa*, which, given the role of this enzyme in the regulation of redox homeostasis under pathogenic infection^38–40^, prompts the question of whether this protein has an active function as a regulator of pathogen sensitivity in stressed plants.

Our proteomic analysis also provide evidence that *S*-formylglutathione hydrolase (SFGH) may be involved in fruit response to pathogen attack since we observed that this protein was down-regulated in ‘RL’ fruits inoculated with *M. laxa*. SFGH is a glutathione thiol esterase that hydrolyzes *S*-formylglutathione to glutathione and formate and participates in the formaldehyde detoxification pathway in plants^41^. However, we cannot speculate on its role in resistance to brown rot as there is little information regarding this protein. It is widely recognized that pathogen attack promotes the site-specific generation of reactive oxygen species (ROS) that can exacerbate the oxidative stress at the local site^42^. One of the consequences of the oxidative stress is lipid per-oxidation that might generate formaldehyde and other reactive lipid peroxidation products that can be provoke oxidative damage to peach fruit^27^. We speculate that these differences in the SFGH abundance between cultivars reflect the differences in the ROS-originated oxidative damage contribution to brown rot sensitivity.

This study also documents that *Monilinia* species increased few proteins in ‘RL’ which are not affected in the ‘RG’ fruit, and involve, formate dehydrogenase (FDH), UDP-glycosyltransferase (UGT) and 1-aminocyclopropane-1-carboxylate. In this sense, FDH abundance was increased in the artificially-inoculated ‘RL’ fruit compared to the non-inoculated fruit, suggesting that it might be involved in fruit responses to brown rot agents.

In higher plants formate dehydrogenase is a mitochondrial, NAD-dependent enzyme that catalyzes the oxidation of formate to carbon dioxide^43^. Formate results from photorespiratory glyoxylate and play a metabolic role as a signal in response to different stressful conditions^44^. Formate dehydrogenase is induced by several abiotic stresses such as hypoxia, wounding, chilling and heat^43^. There is convincing evidence that FDH may be involved in host-pathogen interactions^45,46^; however, the role of FDHs in defense response to pathogen attack remain unclear. It has been reported that *FDH1* participates in the cell death signaling pathway, defense-related hormone and gene regulation, leading to hypersensitive cell death and defense response against pathogens^47^. Particularly, in peach fruits it was evidenced that formate may derive from the conversion of methionine to ethylene as it was expressed in high levels during fruit ripening, coinciding with the start of the climacteric period^48–50^. Therefore, FDH1 may regulate diverse peach fruit defense responses and ripening syndrome.

Glycosylation plays an important role in the phenylpropanoids by enhancing their solubility and stability, facilitating their storage and accumulation in living cells, and regulating their chemical properties and bioactivities^51^. Glycosylation is regulated by UDP-glycosyltransferase, which catalyzes the transfer of a saccharide moiety from an activated glycosyl donor to a nucleophilic glycosyl acceptor molecule, establishing natural glysodic linkages^52^. Several lines of evidence show that phenylpropanoid compounds (i.e. chlorogenic acid) play a crucial role in peach resistance to *Monilinia* spp.^20,21,53^ while studies of UDP-glycosyl transferase in pathogen plant–pathogen interactions have revealed its function in disease resistance^54,55^. Despite the increase in UDP-glycotransferase abundance and expression in the tissues of the ‘RL’ fruit in the presence of *M. laxa* the fruit of this cultivar remained extremely susceptible to the pathogen, suggesting that other factors contribute to this increased susceptibility, remaining to be elucidated.

The abundance of the 1-aminocyclopropane-1-carboxylate deaminase was increased in fruits of ‘RL’ inoculated with *M. laxa*. Recent studies have shown that on climacteric fruits such as kiwifruit or apple, it degrades 1-aminocyclopropane-1-carboxylate (ACC)^56,57^. ACC constitutes the immediate precursor of ethylene and its degradation leads to a reduction in ethylene biosynthesis in these fruits. It is well established that ethylene plays a crucial role in the regulation of fruit defense against microbial pathogens^58^. Depending on the pathogen type (necrotrophic vs biotrophic pathogens) and the plant species, ethylene can promote disease development acting as a virulence factor, while, in other cases, ethylene modulates resistance responses acting as a signaling compound for resistance to the pathogens^59,60^. In several climacteric fruits has been shown that reduced ethylene production leads to an increase of their susceptibility to necrotrophic pathogens due to ethylene involvement into the regulation of defense-associated genes^60,61^. However, in a recent study aiming to explore the role of ethylene during *Monilinia* spp. infection has been proposed that brown rot agents try to suppress ethylene biosynthesis aiming to an inhibition of fruit defense reactions^62^. The different level of 1-aminocyclopropane-1-carboxylate deaminase in the present work might be due to the spatial and temporal action of ethylene that was produced both during fruit-pathogen interactions and peach fruit ripening process. An alternate action, that needs to be considered, is whether ethylene directly affects the growth of *Monilinia* spp. as speculated by Sharon *et al*.^63^ for *B. cinerea* in other types of fruits. A previous report suggested that applications of exogenous ethylene did not affect the development of *M. fructicola* on peach, nectarine and plum fruit^64^. However, it has not been known yet what is the effect of endogenous ethylene produced within the host tissues. Thus, further research is required to highlight the precise role of the 1-aminocyclopropane-1-carboxylate deaminase, notably the ethylene metabolism, as a mechanism to account for fruit responses to *Monilinia* spp.

This study provides the first information concerning the peach fruit proteomic response to infection by brown rot pathogens such as *M. fructicola* and *M. laxa*. It is also illustrated the differential accumulation of proteins in response to both *M. fructicola* and *M. laxa* in peach cultivars with different resistance levels against brown rot. The significant up-accumulation of several proteins associated with defense in the disease-resistance cultivar and the down-accumulation of the defense proteins in the susceptible cultivar, may explain their differences in disease resistance levels. Further research should be conducted to determine their precise role and relationship of these proteins in peach fruit defense against brown rot, thereby enabling our ability to control this disorder using knowledge-based new peach breeding programs.

## Methods

### Fruit samples

Fruit used in the study were collected from an experimental orchard in Imathia (North Greece). Two medium-early harvest peach (*Prunus persica*) cultivars, Rich Lady and Royal Glory, grafted onto GF-677 (*Prunus persica* × *Prunus amygdalus*) rootstock, trained in an open vase, were used. Healthy with no visual defect fruits were sampled at commercial harvest stage (firmness 36.5 ± 5 N) and transported immediately at the laboratory. All experimental fruit had received no previous fungicide applications during the vegetative period.

### Artificial inoculations

Two isolates of *M. fructicola* (Mf 101, Mf 160) and two isolates of *M. laxa* (Ml 1, Ml 66) were used for the artificial inoculations. Isolates were recovered from infected peaches of commercial orchards in Imathia region and identified to species level with a PCR assay developed by Hily *et al*.^65^. Artificial inoculation was conducted as described by Papavasileiou *et al*.^3^. Briefly, for conidia production, isolates were cultured in Petri dishes on V8 agar juice nutrient medium for 7 days at 25 °C, under continuous light. Five ml of sterile and distilled water were then added in each Petri dish, while the conidia were scraped off the cultures with a surgical scalpel. The conidial suspension was then filtered twice through a cheesecloth to withhold mycelia and adjusted to a concentration of 10^5^ spores ml^−1^ with a Neubauer Haemotocytometer. Before the inoculation, fruit were disinfected by dipping them in a 10% sodium chloride solution for 1 min and then rinsed twice using distilled water and let to dry. Fruit were then placed in sterilized plastic containers lined with wet paper towels. The inoculation was carried out by pipetting a drop of 20 μl spore solution on the un-wounded surface of each fruit. In total 15 replicate fruits (3 × 5 fruit, in 3 independent replications) of each peach cultivar were inoculated with each isolate. The containers were then sealed airtight with a plastic lid to ensure 100% relative humidity necessary for the infection. The fruit were incubated for 6 days at 22 °C. After the incubation period the disease incidence was determined by measuring the number of fruit showing typical brown rot symptoms. The observed frequencies (%) of diseased fruits of the 2 cultivars were compared using the chi-square test. The statistical analysis tests were performed using SPSS Statistics (version 11.0; IBM, NY).

### Plant material utilized for proteomic analysis

The experimental design included the following fruit classes: (i) fruit of the two cultivars artificially inoculated with *M. fructicola* isolates, (ii) fruit of the two cultivars artificially inoculated with *M. laxa* isolates, iii) fruit of the two cultivars inoculated with sterile water (control). For the proteomic analysis, samples were taken from the mesocarp of the artificially inoculated and control fruit after 6 days of incubation at 22 °C. Approximately 50–70 gr of skinless flesh of the mesocarp were carefully received from each fruit at 1 cm distance from the rotten tissue, to avoid the presence of mycelia in the proteomic analysis. Samples were cut into small pieces, placed in polyethylene bags (3 fruits per bag), frozen instantly in liquid nitrogen and stored at −80 °C until further analysis. Totally, 15 fruits (3 × 5 fruit in each replicate) were utilized for the analysis.

### Protein extraction

The extraction was performed according to a previous report^50^ following phenol-based extraction protocol. After pellet complete drying the proteins were solubilized in buffer containing 4% CHAPS (3-[(3-Cholamidopropyl) dimethylammonio]-1-propanesulfonate), 7 M urea, 2 M thiourea, 20 mM DTT, 0.5% ampholyte pH 3–10, 2% Triton X-100 and bromophenol blue. Protein concentrations were measured according to Bradford^66^ using bovine serum albumin (BSA; Sigma) as a standard.

### Two-dimensional gel electrophoresis and protein quantification

Proteins (50 μg) were separated by 2D-PAGE as described by Minas *et al*.^44^. Isoelectric focusing was run onto immobilized pH gradient gel strips (IPG strip pH 3–10 NL, 11 cm; Biorad) and then SDS–PAGE onto 12.5% polyacrylamide gels (Criterion Tris-HCl Precast Gels-Biorad). For each treatment, 2-D gels were run in triplicate and for three independent extractions. Following silver-nitrate stained 2-D gels, were scanned with a Bio-Rad GS-800 Calibrated Densitometer equipped with PDQuest Advanced 2-D Gel Analysis Software. Spots were detected, background subtracted, matched and quantitative determination of the spot volumes was performed (mode: total quantity of valid spots normalization) as reported earlier^44^. Individual means were compared using Student’s t-test (significance level 95%). To validate significant differences, Student’s t-test was further combined by the quantitative 2-fold change of spot volume.

### Protein identification by tryptic in-gel digestion, mass spectrometry and database searching

Cut silver-stained spots were destained and processed using classical tryptic-mediated in-gel digestion^67^. The generated peptide mixture was solubilized in 10 μl solution A (2% acetonitrile in 0.1% formic acid) and pre-concentrated with a flow of 5 μl/min for 10 min on a C18 trap column (Acclaim PepMap, Thermo Scientific) and then loaded onto a 50 cm long C18 column (75 μm ID, particle size 2 μm, 100 Å, Acclaim PepMap RSLC, Thermo Scientific). The binary pumps of the HPLC (Ultimate 3000, RSLCnano, Thermo Scientific) consisted of solution A (2% acetonitrile in 0.1% formic acid) and solution B (80% acetonitrile in 0.1% formic acid). The peptides were separated using a linear gradient from 4% B up to 40% in 40 min for a 1-h gradient run with a flow rate of 300 nl/min. The column was placed in an oven operating at 35 °C. The HPLC was coupled online to a LTQ Orbitrap XL Mass spectrometer (Thermo Scientific). Full scan MS spectra were acquired in the orbitrap (m/z 350–2000) in a profile mode and data-dependent acquisition with the resolution set to 60,000 at m/z 400 and automatic gain control target at 10^6^. The six most intense parental ions were sequentially isolated for collision-induced MS/MS fragmentation (CID) and their daughter fragments were detected in the linear ion trap. Dynamic exclusion was set to 1 minute and activated for 90 s. Ions with single charge states were excluded. Lock mass of m/z 445,120025 was used for internal calibration. The Xcalibur software (Thermo Scientific) was used to control the system and acquire the raw files. The raw files were analyzed with Proteome discoverer 1.4 (Thermo Scientific) using the complete Uniprot database of *Prunus persica* (3760, 28650 entries). Search parameters were strict trypsin specificity, allowing up to two missed cleavage sites. Oxidation of methionines, cysteine caramidomethylation as well as deamidation of asparagines and glutamines were set as variable modifications. The false discovery rate (FDR) was set to 5%. In case the *Prunus persica* protein identified was not yet annotated its sequence was processed through a BLAST-mediated search against current plant databases in order to find homologous and annotated proteins suggesting its potential function.

### RNA preparation and quantitative RT-PCR (RT-qPCR)

For some of the proteins that showed differential accumulation among treatments (peach cultivar or pathogen) a RT-qPCR was employed to study whether protein modulation profile follows gene expression pattern. Genes utilized in the RT-qPCR were thaumatin, UDP-glycoltransferase, major latex allergen, glutathione *S*-transferase, formate dehydrogenase, 1-aminocyclopropane-1-carboxylate deaminase, all involved in disease/defense mechanisms. Gene specific primers were designed based on the respective mRNA gene sequences deposited in GenBank (GenBank accession numbers NW_006760268.1, NW_006760201.1, NW_006760194.1, NW_006760268.1, NW_006760268.1 and NW_006760212.1, respectively). Primers were designed using the online software Primer3Plus^68^. All primers are listed in Table 1.

**Table 1:** List of primers used in the RT-qPCR assay.

Primer name	Nucleotidesequence (5′ – 3′)	Amplicon size (bp)	Accession number (GenBank)	Target gene
Thaum-Fw	ATGGAAGCGTAATTGCGTGC	92	NW_006760268.1	Thaumatin
Thaum-Rev	GCACTGGGTCTTGAAGAGCT			
UDP-Fw	ACATGCACCTGATGGCGTAA	103	NW_006760201.1	UDP-glycotransferase
UDP-Rev	GCTCGATAGCTGCATTGTGG			
Major-Fw	CTGTGGCAGGAAGTGGTAGG	111	NW_006760194.1	Major latex allergen
Major-Rev	ACCACACACGAATCCAACGT			
Glutath-Fw	CGTGAACGACATGTGAGGGA	107	NW_006760268.1	Glutathione-*S*-transferase
Glutath-Rev	AGCAAATTACAAGGGGCGGA			
Form-Fw	GCCCAGCACGGTAGAAAGGA	95	NW_006760268.1	Formate dehydrogenase
Form-Rev	GTCCTTTCGCATCGTGCACC			
Aminicycloprop-Fw	GATTGGGCTCTTGGCTGAGT			
Aminicycloprop-Rev Actin – Fw Actin - Rv NADH –Fw NADH - Rv	GACAGAAAAGGCACGGACCT TCAATGTGCCTGCCATGTAT AGCAAGGTCCAGACGAAGAA GTGGATGGGACAACTTGCTT GCAATTGCATCCACAATGTC	104 164 178	NW_006760212.1 XM_007211382.2 XM_020565692.1	1-aminocyclopropane-1 carboxyalte deaminase Actin-7 NADH dehydrogenase [ubiquinone]

RNA was extracted from fruit samples taken from the mesocarp of the artificially inoculated and control fruit after 72 h of incubation at 22 °C. Skinless pulp samples were removed from each fruit around the inoculation point using a cork borer and immediately frozen with liquid nitrogen and stored at −80 °C until use. Total RNA was extracted using the NucleoSpin RNA Plant kit following the manufacturer‘ protocol (Macherey-Nagel GmbH & Co. KG, Germany) and its concentration was measured using a P330 nanophotometer (Implen GmbH). The actin and ubiquinone genes were used as reference genes.

The qRT-PCR amplification conditions were as those described by Pappi *et al*.^69^. The 25 μl qRT-PCR reaction consisted of buffer 5x, 1.5 μl of EVA Green, 0.2 mM of each dNTP, 4 mM DTT, 1 U of Superscript III RNaseH−Reverse Transcriptase, 1.5 mM additional MgSO_4_ (Invitrogen-Life Technologies, Groningen, The Netherlands), 3 U of HotStartTaq DNA polymerase (Qiagen, Hilden, Germany) and 1 M of each primer. The thermal cycling conditions were the following: 50 °C for 30 min, followed by 95 °C for 12 min for cDNA synthesis and 40 cycles in 3 steps: (a) 30 s at 95 °C (denaturation), (b) 30 s at 56 °C (annealing) and (c) 20 s at 72 °C (extension). The fluorescence levels were measured at the end of each cycle. The assay was performed using the Mx3005Pro Real-Time PCR detection system (Strategen, USA). The analysis of fluorescence data was conducted using the Mx3005P Software (version 4.00 Build 367, USA). The Cq value for each gene was measured and the expression level of the genes in the different samples was calculated using the formula 2–ΔΔCq^70^.

## Supplementary information

Supplementary information.

## References

[1] Hong C, Holtz BA, Morgan DP, Michailides TJ (1997). Significance of thinned fruit as a source of secondary inoculums of *Monilinia fructicola* in California nectarine orchards. Plant Dis..

[2] Holb IJ, Szoke S, Abonyi F (2013). Temporal development and relationship amongst brown rot blossom blight, fruit blight and fruit rot in integrated and organic sour cherry orchards. Plant Pathol..

[3] Papavasileiou A, Karaoglanidis GS, Michailides TJ (2015). Intraspesific diversity of *Monilinia fructicola* and *M. laxa* populations from blossoms and fruit of different hosts in Greece. Plant Dis..

[4] Abate D (2018). Characterization of *Monilinia* spp. populations on stone fruit in South Italy. Plant Dis..

[5] Bosshard E, Hilber-Bodmer M, Schärer HJ, Bünter M, Duffy B (2006). First report of quarantine brown rot pathogen *Monilinia fructicola* on imported stone fruits in Switzerland. Plant Dis..

[6] De Cal A, Gell I, Usall J, Viñas I, Melgarejo P (2009). First report of brown rot caused by *Monilinia fructicola* in peach orchards in Ebro Valley, Spain. Plant Dis..

[7] Papavasileiou A, Testempasis S, Michailides TJ, Karaoglanidis GS (2015). Frequency of brown rot fungi on blossoms and fruit in stone fruit orchards in Greece. Plant Pathol..

[8] Lee MH, Bostock RM (2007). Fruit exocarp phenols in relation to quiescence and development of *Monilinia fructicola* infections in *Prunus* spp.: a role for cellular redox?. Phytopathology.

[9] Nanni V (2013). The peach (*Prunus persica*) defensin PpDFN1 displays antifungal activity through specific interactions with the membrane lipids. Plant Pathol..

[10] Obi VI, Barriuso JJ, Gogorcena Y (2018). Peach brown rot: still in search of an ideal management option. Agriculture.

[11] Gell I, De Cal A, Torres R, Usall J, Melgarejo P (2008). Relationship between the incidence of latent infections caused by *Monilinia* spp. and the incidence of brown rot of peach fruit: factors affecting latent infection. Eur. J. Plant Pathol..

[12] Ma Z, Yanga L, Yana H, Kennedy JF, Menga X (2013). Chitosan and oligochitosan enhance the resistance of peach fruit to brown rot. Carbohyd. Polym..

[13] Droby S, Wisniewski M, Macarisin D, Wilson C (2009). Twenty years of postharvest biocontrol research: Is it time for a new paradigm?. Postharvest Biol. Technol..

[14] Dias, M. C. Phytotoxicity: An Overview of the physiological responses of plants exposed to fungicides. *J. Bot*. 135479, 10.1155/2012/135479 (2012).

[15] Gradziel TM, Wang DC (1993). Evaluation of brown-rot resistance and its relation to enzymatic browning in clingstone peach germplasm. J. Am. Soc. Hortic. Sci..

[16] Martinez-Garcia PJ (2013). Application of genomic and quantitative genetic tools to identify candidate resistance genes for Brown Rot resistance in peach. PLoS One.

[17] Baro-Montel N (2019). Exploring sources of resistance to brown rot in an interspecific almond x peach population. J. Sci. Food Agric..

[18] Prusky D (1996). Pathogen quiescence in postharvest diseases. Annu. Rev. Phytopathol..

[19] Gradziel, T. M., Thorpe, M. A., Bostock, R. M. & Wilcox, S. Breeding for brown rot (*Monilinia fructicola*) resistance in clingstone peach with emphasis on the role of fruit phenolics. ISHS Acta Horticulturae 465: IV International Peach Symposium (1998).

[20] Lee MH, Bostock RM (2006). Induction, regulation, and role in pathogenesis of appressoria in *Monilinia fructicola*. Phytopathology.

[21] Villarino M, Sandin-Espana P, Melgarejo P, De Cal A (2011). High chlorogenic and neochlorogenic acid levels in immature peach reduce *Monilinia laxa* infection by interfering with fungal melanin biosynthesis. J. Agric. Food Chem..

[22] De Cal A (2013). Role of gluconic acid and pH modulation in virulence of *Monilinia fructicola* on peach fruit. Postharvest Biol. Technol..

[23] Mehta A (2008). Plant–pathogen interactions: what is proteomics telling us?. FEBS J..

[24] Tan K-C, Ipcho SVS, Trengove RD, Oliver RP, Solomon PS (2009). Assessing the impact of transcriptomics, proteomics and metabolomics on fungal phytopathology. Mol. Plant Pathol..

[25] Kaur, A., Kumar, A. & Sudhakara Reddy, M. Plant–pathogen interactions: A proteomic approach. (eds. Singh, R., Kothari, R., Koringa, P. & Singh S.) In *Understanding Host-Microbiome Interactions - An Omics Approach*. 207–225, (Springer, 2017).

[26] Bevan M (1998). Analysis of 1.9 Mb of contiguous sequence from chromosome 4 of *Arabidopsis thaliana*. Nature.

[27] Molassiotis A, Tanou G, Filippou P, Fotopoulos V (2013). Proteomics in the fruit tree science arena: New insights into fruit defense, development, and ripening. Proteomics.

[28] Hamamouch N, Li C, Seo PJ, Park CM, Davis EL (2011). Expression of Arabidopsis pathogenesis-related genes during nematode infection. Mol. Plant Pathol..

[29] Marra R (2006). Study of the three-way interaction between *Trichoderma atroviride*, plant and fungal pathogens by using a proteomic approach. Curr. Genet..

[30] Ho VSM, Wong JH, Ng TB (2007). A thaumatin-like antifungal protein from the emperor banana. Peptides.

[31] Nilo R (2010). Proteomic analysis of peach fruit mesocarp softening and chilling injury using difference gel electrophoresis (DIGE). BMC Genom..

[32] Tanou G (2015). The impact of sodium nitroprusside and ozone in kiwifruit ripening physiology: A combined gene and protein expression profiling approach. Ann. Bot.-London.

[33] Sung DY, Kaplan F, Guy CL (2001). Plant Hsp70 molecular chaperones: Protein structure, gene family, expression and function. Physiol. Plantarum.

[34] Kim NH, Hwang BK (2015). Pepper heat shock protein 70a interacts with the type III effector *AvrBsT* and triggers plant cell death and immunity. Plant Physiol..

[35] Ainalidou A (2016). Integrated analysis of metabolites and proteins reveal aspects of the tissue-specific function of synthetic cytokinin in kiwifruit development and ripening. J. Proteomics.

[36] Gullner G, Komives T, Király L, Schröder P (2018). Glutathione S-transferase enzymes in plant-pathogen interactions. Front. Plant Sci..

[37] Wagner U, Edwards R, Dixon DP, Mauch F (2002). Probing the diversity of the *Arabidopsis* glutathione S-transferase gene family. Plant Mol. Biol..

[38] Mou S (2015). Over-expression of rice CBS domain containing protein, OsCBSX3, confers rice resistance to *Magnaporthe oryzae* inoculation. *Int*. J. Mol. Sci..

[39] Frederickson Matika DE, Loake GJ (2014). Redox regulation in plant immune function. Antioxid. Redox Signal..

[40] Ok SH, Yoo KS, Shin JS (2012). CBSXs are sensor relay proteins sensing adenosine-containing ligands in Arabidopsis. Plant Signal. Behav..

[41] Yurimoto H, Lee B, Yano T, Sakai Y, Kato N (2003). Physiological role of *S*-formylglutathione hydrolase in C1 metabolism of the methylotrophic yeast *Candida boidinii*. Microbiology.

[42] Singh R (2016). Reactive oxygen species (ROS): Beneficial companions of plants’ developmental processes. Front. Plant Sci..

[43] Herman PL, Ramberg H, Baack RD, Markwell J, Osterman JC (2002). Formate dehydrogenase in *Arabidopsis thaliana*: overexpression and subcellular localization in leaves. Plant Sci..

[44] Minas IS, Tanou G, Karagiannis E, Belghazi M, Molassiotis A (2016). Coupling of physiological and proteomic analysis to understand the ethylene -and chilling-induced kiwifruit ripening syndrome. Front. Plant Sci..

[45] David P (2010). Three highly similar formate dehydrogenase genes located in the vicinity of the B4 resistance gene cluster are differentially expressed under biotic and abiotic stresses in *Phaseolus vulgaris*. Theor. Appl. Genet..

[46] Yang L (2011). Proteomic analysis of grapevine stem in response to *Xylella fastidiosa* inoculation. *Physiol*. Mol. Plant Pathol..

[47] Choi DS, Kim NH, Hwang BK (2014). Pepper mitochondrial formate dehydrogenase 1 regulates cell death and defense responses against bacterial pathogens. Plant Physiol..

[48] Prinsi B (2011). Peach fruit ripening: A proteomic comparative analysis of the mesocarp of two cultivars with different flesh firmness at two ripening stages. Phytochemistry.

[49] Karagiannis E (2016). Comparative physiological and proteomic analysis reveal distinct regulation of peach skin quality traits by altitude. Front. Plant Sci..

[50] Tanou G (2017). Exploring priming responses involved in peach fruit acclimation to cold stress. Sci. Rep..

[51] Le Roy J, Huss B, Creach A, Hawkins S, Neutelings G (2016). Glycosylation is a major regulator of phenylpropanoid availability and biological activity in plants. Front. Plant Sci..

[52] Li Y, Baldauf S, Lim EK, Bowles DJ (2001). Phylogenetic analysis of the UDP-glycosyltransferase multigene family of *Arabidopsis thaliana*. J. Biol. Chem..

[53] Guidarelli M (2014). Gene expression analysis of peach fruit at different growth stages and with different susceptibility to *Monilinia laxa*. Eur. J. Plant Pathol..

[54] Chong J (2002). Downregulation of a pathogen-responsive tobacco UDP-Glc:phenylpropanoid glucosyltransferase reduces scopoletin glucoside accumulation, enhances oxidative stress, and weakens virus resistance. Plant Cell.

[55] Ha X, Koopmann B, von Tiedemann A (2016). Wheat blast and fusarium head blight display contrasting interaction patterns on ears of wheat genotypes differing in resistance. Phytopathology.

[56] Karagiannis E (2018). A. Ethylene –dependent and –independent superficial scald resistance mechanisms in ‘Granny Smith’ apple fruit. Sci. Rep..

[57] Minas IS (2018). Ozone-induced inhibition of kiwifruit ripening is amplified by 1-methylcyclopropene and reversed by exogenous ethylene. BMC Plant Biol..

[58] van Loon LC, Geraats BPJ, Linthoorst HJM (2006). Ethylene as a modulator of disease resistance in plants. Trends Plant Sci.,.

[59] Broekaert WF, Delauré SL, De Bolle MF, Cammue BP (2006). The role of ethylene in host-pathogen interactions. Annu. Rev. Phytopathol..

[60] Alkan N, Fortes AM (2015). Insights into molecular and metabolic events associated with fruit response to post-harvest fungal pathogens. Front. Plant Sci..

[61] Marcos JF, González-Candelas L, Zacarías L (2005). Involvement of ethylene biosynthesis and perception in the susceptibility of citrus fruits to *Penicillium digitatum* infection and the accumulation of defense-related mRNAs. J. Exp. Bot..

[62] Baro-Montel N (2019). Double-sided battle: The role of ethylene during *Monilinia* spp. infection in peach at different phenological stages. Plant Physiol. Biochem..

[63] Sharon, A., Elad, Y., Barakat, R. & Tudzynski, P. Phytohormones in Botrytis–plant interactions. (eds. Elad, Y., Williamson, B., Tudzynski, P. & Delen, N.) In Botrytis: Biology, Pathology and Control. 163–179 (Springer, 2004).

[64] Palou L, Crisosto CH, Garner D, Basinal LM (2003). Effect of continuous exposure to exogenous ethylene during cold storage on postharvest decay development and quality attributes of stone fruits and table grapes. Postharvest Biol. Technol..

[65] Hily JM, Singer SD, Villani SΜ, Cox KD (2011). Characterization of the cytochrome b (*cyt*b) gene from *Monilinia* species causing brown rot of stone and pome fruit and its significance in the development of QoI resistance. *Pest*. Manage. Sci..

[66] Bradford MM (1976). A rapid and sensitive method for the quantitation of microgram quantities of protein utilizing the principle of protein-dye binding. Anal. Biochem..

[67] Shevchenko A (2006). In-gel digestion for mass spectrometric characterization of proteins and proteomes. Nat. Protoc..

[68] Untergasser, A. *et al*. Primer3Plus, an enhanced web interface to Primer3. *Nucleic Acids Res*. **35**, 10.1093/nar/gkm306 (2007). 10.1093/nar/gkm306 PMC1933133 17485472

[69] Pappi P (2015). Development of one-tube real-time qRT-PCR and evaluation of RNA extraction methods for the detection of Eggplant mottled dwarf virus indifferent species. J. Virol. Methods.

[70] Livak KJ, Schmittgen TD (2001). Analysis of relative gene expression data using real-time quantitative. PCR. Methods.

