# Supplementary material for: Proteomic analysis upon peach fruit infection with Monilinia fructicola and M. laxa identify responses contributing to brown rot resistance
Source: Sci Rep. 2020 May 8;10:7807. doi: 10.1038/s41598-020-64864-x (PMC7210933; doi:10.1038/s41598-020-64864-x)
Supplement: Supplementary file 1 — Supplementary information. [file 41598_2020_64864_MOESM1_ESM.docx]

**"Supplementary Materials of “Proteomic analysis upon peach fruit infection with *Monilinia fructicola* and *M. laxa* identify responses contributing to brown rot resistance”, by Antonios Papavasileiou, Georgia Tanou, Anastasios Samaras, Martina Samiotaki, Athanassios Molassiotis, George Karaoglanidis"**

**Table S1.** Identification details of differentiable proteins in the mesocarp of the Rich Lady and Royal Glory peach (*Prunus persica)* cultivars after their inoculation with *Monilinia fructicola* and *M. laxa* isolates.

| **Spot No^a^** | **Accession Number^b^** | **Suggested Name^c^** | **Description** | **Matching citeria^d^** | **Organism^e^** | **Subcellular localization^f^** | **Functinal Category^g^** | **SEQUEST Score** | **% Coverage** | **# Proteins** | **# Unique Peptides** | **# Peptides** | **# PSMs** | **# AAs** | **MW [kDa]** | **calc. pI** | **Peptide confidence** | **Sequence** | **# PSMs** | **Modifications** | **ΔCn** | **XCorr** | **Charge** | **MH+ [Da]** | **ΔM [ppm]** | **RT [min]** |
| --- | --- | --- | --- | --- | --- | --- | --- | --- | --- | --- | --- | --- | --- | --- | --- | --- | --- | --- | --- | --- | --- | --- | --- | --- | --- | --- |
| **13** | M5WGH9 | plastid-lipid-associated protein | Uncharacterized protein OS=Prunus persica GN=PRUPE_ppa005787mg PE=4 SV=1 - [M5WGH9_PRUPE] | 97% to GI:645236410 Prunus persica, E value 0 | Prunus persica | Chloroplast | 11.05-Disease/Defense/Stress responses | 118,83 | 23,20 | 2 | 9 | 10 | 61 | 444 | 48,8 | 6,74 | High | ETESAVAVAEDDKPAEPESAETSR | 8 |  | 0,0000 | 4,74 | 3 | 2518,14548 | 1,04 | 41,44 |
|  |  |  |  |  |  |  |  |  |  |  |  |  |  |  |  |  | High | GDNGSVFVLIK | 2 |  | 0,0000 | 3,35 | 2 | 1148,63054 | -0,45 | 49,50 |
|  |  |  |  |  |  |  |  |  |  |  |  |  |  |  |  |  | High | ALVDSFYGTDR | 10 |  | 0,0000 | 2,90 | 2 | 1243,59587 | 0,39 | 50,33 |
|  |  |  |  |  |  |  |  |  |  |  |  |  |  |  |  |  | High | GDnGSVFVLIK | 2 | N3(Deamidated) | 0,0000 | 2,88 | 2 | 1149,61504 | -0,03 | 51,20 |
|  |  |  |  |  |  |  |  |  |  |  |  |  |  |  |  |  | High | vVDEDEWGPEK | 3 | N-Term(Acetyl) | 0,0000 | 2,79 | 2 | 1344,59587 | 0,29 | 50,61 |
|  |  |  |  |  |  |  |  |  |  |  |  |  |  |  |  |  | High | SLLTSVQDTASSVVK | 1 |  | 0,0000 | 2,62 | 2 | 1534,83330 | 0,62 | 48,80 |
|  |  |  |  |  |  |  |  |  |  |  |  |  |  |  |  |  | High | VVDEDEWGPEK | 12 |  | 0,0000 | 2,34 | 2 | 1302,58562 | 0,54 | 50,93 |
|  |  |  |  |  |  |  |  |  |  |  |  |  |  |  |  |  | High | LDLTVFK | 5 |  | 0,0000 | 2,29 | 2 | 835,49254 | 0,14 | 49,22 |
|  |  |  |  |  |  |  |  |  |  |  |  |  |  |  |  |  | High | FSISnSK | 2 | N5(Deamidated) | 0,0000 | 2,26 | 2 | 783,38823 | -0,14 | 39,90 |
|  |  |  |  |  |  |  |  |  |  |  |  |  |  |  |  |  | Medium | TISSQPPFK | 4 |  | 0,0000 | 1,68 | 2 | 1004,54100 | -0,16 | 42,27 |
|  |  |  |  |  |  |  |  |  |  |  |  |  |  |  |  |  | Medium | FSISNSK | 1 |  | 0,0000 | 1,61 | 2 | 782,40422 | -0,13 | 38,76 |
|  |  |  |  |  |  |  |  |  |  |  |  |  |  |  |  |  | Medium | GTLPLVK | 10 |  | 0,0000 | 1,30 | 2 | 727,47161 | 0,43 | 42,95 |
|  |  |  |  |  |  |  |  |  |  |  |  |  |  |  |  |  | Medium | KALVDSFYGTDR | 1 |  | 0,1517 | 1,23 | 3 | 1371,68955 | -0,59 | 43,74 |
| **19** | M5WVK0 | major latex allergen | Uncharacterized protein OS=Prunus persica GN=PRUPE_ppa012576mg PE=4 SV=1 - [M5WVK0_PRUPE] | 65% to GI:657984121 Malus domestica, E value 1e-18 | Prunus persica | Cytoplasm | 11.02-Disease/defense/Defense-related | 122,89 | 82,82 | 1 | 8 | 8 | 37 | 163 | 17,0 | 3,87 | High | TPEEPVAAAPAAVTESEAATTEEPK | 8 |  | 0,0000 | 5,71 | 3 | 2496,20212 | 1,28 | 45,19 |
|  |  |  |  |  |  |  |  |  |  |  |  |  |  |  |  |  | High | TEEKTPEEPVAAAPAAVTESEAATTEEPK | 1 |  | 0,0000 | 4,58 | 3 | 2983,43094 | 1,40 | 42,81 |
|  |  |  |  |  |  |  |  |  |  |  |  |  |  |  |  |  | High | AAEAEAEEPAATETEK | 7 |  | 0,0000 | 3,84 | 2 | 1646,74028 | 0,62 | 37,09 |
|  |  |  |  |  |  |  |  |  |  |  |  |  |  |  |  |  | High | EVTAAEPVAAVAEETK | 7 |  | 0,0000 | 3,60 | 2 | 1614,82366 | 0,90 | 45,06 |
|  |  |  |  |  |  |  |  |  |  |  |  |  |  |  |  |  | High | EETTESAETPAAPPVEEEKPEETTTDVPVEK | 3 |  | 0,0000 | 3,53 | 3 | 3369,56504 | 1,70 | 42,86 |
|  |  |  |  |  |  |  |  |  |  |  |  |  |  |  |  |  | High | AAEAEAEEPAATETEKTEAEEPK | 6 |  | 0,0000 | 2,93 | 2 | 2431,10381 | 1,73 | 38,48 |
|  |  |  |  |  |  |  |  |  |  |  |  |  |  |  |  |  | Medium | ETTPVEAEAEAEAPAAPEAEAPVPAEVETK | 2 |  | 0,0000 | 2,21 | 3 | 3033,44529 | 0,94 | 45,97 |
|  |  |  |  |  |  |  |  |  |  |  |  |  |  |  |  |  | High | TVEVIKTEEK | 3 |  | 0,0000 | 2,11 | 2 | 1175,65141 | -0,37 | 36,81 |
| **20** | M5XHX6 | 1,2-dihydroxy-3-keto-5-methylthiopentene dioxygenase | 1,2-dihydroxy-3-keto-5-methylthiopentene dioxygenase OS=Prunus persica GN=PRUPE_ppa010941mg PE=3 SV=1 - [M5XHX6_PRUPE] |  | Prunus persica | Chloroplast | 01.01-Metabolism/Amino Acid | 58,23 | 39,30 | 1 | 8 | 8 | 24 | 229 | 26,9 | 5,82 | High | LDADNYETDEELK | 5 |  | 0,0000 | 4,09 | 2 | 1554,68205 | 0,90 | 43,95 |
|  |  |  |  |  |  |  |  |  |  |  |  |  |  |  |  |  | High | EEVIQAWYMDDSDEDQR | 2 |  | 0,0000 | 3,72 | 2 | 2128,88042 | 1,84 | 48,96 |
|  |  |  |  |  |  |  |  |  |  |  |  |  |  |  |  |  | High | GYSYMDFcEVSPEK | 2 | C8(Carbamidomethyl) | 0,0000 | 3,44 | 2 | 1711,69963 | 1,01 | 47,81 |
|  |  |  |  |  |  |  |  |  |  |  |  |  |  |  |  |  | High | LDADNYETDEELKK | 5 |  | 0,0000 | 3,27 | 2 | 1682,77666 | 0,62 | 39,17 |
|  |  |  |  |  |  |  |  |  |  |  |  |  |  |  |  |  | High | GYSYmDFcEVSPEK | 3 | M5(Oxidation); C8(Carbamidomethyl) | 0,0000 | 2,74 | 2 | 1727,69536 | 1,48 | 44,51 |
|  |  |  |  |  |  |  |  |  |  |  |  |  |  |  |  |  | High | YAVAGSGYFDVR | 1 |  | 0,0000 | 2,60 | 2 | 1304,62737 | 0,26 | 45,55 |
|  |  |  |  |  |  |  |  |  |  |  |  |  |  |  |  |  | High | FTLDTNNYIK | 2 |  | 0,0000 | 2,53 | 2 | 1228,62114 | 0,23 | 46,49 |
|  |  |  |  |  |  |  |  |  |  |  |  |  |  |  |  |  | High | LPNYEEK | 2 |  | 0,0000 | 2,31 | 2 | 892,44042 | -0,77 | 37,65 |
|  |  |  |  |  |  |  |  |  |  |  |  |  |  |  |  |  | High | EEVIQAWYmDDSDEDQR | 1 | M9(Oxidation) | 0,0000 | 2,02 | 2 | 2144,87505 | 1,69 | 48,22 |
|  |  |  |  |  |  |  |  |  |  |  |  |  |  |  |  |  | Medium | AFVHKETGDHAVDAAA | 1 |  | 0,0000 | 1,42 | 3 | 1638,78696 | -0,11 | 36,22 |
| **407** | M5WXC0 | alanine aminotransferase | Uncharacterized protein OS=Prunus persica GN=PRUPE_ppa003850mg PE=4 SV=1 - [M5WXC0_PRUPE] | 99% to GI:645243725 Prunus mume, E value 0 | Prunus mume | Chloroplast | 01.01-Metabolism/Amino acid | 25,68 | 14,15 | 1 | 7 | 7 | 11 | 544 | 59,6 | 6,15 | High | VGDESYELYcAEK | 2 | C10(Carbamidomethyl) | 0,0000 | 3,12 | 2 | 1562,66924 | 0,81 | 43,57 |
|  |  |  |  |  |  |  |  |  |  |  |  |  |  |  |  |  | High | DTIAAGIEAR | 2 |  | 0,0000 | 2,72 | 2 | 1016,53703 | -0,12 | 42,36 |
|  |  |  |  |  |  |  |  |  |  |  |  |  |  |  |  |  | High | GEIVTLAQR | 2 |  | 0,0000 | 2,66 | 2 | 986,56285 | -0,12 | 41,88 |
|  |  |  |  |  |  |  |  |  |  |  |  |  |  |  |  |  | High | AAEAANTAPDAFYcR | 2 | C14(Carbamidomethyl) | 0,0000 | 2,65 | 2 | 1627,71807 | 0,65 | 42,66 |
|  |  |  |  |  |  |  |  |  |  |  |  |  |  |  |  |  | Medium | SETQGLFSADAIER | 1 |  | 0,0000 | 1,84 | 2 | 1523,73393 | 0,15 | 46,87 |
|  |  |  |  |  |  |  |  |  |  |  |  |  |  |  |  |  | Medium | IPAIVTR | 1 |  | 0,0000 | 1,77 | 2 | 769,49352 | 0,55 | 41,34 |
|  |  |  |  |  |  |  |  |  |  |  |  |  |  |  |  |  | Medium | cTILPQEDK | 1 | C1(Carbamidomethyl) | 0,0061 | 1,64 | 2 | 1103,54033 | 0,13 | 40,54 |
| **812** | M5Y2Q5 | patellin | Uncharacterized protein (Fragment) OS=Prunus persica GN=PRUPE_ppa023884mg PE=4 SV=1 - [M5Y2Q5_PRUPE] | 79% to GI:470127731 Fragaria vesca subsp. vesca, E value 0 | Fragaria vesca subsp. vesca | Cytoplasm | 08.07-Intracellular traffic/Vesicular | 140,76 | 34,93 | 2 | 22 | 22 | 87 | 584 | 65,3 | 4,87 | High | KAEEEPKAEVAAAEEPK | 2 |  | 0,0000 | 3,20 | 3 | 1825,91690 | -0,55 | 37,66 |
|  |  |  |  |  |  |  |  |  |  |  |  |  |  |  |  |  | High | AEVAAAEEPK | 3 |  | 0,0000 | 3,13 | 2 | 1014,50981 | -0,46 | 37,28 |
|  |  |  |  |  |  |  |  |  |  |  |  |  |  |  |  |  | High | HEFTAPPTPPPAK | 5 |  | 0,0000 | 3,10 | 2 | 1389,71684 | 0,47 | 39,61 |
|  |  |  |  |  |  |  |  |  |  |  |  |  |  |  |  |  | High | KVAPADEPVISNSYK | 4 |  | 0,0000 | 3,08 | 2 | 1617,85015 | 1,12 | 39,64 |
|  |  |  |  |  |  |  |  |  |  |  |  |  |  |  |  |  | High | EESYVVGELPEPQK | 1 |  | 0,0000 | 2,93 | 2 | 1603,78594 | 0,55 | 46,28 |
|  |  |  |  |  |  |  |  |  |  |  |  |  |  |  |  |  | High | IIAQSVSFK | 3 |  | 0,0000 | 2,79 | 2 | 992,57762 | 0,07 | 42,46 |
|  |  |  |  |  |  |  |  |  |  |  |  |  |  |  |  |  | High | VVLTIDNQSSK | 5 |  | 0,0000 | 2,79 | 2 | 1203,65813 | 0,11 | 43,56 |
|  |  |  |  |  |  |  |  |  |  |  |  |  |  |  |  |  | High | VAPADEPVISNSYK | 5 |  | 0,0000 | 2,50 | 2 | 1489,75456 | 0,81 | 41,84 |
|  |  |  |  |  |  |  |  |  |  |  |  |  |  |  |  |  | High | mISPFLTQR | 3 | M1(Oxidation) | 0,0000 | 2,45 | 2 | 1108,58183 | -0,15 | 45,92 |
|  |  |  |  |  |  |  |  |  |  |  |  |  |  |  |  |  | High | ELYQNTFTDEEKR | 5 |  | 0,0000 | 2,45 | 2 | 1672,78313 | 1,06 | 40,90 |
|  |  |  |  |  |  |  |  |  |  |  |  |  |  |  |  |  | High | MISPFLTQR | 5 |  | 0,0000 | 2,36 | 2 | 1092,58696 | -0,11 | 46,91 |
|  |  |  |  |  |  |  |  |  |  |  |  |  |  |  |  |  | High | VVFTHGVDK | 6 |  | 0,0000 | 2,31 | 2 | 1001,54106 | -0,44 | 38,33 |
|  |  |  |  |  |  |  |  |  |  |  |  |  |  |  |  |  | High | SAETLFK | 5 |  | 0,0000 | 2,27 | 2 | 795,42461 | -0,15 | 43,79 |
|  |  |  |  |  |  |  |  |  |  |  |  |  |  |  |  |  | High | QLIQEALNK | 3 |  | 0,0000 | 2,25 | 2 | 1056,60430 | -0,51 | 42,21 |
|  |  |  |  |  |  |  |  |  |  |  |  |  |  |  |  |  | High | FVFAGPSK | 5 |  | 0,0000 | 2,15 | 2 | 852,46117 | -0,34 | 45,22 |
|  |  |  |  |  |  |  |  |  |  |  |  |  |  |  |  |  | High | ELYQNTFTDEEK | 4 |  | 0,0000 | 2,14 | 2 | 1516,68205 | 1,20 | 46,22 |
|  |  |  |  |  |  |  |  |  |  |  |  |  |  |  |  |  | High | SDVVLLK | 2 |  | 0,0000 | 2,13 | 2 | 773,47661 | -0,21 | 42,63 |
|  |  |  |  |  |  |  |  |  |  |  |  |  |  |  |  |  | High | DAFAMIK | 5 |  | 0,0000 | 2,09 | 2 | 795,40703 | 0,05 | 48,55 |
|  |  |  |  |  |  |  |  |  |  |  |  |  |  |  |  |  | Medium | VKDAFAMIK | 1 |  | 0,0000 | 2,01 | 3 | 1022,57032 | -0,06 | 42,42 |
|  |  |  |  |  |  |  |  |  |  |  |  |  |  |  |  |  | High | DAFAmIK | 6 | M5(Oxidation) | 0,0000 | 2,01 | 2 | 811,40166 | -0,30 | 42,62 |
|  |  |  |  |  |  |  |  |  |  |  |  |  |  |  |  |  | Medium | EGPPEAEAEAVAEKPVAEEK | 3 |  | 0,0000 | 1,86 | 3 | 2080,00815 | -0,02 | 43,36 |
|  |  |  |  |  |  |  |  |  |  |  |  |  |  |  |  |  | Medium | IQFLEK | 2 |  | 0,0000 | 1,84 | 2 | 777,45049 | -0,08 | 43,59 |
|  |  |  |  |  |  |  |  |  |  |  |  |  |  |  |  |  | Medium | VKDAFAmIK | 1 | M7(Oxidation) | 0,0000 | 1,77 | 3 | 1038,56540 | 0,10 | 39,40 |
|  |  |  |  |  |  |  |  |  |  |  |  |  |  |  |  |  | Medium | ALEELK | 2 |  | 0,0000 | 1,71 | 2 | 702,40325 | -0,04 | 41,00 |
|  |  |  |  |  |  |  |  |  |  |  |  |  |  |  |  |  | Medium | EGEQEFTTSDPVTEITVKPATK | 1 |  | 0,0000 | 1,41 | 3 | 2407,18961 | 0,83 | 44,77 |
| **1013** | M5XG10 | Proteasome subunit beta type | Proteasome subunit beta type OS=Prunus persica GN=PRUPE_ppa010791mg PE=3 SV=1 - [M5XG10_PRUPE] |  | Prunus persica | Chloroplast | 06.13-Protein destination and storage/Proteolysis | 26,50 | 28,39 | 2 | 7 | 7 | 11 | 236 | 25,4 | 5,21 | High | TVVINSEGVTR | 2 |  | 0,0000 | 3,13 | 2 | 1174,64275 | 0,06 | 40,45 |
|  |  |  |  |  |  |  |  |  |  |  |  |  |  |  |  |  | High | TSTGVYVANR | 1 |  | 0,0000 | 2,82 | 2 | 1067,54753 | -0,47 | 38,13 |
|  |  |  |  |  |  |  |  |  |  |  |  |  |  |  |  |  | High | DEAEQLVVK | 1 |  | 0,0000 | 2,81 | 2 | 1030,54119 | -0,37 | 42,27 |
|  |  |  |  |  |  |  |  |  |  |  |  |  |  |  |  |  | High | SGSAADSQVVSDYVR | 2 |  | 0,0000 | 2,68 | 2 | 1540,72539 | 1,00 | 42,87 |
|  |  |  |  |  |  |  |  |  |  |  |  |  |  |  |  |  | High | AVSLAIAR | 2 |  | 0,0000 | 2,12 | 2 | 800,49858 | -0,41 | 42,32 |
|  |  |  |  |  |  |  |  |  |  |  |  |  |  |  |  |  | Medium | DEAEqLVVK | 1 | Q5(Deamidated) | 0,0000 | 1,71 | 2 | 1031,51897 | -6,42 | 43,19 |
|  |  |  |  |  |  |  |  |  |  |  |  |  |  |  |  |  | Medium | VcANLVR | 1 | C2(Carbamidomethyl) | 0,0000 | 1,66 | 2 | 831,45036 | -0,25 | 38,80 |
|  |  |  |  |  |  |  |  |  |  |  |  |  |  |  |  |  | Medium | LLSYGNK | 1 |  | 0,0000 | 1,61 | 2 | 794,44042 | -0,36 | 39,31 |
| **1109** | M5WB91 | malonyl-CoA-acyl carrier protein transacylase | Uncharacterized protein OS=Prunus persica GN=PRUPE_ppa007010mg PE=4 SV=1 - [M5WB91_PRUPE] | 98% to GI:645215431 Prunus mume, E value 0 | Prunus mume | Mitochondrion | 01.06-Metabolism/Lipid and sterol | 40,57 | 23,83 | 1 | 9 | 9 | 18 | 386 | 40,9 | 8,12 | High | VQQLcDAANQEVDDANK | 2 | C5(Carbamidomethyl) | 0,0000 | 4,20 | 2 | 1917,86357 | 1,45 | 40,90 |
|  |  |  |  |  |  |  |  |  |  |  |  |  |  |  |  |  | High | SAMVSVIGLDSDK | 2 |  | 0,0000 | 3,67 | 2 | 1321,66753 | 0,50 | 47,84 |
|  |  |  |  |  |  |  |  |  |  |  |  |  |  |  |  |  | High | GEAMQEAADAAK | 2 |  | 0,0000 | 3,10 | 2 | 1191,53008 | -0,87 | 38,19 |
|  |  |  |  |  |  |  |  |  |  |  |  |  |  |  |  |  | High | LRGEAmQEAADAAK | 2 | M6(Oxidation) | 0,0000 | 2,75 | 3 | 1476,71143 | 0,14 | 36,51 |
|  |  |  |  |  |  |  |  |  |  |  |  |  |  |  |  |  | High | LRGEAMQEAADAAK | 1 |  | 0,0000 | 2,57 | 3 | 1460,71534 | -0,66 | 38,39 |
|  |  |  |  |  |  |  |  |  |  |  |  |  |  |  |  |  | High | SYELGPGK | 1 |  | 0,0000 | 2,28 | 2 | 850,43028 | -0,30 | 39,02 |
|  |  |  |  |  |  |  |  |  |  |  |  |  |  |  |  |  | High | QVTSPVQWETTVK | 2 |  | 0,0000 | 2,23 | 2 | 1502,78545 | 0,30 | 46,09 |
|  |  |  |  |  |  |  |  |  |  |  |  |  |  |  |  |  | High | LAATQIR | 2 |  | 0,0000 | 2,22 | 2 | 772,46752 | -0,11 | 37,79 |
|  |  |  |  |  |  |  |  |  |  |  |  |  |  |  |  |  | Medium | EAQSVPAAAELYK | 2 |  | 0,0000 | 1,62 | 2 | 1376,70659 | 0,66 | 44,34 |
|  |  |  |  |  |  |  |  |  |  |  |  |  |  |  |  |  | Medium | VIAGIVK | 2 |  | 0,0000 | 1,59 | 2 | 699,47637 | -0,02 | 40,47 |
| **1119** | Q43618 | 1-aminocyclopropane 1-carboxylic acid oxidase | 1-aminocyclopropane 1-carboxylic acid oxidase OS=Prunus persica GN=PAO1 PE=2 SV=1 - [Q43618_PRUPE] |  | Prunus persica | Cytoplasm | 11.05-Disease/Defense/Stress responses | 175,29 | 58,62 | 10 | 4 | 20 | 108 | 319 | 36,1 | 5,30 | High | SNISEVPDLEDQYR | 8 |  | 0,0000 | 3,81 | 2 | 1664,77788 | 0,96 | 45,36 |
|  |  |  |  |  |  |  |  |  |  |  |  |  |  |  |  |  | High | KAFYGTnGPTFGTK | 2 | N7(Deamidated) | 0,0000 | 3,55 | 2 | 1489,73076 | -0,98 | 42,11 |
|  |  |  |  |  |  |  |  |  |  |  |  |  |  |  |  |  | High | AFYGTNGPTFGTK | 10 |  | 0,0000 | 3,34 | 2 | 1360,65422 | 0,72 | 45,27 |
|  |  |  |  |  |  |  |  |  |  |  |  |  |  |  |  |  | High | AFYGTnGPTFGTK | 4 | N6(Deamidated) | 0,0000 | 3,27 | 2 | 1361,63909 | 1,34 | 46,29 |
|  |  |  |  |  |  |  |  |  |  |  |  |  |  |  |  |  | High | AHTDAGGLILLFQDDK | 2 |  | 0,0000 | 3,25 | 3 | 1713,88089 | 0,11 | 50,36 |
|  |  |  |  |  |  |  |  |  |  |  |  |  |  |  |  |  | High | SVEHRVIAQTDGTR | 1 |  | 0,0000 | 2,70 | 3 | 1568,81321 | -0,51 | 35,84 |
|  |  |  |  |  |  |  |  |  |  |  |  |  |  |  |  |  | High | FKELVASK | 6 |  | 0,0000 | 2,66 | 2 | 921,53996 | -0,52 | 38,21 |
|  |  |  |  |  |  |  |  |  |  |  |  |  |  |  |  |  | High | DGQWIDVPPMR | 11 |  | 0,0000 | 2,61 | 2 | 1313,63091 | 0,11 | 50,87 |
|  |  |  |  |  |  |  |  |  |  |  |  |  |  |  |  |  | High | KAFYGTNGPTFGTK | 4 |  | 0,0000 | 2,57 | 3 | 1488,74796 | -0,17 | 40,78 |
|  |  |  |  |  |  |  |  |  |  |  |  |  |  |  |  |  | High | ELVASKGLEAVK | 1 |  | 0,0000 | 2,51 | 2 | 1243,72527 | -0,35 | 40,38 |
|  |  |  |  |  |  |  |  |  |  |  |  |  |  |  |  |  | High | VSGLQLLK | 3 |  | 0,0000 | 2,45 | 2 | 857,54564 | 0,14 | 45,91 |
|  |  |  |  |  |  |  |  |  |  |  |  |  |  |  |  |  | High | FVFEDYMK | 8 |  | 0,0000 | 2,41 | 2 | 1078,49138 | -0,06 | 48,31 |
|  |  |  |  |  |  |  |  |  |  |  |  |  |  |  |  |  | High | DGQWIDVPPmR | 6 | M10(Oxidation) | 0,0000 | 2,40 | 2 | 1329,62566 | -0,01 | 45,85 |
|  |  |  |  |  |  |  |  |  |  |  |  |  |  |  |  |  | High | TEVNDMDWESTFYLR | 1 |  | 0,0000 | 2,35 | 2 | 1905,83293 | 0,26 | 51,55 |
|  |  |  |  |  |  |  |  |  |  |  |  |  |  |  |  |  | High | TEVNDmDWESTFYLR | 1 | M6(Oxidation) | 0,0000 | 2,29 | 2 | 1921,83074 | 1,76 | 50,29 |
|  |  |  |  |  |  |  |  |  |  |  |  |  |  |  |  |  | High | VSNYPPcPNPELIK | 13 | C7(Carbamidomethyl) | 0,0000 | 2,25 | 2 | 1627,81658 | 1,02 | 44,38 |
|  |  |  |  |  |  |  |  |  |  |  |  |  |  |  |  |  | High | NVMKEFALK | 1 |  | 0,0000 | 2,21 | 2 | 1079,59148 | -0,34 | 42,74 |
|  |  |  |  |  |  |  |  |  |  |  |  |  |  |  |  |  | High | FVFEDYmK | 8 | M7(Oxidation) | 0,0000 | 2,14 | 2 | 1094,48638 | 0,02 | 48,20 |
|  |  |  |  |  |  |  |  |  |  |  |  |  |  |  |  |  | High | mENFPIINLEGLNGEGR | 1 | N-Term(Acetyl) | 0,0000 | 2,02 | 2 | 1944,95281 | 2,22 | 57,01 |
|  |  |  |  |  |  |  |  |  |  |  |  |  |  |  |  |  | Medium | AVETNISLGPIATA | 2 |  | 0,0000 | 1,83 | 2 | 1356,73711 | 0,09 | 51,37 |
|  |  |  |  |  |  |  |  |  |  |  |  |  |  |  |  |  | Medium | VIAQTDGTR | 2 |  | 0,0000 | 1,74 | 2 | 960,51103 | 0,11 | 36,17 |
|  |  |  |  |  |  |  |  |  |  |  |  |  |  |  |  |  | Medium | NQVYPK | 1 |  | 0,0000 | 1,52 | 2 | 748,39855 | -0,38 | 35,74 |
|  |  |  |  |  |  |  |  |  |  |  |  |  |  |  |  |  | Medium | LYAGLK | 10 |  | 0,0000 | 1,10 | 2 | 664,40318 | 0,49 | 40,87 |
| **1314** | M5XRM0 | 3-oxo-Delta(4,5)-steroid 5-beta-reductase | Uncharacterized protein OS=Prunus persica GN=PRUPE_ppa006780mg PE=4 SV=1 - [M5XRM0_PRUPE] | 89% to GI:694311973 Pyrus x bretschneideri, E value 0 | Pyrus x bretschneideri | Chloroplast | 01.06-Metabolism/Lipid and sterol | 28,07 | 15,95 | 2 | 5 | 6 | 21 | 395 | 44,6 | 5,27 | High | ANEIENcEAnGAMFR | 2 | C7(Carbamidomethyl); N10(Deamidated) | 0,0000 | 4,86 | 2 | 1726,71147 | -2,65 | 44,94 |
|  |  |  |  |  |  |  |  |  |  |  |  |  |  |  |  |  | High | ANEIENcEANGAMFR | 1 | C7(Carbamidomethyl) | 0,0000 | 3,29 | 2 | 1725,73430 | 1,32 | 43,46 |
|  |  |  |  |  |  |  |  |  |  |  |  |  |  |  |  |  | High | ANEIENcEANGAmFR | 3 | C7(Carbamidomethyl); M13(Oxidation) | 0,0000 | 3,12 | 2 | 1741,72905 | 1,21 | 41,40 |
|  |  |  |  |  |  |  |  |  |  |  |  |  |  |  |  |  | High | AHDPPFTEDLPR | 1 |  | 0,0000 | 2,68 | 3 | 1394,66938 | -0,43 | 43,09 |
|  |  |  |  |  |  |  |  |  |  |  |  |  |  |  |  |  | High | LSLVEMmK | 6 | M7(Oxidation) | 0,0000 | 2,39 | 2 | 966,49950 | -0,43 | 45,27 |
|  |  |  |  |  |  |  |  |  |  |  |  |  |  |  |  |  | High | LSLVEMMK | 1 |  | 0,0000 | 2,24 | 2 | 950,50456 | -0,46 | 48,85 |
|  |  |  |  |  |  |  |  |  |  |  |  |  |  |  |  |  | Medium | VVIPVAPNLR | 3 |  | 0,0000 | 1,96 | 2 | 1077,67778 | -0,16 | 47,16 |
|  |  |  |  |  |  |  |  |  |  |  |  |  |  |  |  |  | Medium | EGVWEEIVK | 1 |  | 0,0000 | 1,56 | 2 | 1088,56243 | 0,10 | 48,48 |
|  |  |  |  |  |  |  |  |  |  |  |  |  |  |  |  |  | Medium | LSLVEmmK | 1 | M6(Oxidation); M7(Oxidation) | 0,0000 | 1,53 | 2 | 982,49443 | -0,41 | 41,34 |
| **1319** | M5VPZ0 | Eukaryotic initiation factor | Uncharacterized protein OS=Prunus persica GN=PRUPE_ppa006450mg PE=4 SV=1 - [M5VPZ0_PRUPE] | 99% to GI:658005180 Malus domestica, E value 0 | Malus domestica | Chloroplast | 05.04-Protein synthesis/Translation factors | 34,44 | 22,38 | 5 | 4 | 12 | 22 | 411 | 46,7 | 5,58 | High | GLDVIQQAQSGTGK | 2 |  | 0,0000 | 4,08 | 2 | 1401,73406 | 0,54 | 42,71 |
|  |  |  |  |  |  |  |  |  |  |  |  |  |  |  |  |  | High | ALGDYLGVR | 2 |  | 0,0000 | 2,50 | 2 | 963,52544 | -0,43 | 46,43 |
|  |  |  |  |  |  |  |  |  |  |  |  |  |  |  |  |  | High | RDELTLEGIK | 1 |  | 0,0000 | 2,35 | 2 | 1173,64824 | 0,68 | 42,93 |
|  |  |  |  |  |  |  |  |  |  |  |  |  |  |  |  |  | High | VHAcVGGTSVR | 3 | C4(Carbamidomethyl) | 0,0000 | 2,06 | 2 | 1142,57378 | 0,19 | 35,60 |
|  |  |  |  |  |  |  |  |  |  |  |  |  |  |  |  |  | High | VFDMLR | 2 |  | 0,0000 | 2,06 | 2 | 780,40709 | -0,30 | 47,24 |
|  |  |  |  |  |  |  |  |  |  |  |  |  |  |  |  |  | High | DELTLEGIK | 1 |  | 0,0000 | 2,03 | 2 | 1017,54637 | 0,05 | 46,74 |
|  |  |  |  |  |  |  |  |  |  |  |  |  |  |  |  |  | Medium | ELAQQIEK | 2 |  | 0,0000 | 1,93 | 2 | 958,52031 | -0,13 | 38,44 |
|  |  |  |  |  |  |  |  |  |  |  |  |  |  |  |  |  | Medium | GVAINFVTK | 2 |  | 0,0000 | 1,70 | 2 | 948,55113 | -0,22 | 44,97 |
|  |  |  |  |  |  |  |  |  |  |  |  |  |  |  |  |  | Medium | KGVAINFVTK | 1 |  | 0,0000 | 1,67 | 2 | 1076,64629 | -0,02 | 42,02 |
|  |  |  |  |  |  |  |  |  |  |  |  |  |  |  |  |  | Medium | MLFDIQK | 1 |  | 0,0000 | 1,52 | 2 | 894,47472 | -0,76 | 47,57 |
|  |  |  |  |  |  |  |  |  |  |  |  |  |  |  |  |  | Medium | GIVPFcK | 1 | C6(Carbamidomethyl) | 0,0000 | 1,26 | 2 | 820,43877 | 0,19 | 44,67 |
|  |  |  |  |  |  |  |  |  |  |  |  |  |  |  |  |  | Medium | VFDmLR | 2 | M4(Oxidation) | 0,0000 | 1,25 | 2 | 796,40178 | -0,57 | 46,57 |
|  |  |  |  |  |  |  |  |  |  |  |  |  |  |  |  |  | Medium | VLITTDLLAR | 2 |  | 0,0000 | 1,16 | 2 | 1114,68401 | 0,82 | 50,19 |
| **1321** | M5WE97 | UDP-glycosyltransferase | Uncharacterized protein OS=Prunus persica GN=PRUPE_ppa005294mg PE=3 SV=1 - [M5WE97_PRUPE] | 95% to GI:645276064 Prunus mume, E value 0 | Prunus mume | Cytoplasm | 11.05-Disease/Defense/Stress responses | 119,88 | 32,05 | 2 | 14 | 14 | 57 | 468 | 52,1 | 5,43 | High | IEDDKEYGVQLYK | 3 |  | 0,0000 | 4,51 | 3 | 1599,78971 | -0,27 | 42,93 |
|  |  |  |  |  |  |  |  |  |  |  |  |  |  |  |  |  | High | LEQQVVDSmSK | 5 | M9(Oxidation) | 0,0000 | 3,81 | 2 | 1279,62053 | 0,49 | 38,17 |
|  |  |  |  |  |  |  |  |  |  |  |  |  |  |  |  |  | High | VMEGETAIEMK | 4 |  | 0,0000 | 3,63 | 2 | 1237,58135 | 0,79 | 41,95 |
|  |  |  |  |  |  |  |  |  |  |  |  |  |  |  |  |  | High | LEQQVVDSMSK | 3 |  | 0,0000 | 3,42 | 2 | 1263,62517 | 0,14 | 40,53 |
|  |  |  |  |  |  |  |  |  |  |  |  |  |  |  |  |  | High | VTVANTIFISK | 2 |  | 0,0000 | 3,39 | 2 | 1192,69328 | -0,31 | 46,21 |
|  |  |  |  |  |  |  |  |  |  |  |  |  |  |  |  |  | High | VMEGETAIEmK | 6 | M10(Oxidation) | 0,0000 | 3,22 | 2 | 1253,57561 | 0,26 | 39,41 |
|  |  |  |  |  |  |  |  |  |  |  |  |  |  |  |  |  | High | KVMEGETAIEMK | 3 |  | 0,0000 | 3,00 | 2 | 1365,67571 | 0,27 | 40,51 |
|  |  |  |  |  |  |  |  |  |  |  |  |  |  |  |  |  | High | AESIAAYLDR | 4 |  | 0,0000 | 2,91 | 2 | 1108,56316 | -0,18 | 47,10 |
|  |  |  |  |  |  |  |  |  |  |  |  |  |  |  |  |  | High | EMVLNQYSNLHK | 3 |  | 0,0000 | 2,77 | 2 | 1475,73149 | 0,22 | 41,79 |
|  |  |  |  |  |  |  |  |  |  |  |  |  |  |  |  |  | High | VmEGETAIEmK | 1 | M2(Oxidation); M10(Oxidation) | 0,0000 | 2,66 | 2 | 1269,56963 | -0,45 | 37,53 |
|  |  |  |  |  |  |  |  |  |  |  |  |  |  |  |  |  | High | ADFIFANSFYK | 2 |  | 0,0000 | 2,43 | 2 | 1322,64153 | -0,06 | 51,20 |
|  |  |  |  |  |  |  |  |  |  |  |  |  |  |  |  |  | High | LVQDVWK | 1 |  | 0,0000 | 2,42 | 2 | 887,49870 | 0,15 | 43,43 |
|  |  |  |  |  |  |  |  |  |  |  |  |  |  |  |  |  | High | SSGSVQFDTISDGYDEEGFAR | 1 |  | 0,0000 | 2,39 | 2 | 2266,97734 | 1,66 | 47,85 |
|  |  |  |  |  |  |  |  |  |  |  |  |  |  |  |  |  | High | KVMEGETAIEmK | 6 | M11(Oxidation) | 0,0000 | 2,24 | 2 | 1381,67009 | -0,12 | 37,16 |
|  |  |  |  |  |  |  |  |  |  |  |  |  |  |  |  |  | High | QMEELALGLK | 3 |  | 0,0687 | 2,17 | 2 | 1131,60808 | 0,17 | 49,78 |
|  |  |  |  |  |  |  |  |  |  |  |  |  |  |  |  |  | High | TLIELITK | 3 |  | 0,0000 | 2,06 | 2 | 930,58660 | -0,49 | 49,47 |
|  |  |  |  |  |  |  |  |  |  |  |  |  |  |  |  |  | Medium | EEIEScIR | 2 | C6(Carbamidomethyl) | 0,0000 | 1,95 | 2 | 1035,47734 | -0,23 | 39,50 |
|  |  |  |  |  |  |  |  |  |  |  |  |  |  |  |  |  | Medium | QmEELALGLK | 3 | M2(Oxidation) | 0,0000 | 1,76 | 2 | 1147,60271 | -0,09 | 44,96 |
|  |  |  |  |  |  |  |  |  |  |  |  |  |  |  |  |  | Medium | MLTIGPTIPYTYLDNR | 1 |  | 0,0000 | 1,73 | 2 | 1867,96416 | 0,99 | 51,28 |
|  |  |  |  |  |  |  |  |  |  |  |  |  |  |  |  |  | Medium | EmVLNQYSNLHK | 1 | M2(Oxidation) | 0,0000 | 1,38 | 3 | 1491,72599 | -0,07 | 40,68 |
|  | M5WTK5 | actin | Uncharacterized protein OS=Prunus persica GN=PRUPE_ppa007242mg PE=3 SV=1 - [M5WTK5_PRUPE] | 100% to GI:225431585 Vitis vinifera, E value 0 | Vitis vinifera | Cytoplasm | 09.04-Cell structure/Cytoskeleton | 76,03 | 32,10 | 4 | 3 | 12 | 44 | 377 | 41,7 | 5,49 | High | AGFAGDDAPR | 3 |  | 0,0000 | 3,12 | 2 | 976,44798 | -0,38 | 38,18 |
|  |  |  |  |  |  |  |  |  |  |  |  |  |  |  |  |  | High | GYmFTTTAER | 3 | M3(Oxidation) | 0,0000 | 2,96 | 2 | 1192,53069 | 0,29 | 41,76 |
|  |  |  |  |  |  |  |  |  |  |  |  |  |  |  |  |  | High | GEYDESGPSIVHR | 5 |  | 0,0000 | 2,82 | 2 | 1445,66643 | 0,57 | 40,20 |
|  |  |  |  |  |  |  |  |  |  |  |  |  |  |  |  |  | High | GYMFTTTAER | 3 |  | 0,0000 | 2,78 | 2 | 1176,53569 | 0,22 | 44,11 |
|  |  |  |  |  |  |  |  |  |  |  |  |  |  |  |  |  | High | DAYVGDEAQSK | 2 |  | 0,0000 | 2,73 | 2 | 1182,52751 | 0,12 | 37,48 |
|  |  |  |  |  |  |  |  |  |  |  |  |  |  |  |  |  | High | NYELPDGQVITIGAER | 2 |  | 0,0000 | 2,62 | 2 | 1774,89946 | 1,34 | 49,60 |
|  |  |  |  |  |  |  |  |  |  |  |  |  |  |  |  |  | High | HTGVmVGMGQK | 4 | M5(Oxidation) | 0,0000 | 2,59 | 2 | 1160,55510 | -0,04 | 36,47 |
|  |  |  |  |  |  |  |  |  |  |  |  |  |  |  |  |  | High | VAPEEHPVLLTEAPLNPK | 2 |  | 0,0000 | 2,57 | 3 | 1954,06528 | 0,40 | 45,58 |
|  |  |  |  |  |  |  |  |  |  |  |  |  |  |  |  |  | High | HTGVMVGMGQK | 4 |  | 0,0000 | 2,30 | 2 | 1144,56023 | 0,00 | 38,38 |
|  |  |  |  |  |  |  |  |  |  |  |  |  |  |  |  |  | High | EITALAPSSmK | 4 | M10(Oxidation) | 0,0000 | 2,12 | 2 | 1163,59722 | -0,43 | 40,42 |
|  |  |  |  |  |  |  |  |  |  |  |  |  |  |  |  |  | High | EITALAPSSMK | 2 |  | 0,0000 | 2,08 | 2 | 1147,60295 | 0,13 | 43,91 |
|  |  |  |  |  |  |  |  |  |  |  |  |  |  |  |  |  | Medium | DLTDALmK | 1 | M7(Oxidation) | 0,0000 | 1,86 | 2 | 922,45470 | -0,40 | 44,31 |
|  |  |  |  |  |  |  |  |  |  |  |  |  |  |  |  |  | Medium | DLTDALMK | 2 |  | 0,0000 | 1,74 | 2 | 906,45970 | -0,49 | 47,55 |
|  |  |  |  |  |  |  |  |  |  |  |  |  |  |  |  |  | Medium | RGILTLK | 1 |  | 0,0000 | 1,66 | 2 | 800,53496 | -0,41 | 39,93 |
|  |  |  |  |  |  |  |  |  |  |  |  |  |  |  |  |  | Medium | LDLAGR | 2 |  | 0,0000 | 1,13 | 2 | 644,37285 | 0,32 | 40,60 |
|  |  |  |  |  |  |  |  |  |  |  |  |  |  |  |  |  | Medium | GILTLK | 4 |  | 0,0000 | 1,09 | 2 | 644,43450 | 0,50 | 43,50 |
| **1628** | M5W3N4 | mediator of RNA polymerase II transcription subunit | Uncharacterized protein OS=Prunus persica GN=PRUPE_ppa024477mg PE=3 SV=1 - [M5W3N4_PRUPE] | 98% to GI:645220654 Prunus mume, E value 0 | Prunus mume | Endoplasmic reticulum | 04.05.01.04-Transcription/transcriptional control | 74,06 | 33,23 | 7 | 6 | 15 | 31 | 635 | 69,5 | 5,36 | High | MYQGAGADGAGPVDDEAPAGASGPGPK | 1 |  | 0,0000 | 3,53 | 3 | 2443,08506 | 0,76 | 41,59 |
|  |  |  |  |  |  |  |  |  |  |  |  |  |  |  |  |  | High | FSDASVQSDMK | 2 |  | 0,0000 | 3,28 | 2 | 1214,53655 | 0,58 | 40,15 |
|  |  |  |  |  |  |  |  |  |  |  |  |  |  |  |  |  | High | VEIIANDQGNR | 3 |  | 0,0000 | 3,18 | 2 | 1228,62834 | 0,20 | 39,67 |
|  |  |  |  |  |  |  |  |  |  |  |  |  |  |  |  |  | High | TTPSYVAFTDTER | 3 |  | 0,0000 | 3,03 | 2 | 1487,70171 | 0,27 | 44,59 |
|  |  |  |  |  |  |  |  |  |  |  |  |  |  |  |  |  | High | NALENYAYNMR | 2 |  | 0,0000 | 3,02 | 2 | 1358,61711 | 0,97 | 45,92 |
|  |  |  |  |  |  |  |  |  |  |  |  |  |  |  |  |  | High | EIAEAYLGSTIK | 2 |  | 0,0000 | 2,69 | 2 | 1294,68901 | 0,04 | 46,87 |
|  |  |  |  |  |  |  |  |  |  |  |  |  |  |  |  |  | High | ELESIcNPIIAK | 3 | C6(Carbamidomethyl) | 0,0000 | 2,67 | 2 | 1386,73052 | 0,52 | 47,05 |
|  |  |  |  |  |  |  |  |  |  |  |  |  |  |  |  |  | High | NALENYAYNmR | 2 | M10(Oxidation) | 0,0000 | 2,64 | 2 | 1374,61125 | 0,39 | 43,16 |
|  |  |  |  |  |  |  |  |  |  |  |  |  |  |  |  |  | High | NQVAMNPINTVFDAK | 2 |  | 0,0000 | 2,60 | 2 | 1661,83574 | 2,46 | 49,01 |
|  |  |  |  |  |  |  |  |  |  |  |  |  |  |  |  |  | High | DAGVIAGINVLR | 1 |  | 0,0000 | 2,44 | 2 | 1197,69499 | -0,06 | 49,54 |
|  |  |  |  |  |  |  |  |  |  |  |  |  |  |  |  |  | Medium | STVHDVVLVGGSTR | 1 |  | 0,0000 | 2,40 | 3 | 1426,76401 | -0,64 | 40,61 |
|  |  |  |  |  |  |  |  |  |  |  |  |  |  |  |  |  | High | ATAGDTHLGGEDFDNR | 4 |  | 0,0000 | 2,29 | 2 | 1675,73137 | 0,37 | 39,53 |
|  |  |  |  |  |  |  |  |  |  |  |  |  |  |  |  |  | High | IINEPTAAAIAYGLDK | 1 |  | 0,0000 | 2,22 | 2 | 1659,89739 | 1,26 | 48,39 |
|  |  |  |  |  |  |  |  |  |  |  |  |  |  |  |  |  | High | NAVVTVPAYFNDSQR | 1 |  | 0,0000 | 2,08 | 2 | 1680,83611 | 1,21 | 47,20 |
|  |  |  |  |  |  |  |  |  |  |  |  |  |  |  |  |  | Medium | FEELNMDLFR | 1 |  | 0,0000 | 1,86 | 2 | 1313,61931 | -0,16 | 51,23 |
|  |  |  |  |  |  |  |  |  |  |  |  |  |  |  |  |  | Medium | NQVAMnPInTVFDAK | 1 | N6(Deamidated); N9(Deamidated) | 0,2634 | 1,79 | 2 | 1663,81243 | 7,66 | 45,85 |
| **1705** | M5WY92 | HSP70 | Uncharacterized protein OS=Prunus persica GN=PRUPE_ppa002598mg PE=3 SV=1 - [M5WY92_PRUPE] | 99% to GI:645245339 Prunus mume, E value 0 | Prunus mume | Endoplasmic reticulum | 06.01-Protein destination and storage/Folding and stability | 105,98 | 23,09 | 7 | 6 | 12 | 39 | 654 | 71,7 | 5,38 | High | NQVAMNPQNTVFDAK | 3 |  | 0,0000 | 4,04 | 2 | 1676,80852 | 1,41 | 45,78 |
|  |  |  |  |  |  |  |  |  |  |  |  |  |  |  |  |  | High | VDEVVLVGGSTR | 3 |  | 0,0000 | 3,55 | 2 | 1230,66899 | 0,07 | 44,49 |
|  |  |  |  |  |  |  |  |  |  |  |  |  |  |  |  |  | High | ATAGDTHLGGEDFDNR | 2 |  | 0,0000 | 3,55 | 2 | 1675,73186 | 0,66 | 39,56 |
|  |  |  |  |  |  |  |  |  |  |  |  |  |  |  |  |  | High | NQVAmNPQNTVFDAK | 3 | M5(Oxidation) | 0,0000 | 3,47 | 2 | 1692,80303 | 1,15 | 41,47 |
|  |  |  |  |  |  |  |  |  |  |  |  |  |  |  |  |  | High | VEIIANDQGNR | 3 |  | 0,0000 | 3,37 | 2 | 1228,62822 | 0,10 | 40,51 |
|  |  |  |  |  |  |  |  |  |  |  |  |  |  |  |  |  | High | DAGVIAGLNVmR | 2 | M11(Oxidation) | 0,0000 | 3,35 | 2 | 1231,64592 | -0,40 | 46,14 |
|  |  |  |  |  |  |  |  |  |  |  |  |  |  |  |  |  | High | NSLENYAYSMR | 2 |  | 0,0000 | 3,35 | 2 | 1347,60051 | 0,52 | 44,81 |
|  |  |  |  |  |  |  |  |  |  |  |  |  |  |  |  |  | High | FSDPSVQSDMK | 3 |  | 0,0000 | 3,21 | 2 | 1240,55193 | 0,35 | 41,52 |
|  |  |  |  |  |  |  |  |  |  |  |  |  |  |  |  |  | High | DAGVIAGLNVMR | 1 |  | 0,0000 | 3,17 | 2 | 1215,65166 | 0,13 | 48,92 |
|  |  |  |  |  |  |  |  |  |  |  |  |  |  |  |  |  | High | IINEPTAAAIAYGLDK | 1 |  | 0,0000 | 3,08 | 2 | 1659,89629 | 0,60 | 48,21 |
|  |  |  |  |  |  |  |  |  |  |  |  |  |  |  |  |  | High | NQLAEVEEFEDK | 2 |  | 0,0000 | 2,96 | 2 | 1450,67009 | 0,27 | 45,37 |
|  |  |  |  |  |  |  |  |  |  |  |  |  |  |  |  |  | High | TTPSYVAFTDTER | 4 |  | 0,0000 | 2,79 | 2 | 1487,70232 | 0,68 | 45,41 |
|  |  |  |  |  |  |  |  |  |  |  |  |  |  |  |  |  | High | ELEGLcNPIIAK | 2 | C6(Carbamidomethyl) | 0,0000 | 2,75 | 2 | 1356,71990 | 0,49 | 47,02 |
|  |  |  |  |  |  |  |  |  |  |  |  |  |  |  |  |  | High | NSLENYAYSmR | 1 | M10(Oxidation) | 0,0000 | 2,53 | 2 | 1363,59538 | 0,48 | 41,75 |
|  |  |  |  |  |  |  |  |  |  |  |  |  |  |  |  |  | High | FEEMNmDLFR | 4 | M6(Oxidation) | 0,0000 | 2,45 | 2 | 1347,57207 | 0,89 | 47,52 |
|  |  |  |  |  |  |  |  |  |  |  |  |  |  |  |  |  | High | FSDPSVQSDmK | 2 | M10(Oxidation) | 0,0000 | 2,37 | 2 | 1256,54741 | 0,80 | 38,25 |
|  |  |  |  |  |  |  |  |  |  |  |  |  |  |  |  |  | High | FEEmNmDLFR | 1 | M4(Oxidation); M6(Oxidation) | 0,0000 | 2,13 | 2 | 1363,56682 | 0,76 | 44,65 |
|  | M5VX34 | Peptidyl-prolyl cis-trans isomerase | Uncharacterized protein OS=Prunus persica GN=PRUPE_ppa003471mg PE=4 SV=1 - [M5VX34_PRUPE] | 99% to GI:645279361 Prunus mume, E value 0 | Prunus mume | Endoplasmic reticulum | 06.01-Protein destination and storage/Folding and stability | 47,52 | 24,30 | 1 | 16 | 17 | 34 | 572 | 63,9 | 5,34 | High | TDEEEVIDGLDR | 3 |  | 0,0000 | 3,31 | 2 | 1390,63396 | 0,46 | 46,65 |
|  |  |  |  |  |  |  |  |  |  |  |  |  |  |  |  |  | High | VAcNLNNAAcK | 2 | C3(Carbamidomethyl); C10(Carbamidomethyl) | 0,0000 | 3,30 | 2 | 1234,56706 | 0,25 | 36,49 |
|  |  |  |  |  |  |  |  |  |  |  |  |  |  |  |  |  | High | SDRVEFTVK | 1 |  | 0,0000 | 2,51 | 3 | 1080,56806 | -0,36 | 39,53 |
|  |  |  |  |  |  |  |  |  |  |  |  |  |  |  |  |  | High | DKESWDMNTEEK | 2 |  | 0,0000 | 2,41 | 2 | 1511,63298 | 0,69 | 39,78 |
|  |  |  |  |  |  |  |  |  |  |  |  |  |  |  |  |  | High | DGYFcPALSK | 3 | C5(Carbamidomethyl) | 0,0000 | 2,22 | 2 | 1157,52971 | 0,08 | 46,64 |
|  |  |  |  |  |  |  |  |  |  |  |  |  |  |  |  |  | High | ALEIDPNNR | 1 |  | 0,0000 | 2,21 | 2 | 1041,53203 | -0,36 | 40,92 |
|  |  |  |  |  |  |  |  |  |  |  |  |  |  |  |  |  | High | DAEPMSVDSK | 1 |  | 0,0000 | 2,19 | 2 | 1078,47197 | -0,19 | 38,80 |
|  |  |  |  |  |  |  |  |  |  |  |  |  |  |  |  |  | High | VLEIEGR | 3 |  | 0,0000 | 2,14 | 2 | 815,46263 | 0,54 | 41,71 |
|  |  |  |  |  |  |  |  |  |  |  |  |  |  |  |  |  | Medium | LQDGTEFLK | 2 |  | 0,0000 | 2,00 | 2 | 1050,54619 | -0,44 | 44,71 |
|  |  |  |  |  |  |  |  |  |  |  |  |  |  |  |  |  | Medium | EGEGWDTPK | 2 |  | 0,0000 | 1,99 | 2 | 1018,44762 | -0,05 | 39,84 |
|  |  |  |  |  |  |  |  |  |  |  |  |  |  |  |  |  | Medium | FTLGQGQVIK | 3 |  | 0,0000 | 1,98 | 2 | 1090,62541 | -0,14 | 44,90 |
|  |  |  |  |  |  |  |  |  |  |  |  |  |  |  |  |  | Medium | DAEPMSVDSKA | 1 |  | 0,0000 | 1,78 | 2 | 1149,50957 | 0,23 | 39,85 |
|  |  |  |  |  |  |  |  |  |  |  |  |  |  |  |  |  | Medium | DKESWDmNTEEK | 2 | M7(Oxidation) | 0,0000 | 1,45 | 2 | 1527,62663 | -0,15 | 37,29 |
|  |  |  |  |  |  |  |  |  |  |  |  |  |  |  |  |  | Medium | GWDEGIR | 1 |  | 0,0000 | 1,38 | 2 | 832,39598 | 1,37 | 42,10 |
|  |  |  |  |  |  |  |  |  |  |  |  |  |  |  |  |  | Medium | EGEGFERPNEGAVVK | 2 |  | 0,0000 | 1,37 | 3 | 1617,78586 | -0,59 | 41,02 |
|  |  |  |  |  |  |  |  |  |  |  |  |  |  |  |  |  | Medium | VEFTVK | 1 |  | 0,0160 | 1,23 | 2 | 722,40825 | -0,14 | 41,82 |
|  |  |  |  |  |  |  |  |  |  |  |  |  |  |  |  |  | Medium | FYGNMFAK | 2 |  | 0,0000 | 1,09 | 2 | 977,45470 | -0,30 | 46,23 |
|  |  |  |  |  |  |  |  |  |  |  |  |  |  |  |  |  | Medium | DAEPmSVDSK | 1 | M5(Oxidation) | 0,0000 | 1,07 | 2 | 1094,46697 | -0,12 | 36,31 |
| **1820** | M5XT23 | Protein transport protein | Uncharacterized protein OS=Prunus persica GN=PRUPE_ppa000665mg PE=4 SV=1 - [M5XT23_PRUPE] | 96% to GI:645251127 Prunus mume, E value 0 | Prunus mume | Chloroplast | 07.99-Transporters/Others | 19,31 | 7,08 | 1 | 7 | 7 | 10 | 1045 | 113,0 | 5,38 | High | DLPVVGESTSSEK | 1 |  | 0,0000 | 3,57 | 2 | 1347,66460 | 0,53 | 42,85 |
|  |  |  |  |  |  |  |  |  |  |  |  |  |  |  |  |  | High | SSEFEAAIQNGEK | 1 |  | 0,0000 | 3,04 | 2 | 1409,65715 | 1,97 | 42,04 |
|  |  |  |  |  |  |  |  |  |  |  |  |  |  |  |  |  | High | TIVLALASGQK | 1 |  | 0,0000 | 2,65 | 2 | 1100,66716 | -0,25 | 45,56 |
|  |  |  |  |  |  |  |  |  |  |  |  |  |  |  |  |  | High | IALSTEPENVSK | 2 |  | 0,0000 | 2,60 | 2 | 1287,68010 | 0,75 | 41,43 |
|  |  |  |  |  |  |  |  |  |  |  |  |  |  |  |  |  | High | VMLEDDGTAR | 2 |  | 0,0000 | 2,16 | 2 | 1106,51409 | -0,57 | 39,63 |
|  |  |  |  |  |  |  |  |  |  |  |  |  |  |  |  |  | Medium | FSASLcK | 1 | C6(Carbamidomethyl) | 0,0000 | 1,64 | 2 | 812,39665 | -0,59 | 38,97 |
|  |  |  |  |  |  |  |  |  |  |  |  |  |  |  |  |  | Medium | MSHSPYLK | 1 |  | 0,0071 | 1,39 | 3 | 962,47693 | 0,51 | 36,72 |
|  |  |  |  |  |  |  |  |  |  |  |  |  |  |  |  |  | Medium | VmLEDDGTAR | 1 | M2(Oxidation) | 0,0000 | 1,08 | 2 | 1122,51067 | 0,92 | 37,88 |
| **1821** | M5WX60 | HSP70 | Uncharacterized protein OS=Prunus persica GN=PRUPE_ppa001317mg PE=3 SV=1 - [M5WX60_PRUPE] | 98% to GI:645244630 Prunus mume, E value 0 | Prunus mume | Mitochondrion | 06.01-Protein destination and storage/Folding and stability | 81,42 | 26,32 | 2 | 20 | 22 | 53 | 855 | 94,6 | 5,27 | High | FIGTAGAASSLmNPK | 1 | M12(Oxidation) | 0,0000 | 4,06 | 2 | 1480,74785 | 0,89 | 42,43 |
|  |  |  |  |  |  |  |  |  |  |  |  |  |  |  |  |  | High | FIGTAGAASSLMNPK | 2 |  | 0,0000 | 3,57 | 2 | 1464,75261 | 0,68 | 46,50 |
|  |  |  |  |  |  |  |  |  |  |  |  |  |  |  |  |  | High | YANPVLLSADVR | 2 |  | 0,0000 | 3,55 | 2 | 1317,71660 | 0,32 | 47,53 |
|  |  |  |  |  |  |  |  |  |  |  |  |  |  |  |  |  | High | FEHIDISDK | 4 |  | 0,0000 | 3,14 | 3 | 1103,53653 | -0,25 | 41,69 |
|  |  |  |  |  |  |  |  |  |  |  |  |  |  |  |  |  | High | YQEFVTEPER | 2 |  | 0,0000 | 3,03 | 2 | 1297,60661 | 0,51 | 42,74 |
|  |  |  |  |  |  |  |  |  |  |  |  |  |  |  |  |  | High | GIDVVLNDESK | 1 |  | 0,0000 | 3,01 | 2 | 1188,61064 | -0,07 | 44,46 |
|  |  |  |  |  |  |  |  |  |  |  |  |  |  |  |  |  | High | EFEMALQDR | 3 |  | 0,0000 | 2,72 | 2 | 1138,51982 | 0,02 | 45,92 |
|  |  |  |  |  |  |  |  |  |  |  |  |  |  |  |  |  | High | ILAHSFDQSLGGR | 1 |  | 0,0000 | 2,59 | 3 | 1400,72751 | -0,46 | 42,26 |
|  |  |  |  |  |  |  |  |  |  |  |  |  |  |  |  |  | High | TMNASEcVAR | 3 | C7(Carbamidomethyl) | 0,0000 | 2,49 | 2 | 1138,49785 | -0,14 | 36,75 |
|  |  |  |  |  |  |  |  |  |  |  |  |  |  |  |  |  | High | NAVEAYVYDmR | 1 | M10(Oxidation) | 0,0000 | 2,47 | 2 | 1346,60503 | 0,34 | 43,57 |
|  |  |  |  |  |  |  |  |  |  |  |  |  |  |  |  |  | High | ETPALVcFGDK | 2 | C7(Carbamidomethyl) | 0,0000 | 2,35 | 2 | 1236,59233 | -0,51 | 45,70 |
|  |  |  |  |  |  |  |  |  |  |  |  |  |  |  |  |  | High | IDVFQNAR | 3 |  | 0,0000 | 2,32 | 2 | 962,50554 | 0,09 | 42,96 |
|  |  |  |  |  |  |  |  |  |  |  |  |  |  |  |  |  | High | GSGPDTQnGAVDNNQSTIVFPK | 7 | N8(Deamidated) | 0,0000 | 2,25 | 2 | 2247,05449 | 0,89 | 46,85 |
|  |  |  |  |  |  |  |  |  |  |  |  |  |  |  |  |  | High | ISTYTIGPFQSTK | 2 |  | 0,0000 | 2,08 | 2 | 1442,75383 | 0,85 | 47,42 |
|  |  |  |  |  |  |  |  |  |  |  |  |  |  |  |  |  | Medium | RTMNASEcVAR | 2 | C8(Carbamidomethyl) | 0,0000 | 1,92 | 3 | 1294,59858 | -0,43 | 35,38 |
|  |  |  |  |  |  |  |  |  |  |  |  |  |  |  |  |  | Medium | QFSDPVVQR | 2 |  | 0,0000 | 1,88 | 2 | 1075,55278 | -0,32 | 40,91 |
|  |  |  |  |  |  |  |  |  |  |  |  |  |  |  |  |  | Medium | VPAIIK | 3 |  | 0,0000 | 1,69 | 2 | 640,43932 | 0,09 | 41,26 |
|  |  |  |  |  |  |  |  |  |  |  |  |  |  |  |  |  | Medium | EFEmALQDR | 1 | M4(Oxidation) | 0,0000 | 1,65 | 2 | 1154,51470 | -0,02 | 41,72 |
|  |  |  |  |  |  |  |  |  |  |  |  |  |  |  |  |  | Medium | METDEAPSDAAPPSTNETDVNMQDAK | 2 |  | 0,0000 | 1,57 | 3 | 2764,15940 | 1,17 | 43,07 |
|  |  |  |  |  |  |  |  |  |  |  |  |  |  |  |  |  | Medium | nAVEAYVYDMRnK | 1 | N1(Deamidated); N12(Deamidated) | 0,0000 | 1,29 | 2 | 1574,73027 | 9,32 | 41,40 |
|  |  |  |  |  |  |  |  |  |  |  |  |  |  |  |  |  | Medium | GNPIPSIK | 2 |  | 0,0000 | 1,04 | 2 | 825,48296 | 0,04 | 41,03 |
|  |  |  |  |  |  |  |  |  |  |  |  |  |  |  |  |  | Medium | GSGPDTQNGAVDNNQSTIVFPK | 1 |  | 0,0000 | 1,02 | 2 | 2246,07158 | 1,38 | 44,64 |
|  |  |  |  |  |  |  |  |  |  |  |  |  |  |  |  |  | Medium | EAFIAR | 1 |  | 0,0105 | 0,94 | 2 | 706,38835 | 0,09 | 39,55 |
| **2007** | M5XE29 | 3-hydroxyisobutyrate dehydrogenase | Uncharacterized protein OS=Prunus persica GN=PRUPE_ppa009081mg PE=4 SV=1 - [M5XE29_PRUPE] | 98% to GI:645223765 Prunus mume, E value 0 | Prunus mume | Mitochondrion | 01.01-Metabolism/Amino Acid | 22,74 | 29,32 | 2 | 7 | 7 | 13 | 307 | 32,0 | 5,66 | High | DcWTVDAPVSGGDIGAR | 1 | C2(Carbamidomethyl) | 0,0000 | 3,49 | 2 | 1775,80522 | 1,91 | 47,86 |
|  |  |  |  |  |  |  |  |  |  |  |  |  |  |  |  |  | High | VTYVGPAGSGQNcK | 2 | C13(Carbamidomethyl) | 0,0000 | 3,05 | 2 | 1437,67998 | 0,58 | 39,08 |
|  |  |  |  |  |  |  |  |  |  |  |  |  |  |  |  |  | High | QLFSGMVANGDGK | 2 |  | 0,0000 | 2,62 | 2 | 1323,63689 | 0,49 | 46,23 |
|  |  |  |  |  |  |  |  |  |  |  |  |  |  |  |  |  | High | VAVLPGAALcK | 2 | C10(Carbamidomethyl) | 0,0000 | 2,60 | 2 | 1098,63396 | -0,08 | 45,34 |
|  |  |  |  |  |  |  |  |  |  |  |  |  |  |  |  |  | High | QLFSGMVAnGDGK | 2 | N9(Deamidated) | 0,0000 | 2,04 | 2 | 1324,62187 | 1,22 | 46,39 |
|  |  |  |  |  |  |  |  |  |  |  |  |  |  |  |  |  | Medium | LGGQGLITVIER | 1 |  | 0,0000 | 1,94 | 2 | 1255,73845 | 1,21 | 48,16 |
|  |  |  |  |  |  |  |  |  |  |  |  |  |  |  |  |  | Medium | DLGMGVDVVEENEEGR | 1 |  | 0,0000 | 1,49 | 2 | 1747,78252 | 1,19 | 48,51 |
|  |  |  |  |  |  |  |  |  |  |  |  |  |  |  |  |  | Medium | EIFNAAR | 2 |  | 0,0000 | 1,45 | 2 | 820,43114 | -0,10 | 41,14 |
| **2104** | M5XCH9 | Nudix hydrolase | Uncharacterized protein OS=Prunus persica GN=PRUPE_ppa007741mg PE=3 SV=1 - [M5XCH9_PRUPE] | 96% to GI:645247643 Prunus mume, E value 0 | Prunus mume | Chloroplast | 11.05-Disease/Defense/Stress responses | 64,32 | 30,38 | 3 | 9 | 9 | 31 | 293 | 32,8 | 5,43 | High | SDKDYAGFSPMLTTTSSGK | 1 |  | 0,0000 | 3,94 | 3 | 1992,92301 | 0,52 | 44,79 |
|  |  |  |  |  |  |  |  |  |  |  |  |  |  |  |  |  | High | DMANIQATGDHQ | 3 |  | 0,0000 | 3,77 | 2 | 1300,55913 | 0,33 | 42,20 |
|  |  |  |  |  |  |  |  |  |  |  |  |  |  |  |  |  | High | DmANIQATGDHQ | 4 | M2(Oxidation) | 0,0000 | 3,52 | 2 | 1316,55351 | -0,08 | 38,19 |
|  |  |  |  |  |  |  |  |  |  |  |  |  |  |  |  |  | High | EILVVQEInGR | 2 | N9(Deamidated) | 0,0000 | 3,48 | 2 | 1270,70036 | 0,12 | 48,40 |
|  |  |  |  |  |  |  |  |  |  |  |  |  |  |  |  |  | High | EILVVQEINGR | 2 |  | 0,0000 | 3,12 | 2 | 1269,71623 | 0,03 | 46,78 |
|  |  |  |  |  |  |  |  |  |  |  |  |  |  |  |  |  | High | VGIGAFVMNSER | 2 |  | 0,0000 | 2,95 | 2 | 1279,64702 | 0,48 | 47,54 |
|  |  |  |  |  |  |  |  |  |  |  |  |  |  |  |  |  | High | VGIGAFVmNSER | 3 | M8(Oxidation) | 0,0000 | 2,36 | 2 | 1295,64250 | 0,91 | 46,10 |
|  |  |  |  |  |  |  |  |  |  |  |  |  |  |  |  |  | High | ASMSQWK | 3 |  | 0,0000 | 2,06 | 2 | 837,39220 | -0,23 | 40,85 |
|  |  |  |  |  |  |  |  |  |  |  |  |  |  |  |  |  | High | GSYLYHNNR | 4 |  | 0,0000 | 2,01 | 2 | 1123,52776 | -0,16 | 36,86 |
|  |  |  |  |  |  |  |  |  |  |  |  |  |  |  |  |  | Medium | MSVSTSSSSSVAK | 2 |  | 0,0000 | 2,00 | 2 | 1257,59905 | -0,09 | 37,18 |
|  |  |  |  |  |  |  |  |  |  |  |  |  |  |  |  |  | Medium | DYAGFSPMLTTTSSGK | 1 |  | 0,0000 | 1,79 | 2 | 1662,77056 | 1,52 | 48,72 |
|  |  |  |  |  |  |  |  |  |  |  |  |  |  |  |  |  | Medium | DTGFWK | 3 |  | 0,0000 | 1,28 | 2 | 753,35655 | -0,13 | 46,19 |
|  | M5VQF7 | 2-oxoglutarate-dependent dioxygenase DAO | Uncharacterized protein OS=Prunus persica GN=PRUPE_ppa009067mg PE=3 SV=1 - [M5VQF7_PRUPE] | 98% to GI:646227285 Prunus mume, E value 0 | Prunus mume | Chloroplast | 02.10-Energy/TCA pathway | 40,69 | 32,57 | 1 | 11 | 11 | 23 | 307 | 34,1 | 5,69 | High | DVIAGSGYMAPSK | 3 |  | 0,0000 | 3,70 | 2 | 1295,63066 | 0,45 | 43,00 |
|  |  |  |  |  |  |  |  |  |  |  |  |  |  |  |  |  | High | QQNKDVIAGSGYMAPSK | 2 |  | 0,0000 | 3,26 | 3 | 1793,88626 | 0,63 | 39,54 |
|  |  |  |  |  |  |  |  |  |  |  |  |  |  |  |  |  | High | FPDAEEYKK | 3 |  | 0,0000 | 2,92 | 3 | 1126,54145 | -0,10 | 39,20 |
|  |  |  |  |  |  |  |  |  |  |  |  |  |  |  |  |  | High | LQAGEALAVLR | 2 |  | 0,0000 | 2,90 | 2 | 1140,67302 | -0,51 | 46,92 |
|  |  |  |  |  |  |  |  |  |  |  |  |  |  |  |  |  | High | DVIAGSGYmAPSK | 2 | M9(Oxidation) | 0,0000 | 2,89 | 2 | 1311,62480 | -0,15 | 43,44 |
|  |  |  |  |  |  |  |  |  |  |  |  |  |  |  |  |  | High | DQEAVEAPPEFVDSEHPR | 2 |  | 0,0000 | 2,59 | 3 | 2051,93186 | 0,62 | 44,68 |
|  |  |  |  |  |  |  |  |  |  |  |  |  |  |  |  |  | High | FPDAEEYK | 1 |  | 0,0000 | 2,35 | 2 | 998,44640 | -0,19 | 41,55 |
|  |  |  |  |  |  |  |  |  |  |  |  |  |  |  |  |  | Medium | EASEIWGcFR | 2 | C8(Carbamidomethyl) | 0,0000 | 1,59 | 2 | 1254,55730 | 0,04 | 48,01 |
|  |  |  |  |  |  |  |  |  |  |  |  |  |  |  |  |  | Medium | IPLALMK | 2 |  | 0,0000 | 1,42 | 2 | 785,49529 | -0,16 | 46,57 |
|  |  |  |  |  |  |  |  |  |  |  |  |  |  |  |  |  | Medium | GWPcQFR | 1 | C4(Carbamidomethyl) | 0,0000 | 1,40 | 2 | 950,42974 | -0,46 | 44,46 |
|  |  |  |  |  |  |  |  |  |  |  |  |  |  |  |  |  | Medium | VSIATFLLGPK | 1 |  | 0,0000 | 1,31 | 2 | 1145,69243 | -0,44 | 52,10 |
|  |  |  |  |  |  |  |  |  |  |  |  |  |  |  |  |  | Medium | IPLALmK | 1 | M6(Oxidation) | 0,0000 | 1,10 | 2 | 801,49047 | 0,17 | 42,54 |
|  |  |  |  |  |  |  |  |  |  |  |  |  |  |  |  |  | Medium | SLLDLPmEIK | 1 | M7(Oxidation) | 0,0000 | 1,00 | 2 | 1174,63823 | -0,53 | 49,67 |
| **2311** | M5W621 | Aminoacylase | Uncharacterized protein OS=Prunus persica GN=PRUPE_ppa005858mg PE=4 SV=1 - [M5W621_PRUPE] | 99% to GI:645260433 Prunus mume, E value 0 | Prunus mume | Chloroplast | 01.01-Metabolism/Amino acid | 58,48 | 23,41 | 1 | 10 | 10 | 25 | 440 | 48,7 | 5,74 | High | FAESDVFK | 3 |  | 0,0000 | 2,98 | 2 | 942,45635 | -0,45 | 44,17 |
|  |  |  |  |  |  |  |  |  |  |  |  |  |  |  |  |  | High | VPPTADQESLER | 3 |  | 0,0000 | 2,91 | 2 | 1341,66472 | 0,13 | 39,87 |
|  |  |  |  |  |  |  |  |  |  |  |  |  |  |  |  |  | High | LGKPEIFPASTDAR | 3 |  | 0,0000 | 2,88 | 3 | 1501,80054 | -0,31 | 41,77 |
|  |  |  |  |  |  |  |  |  |  |  |  |  |  |  |  |  | High | IAEEWAPSSR | 2 |  | 0,0000 | 2,87 | 2 | 1145,55876 | 0,12 | 42,59 |
|  |  |  |  |  |  |  |  |  |  |  |  |  |  |  |  |  | High | ASQFDLVK | 2 |  | 0,0000 | 2,75 | 2 | 907,48802 | -0,42 | 44,18 |
|  |  |  |  |  |  |  |  |  |  |  |  |  |  |  |  |  | High | cVGIQYLEAIR | 1 | C1(Carbamidomethyl) | 0,0000 | 2,61 | 2 | 1321,69316 | -0,13 | 48,71 |
|  |  |  |  |  |  |  |  |  |  |  |  |  |  |  |  |  | High | AYASYVDHGR | 4 |  | 0,0000 | 2,45 | 2 | 1138,52739 | -0,20 | 37,92 |
|  |  |  |  |  |  |  |  |  |  |  |  |  |  |  |  |  | High | LYDNTATENLFK | 3 |  | 0,0000 | 2,42 | 2 | 1428,70012 | -0,32 | 48,54 |
|  |  |  |  |  |  |  |  |  |  |  |  |  |  |  |  |  | High | GIEIYESIIK | 1 |  | 0,0000 | 2,30 | 2 | 1164,65154 | 0,37 | 49,13 |
|  |  |  |  |  |  |  |  |  |  |  |  |  |  |  |  |  | High | cPMWLVIK | 2 | C1(Carbamidomethyl) | 0,0000 | 2,07 | 2 | 1046,55168 | -0,89 | 50,23 |
|  |  |  |  |  |  |  |  |  |  |  |  |  |  |  |  |  | Medium | LYDnTATEnLFK | 1 | N4(Deamidated); N9(Deamidated) | 0,0102 | 0,97 | 2 | 1430,67803 | 6,58 | 49,93 |
| **2312** | D2X5K2 | DEAD-box RNA helicase | DEAD-box RNA helicase-like protein OS=Prunus persica GN=PRUPE_ppa006399mg PE=2 SV=1 - [D2X5K2_PRUPE] | 99% to GI:645265533 Prunus mume, E value 2e-146 | Prunus persica | Chloroplast | 11.05-Disease/Defense/Stress responses | 89,79 | 35,11 | 6 | 5 | 17 | 55 | 413 | 46,9 | 5,69 | High | GLDVIQQAQSGTGK | 4 |  | 0,0000 | 4,83 | 2 | 1401,73406 | 0,54 | 43,63 |
|  |  |  |  |  |  |  |  |  |  |  |  |  |  |  |  |  | High | GVAINFVTR | 2 |  | 0,0000 | 2,95 | 2 | 976,55736 | -0,13 | 45,17 |
|  |  |  |  |  |  |  |  |  |  |  |  |  |  |  |  |  | High | DELTLEGIK | 3 |  | 0,0000 | 2,61 | 2 | 1017,54570 | -0,61 | 47,46 |
|  |  |  |  |  |  |  |  |  |  |  |  |  |  |  |  |  | High | DHTVSATHGDMDQNTR | 3 |  | 0,0000 | 2,57 | 3 | 1784,76139 | -0,19 | 35,95 |
|  |  |  |  |  |  |  |  |  |  |  |  |  |  |  |  |  | High | VDWLTDK | 2 |  | 0,0000 | 2,56 | 2 | 876,44585 | -0,40 | 45,46 |
|  |  |  |  |  |  |  |  |  |  |  |  |  |  |  |  |  | High | VLITTDLLAR | 3 |  | 0,0000 | 2,55 | 2 | 1114,68279 | -0,27 | 49,57 |
|  |  |  |  |  |  |  |  |  |  |  |  |  |  |  |  |  | High | MLYDIQR | 3 |  | 0,0000 | 2,45 | 2 | 938,47612 | -0,35 | 44,04 |
|  |  |  |  |  |  |  |  |  |  |  |  |  |  |  |  |  | High | ALGDYLGVK | 3 |  | 0,0000 | 2,45 | 2 | 935,51946 | -0,26 | 47,95 |
|  |  |  |  |  |  |  |  |  |  |  |  |  |  |  |  |  | High | MFVLDEADEMLSR | 1 |  | 0,0000 | 2,38 | 2 | 1555,71379 | 0,39 | 52,14 |
|  |  |  |  |  |  |  |  |  |  |  |  |  |  |  |  |  | High | mLYDIQR | 2 | M1(Oxidation) | 0,0000 | 2,31 | 2 | 954,47118 | -0,20 | 42,18 |
|  |  |  |  |  |  |  |  |  |  |  |  |  |  |  |  |  | High | RDELTLEGIK | 2 |  | 0,0000 | 2,31 | 2 | 1173,64751 | 0,06 | 43,68 |
|  |  |  |  |  |  |  |  |  |  |  |  |  |  |  |  |  | High | VFDMLR | 3 |  | 0,0000 | 2,28 | 2 | 780,40709 | -0,30 | 46,00 |
|  |  |  |  |  |  |  |  |  |  |  |  |  |  |  |  |  | High | KGVAINFVTR | 2 |  | 0,0000 | 2,14 | 2 | 1104,65141 | -0,95 | 41,92 |
|  |  |  |  |  |  |  |  |  |  |  |  |  |  |  |  |  | High | VHAcVGGTSVR | 4 | C4(Carbamidomethyl) | 0,0000 | 2,05 | 2 | 1142,57329 | -0,23 | 35,42 |
|  |  |  |  |  |  |  |  |  |  |  |  |  |  |  |  |  | Medium | QSLRPDYIK | 3 |  | 0,0000 | 1,95 | 3 | 1119,61512 | -0,54 | 39,86 |
|  |  |  |  |  |  |  |  |  |  |  |  |  |  |  |  |  | Medium | DHTVSATHGDmDQNTR | 4 | M11(Oxidation) | 0,0000 | 1,93 | 3 | 1800,75638 | -0,14 | 34,01 |
|  |  |  |  |  |  |  |  |  |  |  |  |  |  |  |  |  | Medium | ELAQQIEK | 3 |  | 0,0000 | 1,88 | 2 | 958,51976 | -0,70 | 40,29 |
|  |  |  |  |  |  |  |  |  |  |  |  |  |  |  |  |  | Medium | VFDmLR | 6 | M4(Oxidation) | 0,0000 | 1,55 | 2 | 796,40251 | 0,35 | 41,76 |
|  |  |  |  |  |  |  |  |  |  |  |  |  |  |  |  |  | Medium | GIVPFcK | 1 | C6(Carbamidomethyl) | 0,0000 | 1,52 | 2 | 820,43834 | -0,33 | 45,48 |
|  |  |  |  |  |  |  |  |  |  |  |  |  |  |  |  |  | Medium | QFYVNVDK | 1 |  | 0,0000 | 0,99 | 2 | 1012,50945 | -0,40 | 42,64 |
| **2319** | M5XCC8 | dihydrolipoyllysine-residue acetyltransferase component 3 of pyruvate dehydrogenase complex | Uncharacterized protein OS=Prunus persica GN=PRUPE_ppa003847mg PE=3 SV=1 - [M5XCC8_PRUPE] | 98% to GI:645244029 Prunus mume, E value 0 | Prunus mume | Mitochondrion | 02.01-Energy/Glycolysis | 58,99 | 28,86 | 2 | 16 | 17 | 32 | 544 | 58,8 | 7,96 | High | ADVEEYLASR | 2 |  | 0,0000 | 3,67 | 2 | 1152,55303 | -0,14 | 45,91 |
|  |  |  |  |  |  |  |  |  |  |  |  |  |  |  |  |  | High | GQLNALQEASGGK | 2 |  | 0,0000 | 3,26 | 2 | 1272,65447 | 0,11 | 41,05 |
|  |  |  |  |  |  |  |  |  |  |  |  |  |  |  |  |  | High | FKDYTPSASGASAAAAK | 1 |  | 0,0000 | 3,20 | 3 | 1642,80539 | -1,10 | 38,25 |
|  |  |  |  |  |  |  |  |  |  |  |  |  |  |  |  |  | High | GLSSIAEEVR | 2 |  | 0,0000 | 2,88 | 2 | 1060,56291 | -0,43 | 44,24 |
|  |  |  |  |  |  |  |  |  |  |  |  |  |  |  |  |  | High | VPQcNSSWTDDHIR | 1 | C4(Carbamidomethyl) | 0,0000 | 2,87 | 3 | 1714,75925 | -0,59 | 40,31 |
|  |  |  |  |  |  |  |  |  |  |  |  |  |  |  |  |  | High | EVVEEPVTSPEPK | 3 |  | 0,0000 | 2,83 | 2 | 1439,72698 | 0,34 | 40,44 |
|  |  |  |  |  |  |  |  |  |  |  |  |  |  |  |  |  | High | VSKPTAAAPSGDR | 5 |  | 0,0000 | 2,79 | 3 | 1256,65921 | -0,16 | 35,60 |
|  |  |  |  |  |  |  |  |  |  |  |  |  |  |  |  |  | High | KEVVEEPVTSPEPK | 1 |  | 0,0000 | 2,69 | 3 | 1567,82132 | -0,09 | 38,58 |
|  |  |  |  |  |  |  |  |  |  |  |  |  |  |  |  |  | High | DYTPSASGASAAAAK | 1 |  | 0,0000 | 2,58 | 2 | 1367,64433 | 0,38 | 38,64 |
|  |  |  |  |  |  |  |  |  |  |  |  |  |  |  |  |  | High | AAALALQK | 2 |  | 0,0000 | 2,30 | 2 | 785,48784 | -0,22 | 39,26 |
|  |  |  |  |  |  |  |  |  |  |  |  |  |  |  |  |  | High | VIDGAIGAEWLK | 1 |  | 0,0000 | 2,05 | 2 | 1271,70061 | 0,88 | 49,21 |
|  |  |  |  |  |  |  |  |  |  |  |  |  |  |  |  |  | Medium | GYIENPESmLL | 2 | M9(Oxidation) | 0,0000 | 1,74 | 2 | 1281,60234 | -0,66 | 50,62 |
|  |  |  |  |  |  |  |  |  |  |  |  |  |  |  |  |  | Medium | GTGPDGSIVK | 3 |  | 0,0000 | 1,70 | 2 | 930,48870 | -0,48 | 37,73 |
|  |  |  |  |  |  |  |  |  |  |  |  |  |  |  |  |  | Medium | IFASPLAR | 2 |  | 0,0000 | 1,51 | 2 | 874,51402 | -0,61 | 45,17 |
|  |  |  |  |  |  |  |  |  |  |  |  |  |  |  |  |  | Medium | LSVNDLVIK | 2 |  | 0,0000 | 1,45 | 2 | 1000,60283 | -0,94 | 47,60 |
|  |  |  |  |  |  |  |  |  |  |  |  |  |  |  |  |  | Medium | VPLSSIK | 1 |  | 0,0208 | 0,94 | 2 | 743,46605 | -0,20 | 40,05 |
| **2609** | M5W1V2 | succinate dehydrogenase [ubiquinone] flavoprotein | Uncharacterized protein OS=Prunus persica GN=PRUPE_ppa002787mg PE=4 SV=1 - [M5W1V2_PRUPE] | 99% to GI:645273001 Prunus mume, E value 0 | Prunus mume | Mitochondrion | 02.10-Energy/TCA pathway | 54,67 | 14,53 | 1 | 8 | 8 | 22 | 633 | 69,7 | 6,39 | High | TQETLEEGSQLIDK | 2 |  | 0,0000 | 4,35 | 2 | 1590,78716 | 0,86 | 43,33 |
|  |  |  |  |  |  |  |  |  |  |  |  |  |  |  |  |  | High | NSNGSLPTSQIR | 2 |  | 0,0000 | 3,23 | 2 | 1273,64983 | 0,22 | 40,23 |
|  |  |  |  |  |  |  |  |  |  |  |  |  |  |  |  |  | High | AFGGQSLDFGK | 2 |  | 0,0000 | 3,03 | 2 | 1126,55254 | -0,24 | 45,66 |
|  |  |  |  |  |  |  |  |  |  |  |  |  |  |  |  |  | High | IMQNNAAVFR | 3 |  | 0,0000 | 2,89 | 2 | 1163,59929 | 0,21 | 41,33 |
|  |  |  |  |  |  |  |  |  |  |  |  |  |  |  |  |  | High | FQAASTILATGGYGR | 1 |  | 0,0000 | 2,64 | 2 | 1512,78081 | 0,15 | 46,49 |
|  |  |  |  |  |  |  |  |  |  |  |  |  |  |  |  |  | High | DSGEQTIAWLDK | 2 |  | 0,0000 | 2,59 | 2 | 1362,65447 | 0,60 | 48,24 |
|  |  |  |  |  |  |  |  |  |  |  |  |  |  |  |  |  | High | NSnGSLPTSQIR | 4 | N3(Deamidated) | 0,0000 | 2,43 | 2 | 1274,63432 | 0,59 | 41,68 |
|  |  |  |  |  |  |  |  |  |  |  |  |  |  |  |  |  | High | ImQNNAAVFR | 3 | M2(Oxidation) | 0,0000 | 2,34 | 2 | 1179,59404 | 0,06 | 40,88 |
|  |  |  |  |  |  |  |  |  |  |  |  |  |  |  |  |  | High | AWESFDNVQVK | 2 |  | 0,0000 | 2,27 | 2 | 1322,63750 | -0,08 | 46,73 |
|  |  |  |  |  |  |  |  |  |  |  |  |  |  |  |  |  | Medium | SMTMEIR | 1 |  | 0,0000 | 1,57 | 2 | 867,40605 | -0,33 | 41,22 |
|  | M5XDI1 | phosphoglucomutase | Uncharacterized protein OS=Prunus persica GN=PRUPE_ppa003354mg PE=3 SV=1 - [M5XDI1_PRUPE] | 99% to GI:645229375 Prunus mume, E value 0 | Prunus mume | Cytoplasm | 02.01-Energy/Glycolysis | 46,98 | 24,91 | 1 | 14 | 14 | 25 | 582 | 63,3 | 6,07 | High | STTQDEPPEFGAAADGDADR | 2 |  | 0,0000 | 4,83 | 2 | 2049,86601 | 1,32 | 43,59 |
|  |  |  |  |  |  |  |  |  |  |  |  |  |  |  |  |  | High | YNMEnGGPAPESITDK | 2 | N5(Deamidated) | 0,0000 | 3,78 | 2 | 1723,74285 | -3,01 | 42,46 |
|  |  |  |  |  |  |  |  |  |  |  |  |  |  |  |  |  | High | YNMENGGPAPESITDK | 1 |  | 0,0000 | 3,69 | 2 | 1722,76592 | 1,10 | 42,08 |
|  |  |  |  |  |  |  |  |  |  |  |  |  |  |  |  |  | High | YDYENVDAGAAK | 1 |  | 0,0000 | 3,12 | 2 | 1315,58171 | 1,20 | 40,21 |
|  |  |  |  |  |  |  |  |  |  |  |  |  |  |  |  |  | High | LSGTGSEGATIR | 2 |  | 0,0000 | 2,98 | 2 | 1148,59062 | -0,02 | 38,18 |
|  |  |  |  |  |  |  |  |  |  |  |  |  |  |  |  |  | High | YLFEDGSR | 3 |  | 0,0000 | 2,31 | 2 | 986,45745 | -0,38 | 43,56 |
|  |  |  |  |  |  |  |  |  |  |  |  |  |  |  |  |  | High | ESSLLNcVPK | 2 | C7(Carbamidomethyl) | 0,0000 | 2,15 | 2 | 1146,58269 | 0,27 | 44,02 |
|  |  |  |  |  |  |  |  |  |  |  |  |  |  |  |  |  | High | LYIEQYEK | 2 |  | 0,0000 | 2,05 | 2 | 1085,55120 | -0,16 | 42,58 |
|  |  |  |  |  |  |  |  |  |  |  |  |  |  |  |  |  | Medium | IFVEELGAK | 1 |  | 0,0000 | 1,98 | 2 | 1005,56096 | -0,62 | 45,57 |
|  |  |  |  |  |  |  |  |  |  |  |  |  |  |  |  |  | Medium | LVSVEDIVR | 1 |  | 0,0000 | 1,95 | 2 | 1029,59368 | -0,26 | 46,87 |
|  |  |  |  |  |  |  |  |  |  |  |  |  |  |  |  |  | Medium | EDFGGGHPDPNLTYAK | 1 |  | 0,0000 | 1,90 | 3 | 1717,78012 | -0,92 | 40,97 |
|  |  |  |  |  |  |  |  |  |  |  |  |  |  |  |  |  | Medium | MQEFTGR | 2 |  | 0,0000 | 1,79 | 2 | 868,39781 | -0,46 | 39,18 |
|  |  |  |  |  |  |  |  |  |  |  |  |  |  |  |  |  | Medium | LYIEqYEKDPSK | 1 | Q5(Deamidated) | 0,0541 | 1,75 | 2 | 1513,73088 | -7,42 | 41,52 |
|  |  |  |  |  |  |  |  |  |  |  |  |  |  |  |  |  | Medium | mQEFTGR | 1 | M1(Oxidation) | 0,0000 | 1,42 | 2 | 884,39379 | 0,75 | 36,98 |
|  |  |  |  |  |  |  |  |  |  |  |  |  |  |  |  |  | Medium | QHWATFGR | 1 |  | 0,0000 | 1,29 | 3 | 1002,49070 | 0,23 | 40,51 |
|  |  |  |  |  |  |  |  |  |  |  |  |  |  |  |  |  | Medium | NMILGK | 2 |  | 0,0000 | 1,08 | 2 | 675,38609 | 0,36 | 40,18 |
| **2615** | M5VWE8 | Malic enzyme | Malic enzyme OS=Prunus persica GN=PRUPE_ppa003142mg PE=3 SV=1 - [M5VWE8_PRUPE] |  | Prunus persica | Chloroplast | 02.30-Energy/Photosynthesis | 19,24 | 12,67 | 11 | 9 | 10 | 21 | 592 | 65,6 | 5,99 | High | AYELGLATR | 2 |  | 0,0000 | 3,31 | 2 | 993,53581 | -0,60 | 42,48 |
|  |  |  |  |  |  |  |  |  |  |  |  |  |  |  |  |  | High | TVQVIVVTDGER | 1 |  | 0,0000 | 3,06 | 2 | 1315,72319 | 1,16 | 42,07 |
|  |  |  |  |  |  |  |  |  |  |  |  |  |  |  |  |  | High | DSLQQFK | 1 |  | 0,0047 | 2,14 | 2 | 865,44121 | -0,27 | 38,67 |
|  |  |  |  |  |  |  |  |  |  |  |  |  |  |  |  |  | High | VLEVLK | 3 |  | 0,0000 | 2,14 | 2 | 700,46050 | 0,15 | 41,20 |
|  |  |  |  |  |  |  |  |  |  |  |  |  |  |  |  |  | High | IWLVDSK | 1 |  | 0,0000 | 2,02 | 2 | 860,48754 | -0,16 | 44,71 |
|  |  |  |  |  |  |  |  |  |  |  |  |  |  |  |  |  | Medium | GLIVSSR | 3 |  | 0,0000 | 1,67 | 2 | 731,44109 | 0,05 | 38,25 |
|  |  |  |  |  |  |  |  |  |  |  |  |  |  |  |  |  | Medium | GLAFTEK | 2 |  | 0,0000 | 1,32 | 2 | 765,41423 | 0,08 | 39,76 |
|  |  |  |  |  |  |  |  |  |  |  |  |  |  |  |  |  | Medium | LMNnIR | 2 | N4(Deamidated) | 0,0000 | 1,26 | 2 | 761,39745 | -0,04 | 37,16 |
|  |  |  |  |  |  |  |  |  |  |  |  |  |  |  |  |  | Medium | QYQVPLQK | 2 |  | 0,0000 | 1,23 | 2 | 1003,55669 | -0,44 | 38,44 |
|  |  |  |  |  |  |  |  |  |  |  |  |  |  |  |  |  | Medium | FGSIFR | 3 |  | 0,0000 | 1,12 | 2 | 726,39342 | 0,07 | 44,51 |
|  |  |  |  |  |  |  |  |  |  |  |  |  |  |  |  |  | Medium | LMNNIR | 1 |  | 0,0208 | 0,94 | 2 | 760,41301 | -0,59 | 35,84 |
| **2617** | M5W741 | oligopeptidase | Uncharacterized protein OS=Prunus persica GN=PRUPE_ppa001572mg PE=3 SV=1 - [M5W741_PRUPE] | 90% to GI:694437692 Pyrus x bretschneideri, E value 0 | Pyrus x bretschneideri | Cytosol | 06.13-Protein destination and storage/Proteolysis | 69,24 | 24,13 | 1 | 7 | 22 | 42 | 800 | 90,2 | 6,89 | High | LEGDLEELER | 1 |  | 0,0000 | 3,07 | 2 | 1202,59001 | 0,02 | 44,30 |
|  |  |  |  |  |  |  |  |  |  |  |  |  |  |  |  |  | High | FQENVLDATK | 3 |  | 0,0000 | 3,00 | 2 | 1164,58965 | 0,06 | 43,16 |
|  |  |  |  |  |  |  |  |  |  |  |  |  |  |  |  |  | High | SAIEEVQPEK | 2 |  | 0,0000 | 2,91 | 2 | 1129,57329 | -0,28 | 38,46 |
|  |  |  |  |  |  |  |  |  |  |  |  |  |  |  |  |  | High | QDEGLVAGIR | 2 |  | 0,0000 | 2,72 | 2 | 1057,56365 | -0,06 | 42,65 |
|  |  |  |  |  |  |  |  |  |  |  |  |  |  |  |  |  | High | LLNYNNYAEVSMATK | 1 |  | 0,0000 | 2,36 | 2 | 1730,84294 | 0,63 | 46,11 |
|  |  |  |  |  |  |  |  |  |  |  |  |  |  |  |  |  | High | AEELLEK | 2 |  | 0,0000 | 2,28 | 2 | 831,44591 | 0,05 | 39,87 |
|  |  |  |  |  |  |  |  |  |  |  |  |  |  |  |  |  | High | HnGLLATASA | 1 | N2(Deamidated) | 0,0000 | 2,22 | 2 | 955,48406 | -0,34 | 44,49 |
|  |  |  |  |  |  |  |  |  |  |  |  |  |  |  |  |  | High | AGSLSLR | 2 |  | 0,0000 | 2,09 | 2 | 703,40990 | 0,21 | 39,38 |
|  |  |  |  |  |  |  |  |  |  |  |  |  |  |  |  |  | High | IEQELER | 3 |  | 0,0000 | 2,05 | 2 | 916,47283 | -0,72 | 36,77 |
|  |  |  |  |  |  |  |  |  |  |  |  |  |  |  |  |  | High | TQVIPPLPEDR | 3 |  | 0,0000 | 2,05 | 2 | 1264,68987 | 0,18 | 45,62 |
|  |  |  |  |  |  |  |  |  |  |  |  |  |  |  |  |  | Medium | HNGLLATASA | 2 |  | 0,0000 | 1,99 | 2 | 954,49999 | -0,39 | 41,09 |
|  |  |  |  |  |  |  |  |  |  |  |  |  |  |  |  |  | Medium | TVEPTWPK | 2 |  | 0,0000 | 1,87 | 2 | 957,50377 | -0,29 | 42,77 |
|  |  |  |  |  |  |  |  |  |  |  |  |  |  |  |  |  | Medium | ASSGDMDNTAIIDSILK | 1 |  | 0,0000 | 1,84 | 2 | 1750,85564 | 1,59 | 51,30 |
|  |  |  |  |  |  |  |  |  |  |  |  |  |  |  |  |  | Medium | DSLELR | 2 |  | 0,0000 | 1,74 | 2 | 732,38872 | 0,05 | 40,79 |
|  |  |  |  |  |  |  |  |  |  |  |  |  |  |  |  |  | Medium | RIVESQIK | 1 |  | 0,0000 | 1,74 | 2 | 972,58385 | 0,15 | 36,08 |
|  |  |  |  |  |  |  |  |  |  |  |  |  |  |  |  |  | Medium | DTLMSIAK | 3 |  | 0,0000 | 1,73 | 2 | 878,46514 | -0,11 | 44,92 |
|  |  |  |  |  |  |  |  |  |  |  |  |  |  |  |  |  | Medium | IVESQIK | 1 |  | 0,0000 | 1,66 | 2 | 816,48241 | -0,21 | 36,41 |
|  |  |  |  |  |  |  |  |  |  |  |  |  |  |  |  |  | Medium | FASVDLK | 2 |  | 0,0000 | 1,66 | 2 | 779,42974 | -0,11 | 42,20 |
|  |  |  |  |  |  |  |  |  |  |  |  |  |  |  |  |  | Medium | EAVLcGVSLEDDK | 1 | C5(Carbamidomethyl) | 0,0000 | 1,63 | 2 | 1434,68059 | 1,69 | 45,49 |
|  |  |  |  |  |  |  |  |  |  |  |  |  |  |  |  |  | Medium | EAVLcGVSLEDDKR | 1 | C5(Carbamidomethyl) | 0,0000 | 1,57 | 3 | 1590,77982 | 0,34 | 42,31 |
|  |  |  |  |  |  |  |  |  |  |  |  |  |  |  |  |  | Medium | DTLmSIAK | 1 | M4(Oxidation) | 0,0000 | 1,42 | 2 | 894,45970 | -0,50 | 40,54 |
|  |  |  |  |  |  |  |  |  |  |  |  |  |  |  |  |  | Medium | LVVPLEK | 3 |  | 0,0000 | 1,36 | 2 | 797,51305 | -0,14 | 44,77 |
|  |  |  |  |  |  |  |  |  |  |  |  |  |  |  |  |  | Medium | SHVLSR | 1 |  | 0,0000 | 1,28 | 2 | 698,39452 | 0,14 | 32,72 |
|  |  |  |  |  |  |  |  |  |  |  |  |  |  |  |  |  | Medium | AILESPDWQTLTEAR | 1 |  | 0,0000 | 1,19 | 2 | 1729,87847 | 1,64 | 49,31 |
| **2701** | M5VP31 | HSP70 | Uncharacterized protein OS=Prunus persica GN=PRUPE_ppa001973mg PE=3 SV=1 - [M5VP31_PRUPE] | 96% to GI:657963243 Malus domestica, E value 0 | Malus domestica | Mitochondrion | 06.01-Protein destination and storage/Folding and stability | 90,58 | 30,52 | 3 | 18 | 19 | 40 | 734 | 79,5 | 8,46 | High | GSGGDSSSGGSGTEGGDQAPEADYEEVKK | 2 |  | 0,0000 | 5,13 | 3 | 2758,16013 | 1,51 | 38,70 |
|  |  |  |  |  |  |  |  |  |  |  |  |  |  |  |  |  | High | SQVFSTAADNQTQVGIK | 2 |  | 0,0000 | 4,01 | 2 | 1793,90459 | 0,95 | 43,75 |
|  |  |  |  |  |  |  |  |  |  |  |  |  |  |  |  |  | High | NSADTTIYSIEK | 3 |  | 0,0000 | 3,77 | 2 | 1341,65386 | 0,42 | 42,73 |
|  |  |  |  |  |  |  |  |  |  |  |  |  |  |  |  |  | High | QAVTNPTNTVFGTK | 3 |  | 0,0000 | 3,62 | 2 | 1477,76592 | 0,90 | 43,83 |
|  |  |  |  |  |  |  |  |  |  |  |  |  |  |  |  |  | High | VQEVVTQIFGK | 1 |  | 0,0000 | 3,54 | 2 | 1247,69963 | 0,13 | 47,24 |
|  |  |  |  |  |  |  |  |  |  |  |  |  |  |  |  |  | High | SSGGLSEDEIEK | 2 |  | 0,0000 | 3,41 | 2 | 1250,57500 | 0,21 | 40,41 |
|  |  |  |  |  |  |  |  |  |  |  |  |  |  |  |  |  | High | GELLVGTPAK | 2 |  | 0,0000 | 3,25 | 2 | 984,57207 | -0,42 | 42,82 |
|  |  |  |  |  |  |  |  |  |  |  |  |  |  |  |  |  | High | IINEPTAAALSYGMNNK | 2 |  | 0,0000 | 2,80 | 2 | 1806,90788 | 1,30 | 46,84 |
|  |  |  |  |  |  |  |  |  |  |  |  |  |  |  |  |  | High | TTPSVVAFNQK | 2 |  | 0,0000 | 2,45 | 2 | 1191,63689 | 0,02 | 42,92 |
|  |  |  |  |  |  |  |  |  |  |  |  |  |  |  |  |  | High | TAIGGDNAEEIK | 2 |  | 0,0000 | 2,40 | 2 | 1217,60112 | 0,19 | 40,01 |
|  |  |  |  |  |  |  |  |  |  |  |  |  |  |  |  |  | High | EIEDAVAELR | 4 |  | 0,0000 | 2,36 | 2 | 1144,58464 | 0,11 | 47,33 |
|  |  |  |  |  |  |  |  |  |  |  |  |  |  |  |  |  | High | IINEPTAAALSYGmNNK | 1 | M14(Oxidation) | 0,0000 | 2,33 | 2 | 1822,90264 | 1,20 | 44,51 |
|  |  |  |  |  |  |  |  |  |  |  |  |  |  |  |  |  | Medium | MKETAEAYLGK | 1 |  | 0,0000 | 2,19 | 3 | 1240,62329 | -0,78 | 39,51 |
|  |  |  |  |  |  |  |  |  |  |  |  |  |  |  |  |  | High | DVDEVLLVGGMTR | 2 |  | 0,0000 | 2,18 | 2 | 1403,72124 | 0,90 | 50,81 |
|  |  |  |  |  |  |  |  |  |  |  |  |  |  |  |  |  | High | IAGLDVQR | 1 |  | 0,0000 | 2,03 | 2 | 871,49950 | -0,16 | 41,39 |
|  |  |  |  |  |  |  |  |  |  |  |  |  |  |  |  |  | Medium | ETAEAYLGK | 2 |  | 0,0000 | 1,80 | 2 | 981,48857 | -0,22 | 41,40 |
|  |  |  |  |  |  |  |  |  |  |  |  |  |  |  |  |  | Medium | DVDEVLLVGGmTR | 1 | M11(Oxidation) | 0,0000 | 1,66 | 2 | 1419,71501 | 0,08 | 48,76 |
|  |  |  |  |  |  |  |  |  |  |  |  |  |  |  |  |  | Medium | TEGIDLAK | 1 |  | 0,0000 | 1,63 | 2 | 846,45671 | -0,07 | 40,31 |
|  |  |  |  |  |  |  |  |  |  |  |  |  |  |  |  |  | Medium | DKIPSEVAK | 2 |  | 0,0000 | 1,61 | 3 | 986,55182 | 0,08 | 36,34 |
|  |  |  |  |  |  |  |  |  |  |  |  |  |  |  |  |  | Medium | ALIDIR | 2 |  | 0,0000 | 1,07 | 2 | 700,43517 | -0,11 | 46,41 |
| **2703** | M5WQS2 | V-type proton ATPase subunit A | Uncharacterized protein OS=Prunus persica GN=PRUPE_ppa002892mg PE=3 SV=1 - [M5WQS2_PRUPE] | 99% to GI:645243457 Prunus mume, E value 0 | Prunus mume | Cytoplasm | 07.22-Transporters/Transport ATPases | 140,13 | 39,33 | 2 | 23 | 24 | 75 | 623 | 68,6 | 5,45 | High | TTLVANTSNMPVAAR | 3 |  | 0,0000 | 4,20 | 2 | 1545,80705 | 1,05 | 42,95 |
|  |  |  |  |  |  |  |  |  |  |  |  |  |  |  |  |  | High | FEDPAEGEEVIVGK | 3 |  | 0,0000 | 3,81 | 2 | 1518,73345 | 0,74 | 45,76 |
|  |  |  |  |  |  |  |  |  |  |  |  |  |  |  |  |  | High | YSNSDTVVYVGcGER | 2 | C12(Carbamidomethyl) | 0,0000 | 3,67 | 2 | 1705,75078 | 1,23 | 42,57 |
|  |  |  |  |  |  |  |  |  |  |  |  |  |  |  |  |  | High | DMGYNVSMMADSTSR | 2 |  | 0,0000 | 3,67 | 2 | 1664,67327 | 1,13 | 46,52 |
|  |  |  |  |  |  |  |  |  |  |  |  |  |  |  |  |  | High | LAEmPADSGYPAYLAAR | 2 | M4(Oxidation) | 0,0000 | 3,61 | 2 | 1811,86528 | 1,07 | 44,86 |
|  |  |  |  |  |  |  |  |  |  |  |  |  |  |  |  |  | High | TTLVANTSNmPVAAR | 4 | M10(Oxidation) | 0,0000 | 3,58 | 2 | 1561,80144 | 0,70 | 40,18 |
|  |  |  |  |  |  |  |  |  |  |  |  |  |  |  |  |  | High | DALAEGDKITLETAK | 2 |  | 0,0000 | 3,36 | 2 | 1574,82878 | 0,95 | 42,90 |
|  |  |  |  |  |  |  |  |  |  |  |  |  |  |  |  |  | High | YSnSDTVVYVGcGER | 1 | N3(Deamidated); C12(Carbamidomethyl) | 0,0000 | 3,36 | 2 | 1706,72661 | -3,56 | 43,36 |
|  |  |  |  |  |  |  |  |  |  |  |  |  |  |  |  |  | High | EDYLAQNAFTPYDK | 2 |  | 0,0000 | 3,29 | 2 | 1674,76628 | 0,98 | 46,94 |
|  |  |  |  |  |  |  |  |  |  |  |  |  |  |  |  |  | High | DMGYNVSMmADSTSR | 6 | M9(Oxidation) | 0,0000 | 3,26 | 2 | 1680,66765 | 0,80 | 44,55 |
|  |  |  |  |  |  |  |  |  |  |  |  |  |  |  |  |  | High | VAADTPLLTGQR | 6 |  | 0,0000 | 3,10 | 2 | 1241,68523 | 0,27 | 43,05 |
|  |  |  |  |  |  |  |  |  |  |  |  |  |  |  |  |  | High | ISGDVYIPR | 5 |  | 0,0000 | 3,08 | 2 | 1019,55187 | -0,19 | 43,55 |
|  |  |  |  |  |  |  |  |  |  |  |  |  |  |  |  |  | High | LAEMPADSGYPAYLAAR | 2 |  | 0,0000 | 3,07 | 2 | 1795,87041 | 1,10 | 47,77 |
|  |  |  |  |  |  |  |  |  |  |  |  |  |  |  |  |  | High | LGDLFYR | 3 |  | 0,0000 | 2,55 | 2 | 883,46721 | -0,06 | 47,84 |
|  |  |  |  |  |  |  |  |  |  |  |  |  |  |  |  |  | High | LHEDLTSGFR | 3 |  | 0,0000 | 2,41 | 2 | 1174,58513 | -0,03 | 40,82 |
|  |  |  |  |  |  |  |  |  |  |  |  |  |  |  |  |  | High | ITYVAPPGQYSLK | 3 |  | 0,0000 | 2,38 | 2 | 1436,77971 | 0,89 | 44,11 |
|  |  |  |  |  |  |  |  |  |  |  |  |  |  |  |  |  | High | DILWEFQPK | 1 |  | 0,0000 | 2,21 | 2 | 1175,60954 | -0,04 | 50,74 |
|  |  |  |  |  |  |  |  |  |  |  |  |  |  |  |  |  | High | TVISQALSK | 3 |  | 0,0000 | 2,12 | 2 | 946,55644 | -0,39 | 43,27 |
|  |  |  |  |  |  |  |  |  |  |  |  |  |  |  |  |  | Medium | LTTFEDSEK | 2 |  | 0,0000 | 2,00 | 2 | 1069,50444 | -0,37 | 40,28 |
|  |  |  |  |  |  |  |  |  |  |  |  |  |  |  |  |  | Medium | ISYSLIK | 3 |  | 0,0000 | 1,82 | 2 | 823,49242 | 0,02 | 43,15 |
|  |  |  |  |  |  |  |  |  |  |  |  |  |  |  |  |  | Medium | LASFYER | 2 |  | 0,0000 | 1,81 | 2 | 885,44634 | -0,21 | 41,14 |
|  |  |  |  |  |  |  |  |  |  |  |  |  |  |  |  |  | Medium | WAEALR | 2 |  | 0,0000 | 1,76 | 2 | 745,39910 | -0,13 | 40,58 |
|  |  |  |  |  |  |  |  |  |  |  |  |  |  |  |  |  | Medium | ESEYGYVR | 2 |  | 0,0000 | 1,58 | 2 | 1002,45226 | -0,46 | 39,78 |
|  |  |  |  |  |  |  |  |  |  |  |  |  |  |  |  |  | Medium | ITLETAK | 2 |  | 0,0000 | 1,49 | 2 | 775,45592 | -0,14 | 39,20 |
|  |  |  |  |  |  |  |  |  |  |  |  |  |  |  |  |  | Medium | tTLVAnTSnMPVAAR | 1 | N-Term(Acetyl); N6(Deamidated); N9(Deamidated) | 0,0438 | 1,31 | 2 | 1589,79729 | 8,34 | 40,25 |
|  |  |  |  |  |  |  |  |  |  |  |  |  |  |  |  |  | Medium | SVWMMR | 2 |  | 0,0000 | 1,31 | 2 | 809,37956 | -0,21 | 45,35 |
|  |  |  |  |  |  |  |  |  |  |  |  |  |  |  |  |  | Medium | FcPFYK | 2 | C2(Carbamidomethyl) | 0,0000 | 1,18 | 2 | 861,39617 | -0,27 | 44,18 |
|  |  |  |  |  |  |  |  |  |  |  |  |  |  |  |  |  | Medium | DTVLELEFQGVK | 1 |  | 0,0000 | 1,06 | 2 | 1377,71807 | -5,83 | 50,39 |
| **2704** | M5VP31 | HSP70 | Uncharacterized protein OS=Prunus persica GN=PRUPE_ppa001973mg PE=3 SV=1 - [M5VP31_PRUPE] | 96% to GI:657963243 Malus domestica, E value 0 | Malus domestica | Mitochondrion | 06.01-Protein destination and storage/Folding and stability | 273,80 | 36,10 | 3 | 29 | 29 | 112 | 734 | 79,5 | 8,46 | High | GSGGDSSSGGSGTEGGDQAPEADYEEVKK | 3 |  | 0,0000 | 5,37 | 3 | 2758,15976 | 1,38 | 38,24 |
|  |  |  |  |  |  |  |  |  |  |  |  |  |  |  |  |  | High | IINEPTAAALSYGMNNK | 6 |  | 0,0000 | 4,90 | 2 | 1806,90788 | 1,30 | 47,61 |
|  |  |  |  |  |  |  |  |  |  |  |  |  |  |  |  |  | High | IINEPTAAALSYGmNNK | 4 | M14(Oxidation) | 0,0000 | 4,69 | 2 | 1822,90288 | 1,33 | 45,99 |
|  |  |  |  |  |  |  |  |  |  |  |  |  |  |  |  |  | High | GVNPDEAVAMGAAIQGGILR | 1 |  | 0,0000 | 4,30 | 2 | 1939,00884 | 1,10 | 50,49 |
|  |  |  |  |  |  |  |  |  |  |  |  |  |  |  |  |  | High | SQVFSTAADNQTQVGIK | 7 |  | 0,0000 | 4,23 | 2 | 1793,90508 | 1,22 | 43,53 |
|  |  |  |  |  |  |  |  |  |  |  |  |  |  |  |  |  | High | MKETAEAYLGK | 4 |  | 0,0000 | 3,93 | 3 | 1240,62412 | -0,12 | 40,02 |
|  |  |  |  |  |  |  |  |  |  |  |  |  |  |  |  |  | High | VQEVVTQIFGK | 5 |  | 0,0000 | 3,68 | 2 | 1247,69988 | 0,32 | 48,15 |
|  |  |  |  |  |  |  |  |  |  |  |  |  |  |  |  |  | High | NSADTTIYSIEK | 5 |  | 0,0000 | 3,60 | 2 | 1341,65373 | 0,33 | 43,74 |
|  |  |  |  |  |  |  |  |  |  |  |  |  |  |  |  |  | High | GVNPDEAVAmGAAIQGGILR | 2 | M10(Oxidation) | 0,0000 | 3,43 | 2 | 1955,00420 | 1,32 | 47,45 |
|  |  |  |  |  |  |  |  |  |  |  |  |  |  |  |  |  | High | GSGGDSSSGGSGTEGGDQAPEADYEEVK | 3 |  | 0,0000 | 3,37 | 2 | 2630,06255 | 0,60 | 40,47 |
|  |  |  |  |  |  |  |  |  |  |  |  |  |  |  |  |  | High | GELLVGTPAK | 5 |  | 0,0000 | 3,23 | 2 | 984,57219 | -0,29 | 42,82 |
|  |  |  |  |  |  |  |  |  |  |  |  |  |  |  |  |  | High | mKETAEAYLGK | 4 | M1(Oxidation) | 0,0000 | 3,17 | 3 | 1256,61847 | -0,56 | 39,00 |
|  |  |  |  |  |  |  |  |  |  |  |  |  |  |  |  |  | High | QAVTNPTNTVFGTK | 5 |  | 0,0000 | 3,08 | 2 | 1477,76579 | 0,81 | 44,77 |
|  |  |  |  |  |  |  |  |  |  |  |  |  |  |  |  |  | High | RTEGIDLAK | 1 |  | 0,0000 | 2,86 | 2 | 1002,55766 | -0,23 | 37,55 |
|  |  |  |  |  |  |  |  |  |  |  |  |  |  |  |  |  | High | DVDEVLLVGGmTR | 4 | M11(Oxidation) | 0,0000 | 2,81 | 2 | 1419,71550 | 0,43 | 49,49 |
|  |  |  |  |  |  |  |  |  |  |  |  |  |  |  |  |  | High | AVITVPAYFNDAQR | 3 |  | 0,0000 | 2,74 | 2 | 1564,81365 | 1,13 | 48,92 |
|  |  |  |  |  |  |  |  |  |  |  |  |  |  |  |  |  | High | TAIGGDNAEEIK | 3 |  | 0,0000 | 2,65 | 2 | 1217,60112 | 0,19 | 38,96 |
|  |  |  |  |  |  |  |  |  |  |  |  |  |  |  |  |  | High | SSGGLSEDEIEK | 3 |  | 0,0000 | 2,62 | 2 | 1250,57500 | 0,21 | 42,07 |
|  |  |  |  |  |  |  |  |  |  |  |  |  |  |  |  |  | Medium | KSQVFSTAADNQTQVGIK | 1 |  | 0,0000 | 2,49 | 3 | 1921,99784 | -0,01 | 40,36 |
|  |  |  |  |  |  |  |  |  |  |  |  |  |  |  |  |  | High | TTPSVVAFNQK | 3 |  | 0,0000 | 2,29 | 2 | 1191,63689 | 0,02 | 43,94 |
|  |  |  |  |  |  |  |  |  |  |  |  |  |  |  |  |  | High | IAGLDVQR | 5 |  | 0,0000 | 2,13 | 2 | 871,50035 | 0,82 | 42,34 |
|  |  |  |  |  |  |  |  |  |  |  |  |  |  |  |  |  | High | EIEDAVAELR | 7 |  | 0,0000 | 2,12 | 2 | 1144,58476 | 0,22 | 48,38 |
|  |  |  |  |  |  |  |  |  |  |  |  |  |  |  |  |  | Medium | AVITVPAYFnDAQR | 3 | N10(Deamidated) | 0,0000 | 2,10 | 3 | 1565,79416 | -1,11 | 48,34 |
|  |  |  |  |  |  |  |  |  |  |  |  |  |  |  |  |  | High | TEGIDLAK | 1 |  | 0,0000 | 2,10 | 2 | 846,45659 | -0,21 | 40,31 |
|  |  |  |  |  |  |  |  |  |  |  |  |  |  |  |  |  | High | ETAEAYLGK | 2 |  | 0,0000 | 2,09 | 2 | 981,48845 | -0,35 | 41,31 |
|  |  |  |  |  |  |  |  |  |  |  |  |  |  |  |  |  | High | DKIPSEVAK | 4 |  | 0,0000 | 2,08 | 2 | 986,55138 | -0,37 | 37,22 |
|  |  |  |  |  |  |  |  |  |  |  |  |  |  |  |  |  | High | DVDEVLLVGGMTR | 4 |  | 0,0000 | 2,08 | 2 | 1403,72063 | 0,46 | 52,45 |
|  |  |  |  |  |  |  |  |  |  |  |  |  |  |  |  |  | Medium | TAIGGDNAEEIKAK | 1 |  | 0,0000 | 2,02 | 3 | 1416,73294 | -0,03 | 37,31 |
|  |  |  |  |  |  |  |  |  |  |  |  |  |  |  |  |  | Medium | KALIDIR | 2 |  | 0,0000 | 1,95 | 2 | 828,53020 | -0,01 | 40,53 |
|  |  |  |  |  |  |  |  |  |  |  |  |  |  |  |  |  | Medium | HLNITLTR | 3 |  | 0,0000 | 1,87 | 2 | 967,56804 | -0,34 | 41,33 |
|  |  |  |  |  |  |  |  |  |  |  |  |  |  |  |  |  | Medium | GELLVGTPAKR | 2 |  | 0,0000 | 1,49 | 3 | 1140,67289 | -0,63 | 39,48 |
|  |  |  |  |  |  |  |  |  |  |  |  |  |  |  |  |  | Medium | ALIDIR | 3 |  | 0,0000 | 1,36 | 2 | 700,43517 | -0,11 | 46,23 |
|  |  |  |  |  |  |  |  |  |  |  |  |  |  |  |  |  | Medium | RQAVTNPTNTVFGTK | 1 |  | 0,0074 | 1,34 | 3 | 1633,86509 | -0,38 | 40,41 |
|  |  |  |  |  |  |  |  |  |  |  |  |  |  |  |  |  | Medium | SqVFSTAADNqTQVGIK | 1 | Q2(Deamidated); Q11(Deamidated) | 0,5145 | 1,17 | 2 | 1795,87029 | -0,35 | 47,02 |
|  |  |  |  |  |  |  |  |  |  |  |  |  |  |  |  |  | Medium | EQQITIR | 1 |  | 0,0000 | 1,13 | 2 | 887,49462 | 0,08 | 39,16 |
| **3205** | A9YTE0 | Leucoanthocyanidin dioxygenase | Leucoanthocyanidin dioxygenase OS=Prunus persica GN=LDOX PE=2 SV=1 - [A9YTE0_PRUPE] |  | Prunus persica | Chloroplast | 20.99-Secondary metabolism/Others | 35,85 | 21,85 | 3 | 7 | 8 | 19 | 357 | 40,4 | 5,63 | High | VETLSSSGIATIPK | 3 |  | 0,0000 | 3,29 | 2 | 1402,77959 | 0,53 | 45,28 |
|  |  |  |  |  |  |  |  |  |  |  |  |  |  |  |  |  | High | SQEALLNK | 2 |  | 0,0000 | 2,69 | 2 | 902,49358 | -0,70 | 39,11 |
|  |  |  |  |  |  |  |  |  |  |  |  |  |  |  |  |  | High | VLSLGLGLEEGR | 2 |  | 0,0000 | 2,53 | 2 | 1242,70635 | 0,84 | 48,65 |
|  |  |  |  |  |  |  |  |  |  |  |  |  |  |  |  |  | High | AFFDLPIEQK | 2 |  | 0,0000 | 2,41 | 2 | 1207,63616 | 0,29 | 50,23 |
|  |  |  |  |  |  |  |  |  |  |  |  |  |  |  |  |  | High | STDGPQVPTIDLK | 3 |  | 0,0000 | 2,14 | 2 | 1370,71684 | 0,43 | 46,61 |
|  |  |  |  |  |  |  |  |  |  |  |  |  |  |  |  |  | High | TFAEHIQHK | 2 |  | 0,0000 | 2,12 | 2 | 1110,56914 | 0,03 | 35,72 |
|  |  |  |  |  |  |  |  |  |  |  |  |  |  |  |  |  | High | EIDSENENVR | 4 |  | 0,0000 | 2,02 | 2 | 1204,54460 | 0,43 | 36,34 |
| **3308** | M5W1Z4 | Guanosine nucleotide diphosphate dissociation inhibitor | Uncharacterized protein OS=Prunus persica GN=PRUPE_ppa005772mg PE=4 SV=1 - [M5W1Z4_PRUPE] | 99% to GI:645272258 Prunus mume, E value 0 | Prunus mume | Chloroplast | 08.99-Intracellular traffic/Others | 87,19 | 27,48 | 3 | 11 | 12 | 46 | 444 | 49,9 | 5,95 | High | VIGVTSEGETAR | 2 |  | 0,0000 | 3,47 | 2 | 1218,63262 | 0,08 | 38,52 |
|  |  |  |  |  |  |  |  |  |  |  |  |  |  |  |  |  | High | AVDGSFVYNK | 3 |  | 0,0000 | 3,26 | 2 | 1099,54167 | -0,20 | 42,12 |
|  |  |  |  |  |  |  |  |  |  |  |  |  |  |  |  |  | High | YLEEPALETVK | 4 |  | 0,0000 | 3,21 | 2 | 1291,67827 | 0,16 | 44,80 |
|  |  |  |  |  |  |  |  |  |  |  |  |  |  |  |  |  | High | FMMANGTLVR | 1 |  | 0,0000 | 3,19 | 2 | 1139,57024 | 0,15 | 44,50 |
|  |  |  |  |  |  |  |  |  |  |  |  |  |  |  |  |  | High | FmMAnGTLVR | 4 | M2(Oxidation); N5(Deamidated) | 0,0000 | 2,86 | 2 | 1156,55107 | 1,79 | 44,60 |
|  |  |  |  |  |  |  |  |  |  |  |  |  |  |  |  |  | High | VVcDPSYLPNK | 3 | C3(Carbamidomethyl) | 0,0000 | 2,83 | 2 | 1291,63579 | 0,50 | 41,73 |
|  |  |  |  |  |  |  |  |  |  |  |  |  |  |  |  |  | High | FMMAnGTLVR | 2 | N5(Deamidated) | 0,0000 | 2,80 | 2 | 1140,55425 | 0,14 | 47,28 |
|  |  |  |  |  |  |  |  |  |  |  |  |  |  |  |  |  | High | DYNVDMVPK | 3 |  | 0,0000 | 2,79 | 2 | 1080,50286 | -0,20 | 44,81 |
|  |  |  |  |  |  |  |  |  |  |  |  |  |  |  |  |  | High | VPATDmEALK | 2 | M6(Oxidation) | 0,0000 | 2,76 | 2 | 1090,54521 | 0,24 | 41,50 |
|  |  |  |  |  |  |  |  |  |  |  |  |  |  |  |  |  | High | VLIHTDVTK | 6 |  | 0,0000 | 2,54 | 2 | 1025,59892 | -0,08 | 37,71 |
|  |  |  |  |  |  |  |  |  |  |  |  |  |  |  |  |  | High | FMmANGTLVR | 6 | M3(Oxidation) | 0,0000 | 2,50 | 2 | 1155,56450 | -0,42 | 42,08 |
|  |  |  |  |  |  |  |  |  |  |  |  |  |  |  |  |  | High | SPLMGLFEK | 3 |  | 0,0000 | 2,30 | 2 | 1021,53844 | -0,30 | 49,60 |
|  |  |  |  |  |  |  |  |  |  |  |  |  |  |  |  |  | High | DYNVDmVPK | 1 | M6(Oxidation) | 0,0000 | 2,29 | 2 | 1096,49700 | -0,90 | 45,29 |
|  |  |  |  |  |  |  |  |  |  |  |  |  |  |  |  |  | High | LYAESLAR | 2 |  | 0,0000 | 2,19 | 2 | 922,49901 | -0,30 | 40,31 |
|  |  |  |  |  |  |  |  |  |  |  |  |  |  |  |  |  | High | VPATDMEALK | 2 |  | 0,0000 | 2,05 | 2 | 1074,54985 | -0,18 | 42,22 |
|  |  |  |  |  |  |  |  |  |  |  |  |  |  |  |  |  | Medium | THEGMDLR | 1 |  | 0,0000 | 1,40 | 3 | 958,44120 | 0,06 | 36,33 |
| **3311** | M5VPR4 | enolase | Uncharacterized protein OS=Prunus persica GN=PRUPE_ppa005779mg PE=3 SV=1 - [M5VPR4_PRUPE] | 99% to GI:645272258 Prunus mume, E value 0 | Prunus mume | Cytoplasm | 02.01-Energy/Glycolysis | 110,87 | 38,06 | 3 | 15 | 16 | 61 | 444 | 47,7 | 5,82 | High | KYGQDATNVGDEGGFAPNIQENK | 2 |  | 0,0000 | 5,86 | 3 | 2452,13871 | 0,44 | 42,69 |
|  |  |  |  |  |  |  |  |  |  |  |  |  |  |  |  |  | High | IEEELGAEAVYAGAK | 2 |  | 0,0000 | 4,71 | 2 | 1549,77568 | 0,76 | 45,11 |
|  |  |  |  |  |  |  |  |  |  |  |  |  |  |  |  |  | High | YGQDATNVGDEGGFAPNIQENK | 3 |  | 0,0000 | 4,63 | 2 | 2324,04766 | 2,15 | 45,02 |
|  |  |  |  |  |  |  |  |  |  |  |  |  |  |  |  |  | High | VNQIGSVTESIEAVR | 3 |  | 0,0000 | 3,85 | 2 | 1601,85100 | 1,00 | 46,13 |
|  |  |  |  |  |  |  |  |  |  |  |  |  |  |  |  |  | High | LAmQEFmILPVGASSFK | 2 | M3(Oxidation); M7(Oxidation) | 0,0000 | 3,58 | 2 | 1900,95745 | 1,37 | 49,95 |
|  |  |  |  |  |  |  |  |  |  |  |  |  |  |  |  |  | High | KAGWGVMASHR | 1 |  | 0,0000 | 3,56 | 3 | 1199,61005 | -0,20 | 36,97 |
|  |  |  |  |  |  |  |  |  |  |  |  |  |  |  |  |  | High | AVNNVNSIIGPALIGK | 5 |  | 0,0000 | 3,48 | 2 | 1579,91802 | 0,83 | 49,52 |
|  |  |  |  |  |  |  |  |  |  |  |  |  |  |  |  |  | High | AGWGVMASHR | 4 |  | 0,0000 | 3,10 | 3 | 1071,51514 | -0,17 | 39,80 |
|  |  |  |  |  |  |  |  |  |  |  |  |  |  |  |  |  | High | LAmQEFMILPVGASSFK | 4 | M3(Oxidation) | 0,0000 | 2,81 | 2 | 1884,96111 | 0,63 | 51,56 |
|  |  |  |  |  |  |  |  |  |  |  |  |  |  |  |  |  | High | ScNALLLK | 3 | C2(Carbamidomethyl) | 0,0000 | 2,60 | 2 | 918,50743 | -0,35 | 43,18 |
|  |  |  |  |  |  |  |  |  |  |  |  |  |  |  |  |  | High | MGVEVYHHLK | 6 |  | 0,0000 | 2,51 | 2 | 1212,61968 | 0,20 | 37,93 |
|  |  |  |  |  |  |  |  |  |  |  |  |  |  |  |  |  | High | AGWGVmASHR | 5 | M6(Oxidation) | 0,0000 | 2,02 | 2 | 1087,50981 | -0,39 | 37,47 |
|  |  |  |  |  |  |  |  |  |  |  |  |  |  |  |  |  | High | FRVPVEPY | 3 |  | 0,0000 | 2,00 | 2 | 1006,53533 | -0,35 | 46,93 |
|  |  |  |  |  |  |  |  |  |  |  |  |  |  |  |  |  | Medium | NLVLPVPAFNVInGGSHAGNK | 5 | N13(Deamidated) | 0,0000 | 1,98 | 3 | 2119,13559 | 2,84 | 50,44 |
|  |  |  |  |  |  |  |  |  |  |  |  |  |  |  |  |  | Medium | mGVEVYHHLK | 2 | M1(Oxidation) | 0,0000 | 1,90 | 2 | 1228,61345 | -0,73 | 36,55 |
|  |  |  |  |  |  |  |  |  |  |  |  |  |  |  |  |  | Medium | KIPLYK | 2 |  | 0,0000 | 1,81 | 2 | 761,49205 | 0,05 | 38,64 |
|  |  |  |  |  |  |  |  |  |  |  |  |  |  |  |  |  | Medium | YNQLLR | 2 |  | 0,0000 | 1,78 | 2 | 806,45195 | 0,03 | 40,97 |
|  |  |  |  |  |  |  |  |  |  |  |  |  |  |  |  |  | Medium | KAGWGVmASHR | 2 | M7(Oxidation) | 0,0000 | 1,78 | 3 | 1215,60459 | -0,51 | 34,94 |
|  |  |  |  |  |  |  |  |  |  |  |  |  |  |  |  |  | Medium | EGLELLK | 3 |  | 0,0470 | 1,42 | 2 | 801,47154 | -0,18 | 45,46 |
|  |  |  |  |  |  |  |  |  |  |  |  |  |  |  |  |  | Medium | QIFDSR | 2 |  | 0,0000 | 1,12 | 2 | 765,38890 | -0,14 | 39,59 |
| **3314** | M5XC03 | protein CLP1 homolog | Protein CLP1 homolog OS=Prunus persica GN=PRUPE_ppa006062mg PE=3 SV=1 - [M5XC03_PRUPE] |  | Prunus mume | Chloroplast | 04.22-Transcription/mRNA processing | 16,78 | 18,88 | 2 | 7 | 7 | 10 | 429 | 46,9 | 5,95 | High | SALPIGAEPAADPTR | 2 |  | 0,0000 | 2,50 | 2 | 1465,76518 | 0,38 | 44,60 |
|  |  |  |  |  |  |  |  |  |  |  |  |  |  |  |  |  | High | IEVGNDAPLK | 1 |  | 0,0000 | 2,32 | 2 | 1055,57268 | -0,50 | 41,08 |
|  |  |  |  |  |  |  |  |  |  |  |  |  |  |  |  |  | High | TITYLAPSAGDLPSK | 1 |  | 0,0000 | 2,32 | 2 | 1533,81694 | 0,64 | 45,60 |
|  |  |  |  |  |  |  |  |  |  |  |  |  |  |  |  |  | High | ELAQVIER | 2 |  | 0,0000 | 2,05 | 2 | 957,53630 | -0,12 | 42,79 |
|  |  |  |  |  |  |  |  |  |  |  |  |  |  |  |  |  | Medium | ASPPEDSNSSQPQGPR | 2 |  | 0,0000 | 1,75 | 2 | 1653,74773 | 0,81 | 36,64 |
|  |  |  |  |  |  |  |  |  |  |  |  |  |  |  |  |  | Medium | VIVVGPTDSGK | 1 |  | 0,0000 | 1,71 | 2 | 1071,60381 | -0,66 | 40,67 |
|  |  |  |  |  |  |  |  |  |  |  |  |  |  |  |  |  | Medium | LcSMLR | 1 | C2(Carbamidomethyl) | 0,1037 | 1,21 | 2 | 779,38994 | -0,44 | 40,49 |
| **3316** | M5W621 | Aminoacylase | Uncharacterized protein OS=Prunus persica GN=PRUPE_ppa005858mg PE=4 SV=1 - [M5W621_PRUPE] | 99% to GI:645260436 Prunus mume, E value 0 | Prunus mume | Chloroplast | 01.01-Metabolism/Amino acid | 48,43 | 18,18 | 1 | 8 | 8 | 21 | 440 | 48,7 | 5,74 | High | LYDNTATENLFK | 3 |  | 0,0000 | 3,48 | 2 | 1428,70085 | 0,19 | 47,44 |
|  |  |  |  |  |  |  |  |  |  |  |  |  |  |  |  |  | High | LGKPEIFPASTDAR | 2 |  | 0,0000 | 3,27 | 3 | 1501,80063 | -0,25 | 42,69 |
|  |  |  |  |  |  |  |  |  |  |  |  |  |  |  |  |  | High | VPPTADQESLER | 3 |  | 0,0000 | 3,10 | 2 | 1341,66411 | -0,33 | 39,81 |
|  |  |  |  |  |  |  |  |  |  |  |  |  |  |  |  |  | High | ASQFDLVK | 2 |  | 0,0000 | 2,73 | 2 | 907,48802 | -0,42 | 43,09 |
|  |  |  |  |  |  |  |  |  |  |  |  |  |  |  |  |  | High | AYASYVDHGR | 4 |  | 0,0000 | 2,62 | 2 | 1138,52751 | -0,09 | 37,80 |
|  |  |  |  |  |  |  |  |  |  |  |  |  |  |  |  |  | High | IAEEWAPSSR | 3 |  | 0,0000 | 2,55 | 2 | 1145,55864 | 0,02 | 43,70 |
|  |  |  |  |  |  |  |  |  |  |  |  |  |  |  |  |  | High | FAESDVFK | 3 |  | 0,0000 | 2,06 | 2 | 942,45622 | -0,58 | 43,85 |
|  |  |  |  |  |  |  |  |  |  |  |  |  |  |  |  |  | Medium | TFYAER | 1 |  | 0,0000 | 1,08 | 2 | 786,37834 | 0,31 | 38,48 |
| **3518** | M5XYF8 | T-complex protein 1 subunit beta | Uncharacterized protein OS=Prunus persica GN=PRUPE_ppa004143mg PE=3 SV=1 - [M5XYF8_PRUPE] | 99% to GI:645228826 Prunus mume, E value 0 | Prunus mume | Cytoplasm | 04.22-Transcription/mRNA processing | 80,98 | 31,88 | 1 | 17 | 17 | 37 | 527 | 57,1 | 6,05 | High | VDEIITcAPR | 2 | C7(Carbamidomethyl) | 0,0000 | 4,14 | 2 | 1173,59355 | 0,22 | 42,04 |
|  |  |  |  |  |  |  |  |  |  |  |  |  |  |  |  |  | High | GASHHVLDEAER | 5 |  | 0,0000 | 4,00 | 3 | 1320,62885 | -0,24 | 35,71 |
|  |  |  |  |  |  |  |  |  |  |  |  |  |  |  |  |  | High | ILVANTAMDTDK | 2 |  | 0,0000 | 3,50 | 2 | 1291,65691 | 0,47 | 43,00 |
|  |  |  |  |  |  |  |  |  |  |  |  |  |  |  |  |  | High | GHAVTVTNDGATILK | 2 |  | 0,0000 | 3,39 | 2 | 1496,80681 | 0,01 | 40,44 |
|  |  |  |  |  |  |  |  |  |  |  |  |  |  |  |  |  | High | GSTNLEAIQIIK | 2 |  | 0,0000 | 2,99 | 2 | 1286,73186 | 0,28 | 47,53 |
|  |  |  |  |  |  |  |  |  |  |  |  |  |  |  |  |  | High | SLHIDNAAAK | 2 |  | 0,0000 | 2,84 | 3 | 1039,55298 | -0,14 | 37,32 |
|  |  |  |  |  |  |  |  |  |  |  |  |  |  |  |  |  | High | IIAHGINcFVNR | 1 | C8(Carbamidomethyl) | 0,0000 | 2,81 | 3 | 1413,74091 | -0,79 | 41,87 |
|  |  |  |  |  |  |  |  |  |  |  |  |  |  |  |  |  | High | EGcTAGIDVISGAVGDMAER | 1 | C3(Carbamidomethyl) | 0,0000 | 2,55 | 2 | 2007,91252 | 0,69 | 49,77 |
|  |  |  |  |  |  |  |  |  |  |  |  |  |  |  |  |  | High | IAMTTLSSK | 2 |  | 0,0000 | 2,29 | 2 | 951,51738 | -0,64 | 40,67 |
|  |  |  |  |  |  |  |  |  |  |  |  |  |  |  |  |  | High | IFKDEASEEK | 3 |  | 0,0000 | 2,24 | 2 | 1195,58464 | 0,39 | 35,45 |
|  |  |  |  |  |  |  |  |  |  |  |  |  |  |  |  |  | Medium | IFKDEASEEKGER | 4 |  | 0,0000 | 2,21 | 3 | 1537,74918 | -0,12 | 34,75 |
|  |  |  |  |  |  |  |  |  |  |  |  |  |  |  |  |  | Medium | KSHAIEAFSR | 1 |  | 0,0000 | 2,20 | 3 | 1145,60776 | 1,34 | 35,97 |
|  |  |  |  |  |  |  |  |  |  |  |  |  |  |  |  |  | High | VLVDISK | 3 |  | 0,0000 | 2,16 | 2 | 773,47661 | -0,21 | 42,84 |
|  |  |  |  |  |  |  |  |  |  |  |  |  |  |  |  |  | High | DSFLDEGFILDK | 1 |  | 0,0000 | 2,16 | 2 | 1398,67729 | -1,09 | 52,68 |
|  |  |  |  |  |  |  |  |  |  |  |  |  |  |  |  |  | High | SHAIEAFSR | 4 |  | 0,0000 | 2,05 | 2 | 1017,51085 | -0,40 | 38,85 |
|  |  |  |  |  |  |  |  |  |  |  |  |  |  |  |  |  | Medium | IGLGQPK | 1 |  | 0,0000 | 1,91 | 2 | 712,43523 | -0,02 | 38,21 |
|  |  |  |  |  |  |  |  |  |  |  |  |  |  |  |  |  | Medium | GISESFK | 1 |  | 0,0813 | 1,13 | 2 | 767,39385 | 0,54 | 39,21 |
|  | M5WMW6 | Pyruvate decarboxylase | Uncharacterized protein OS=Prunus persica GN=PRUPE_ppa003458mg PE=3 SV=1 - [M5WMW6_PRUPE] | 99% to GI:645237438 Prunus mume, E value 0 | Prunus mume | Cytoplasm | 11.05-Disease/Defense/Stress responses | 65,52 | 17,98 | 1 | 9 | 11 | 38 | 573 | 62,0 | 6,13 | High | VRTEEDLTEAIAK | 2 |  | 0,0000 | 4,31 | 2 | 1474,77568 | 0,57 | 41,41 |
|  |  |  |  |  |  |  |  |  |  |  |  |  |  |  |  |  | High | mQGFRPANSTGTLGR | 3 | N-Term(Acetyl) | 0,0000 | 4,10 | 2 | 1634,80840 | 0,95 | 45,48 |
|  |  |  |  |  |  |  |  |  |  |  |  |  |  |  |  |  | High | TEEDLTEAIAK | 2 |  | 0,0000 | 3,28 | 2 | 1219,60515 | -0,12 | 43,11 |
|  |  |  |  |  |  |  |  |  |  |  |  |  |  |  |  |  | High | AVKPVIIGGPK | 5 |  | 0,0000 | 2,55 | 3 | 1078,69837 | 0,01 | 38,72 |
|  |  |  |  |  |  |  |  |  |  |  |  |  |  |  |  |  | High | VSAANSRPPNPQ | 4 |  | 0,0000 | 2,49 | 2 | 1237,62895 | 0,42 | 36,26 |
|  |  |  |  |  |  |  |  |  |  |  |  |  |  |  |  |  | High | DPVPFFLAPK | 3 |  | 0,0000 | 2,33 | 2 | 1130,62444 | -0,07 | 52,10 |
|  |  |  |  |  |  |  |  |  |  |  |  |  |  |  |  |  | High | ELLEWGSR | 7 |  | 0,0000 | 2,24 | 2 | 989,50493 | -0,19 | 48,42 |
|  |  |  |  |  |  |  |  |  |  |  |  |  |  |  |  |  | High | VNVLFK | 3 |  | 0,0000 | 2,22 | 2 | 719,44518 | 0,15 | 44,28 |
|  |  |  |  |  |  |  |  |  |  |  |  |  |  |  |  |  | High | mQGFRPAnSTGTLGR | 2 | N-Term(Acetyl); N8(Deamidated) | 0,0000 | 2,06 | 2 | 1635,78850 | -1,44 | 47,33 |
|  |  |  |  |  |  |  |  |  |  |  |  |  |  |  |  |  | Medium | AIIVEPNR | 3 |  | 0,0000 | 1,83 | 2 | 911,53063 | -0,35 | 41,94 |
|  |  |  |  |  |  |  |  |  |  |  |  |  |  |  |  |  | Medium | IFVPPGIPLK | 3 |  | 0,0000 | 1,62 | 2 | 1080,68169 | 0,04 | 50,75 |
|  |  |  |  |  |  |  |  |  |  |  |  |  |  |  |  |  | Medium | nSTAVENYHR | 1 | N1(Deamidated) | 0,0224 | 1,31 | 3 | 1191,53885 | -0,05 | 37,00 |
| **3616** | M5VWE8 | Malic enzyme | Malic enzyme OS=Prunus persica GN=PRUPE_ppa003142mg PE=3 SV=1 - [M5VWE8_PRUPE] |  | Prunus persica | Chloroplast | 02.30-Energy/Photosynthesis | 132,95 | 34,63 | 9 | 19 | 24 | 89 | 592 | 65,6 | 5,99 | High | YAEScMYSPVYR | 5 | C5(Carbamidomethyl) | 0,0000 | 3,60 | 2 | 1525,64653 | 0,97 | 43,19 |
|  |  |  |  |  |  |  |  |  |  |  |  |  |  |  |  |  | High | TVQVIVVTDGER | 2 |  | 0,0000 | 3,54 | 2 | 1315,72173 | 0,04 | 43,87 |
|  |  |  |  |  |  |  |  |  |  |  |  |  |  |  |  |  | High | AYELGLATR | 3 |  | 0,0000 | 3,24 | 2 | 993,53606 | -0,36 | 45,53 |
|  |  |  |  |  |  |  |  |  |  |  |  |  |  |  |  |  | High | AIFASGSPFDPVEYNGK | 1 |  | 0,0000 | 3,11 | 2 | 1798,86614 | 0,79 | 49,30 |
|  |  |  |  |  |  |  |  |  |  |  |  |  |  |  |  |  | High | ASVLDFDPK | 6 |  | 0,0000 | 3,07 | 2 | 991,50902 | -0,53 | 45,95 |
|  |  |  |  |  |  |  |  |  |  |  |  |  |  |  |  |  | High | YAEScmYSPVYR | 4 | C5(Carbamidomethyl); M6(Oxidation) | 0,0000 | 2,81 | 2 | 1541,64141 | 0,94 | 43,32 |
|  |  |  |  |  |  |  |  |  |  |  |  |  |  |  |  |  | High | YMALTELQER | 4 |  | 0,0000 | 2,78 | 2 | 1253,62053 | 0,82 | 45,31 |
|  |  |  |  |  |  |  |  |  |  |  |  |  |  |  |  |  | High | GLLPPATcSQQLQEK | 4 | C8(Carbamidomethyl) | 0,0000 | 2,44 | 2 | 1669,85967 | 1,08 | 44,14 |
|  |  |  |  |  |  |  |  |  |  |  |  |  |  |  |  |  | Medium | AIKPTVLIGSSGVGR | 1 |  | 0,0000 | 2,43 | 3 | 1454,86841 | -0,42 | 42,77 |
|  |  |  |  |  |  |  |  |  |  |  |  |  |  |  |  |  | High | YmALTELQER | 4 | M2(Oxidation) | 0,0000 | 2,41 | 2 | 1269,61540 | 0,78 | 44,53 |
|  |  |  |  |  |  |  |  |  |  |  |  |  |  |  |  |  | High | mESTFNELGDK | 5 | N-Term(Acetyl) | 0,0000 | 2,37 | 2 | 1312,57292 | 0,23 | 51,95 |
|  |  |  |  |  |  |  |  |  |  |  |  |  |  |  |  |  | High | IWLVDSK | 2 |  | 0,0000 | 2,27 | 2 | 860,48747 | -0,23 | 47,26 |
|  |  |  |  |  |  |  |  |  |  |  |  |  |  |  |  |  | High | DSLQQFK | 3 |  | 0,0000 | 2,15 | 2 | 865,44145 | 0,01 | 42,56 |
|  |  |  |  |  |  |  |  |  |  |  |  |  |  |  |  |  | High | KDSLQQFK | 5 |  | 0,0000 | 2,09 | 2 | 993,53612 | -0,29 | 36,72 |
|  |  |  |  |  |  |  |  |  |  |  |  |  |  |  |  |  | Medium | LmNNIR | 3 | M2(Oxidation) | 0,0000 | 1,87 | 2 | 776,40825 | -0,16 | 36,24 |
|  |  |  |  |  |  |  |  |  |  |  |  |  |  |  |  |  | Medium | GLIVSSR | 5 |  | 0,0000 | 1,78 | 2 | 731,44121 | 0,22 | 41,35 |
|  |  |  |  |  |  |  |  |  |  |  |  |  |  |  |  |  | Medium | LMNNIR | 2 |  | 0,0000 | 1,71 | 2 | 760,41210 | -1,80 | 37,73 |
|  |  |  |  |  |  |  |  |  |  |  |  |  |  |  |  |  | Medium | KIWLVDSK | 3 |  | 0,0000 | 1,69 | 2 | 988,58226 | -0,38 | 42,22 |
|  |  |  |  |  |  |  |  |  |  |  |  |  |  |  |  |  | Medium | FGSIFR | 5 |  | 0,0000 | 1,69 | 2 | 726,39348 | 0,15 | 46,56 |
|  |  |  |  |  |  |  |  |  |  |  |  |  |  |  |  |  | Medium | KLmNNIR | 3 | M3(Oxidation) | 0,0000 | 1,52 | 2 | 904,50249 | -0,95 | 35,39 |
|  |  |  |  |  |  |  |  |  |  |  |  |  |  |  |  |  | Medium | LVGGSLSEHK | 5 |  | 0,0000 | 1,48 | 2 | 1026,55669 | -1,16 | 35,89 |
|  |  |  |  |  |  |  |  |  |  |  |  |  |  |  |  |  | Medium | GLAFTEK | 2 |  | 0,0000 | 1,46 | 2 | 765,41442 | 0,32 | 41,63 |
|  |  |  |  |  |  |  |  |  |  |  |  |  |  |  |  |  | Medium | AIFASGSPFDPVEYnGK | 1 | N15(Deamidated) | 0,0000 | 1,37 | 2 | 1799,84770 | -0,57 | 50,22 |
|  |  |  |  |  |  |  |  |  |  |  |  |  |  |  |  |  | Medium | LPRPENLVK | 2 |  | 0,0000 | 1,27 | 2 | 1065,64153 | -0,03 | 39,98 |
|  |  |  |  |  |  |  |  |  |  |  |  |  |  |  |  |  | Medium | VLEVLK | 4 |  | 0,0000 | 1,13 | 2 | 700,46031 | -0,11 | 42,10 |
|  |  |  |  |  |  |  |  |  |  |  |  |  |  |  |  |  | Medium | QYQVPLQK | 3 |  | 0,0000 | 0,91 | 2 | 1003,55651 | -0,62 | 40,12 |
| **4013** | M5WCS1 | Ras-related protein | Uncharacterized protein OS=Prunus persica GN=PRUPE_ppa011297mg PE=3 SV=1 - [M5WCS1_PRUPE] |  | Prunus persica | Chloroplast | 08.99-Intracellular traffic/Others | 15,53 | 28,24 | 17 | 6 | 6 | 10 | 216 | 23,9 | 6,55 | High | SVSEDDGQTLAER | 2 |  | 0,0000 | 3,12 | 2 | 1406,64031 | 0,60 | 35,38 |
|  |  |  |  |  |  |  |  |  |  |  |  |  |  |  |  |  | High | AQIWDTAGQER | 1 |  | 0,0000 | 2,31 | 2 | 1274,61211 | -0,27 | 39,39 |
|  |  |  |  |  |  |  |  |  |  |  |  |  |  |  |  |  | Medium | NEFcLESK | 1 | C4(Carbamidomethyl) | 0,0000 | 1,93 | 2 | 1026,45598 | -0,13 | 38,38 |
|  |  |  |  |  |  |  |  |  |  |  |  |  |  |  |  |  | Medium | IVLIGDSGVGK | 3 |  | 0,0052 | 1,92 | 2 | 1057,62493 | -0,31 | 42,74 |
|  |  |  |  |  |  |  |  |  |  |  |  |  |  |  |  |  | Medium | AITSAYYR | 2 |  | 0,0000 | 1,65 | 2 | 944,48369 | 0,07 | 36,62 |
|  |  |  |  |  |  |  |  |  |  |  |  |  |  |  |  |  | Medium | STIGVEFATR | 1 |  | 0,0000 | 1,43 | 2 | 1080,56853 | 0,07 | 40,71 |
| **4208** | M1QDP5 | S-adenosylmethionine synthase | S-adenosylmethionine synthase OS=Prunus persica GN=PRUPE_ppa006841mg PE=2 SV=1 - [M1QDP5_PRUPE] |  | Prunus persica | Cytoplasm | 01.01-Metabolism/Amino Acid | 18,90 | 17,30 | 3 | 4 | 7 | 14 | 393 | 42,9 | 6,05 | High | IPDKEILK | 4 |  | 0,0000 | 2,56 | 2 | 955,58190 | -0,43 | 37,43 |
|  |  |  |  |  |  |  |  |  |  |  |  |  |  |  |  |  | High | TIGFVSDDVGLDADNcK | 1 | C16(Carbamidomethyl) | 0,0000 | 2,54 | 2 | 1825,82915 | 0,98 | 45,78 |
|  |  |  |  |  |  |  |  |  |  |  |  |  |  |  |  |  | High | TAAYGHFGR | 3 |  | 0,0000 | 2,16 | 2 | 979,47405 | -0,43 | 35,07 |
|  |  |  |  |  |  |  |  |  |  |  |  |  |  |  |  |  | High | SIVASGLAR | 2 |  | 0,0000 | 2,10 | 2 | 873,51488 | -0,47 | 38,50 |
|  |  |  |  |  |  |  |  |  |  |  |  |  |  |  |  |  | Medium | SGAYIVR | 1 |  | 0,0000 | 1,91 | 2 | 765,42522 | -0,23 | 36,64 |
|  |  |  |  |  |  |  |  |  |  |  |  |  |  |  |  |  | Medium | EHVIKPVIPEK | 1 |  | 0,0000 | 1,72 | 2 | 1288,76335 | 0,73 | 35,97 |
|  |  |  |  |  |  |  |  |  |  |  |  |  |  |  |  |  | Medium | GAMVPIR | 2 |  | 0,0000 | 1,50 | 2 | 743,42339 | 0,10 | 39,44 |
| **4209** | M1QDQ3 | methylthioribose kinase | 5-methylthioribose kinase OS=Prunus persica GN=mtrk PE=2 SV=1 - [M1QDQ3_PRUPE] |  | Prunus mume | Chloroplast | 01.01-Metabolism/Amino Acid | 10,08 | 11,51 | 3 | 6 | 6 | 7 | 417 | 47,3 | 6,14 | High | cIGESWPMTK | 1 | C1(Carbamidomethyl) | 0,0000 | 2,62 | 2 | 1208,54375 | -0,13 | 42,63 |
|  |  |  |  |  |  |  |  |  |  |  |  |  |  |  |  |  | High | TMSLIGMR | 1 |  | 0,0000 | 2,21 | 2 | 908,46916 | -0,13 | 43,23 |
|  |  |  |  |  |  |  |  |  |  |  |  |  |  |  |  |  | Medium | AYFEALALR | 1 |  | 0,0000 | 1,86 | 2 | 1053,57231 | -0,46 | 46,13 |
|  |  |  |  |  |  |  |  |  |  |  |  |  |  |  |  |  | Medium | VLIEYIK | 2 |  | 0,0000 | 1,67 | 2 | 877,53960 | 0,27 | 44,46 |
|  |  |  |  |  |  |  |  |  |  |  |  |  |  |  |  |  | Medium | FDNLTIK | 1 |  | 0,0000 | 1,43 | 2 | 850,46666 | -0,32 | 40,62 |
|  |  |  |  |  |  |  |  |  |  |  |  |  |  |  |  |  | Medium | LEVAELK | 1 |  | 0,0000 | 1,38 | 2 | 801,47215 | 0,58 | 39,96 |
| **4211** | M5XCU1 | Delta-aminolevulinic acid dehydratase | Delta-aminolevulinic acid dehydratase OS=Prunus persica GN=PRUPE_ppa006219mg PE=3 SV=1 - [M5XCU1_PRUPE] |  | Prunus persica | Chloroplast | 20.99-Secondary metabolism/Others | 15,46 | 11,37 | 1 | 4 | 4 | 10 | 422 | 45,9 | 7,09 | High | NSTGDEAYNDNGLVPR | 1 |  | 0,0000 | 2,92 | 2 | 1721,77410 | 0,87 | 40,62 |
|  |  |  |  |  |  |  |  |  |  |  |  |  |  |  |  |  | High | NSTGDEAYnDNGLVPR | 4 | N9(Deamidated) | 0,0000 | 2,82 | 2 | 1722,75591 | -0,41 | 41,54 |
|  |  |  |  |  |  |  |  |  |  |  |  |  |  |  |  |  | High | TYQMNPANYR | 2 |  | 0,0000 | 2,24 | 2 | 1257,56792 | -0,15 | 39,11 |
|  |  |  |  |  |  |  |  |  |  |  |  |  |  |  |  |  | High | AGADVVSPSDMMDGR | 1 |  | 0,0000 | 2,21 | 2 | 1507,65410 | 1,62 | 43,01 |
|  |  |  |  |  |  |  |  |  |  |  |  |  |  |  |  |  | Medium | EALIEAR | 2 |  | 0,0000 | 1,61 | 2 | 801,44676 | 0,28 | 38,81 |
| **4214** | M5W463 | Protein disulfide isomerase | Uncharacterized protein OS=Prunus persica GN=PRUPE_ppa008282mg PE=3 SV=1 - [M5W463_PRUPE] | 92% to GI:645221668 Prunus mume, E value 0 | Prunus mume | Chloroplast | 06.01-Protein destination and storage/Folding and stability | 14,91 | 10,95 | 1 | 5 | 5 | 10 | 338 | 37,2 | 8,72 | High | TAEALAEFVNK | 2 |  | 0,0000 | 4,02 | 2 | 1192,62102 | 0,11 | 44,50 |
|  |  |  |  |  |  |  |  |  |  |  |  |  |  |  |  |  | Medium | ADEFTLK | 2 |  | 0,0000 | 2,00 | 2 | 823,41966 | 0,02 | 41,32 |
|  |  |  |  |  |  |  |  |  |  |  |  |  |  |  |  |  | Medium | ADEFTLKK | 3 |  | 0,0000 | 1,97 | 2 | 951,51384 | -0,82 | 36,28 |
|  |  |  |  |  |  |  |  |  |  |  |  |  |  |  |  |  | Medium | NILYTFASSS | 1 |  | 0,0000 | 1,86 | 2 | 1102,54119 | -0,32 | 49,84 |
|  |  |  |  |  |  |  |  |  |  |  |  |  |  |  |  |  | Medium | SLAPTYEK | 2 |  | 0,0000 | 1,43 | 2 | 908,47228 | -0,14 | 36,25 |
| **4215** | M5W9E0 | 1-aminocyclopropane-1-carboxylate deaminase | Uncharacterized protein OS=Prunus persica GN=PRUPE_ppa006016mg PE=4 SV=1 - [M5W9E0_PRUPE] | 96% to GI:645270273 Prunus mume, E value 0 | Prunus mume | Chloroplast | 11.05-Disease/Defense/Stress responses | 37,18 | 20,37 | 2 | 9 | 9 | 19 | 432 | 47,5 | 7,58 | High | EIEEQLQSGTDK | 2 |  | 0,0000 | 3,23 | 2 | 1376,65434 | 0,22 | 37,62 |
|  |  |  |  |  |  |  |  |  |  |  |  |  |  |  |  |  | High | DIVNVQNAR | 2 |  | 0,0000 | 2,85 | 2 | 1028,54900 | 0,60 | 38,61 |
|  |  |  |  |  |  |  |  |  |  |  |  |  |  |  |  |  | High | VEQmAPSLGNWR | 1 | M4(Oxidation) | 0,0000 | 2,79 | 2 | 1403,67449 | 0,57 | 42,30 |
|  |  |  |  |  |  |  |  |  |  |  |  |  |  |  |  |  | High | RDDLSGMQLSGNK | 1 |  | 0,0000 | 2,76 | 3 | 1420,68427 | -0,50 | 38,71 |
|  |  |  |  |  |  |  |  |  |  |  |  |  |  |  |  |  | High | VEQMAPSLGNWR | 2 |  | 0,0000 | 2,68 | 2 | 1387,67949 | 0,52 | 44,01 |
|  |  |  |  |  |  |  |  |  |  |  |  |  |  |  |  |  | High | DDLSGmQLSGNK | 1 | M6(Oxidation) | 0,0000 | 2,35 | 2 | 1280,57854 | -0,19 | 37,74 |
|  |  |  |  |  |  |  |  |  |  |  |  |  |  |  |  |  | High | MDVQESVPPSDGIGK | 3 |  | 0,0000 | 2,18 | 2 | 1558,74419 | 1,51 | 40,33 |
|  |  |  |  |  |  |  |  |  |  |  |  |  |  |  |  |  | Medium | AVGLDFLTK | 2 |  | 0,0000 | 1,96 | 2 | 963,55083 | -0,19 | 47,85 |
|  |  |  |  |  |  |  |  |  |  |  |  |  |  |  |  |  | Medium | RDDLSGmQLSGNK | 1 | M7(Oxidation) | 0,0000 | 1,95 | 3 | 1436,67954 | -0,25 | 35,08 |
|  |  |  |  |  |  |  |  |  |  |  |  |  |  |  |  |  | Medium | AAYGMLK | 2 |  | 0,0000 | 1,60 | 2 | 753,39647 | 0,08 | 38,73 |
|  |  |  |  |  |  |  |  |  |  |  |  |  |  |  |  |  | Medium | FGSGDLTNLLK | 1 |  | 0,0165 | 1,19 | 2 | 1164,62529 | -0,58 | 47,70 |
|  |  |  |  |  |  |  |  |  |  |  |  |  |  |  |  |  | Medium | mDVQESVPPSDGIGK | 1 | M1(Oxidation) | 0,0000 | 0,98 | 2 | 1574,73564 | -0,71 | 40,57 |
| **4313** | M1QDP5 | S-adenosylmethionine synthase | S-adenosylmethionine synthase OS=Prunus persica GN=PRUPE_ppa006841mg PE=2 SV=1 - [M1QDP5_PRUPE] |  | Prunus mume | Cytoplasm | 01.01-Metabolism/Amino Acid | 43,16 | 27,74 | 7 | 10 | 10 | 21 | 393 | 42,8817 | 6,049316 | High | TIGFVSDDVGLDADNcK | 1 | C16(Carbamidomethyl) | 0,0000 | 4,69 | 2 | 1825,83025 | 1,58 | 45,73 |
|  |  |  |  |  |  |  |  |  |  |  |  |  |  |  |  |  | High | FVIGGPHGDAGLTGR | 2 |  | 0,0000 | 4,31 | 2 | 1453,75603 | 0,90 | 39,90 |
|  |  |  |  |  |  |  |  |  |  |  |  |  |  |  |  |  | High | TQVTVEYYNDK | 2 |  | 0,0000 | 3,36 | 2 | 1359,64287 | 0,12 | 38,78 |
|  |  |  |  |  |  |  |  |  |  |  |  |  |  |  |  |  | High | IPDKEILK | 4 |  | 0,0000 | 3,05 | 2 | 955,58171 | -0,63 | 36,47 |
|  |  |  |  |  |  |  |  |  |  |  |  |  |  |  |  |  | High | TIFHLNPSGR | 2 |  | 0,0000 | 2,59 | 2 | 1141,61101 | -0,26 | 39,33 |
|  |  |  |  |  |  |  |  |  |  |  |  |  |  |  |  |  | High | EHVIKPVIPEK | 4 |  | 0,0000 | 2,42 | 2 | 1288,76323 | 0,64 | 35,51 |
|  |  |  |  |  |  |  |  |  |  |  |  |  |  |  |  |  | High | SIVASGLAR | 2 |  | 0,0000 | 2,32 | 2 | 873,51476 | -0,61 | 39,30 |
|  |  |  |  |  |  |  |  |  |  |  |  |  |  |  |  |  | Medium | NGTcAWLRPDGK | 1 | C4(Carbamidomethyl) | 0,0000 | 2,19 | 3 | 1374,65750 | -0,63 | 38,46 |
|  |  |  |  |  |  |  |  |  |  |  |  |  |  |  |  |  | High | TAAYGHFGR | 2 |  | 0,0000 | 2,17 | 2 | 979,47405 | -0,43 | 34,73 |
|  |  |  |  |  |  |  |  |  |  |  |  |  |  |  |  |  | Medium | GAMVPIR | 1 |  | 0,0000 | 1,56 | 2 | 743,42284 | -0,64 | 38,24 |
| **4421** | M5XCZ6 | Adenosylhomocysteinase | Adenosylhomocysteinase OS=Prunus persica GN=PRUPE_ppa004934mg PE=3 SV=1 - [M5XCZ6_PRUPE] |  | Prunus mume | Chloroplast | 01.01-Metabolism/Amino acid | 12,28 | 12,16 | 2 | 6 | 6 | 8 | 485 | 53,3 | 6,19 | High | VSVVcGYGDVGK | 2 | C5(Carbamidomethyl) | 0,0000 | 2,15 | 2 | 1239,59807 | -4,67 | 40,14 |
|  |  |  |  |  |  |  |  |  |  |  |  |  |  |  |  |  | High | LVGVSEETTTGVK | 2 |  | 0,0000 | 2,00 | 2 | 1319,70622 | 0,66 | 38,83 |
|  |  |  |  |  |  |  |  |  |  |  |  |  |  |  |  |  | Medium | DMSQADFGR | 1 |  | 0,0000 | 1,79 | 2 | 1026,43083 | -0,14 | 39,81 |
|  |  |  |  |  |  |  |  |  |  |  |  |  |  |  |  |  | Medium | DSAAVFAWK | 1 |  | 0,0000 | 1,71 | 2 | 994,49938 | 0,07 | 46,51 |
|  |  |  |  |  |  |  |  |  |  |  |  |  |  |  |  |  | Medium | TEFGPSQPFK | 1 |  | 0,0000 | 1,53 | 2 | 1137,55803 | 0,42 | 41,05 |
|  |  |  |  |  |  |  |  |  |  |  |  |  |  |  |  |  | Medium | VYVLPK | 1 |  | 0,1346 | 0,90 | 2 | 718,45000 | 0,26 | 39,57 |
| **4711** | M5W2A2 | Receptor-like protein kinase | Uncharacterized protein OS=Prunus persica GN=PRUPE_ppa001265mg PE=4 SV=1 - [M5W2A2_PRUPE] | 99% to GI:645262841 Prunus mume, E value 0 | Prunus mume | Cell wall | 10.04.04-Signal transduction/Kinases | 5,42 | 1,27 | 1 | 2 | 2 | 3 | 867 | 95,0 | 6,01 | Medium | LDICIGAAR | 1 |  | 0,0054 | 1,84 | 2 | 931,50902 | 6,44 | 37,65 |
|  |  |  |  |  |  |  |  |  |  |  |  |  |  |  |  |  | Medium | qRLDICIGAAR | 1 | Q1(Deamidated) | 0,0109 | 1,81 | 2 | 1216,65715 | 8,56 | 46,67 |
|  |  |  |  |  |  |  |  |  |  |  |  |  |  |  |  |  | Medium | QRLDICIGAAR | 1 |  | 0,0000 | 1,78 | 2 | 1215,66521 | 2,05 | 45,94 |
| **4726** | M5XB32 | Prolyl endopeptidase | Uncharacterized protein OS=Prunus persica GN=PRUPE_ppa001738mg PE=4 SV=1 - [M5XB32_PRUPE] | 95% to GI:645228649 Prunus mume, E value 0 | Prunus mume | Chloroplast | 06.13-Protein destination and storage/Proteolysis | 11,90 | 8,55 | 2 | 8 | 8 | 10 | 772 | 87,1 | 6,43 | High | LVAYAEDTK | 1 |  | 0,0000 | 2,03 | 2 | 1009,52001 | -0,08 | 36,87 |
|  |  |  |  |  |  |  |  |  |  |  |  |  |  |  |  |  | Medium | LEDGLVVLTPR | 1 |  | 0,0000 | 1,96 | 2 | 1211,69890 | -0,48 | 45,85 |
|  |  |  |  |  |  |  |  |  |  |  |  |  |  |  |  |  | Medium | TPPSVYDYDMK | 1 |  | 0,0000 | 1,87 | 2 | 1315,59062 | 2,36 | 41,43 |
|  |  |  |  |  |  |  |  |  |  |  |  |  |  |  |  |  | Medium | MEMFGDVR | 1 |  | 0,0000 | 1,62 | 2 | 984,42772 | -0,10 | 43,64 |
|  |  |  |  |  |  |  |  |  |  |  |  |  |  |  |  |  | Medium | IKEDDISAPER | 3 |  | 0,0000 | 1,55 | 2 | 1272,64336 | 0,21 | 35,54 |
|  |  |  |  |  |  |  |  |  |  |  |  |  |  |  |  |  | Medium | TGISVLK | 1 |  | 0,0000 | 1,41 | 2 | 717,45067 | 0,17 | 39,26 |
|  |  |  |  |  |  |  |  |  |  |  |  |  |  |  |  |  | Medium | EDDISAPER | 1 |  | 0,0000 | 1,25 | 2 | 1031,46416 | 0,10 | 36,34 |
|  |  |  |  |  |  |  |  |  |  |  |  |  |  |  |  |  | Medium | FLFVGSESK | 1 |  | 0,0538 | 1,23 | 2 | 1013,53111 | 0,83 | 44,27 |
| **4805** | M5WQJ8 | neutral alpha-glucosidase | Uncharacterized protein OS=Prunus persica GN=PRUPE_ppa000927mg PE=4 SV=1 - [M5WQJ8_PRUPE] | 94% to GI:645265210 Prunus mume, E value 7e-112 | Prunus mume | Chloroplast | 01.05-Metabolism/Sugars and polysaccharides | 16,22 | 6,36 | 1 | 6 | 7 | 10 | 959 | 106,9 | 6,20 | High | SAGcTEEYNVTER | 2 | C4(Carbamidomethyl) | 0,0000 | 2,26 | 2 | 1515,64018 | 1,40 | 36,97 |
|  |  |  |  |  |  |  |  |  |  |  |  |  |  |  |  |  | High | IDSSIIAR | 1 |  | 0,0000 | 2,21 | 2 | 874,49919 | -0,12 | 39,55 |
|  |  |  |  |  |  |  |  |  |  |  |  |  |  |  |  |  | High | AGFIGSQR | 2 |  | 0,0000 | 2,05 | 2 | 835,44200 | -0,14 | 37,10 |
|  |  |  |  |  |  |  |  |  |  |  |  |  |  |  |  |  | Medium | GGDWFVK | 1 |  | 0,0000 | 1,81 | 2 | 808,39879 | -0,09 | 42,89 |
|  |  |  |  |  |  |  |  |  |  |  |  |  |  |  |  |  | Medium | FDVSQVYK | 2 |  | 0,0000 | 1,78 | 2 | 985,49901 | 0,06 | 42,38 |
|  |  |  |  |  |  |  |  |  |  |  |  |  |  |  |  |  | Medium | VVPWIGGR | 1 |  | 0,0714 | 1,04 | 2 | 883,51457 | -0,37 | 43,66 |
| **4828** | M5VSW8 | aconitate hydratase | Uncharacterized protein OS=Prunus persica GN=PRUPE_ppa001138mg PE=4 SV=1 - [M5VSW8_PRUPE] | 99% to GI:645273719 Prunus mume, E value 0 | Prunus mume | Cytoplasm | 02.10-Energy/TCA pathway | 22,81 | 13,59 | 1 | 8 | 13 | 19 | 898 | 98,2 | 6,33 | High | TGEDADTLGLTGEER | 1 |  | 0,0000 | 3,33 | 2 | 1563,71318 | -0,12 | 40,46 |
|  |  |  |  |  |  |  |  |  |  |  |  |  |  |  |  |  | High | DMTMSPPGAHGVK | 1 |  | 0,0000 | 2,39 | 2 | 1327,61431 | 0,68 | 36,71 |
|  |  |  |  |  |  |  |  |  |  |  |  |  |  |  |  |  | High | ILLESAIR | 2 |  | 0,0000 | 2,17 | 2 | 914,56688 | -0,12 | 41,76 |
|  |  |  |  |  |  |  |  |  |  |  |  |  |  |  |  |  | High | GPMLLGVK | 1 |  | 0,0000 | 2,11 | 2 | 814,48522 | -0,45 | 41,05 |
|  |  |  |  |  |  |  |  |  |  |  |  |  |  |  |  |  | Medium | DFNSYGSR | 1 |  | 0,0000 | 1,98 | 2 | 945,40557 | -0,58 | 36,42 |
|  |  |  |  |  |  |  |  |  |  |  |  |  |  |  |  |  | Medium | MFVDYNEPQVER | 1 |  | 0,0000 | 1,93 | 2 | 1526,69634 | 1,22 | 41,31 |
|  |  |  |  |  |  |  |  |  |  |  |  |  |  |  |  |  | Medium | DFnSYGSR | 1 | N3(Deamidated) | 0,0000 | 1,90 | 2 | 946,39012 | 0,00 | 37,41 |
|  |  |  |  |  |  |  |  |  |  |  |  |  |  |  |  |  | Medium | IIDWENTSPK | 1 |  | 0,0000 | 1,89 | 2 | 1202,60491 | -0,27 | 41,23 |
|  |  |  |  |  |  |  |  |  |  |  |  |  |  |  |  |  | Medium | GPmLLGVK | 1 | M3(Oxidation) | 0,0172 | 1,71 | 2 | 830,48082 | 0,39 | 39,18 |
|  |  |  |  |  |  |  |  |  |  |  |  |  |  |  |  |  | Medium | GFAVPK | 1 |  | 0,0000 | 1,70 | 2 | 618,36131 | 0,49 | 37,04 |
|  |  |  |  |  |  |  |  |  |  |  |  |  |  |  |  |  | Medium | TIHIPTGEK | 2 |  | 0,0000 | 1,59 | 2 | 995,55223 | 0,17 | 35,49 |
|  |  |  |  |  |  |  |  |  |  |  |  |  |  |  |  |  | Medium | VTLQYLK | 1 |  | 0,0000 | 1,52 | 2 | 864,51891 | -0,05 | 40,08 |
|  |  |  |  |  |  |  |  |  |  |  |  |  |  |  |  |  | Medium | GTFANIR | 1 |  | 0,0000 | 1,48 | 2 | 778,42058 | -0,09 | 37,13 |
|  |  |  |  |  |  |  |  |  |  |  |  |  |  |  |  |  | Medium | SFVcTLR | 1 | C4(Carbamidomethyl) | 0,0000 | 1,47 | 2 | 882,45012 | -0,13 | 38,99 |
|  |  |  |  |  |  |  |  |  |  |  |  |  |  |  |  |  | Medium | TSLAPGSGVVTK | 3 |  | 0,0000 | 1,46 | 2 | 1116,62578 | -0,17 | 36,86 |
| **4829** | M5VSW8 | aconitate hydratase | Uncharacterized protein OS=Prunus persica GN=PRUPE_ppa001138mg PE=4 SV=1 - [M5VSW8_PRUPE] | 99% to GI:645273719 Prunus mume, E value 0 | Prunus mume | Chloroplast | 02.10-Energy/TCA pathway | 18,75 | 10,80 | 1 | 6 | 11 | 13 | 898 | 98,2 | 6,33 | High | TGEDADTLGLTGEER | 1 |  | 0,0000 | 3,24 | 2 | 1563,71501 | 1,05 | 40,31 |
|  |  |  |  |  |  |  |  |  |  |  |  |  |  |  |  |  | High | ILLESAIR | 1 |  | 0,0000 | 2,60 | 2 | 914,56688 | -0,12 | 41,64 |
|  |  |  |  |  |  |  |  |  |  |  |  |  |  |  |  |  | Medium | SFVcTLR | 1 | C4(Carbamidomethyl) | 0,0000 | 1,98 | 2 | 882,44988 | -0,41 | 38,85 |
|  |  |  |  |  |  |  |  |  |  |  |  |  |  |  |  |  | Medium | GPmLLGVK | 1 | M3(Oxidation) | 0,0000 | 1,97 | 2 | 830,48088 | 0,47 | 39,06 |
|  |  |  |  |  |  |  |  |  |  |  |  |  |  |  |  |  | Medium | IIDWENTSPK | 1 |  | 0,0000 | 1,92 | 2 | 1202,60600 | 0,64 | 40,50 |
|  |  |  |  |  |  |  |  |  |  |  |  |  |  |  |  |  | Medium | DFNSYGSR | 1 |  | 0,0000 | 1,90 | 2 | 945,40587 | -0,25 | 36,21 |
|  |  |  |  |  |  |  |  |  |  |  |  |  |  |  |  |  | Medium | TSLAPGSGVVTK | 2 |  | 0,0000 | 1,84 | 2 | 1116,62602 | 0,05 | 36,63 |
|  |  |  |  |  |  |  |  |  |  |  |  |  |  |  |  |  | Medium | GFAVPK | 1 |  | 0,0000 | 1,66 | 2 | 618,36119 | 0,29 | 36,94 |
|  |  |  |  |  |  |  |  |  |  |  |  |  |  |  |  |  | Medium | DFnSYGSR | 1 | N3(Deamidated) | 0,0000 | 1,64 | 2 | 946,39031 | 0,19 | 37,16 |
|  |  |  |  |  |  |  |  |  |  |  |  |  |  |  |  |  | Medium | TIHIPTGEK | 1 |  | 0,0000 | 1,60 | 2 | 995,55235 | 0,29 | 35,28 |
|  |  |  |  |  |  |  |  |  |  |  |  |  |  |  |  |  | Medium | VTLQYLK | 1 |  | 0,0000 | 1,49 | 2 | 864,51860 | -0,40 | 39,91 |
|  |  |  |  |  |  |  |  |  |  |  |  |  |  |  |  |  | Medium | GTFANIR | 1 |  | 0,0000 | 1,26 | 2 | 778,42064 | -0,01 | 36,92 |
| **5008** | M5WV03 | Thaumatin | Uncharacterized protein OS=Prunus persica GN=PRUPE_ppa010471mg PE=4 SV=1 - [M5WV03_PRUPE] | 96% to GI:645246258 Prunus mume, E value 8e-172 | Prunus mume | Extracellular | 11.02-Disease/defense/Defense-related | 37,32 | 25,81 | 4 | 5 | 5 | 18 | 248 | 26,0 | 7,30 | High | YccTPPNDKPETcPPTDYSK | 3 | C2(Carbamidomethyl); C3(Carbamidomethyl); C13(Carbamidomethyl) | 0,0000 | 3,14 | 3 | 2430,00620 | 0,60 | 36,71 |
|  |  |  |  |  |  |  |  |  |  |  |  |  |  |  |  |  | High | SAcLAFNQPK | 5 | C3(Carbamidomethyl) | 0,0000 | 3,09 | 2 | 1135,55644 | -0,05 | 40,10 |
|  |  |  |  |  |  |  |  |  |  |  |  |  |  |  |  |  | High | SVDAPSPWSGR | 7 |  | 0,0000 | 2,64 | 2 | 1158,55400 | 0,11 | 41,50 |
|  |  |  |  |  |  |  |  |  |  |  |  |  |  |  |  |  | High | TQcPQAYSYAYDDK | 2 | C3(Carbamidomethyl) | 0,0000 | 2,60 | 2 | 1709,71428 | 1,80 | 40,15 |
|  |  |  |  |  |  |  |  |  |  |  |  |  |  |  |  |  | Medium | VcPAPLQVK | 1 | C2(Carbamidomethyl) | 0,0000 | 1,13 | 2 | 1011,56548 | -0,14 | 39,43 |
| **5414** | M4QFX1 | UDP-glucose 6-dehydrogenase | UDP-glucose 6-dehydrogenase OS=Prunus persica GN=UGD PE=2 SV=1 - [M4QFX1_PRUPE] |  | Prunus persica | Chloroplast | 01.05-Metabolism/Sugars and polysaccharides | 22,92 | 15,15 | 5 | 8 | 8 | 11 | 482 | 53,0 | 6,18 | High | LAANAFLAQR | 1 |  | 0,0000 | 3,06 | 2 | 1074,60539 | -0,11 | 42,92 |
|  |  |  |  |  |  |  |  |  |  |  |  |  |  |  |  |  | High | VVASMFNTVAK | 2 |  | 0,0000 | 2,93 | 2 | 1166,62370 | -0,14 | 42,47 |
|  |  |  |  |  |  |  |  |  |  |  |  |  |  |  |  |  | High | NVVNVDQLR | 2 |  | 0,0000 | 2,70 | 2 | 1056,57964 | -0,04 | 40,42 |
|  |  |  |  |  |  |  |  |  |  |  |  |  |  |  |  |  | High | QVTVVGDAYEAAK | 1 |  | 0,0000 | 2,16 | 2 | 1350,68999 | -0,03 | 40,46 |
|  |  |  |  |  |  |  |  |  |  |  |  |  |  |  |  |  | High | AADLTYWESAAR | 1 |  | 0,0000 | 2,11 | 2 | 1353,64348 | 0,05 | 45,43 |
|  |  |  |  |  |  |  |  |  |  |  |  |  |  |  |  |  | Medium | IEAWNSDK | 1 |  | 0,0000 | 1,69 | 2 | 962,45781 | -0,02 | 37,88 |
|  |  |  |  |  |  |  |  |  |  |  |  |  |  |  |  |  | Medium | ETPAIDVcK | 2 | C8(Carbamidomethyl) | 0,0000 | 1,63 | 2 | 1032,50298 | -0,09 | 38,85 |
|  |  |  |  |  |  |  |  |  |  |  |  |  |  |  |  |  | Medium | RIEAWNSDK | 1 |  | 0,0000 | 1,25 | 2 | 1118,55876 | -0,17 | 35,65 |
| **5509** | M5WP36 | delta-1-pyrroline-5-carboxylate dehydrogenase | Uncharacterized protein OS=Prunus persica GN=PRUPE_ppa003713mg PE=3 SV=1 - [M5WP36_PRUPE] | 99% to GI:645269075 Prunus mume, E value 0 | Prunus persica | Mitochondrion | 01.01-Metabolism/Amino acid | 10,55 | 9,75 | 1 | 6 | 6 | 7 | 554 | 61,7 | 7,02 | High | LEDAGFDWK | 1 |  | 0,0000 | 2,49 | 2 | 1080,50005 | 0,31 | 42,92 |
|  |  |  |  |  |  |  |  |  |  |  |  |  |  |  |  |  | High | EIIYDVGPLPK | 1 |  | 0,0000 | 2,39 | 2 | 1243,69316 | -0,13 | 43,68 |
|  |  |  |  |  |  |  |  |  |  |  |  |  |  |  |  |  | High | LLQISGSK | 1 |  | 0,0000 | 2,10 | 2 | 845,50914 | 0,01 | 37,40 |
|  |  |  |  |  |  |  |  |  |  |  |  |  |  |  |  |  | Medium | GAGIGTPEAIK | 1 |  | 0,0000 | 1,82 | 2 | 1013,56236 | -0,28 | 37,15 |
|  |  |  |  |  |  |  |  |  |  |  |  |  |  |  |  |  | Medium | LLFGGEPLK | 1 |  | 0,0000 | 1,75 | 2 | 973,57158 | -0,18 | 43,47 |
|  |  |  |  |  |  |  |  |  |  |  |  |  |  |  |  |  | Medium | LALDLK | 2 |  | 0,0492 | 1,16 | 2 | 672,42912 | 0,05 | 40,45 |
|  |  |  |  |  |  |  |  |  |  |  |  |  |  |  |  |  | High | mDAQSQQPQVGVVK | 1 | N-Term(Acetyl) | 0,0000 | 2,21 | 2 | 1556,77397 | 0,11 | 41,69 |
| **5703** | M5VIK0 | laccase | Uncharacterized protein OS=Prunus persica GN=PRUPE_ppa003580mg PE=4 SV=1 - [M5VIK0_PRUPE] | 96% to GI:645264281 Prunus mume, E value 0 | Prunus mume | Chloroplast | 20.99-Secondary metabolism/Others | 12,64 | 4,79 | 3 | 3 | 3 | 7 | 564 | 62,2 | 6,40 | High | ATVYGAIIILPK | 1 |  | 0,0000 | 2,71 | 2 | 1258,77751 | 0,41 | 47,62 |
|  |  |  |  |  |  |  |  |  |  |  |  |  |  |  |  |  | High | GDVNEIIK | 2 |  | 0,0000 | 2,17 | 2 | 887,48326 | -0,07 | 38,71 |
|  |  |  |  |  |  |  |  |  |  |  |  |  |  |  |  |  | High | nGWATVR | 2 | N1(Deamidated) | 0,0000 | 2,09 | 2 | 804,39977 | -0,19 | 42,33 |
|  |  |  |  |  |  |  |  |  |  |  |  |  |  |  |  |  | High | NGWATVR | 2 |  | 0,0000 | 2,08 | 2 | 803,41606 | 0,20 | 37,33 |
| **5705** | M5X661 | transketolase | Uncharacterized protein OS=Prunus persica GN=PRUPE_ppa001865mg PE=4 SV=1 - [M5X661_PRUPE] |  | Prunus mume | Chloroplast | 02.30-Energy/Photosynthesis | 34,38 | 13,83 | 1 | 10 | 10 | 23 | 752 | 80,9 | 6,84 | High | VTTTIGFGSPNK | 2 |  | 0,0000 | 3,37 | 2 | 1221,64775 | 0,26 | 41,58 |
|  |  |  |  |  |  |  |  |  |  |  |  |  |  |  |  |  | High | NGNTGYDEIR | 2 |  | 0,0000 | 3,09 | 2 | 1138,51225 | -0,11 | 37,62 |
|  |  |  |  |  |  |  |  |  |  |  |  |  |  |  |  |  | High | nGNTGYDEIR | 6 | N1(Deamidated) | 0,0000 | 2,78 | 2 | 1139,49651 | 0,10 | 40,79 |
|  |  |  |  |  |  |  |  |  |  |  |  |  |  |  |  |  | High | ALPTYTPENPADATR | 2 |  | 0,0000 | 2,64 | 2 | 1616,79338 | 1,14 | 42,19 |
|  |  |  |  |  |  |  |  |  |  |  |  |  |  |  |  |  | High | NPYWFNR | 2 |  | 0,0000 | 2,02 | 2 | 996,46874 | 0,09 | 44,34 |
|  |  |  |  |  |  |  |  |  |  |  |  |  |  |  |  |  | Medium | AVTDRPTLIK | 1 |  | 0,0000 | 1,97 | 2 | 1113,66252 | -0,15 | 37,23 |
|  |  |  |  |  |  |  |  |  |  |  |  |  |  |  |  |  | Medium | ESVLPAAVTAR | 2 |  | 0,0000 | 1,69 | 2 | 1113,62578 | -0,48 | 41,62 |
|  |  |  |  |  |  |  |  |  |  |  |  |  |  |  |  |  | Medium | FLAIDAVEK | 1 |  | 0,0000 | 1,56 | 2 | 1005,56133 | -0,25 | 44,38 |
|  |  |  |  |  |  |  |  |  |  |  |  |  |  |  |  |  | Medium | TTETSLVEK | 2 |  | 0,0000 | 1,49 | 2 | 1007,52538 | -0,19 | 36,10 |
|  |  |  |  |  |  |  |  |  |  |  |  |  |  |  |  |  | Medium | SFGDFQK | 2 |  | 0,0000 | 1,23 | 2 | 828,38915 | 0,56 | 40,63 |
|  |  |  |  |  |  |  |  |  |  |  |  |  |  |  |  |  | Medium | ANSYSVHGAALGAK | 1 |  | 0,0000 | 1,08 | 2 | 1345,68584 | -0,08 | 36,00 |
| **5707** | M5WGM1 | 4-hydroxy-3-methylbut-2-en-1-yl diphosphate synthase | Uncharacterized protein OS=Prunus persica GN=PRUPE_ppa002111mg PE=3 SV=1 - [M5WGM1_PRUPE] | 99% to GI:645217430 Prunus mume, E value 0,00 | [Prunus mume](http://www.ncbi.nlm.nih.gov/Taxonomy/Browser/wwwtax.cgi?id=102107) | Chloroplast | 20.2-Secondary metabolism/Terpenoids | 30,72 | 19,86 | 1 | 15 | 15 | 29 | 715 | 79,6 | 6,55 | High | NTKTEYVScPScGR | 3 | C9(Carbamidomethyl); C12(Carbamidomethyl) | 0,0000 | 2,94 | 3 | 1658,72605 | -0,07 | 34,34 |
|  |  |  |  |  |  |  |  |  |  |  |  |  |  |  |  |  | High | IADKGADLVR | 4 |  | 0,0000 | 2,51 | 3 | 1057,60022 | 0,12 | 35,41 |
|  |  |  |  |  |  |  |  |  |  |  |  |  |  |  |  |  | High | TEYVScPScGR | 3 | C6(Carbamidomethyl); C9(Carbamidomethyl) | 0,0000 | 2,19 | 2 | 1315,54143 | 0,64 | 36,03 |
|  |  |  |  |  |  |  |  |  |  |  |  |  |  |  |  |  | High | NTSFNLLQGcR | 2 | C10(Carbamidomethyl) | 0,0000 | 2,19 | 2 | 1309,63298 | 0,91 | 43,77 |
|  |  |  |  |  |  |  |  |  |  |  |  |  |  |  |  |  | High | IMSYYGDSPR | 1 |  | 0,0000 | 2,17 | 2 | 1188,53642 | 0,84 | 39,88 |
|  |  |  |  |  |  |  |  |  |  |  |  |  |  |  |  |  | High | ASNPVIMVQAYR | 1 |  | 0,0000 | 2,07 | 2 | 1348,70488 | 0,47 | 43,50 |
|  |  |  |  |  |  |  |  |  |  |  |  |  |  |  |  |  | High | VSLTEAPEEEIDPcR | 1 | C14(Carbamidomethyl) | 0,0000 | 2,03 | 2 | 1744,80791 | 1,15 | 43,85 |
|  |  |  |  |  |  |  |  |  |  |  |  |  |  |  |  |  | Medium | IDLYVGK | 2 |  | 0,0000 | 1,91 | 2 | 807,46092 | -0,23 | 41,52 |
|  |  |  |  |  |  |  |  |  |  |  |  |  |  |  |  |  | Medium | WVDPPVEE | 1 |  | 0,0000 | 1,59 | 2 | 970,45250 | 0,83 | 45,17 |
|  |  |  |  |  |  |  |  |  |  |  |  |  |  |  |  |  | Medium | VNPGNFADR | 1 |  | 0,0000 | 1,53 | 2 | 989,47972 | -0,25 | 38,33 |
|  |  |  |  |  |  |  |  |  |  |  |  |  |  |  |  |  | Medium | QLPPVDDNDSR | 3 |  | 0,0000 | 1,52 | 2 | 1255,59160 | 0,17 | 39,08 |
|  |  |  |  |  |  |  |  |  |  |  |  |  |  |  |  |  | Medium | DVAATVEQVmR | 1 | M10(Oxidation) | 0,0000 | 1,50 | 2 | 1234,60991 | 0,18 | 41,31 |
|  |  |  |  |  |  |  |  |  |  |  |  |  |  |  |  |  | Medium | ImSYYGDSPR | 1 | M2(Oxidation) | 0,0000 | 1,40 | 2 | 1204,53008 | -0,22 | 37,84 |
|  |  |  |  |  |  |  |  |  |  |  |  |  |  |  |  |  | Medium | LLPEGTR | 2 |  | 0,0000 | 1,20 | 2 | 785,45146 | -0,20 | 37,17 |
|  |  |  |  |  |  |  |  |  |  |  |  |  |  |  |  |  | Medium | LVVSVR | 2 |  | 0,0000 | 1,19 | 2 | 672,44017 | -0,22 | 38,16 |
|  |  |  |  |  |  |  |  |  |  |  |  |  |  |  |  |  | Medium | GmVESAFEFAR | 1 | M2(Oxidation) | 0,0000 | 1,06 | 2 | 1259,57207 | -0,40 | 44,55 |
| **5712** | M5VZ56 | dehydrogenase/reductase SDR family | Uncharacterized protein OS=Prunus persica GN=PRUPE_ppa008500mg PE=4 SV=1 - [M5VZ56_PRUPE] | 99% to GI:645223245 Prunus mume, E value 0 | Prunus mume | Plasma membrane | 11.05-Disease/Defense/Stress responses | 3,81 | 3,65 | 1 | 1 | 1 | 2 | 329 | 35,8 | 9,38 | High | GHFVVmSSAAGK | 2 | M6(Oxidation) | 0,0000 | 2,08 | 2 | 1206,60161 | 6,61 | 40,27 |
| **6101** | M5X1U6 | 6-phosphogluconolactonase | Uncharacterized protein OS=Prunus persica GN=PRUPE_ppa008941mg PE=4 SV=1 - [M5X1U6_PRUPE] | 98% to GI:645257997 Prunus mume, E value 0,00 | Prunus mume | Chloroplast | 02.07-Energy/Pentose phosphate | 18,40 | 15,97 | 1 | 6 | 6 | 13 | 313 | 34,1 | 8,75 | High | FETEEEVAVR | 2 |  | 0,0000 | 3,01 | 2 | 1208,57976 | 0,28 | 40,10 |
|  |  |  |  |  |  |  |  |  |  |  |  |  |  |  |  |  | High | ASAVcTALGK | 3 | C5(Carbamidomethyl) | 0,0000 | 2,55 | 2 | 977,50810 | -0,40 | 38,32 |
|  |  |  |  |  |  |  |  |  |  |  |  |  |  |  |  |  | Medium | YTADLSAK | 3 |  | 0,0000 | 1,73 | 2 | 868,44084 | -0,30 | 37,10 |
|  |  |  |  |  |  |  |  |  |  |  |  |  |  |  |  |  | Medium | LAEAPYK | 2 |  | 0,0000 | 1,53 | 2 | 791,42955 | -0,33 | 36,50 |
|  |  |  |  |  |  |  |  |  |  |  |  |  |  |  |  |  | Medium | LALDGFLSK | 2 |  | 0,0000 | 1,48 | 2 | 963,55077 | -0,25 | 47,01 |
|  |  |  |  |  |  |  |  |  |  |  |  |  |  |  |  |  | Medium | WVTcIK | 1 | C4(Carbamidomethyl) | 0,0000 | 1,03 | 2 | 806,42345 | 0,61 | 39,96 |
| **6206** | M5X0L9 | Phosphoglycerate kinase | Phosphoglycerate kinase OS=Prunus persica GN=PRUPE_ppa005669mg PE=3 SV=1 - [M5X0L9_PRUPE] |  | Prunus persica | Cytosol | 02.01-Energy/Glycolysis | 156,77 | 36,53 | 2 | 16 | 16 | 81 | 449 | 47,5 | 7,03 | High | AQGYSVGSSLVEEDK | 1 |  | 0,0000 | 4,08 | 2 | 1568,74504 | 0,71 | 39,61 |
|  |  |  |  |  |  |  |  |  |  |  |  |  |  |  |  |  | High | GVTTIIGGGDSVAAVEK | 8 |  | 0,0000 | 3,70 | 2 | 1573,84490 | 1,03 | 42,69 |
|  |  |  |  |  |  |  |  |  |  |  |  |  |  |  |  |  | High | ELDYLVGAVGNPK | 1 |  | 0,0000 | 3,64 | 2 | 1374,72734 | 0,67 | 43,86 |
|  |  |  |  |  |  |  |  |  |  |  |  |  |  |  |  |  | High | IAnDcIGEEVEK | 1 | N3(Deamidated); C5(Carbamidomethyl) | 0,0000 | 3,16 | 2 | 1377,61919 | -0,82 | 38,21 |
|  |  |  |  |  |  |  |  |  |  |  |  |  |  |  |  |  | High | VDLNVPLDDNSK | 15 |  | 0,0000 | 3,04 | 2 | 1328,67046 | 0,87 | 43,27 |
|  |  |  |  |  |  |  |  |  |  |  |  |  |  |  |  |  | High | IANDcIGEEVEK | 20 | C5(Carbamidomethyl) | 0,0000 | 2,99 | 2 | 1376,63603 | -0,19 | 56,06 |
|  |  |  |  |  |  |  |  |  |  |  |  |  |  |  |  |  | High | IANDCIGEEVEK | 1 |  | 0,0000 | 2,60 | 2 | 1319,61589 | 0,80 | 41,18 |
|  |  |  |  |  |  |  |  |  |  |  |  |  |  |  |  |  | High | RPFAAIVGGSK | 2 |  | 0,0000 | 2,55 | 3 | 1102,63694 | 0,10 | 36,96 |
|  |  |  |  |  |  |  |  |  |  |  |  |  |  |  |  |  | High | LSELLGVEVK | 8 |  | 0,0000 | 2,43 | 2 | 1086,64031 | -0,23 | 45,84 |
|  |  |  |  |  |  |  |  |  |  |  |  |  |  |  |  |  | High | FAAGTEAIAK | 2 |  | 0,0000 | 2,34 | 2 | 978,52501 | -0,53 | 36,29 |
|  |  |  |  |  |  |  |  |  |  |  |  |  |  |  |  |  | High | LAELSDK | 1 |  | 0,0000 | 2,28 | 2 | 775,41948 | -0,22 | 34,59 |
|  |  |  |  |  |  |  |  |  |  |  |  |  |  |  |  |  | Medium | YSLKPLVPR | 2 |  | 0,0000 | 1,99 | 3 | 1072,65140 | 0,02 | 38,57 |
|  |  |  |  |  |  |  |  |  |  |  |  |  |  |  |  |  | Medium | KLAELSDK | 1 |  | 0,0000 | 1,98 | 2 | 903,51445 | -0,19 | 32,51 |
|  |  |  |  |  |  |  |  |  |  |  |  |  |  |  |  |  | Medium | AHASTEGVAK | 1 |  | 0,0000 | 1,75 | 2 | 970,49498 | -0,31 | 29,99 |
|  |  |  |  |  |  |  |  |  |  |  |  |  |  |  |  |  | Medium | AAVPTIK | 13 |  | 0,0000 | 1,57 | 2 | 699,44005 | 0,08 | 36,44 |
|  |  |  |  |  |  |  |  |  |  |  |  |  |  |  |  |  | Medium | vDLnVPLDDnSK | 1 | N-Term(Acetyl); N4(Deamidated); N10(Deamidated) | 0,0000 | 1,42 | 2 | 1372,63457 | -9,71 | 41,88 |
|  |  |  |  |  |  |  |  |  |  |  |  |  |  |  |  |  | Medium | EEEKNDPEFSK | 1 |  | 0,0000 | 1,31 | 3 | 1351,60050 | -0,58 | 31,93 |
|  |  |  |  |  |  |  |  |  |  |  |  |  |  |  |  |  | Medium | YLQGHGAK | 1 |  | 0,0000 | 1,17 | 2 | 873,45757 | -0,21 | 29,96 |
|  |  |  |  |  |  |  |  |  |  |  |  |  |  |  |  |  | Medium | SVSTLKEAELK | 1 |  | 0,0000 | 1,06 | 2 | 1204,67961 | 1,01 | 35,48 |
| **6220** | M5XE62 | 26S proteasome non-ATPase regulatory subunit 11 homolog | Uncharacterized protein OS=Prunus persica GN=PRUPE_ppa006204mg PE=4 SV=1 - [M5XE62_PRUPE] | 96% to GI:658009294 Malus domestica, E value 0 | Malus domestica | Nucleus | 06.13-Protein destination and storage/Proteolysis | 15,28 | 23,22 | 20 | 9 | 9 | 9 | 422 | 47,1 | 6,48 | High | ILENPSSSSEALR | 1 |  | 0,0000 | 2,62 | 2 | 1402,71733 | 0,02 | 37,91 |
|  |  |  |  |  |  |  |  |  |  |  |  |  |  |  |  |  | High | AQLEEDPIVHR | 1 |  | 0,0000 | 2,42 | 2 | 1306,67583 | 0,60 | 36,90 |
|  |  |  |  |  |  |  |  |  |  |  |  |  |  |  |  |  | High | IMVSQADDVAGIISSK | 1 |  | 0,0000 | 2,40 | 2 | 1633,85332 | 4,09 | 44,02 |
|  |  |  |  |  |  |  |  |  |  |  |  |  |  |  |  |  | High | VPGTSDLQISLcK | 1 | C12(Carbamidomethyl) | 0,0000 | 2,24 | 2 | 1417,73967 | 2,87 | 42,46 |
|  |  |  |  |  |  |  |  |  |  |  |  |  |  |  |  |  | High | VVDSLYVR | 1 |  | 0,0000 | 2,02 | 2 | 950,53038 | -0,22 | 39,09 |
|  |  |  |  |  |  |  |  |  |  |  |  |  |  |  |  |  | Medium | AGLQYVGPELDAMK | 1 |  | 0,0000 | 1,83 | 2 | 1491,75102 | -0,17 | 44,37 |
|  |  |  |  |  |  |  |  |  |  |  |  |  |  |  |  |  | Medium | LASLLMESK | 1 |  | 0,0000 | 1,74 | 2 | 991,54924 | -0,06 | 41,58 |
|  |  |  |  |  |  |  |  |  |  |  |  |  |  |  |  |  | Medium | EMVQWTR | 1 |  | 0,0000 | 1,56 | 2 | 949,45537 | -0,73 | 39,72 |
|  |  |  |  |  |  |  |  |  |  |  |  |  |  |  |  |  | Medium | LFETALR | 1 |  | 0,0000 | 1,49 | 2 | 849,48290 | -0,03 | 40,89 |
| **6413** | M5WPG7 | 6-phosphogluconate dehydrogenase, decarboxylating | 6-phosphogluconate dehydrogenase, decarboxylating OS=Prunus persica GN=PRUPE_ppa005270mg PE=3 SV=1 - [M5WPG7_PRUPE] |  | Prunus mume | Chloroplast | 02.07-Energy/Pentose phosphate | 36,24 | 19,62 | 2 | 6 | 10 | 23 | 469 | 50,8 | 7,46 | High | LPANLVQAQR | 3 |  | 0,0000 | 3,08 | 2 | 1109,64214 | -0,44 | 39,38 |
|  |  |  |  |  |  |  |  |  |  |  |  |  |  |  |  |  | High | EVIGSVTSGIDK | 3 |  | 0,0000 | 2,98 | 2 | 1204,64238 | 0,30 | 41,62 |
|  |  |  |  |  |  |  |  |  |  |  |  |  |  |  |  |  | High | DDLADGFLVDK | 1 |  | 0,0000 | 2,92 | 2 | 1207,58452 | 0,28 | 48,74 |
|  |  |  |  |  |  |  |  |  |  |  |  |  |  |  |  |  | High | mEASPALSR | 2 | N-Term(Acetyl) | 0,0000 | 2,60 | 2 | 1003,48747 | -0,29 | 43,57 |
|  |  |  |  |  |  |  |  |  |  |  |  |  |  |  |  |  | High | YLSGLKEER | 4 |  | 0,0000 | 2,21 | 2 | 1094,58415 | 0,06 | 36,67 |
|  |  |  |  |  |  |  |  |  |  |  |  |  |  |  |  |  | High | GFPISVYNR | 2 |  | 0,0000 | 2,15 | 2 | 1052,55217 | -0,21 | 44,53 |
|  |  |  |  |  |  |  |  |  |  |  |  |  |  |  |  |  | Medium | SVIILVK | 3 |  | 0,0000 | 1,93 | 2 | 771,53392 | 0,04 | 43,68 |
|  |  |  |  |  |  |  |  |  |  |  |  |  |  |  |  |  | Medium | LIDDVR | 2 |  | 0,0000 | 1,82 | 2 | 730,40941 | -0,01 | 38,37 |
|  |  |  |  |  |  |  |  |  |  |  |  |  |  |  |  |  | Medium | AVFLDR | 2 |  | 0,0000 | 1,53 | 2 | 720,40392 | -0,03 | 40,97 |
|  |  |  |  |  |  |  |  |  |  |  |  |  |  |  |  |  | Medium | LAQKSGSGVGALN | 1 |  | 0,0000 | 1,50 | 2 | 1201,65300 | -0,49 | 36,55 |
| **6419** | M5WP70 | Uncharacterized aminotransferase | Uncharacterized protein OS=Prunus persica GN=PRUPE_ppa004689mg PE=3 SV=1 - [M5WP70_PRUPE] | 98% to GI:645221853 Prunus mume, E value 0,00 | Prunus persica | Mitochondrion | 01.06-Metabolism/Lipid and sterol | 14,90 | 10,71 | 3 | 5 | 6 | 17 | 467 | 52,3 | 6,43 | High | NHDAVVEMGK | 4 |  | 0,0000 | 2,81 | 2 | 1099,52031 | 0,16 | 35,56 |
|  |  |  |  |  |  |  |  |  |  |  |  |  |  |  |  |  | High | DAVNQLVR | 2 |  | 0,0000 | 2,07 | 2 | 914,50517 | -0,30 | 40,79 |
|  |  |  |  |  |  |  |  |  |  |  |  |  |  |  |  |  | High | FGVEVR | 2 |  | 0,0000 | 2,06 | 2 | 706,38854 | 0,35 | 39,89 |
|  |  |  |  |  |  |  |  |  |  |  |  |  |  |  |  |  | Medium | NHDAVVEmGK | 3 | M8(Oxidation) | 0,0000 | 1,60 | 2 | 1115,51421 | -0,75 | 32,72 |
|  |  |  |  |  |  |  |  |  |  |  |  |  |  |  |  |  | Medium | DGEVGSTTGYVR | 1 |  | 0,0000 | 1,51 | 2 | 1240,58196 | 1,20 | 38,39 |
|  |  |  |  |  |  |  |  |  |  |  |  |  |  |  |  |  | Medium | TQVPIEPK | 3 |  | 0,0000 | 1,48 | 2 | 911,51921 | -0,54 | 37,94 |
|  |  |  |  |  |  |  |  |  |  |  |  |  |  |  |  |  | Medium | IVIFcR | 2 | C5(Carbamidomethyl) | 0,0000 | 1,21 | 2 | 807,45433 | -0,33 | 43,09 |
| **6708** | M5XQJ0 | Phosphoenolpyruvate carboxykinase [ATP] | Uncharacterized protein OS=Prunus persica GN=PRUPE_ppa002490mg PE=3 SV=1 - [M5XQJ0_PRUPE] | 99% to GI:645224569 Prunus mume, E value 0 | Prunus mume | Cytoplasm | 02.02-Energy/Gluconeogenesis | 34,92 | 21,29 | 2 | 12 | 12 | 26 | 667 | 74,0 | 7,23 | High | AAEnGGFSFTR | 2 | N4(Deamidated) | 0,0000 | 3,52 | 2 | 1157,52361 | 1,18 | 40,62 |
|  |  |  |  |  |  |  |  |  |  |  |  |  |  |  |  |  | High | AAENGGFSFTR | 1 |  | 0,0000 | 3,14 | 2 | 1156,53850 | 0,24 | 40,05 |
|  |  |  |  |  |  |  |  |  |  |  |  |  |  |  |  |  | High | AAYPIEYIPnAK | 2 | N10(Deamidated) | 0,0000 | 3,06 | 2 | 1350,69292 | -0,83 | 43,53 |
|  |  |  |  |  |  |  |  |  |  |  |  |  |  |  |  |  | High | EMVILGTQYAGEmK | 1 | M13(Oxidation) | 0,0000 | 2,49 | 2 | 1585,76225 | 1,34 | 42,67 |
|  |  |  |  |  |  |  |  |  |  |  |  |  |  |  |  |  | Medium | IQTHPEATSEVcHDDSGTPVK | 2 | C12(Carbamidomethyl) | 0,0000 | 2,48 | 3 | 2308,05338 | 0,99 | 34,14 |
|  |  |  |  |  |  |  |  |  |  |  |  |  |  |  |  |  | High | YAAMLSEK | 1 |  | 0,0000 | 2,30 | 2 | 912,44902 | -0,60 | 37,09 |
|  |  |  |  |  |  |  |  |  |  |  |  |  |  |  |  |  | High | AAYPIEYIPNAK | 5 |  | 0,0000 | 2,23 | 2 | 1349,71086 | 0,62 | 43,41 |
|  |  |  |  |  |  |  |  |  |  |  |  |  |  |  |  |  | High | IPcVGPHPK | 2 | C3(Carbamidomethyl) | 0,0000 | 2,10 | 2 | 1004,53441 | -0,23 | 33,91 |
|  |  |  |  |  |  |  |  |  |  |  |  |  |  |  |  |  | High | AVDYLNSLDK | 1 |  | 0,0000 | 2,01 | 2 | 1137,57878 | 0,10 | 41,94 |
|  |  |  |  |  |  |  |  |  |  |  |  |  |  |  |  |  | Medium | LLNASYTK | 1 |  | 0,0000 | 1,95 | 2 | 909,50377 | -0,29 | 36,28 |
|  |  |  |  |  |  |  |  |  |  |  |  |  |  |  |  |  | Medium | GSFITATGALATLSGAK | 1 |  | 0,0000 | 1,89 | 2 | 1565,85503 | 1,02 | 46,85 |
|  |  |  |  |  |  |  |  |  |  |  |  |  |  |  |  |  | Medium | EMVILGTQYAGEMK | 1 |  | 0,0000 | 1,73 | 2 | 1569,76579 | 0,37 | 44,31 |
|  |  |  |  |  |  |  |  |  |  |  |  |  |  |  |  |  | Medium | SSQPTTPInGK | 1 | N9(Deamidated) | 0,0000 | 1,59 | 2 | 1130,56914 | 0,27 | 34,15 |
|  |  |  |  |  |  |  |  |  |  |  |  |  |  |  |  |  | Medium | IIDAIHSGR | 2 |  | 0,0000 | 1,59 | 2 | 981,54723 | -0,43 | 34,54 |
|  |  |  |  |  |  |  |  |  |  |  |  |  |  |  |  |  | Medium | LTEEILAAGPNF | 1 |  | 0,0000 | 1,56 | 2 | 1274,66265 | -0,09 | 48,76 |
|  |  |  |  |  |  |  |  |  |  |  |  |  |  |  |  |  | Medium | EmVILGTQYAGEmK | 1 | M2(Oxidation); M13(Oxidation) | 0,0000 | 1,55 | 2 | 1601,75481 | -0,15 | 40,12 |
|  |  |  |  |  |  |  |  |  |  |  |  |  |  |  |  |  | Medium | YAAmLSEK | 1 | M4(Oxidation) | 0,0000 | 1,34 | 2 | 928,44396 | -0,57 | 34,52 |
| **6709** | M5XQL3 | subtilisin-like protease | Uncharacterized protein OS=Prunus persica GN=PRUPE_ppa001756mg PE=4 SV=1 - [M5XQL3_PRUPE] | 98% to GI:645227002 Prunus mume, E value 0,00 | Prunus mume | Chloroplast | 06.13-Protein destination and storage/Proteolysis | 51,03 | 15,47 | 3 | 10 | 11 | 25 | 769 | 81,0 | 6,32 | High | GYEAALGPIDEK | 3 |  | 0,0000 | 3,95 | 2 | 1262,62712 | 0,60 | 41,77 |
|  |  |  |  |  |  |  |  |  |  |  |  |  |  |  |  |  | High | TLTNVGTPATYK | 3 |  | 0,0000 | 3,58 | 2 | 1265,67400 | 0,29 | 39,28 |
|  |  |  |  |  |  |  |  |  |  |  |  |  |  |  |  |  | High | SYDDTGLAAVPSSWK | 2 |  | 0,0000 | 3,13 | 2 | 1596,75530 | 0,75 | 45,26 |
|  |  |  |  |  |  |  |  |  |  |  |  |  |  |  |  |  | High | LTTHKDFTcDSSK | 3 | C9(Carbamidomethyl) | 0,0000 | 3,12 | 3 | 1539,71097 | 0,09 | 33,27 |
|  |  |  |  |  |  |  |  |  |  |  |  |  |  |  |  |  | High | VSVSSQTPSVK | 3 |  | 0,0000 | 2,76 | 2 | 1118,60527 | 0,05 | 36,59 |
|  |  |  |  |  |  |  |  |  |  |  |  |  |  |  |  |  | High | SEAFLPASDK | 2 |  | 0,0000 | 2,59 | 2 | 1064,52593 | 0,00 | 39,74 |
|  |  |  |  |  |  |  |  |  |  |  |  |  |  |  |  |  | High | TPEFLGLGK | 3 |  | 0,0000 | 2,38 | 2 | 961,53520 | -0,16 | 45,23 |
|  |  |  |  |  |  |  |  |  |  |  |  |  |  |  |  |  | Medium | LEWSDGK | 3 |  | 0,0000 | 1,99 | 2 | 834,40044 | 1,42 | 39,53 |
|  |  |  |  |  |  |  |  |  |  |  |  |  |  |  |  |  | Medium | SALMTTSYTTYK | 1 |  | 0,0000 | 1,70 | 2 | 1366,65691 | 0,72 | 40,67 |
|  |  |  |  |  |  |  |  |  |  |  |  |  |  |  |  |  | Medium | DFPAFVSLGNEK | 1 |  | 0,0000 | 1,19 | 2 | 1323,65752 | -0,37 | 48,45 |
|  |  |  |  |  |  |  |  |  |  |  |  |  |  |  |  |  | Medium | GISLYR | 1 |  | 0,0000 | 1,17 | 2 | 708,40410 | 0,25 | 39,38 |
| **7004** | M5WTZ4 | Glutathione S-transferase | Uncharacterized protein OS=Prunus persica GN=PRUPE_ppa011337mg PE=3 SV=1 - [M5WTZ4_PRUPE] | 95% to GI:645246909 Prunus mume, E value 8e-145 | Prunus mume | Cytoplasm | 11.06-Disease/Defense/Detoxification | 27,44 | 16,82 | 4 | 4 | 4 | 14 | 214 | 24,7 | 6,30 | High | VLDIYEER | 3 |  | 0,0000 | 2,72 | 2 | 1036,53069 | -0,29 | 41,32 |
|  |  |  |  |  |  |  |  |  |  |  |  |  |  |  |  |  | High | VIQESEEKLGK | 2 |  | 0,0000 | 2,33 | 2 | 1259,68401 | -0,17 | 34,70 |
|  |  |  |  |  |  |  |  |  |  |  |  |  |  |  |  |  | High | VLVcLVEK | 6 | C4(Carbamidomethyl) | 0,0000 | 2,21 | 2 | 959,55876 | -0,73 | 42,00 |
|  |  |  |  |  |  |  |  |  |  |  |  |  |  |  |  |  | Medium | SQGTALLGK | 3 |  | 0,0000 | 1,78 | 2 | 874,49889 | -0,47 | 39,18 |
| **7106** | M5WGS1 | extradiol ring-cleavage dioxygenase | Uncharacterized protein OS=Prunus persica GN=PRUPE_ppa009849mg PE=4 SV=1 - [M5WGS1_PRUPE] | 97% to GI:645218731 Prunus mume, E value 0 | Prunus persica | Cell wall | 20.99-Secondary metabolism/Others | 99,78 | 22,26 | 1 | 7 | 7 | 54 | 274 | 30,1 | 6,86 | High | YEDVNQYEEK | 18 |  | 0,0000 | 3,11 | 2 | 1316,56462 | 0,37 | 35,80 |
|  |  |  |  |  |  |  |  |  |  |  |  |  |  |  |  |  | High | DATYHYNMGR | 4 |  | 0,0000 | 2,73 | 2 | 1227,52178 | 0,50 | 36,50 |
|  |  |  |  |  |  |  |  |  |  |  |  |  |  |  |  |  | High | YEDVnQYEEK | 2 | N5(Deamidated) | 0,0000 | 2,65 | 2 | 1317,54888 | 0,55 | 35,25 |
|  |  |  |  |  |  |  |  |  |  |  |  |  |  |  |  |  | High | YPAPGSPNLAGR | 21 |  | 0,0000 | 2,34 | 2 | 1199,61711 | 0,26 | 50,96 |
|  |  |  |  |  |  |  |  |  |  |  |  |  |  |  |  |  | Medium | DALLEGR | 2 |  | 0,0000 | 1,79 | 2 | 773,41484 | -0,52 | 37,96 |
|  |  |  |  |  |  |  |  |  |  |  |  |  |  |  |  |  | Medium | FLQSWK | 1 |  | 0,0000 | 1,75 | 2 | 808,43511 | -0,15 | 39,71 |
|  |  |  |  |  |  |  |  |  |  |  |  |  |  |  |  |  | Medium | DLLTASGFPR | 1 |  | 0,0000 | 1,71 | 2 | 1076,57317 | -0,35 | 45,33 |
|  |  |  |  |  |  |  |  |  |  |  |  |  |  |  |  |  | Medium | SmYQLK | 1 | M2(Oxidation) | 0,1317 | 1,45 | 2 | 785,38616 | -0,09 | 34,40 |
|  |  |  |  |  |  |  |  |  |  |  |  |  |  |  |  |  | Medium | DATYHYNmGR | 3 | M8(Oxidation) | 0,0000 | 1,18 | 2 | 1243,51677 | 0,56 | 33,02 |
|  |  |  |  |  |  |  |  |  |  |  |  |  |  |  |  |  | Medium | SMYQLK | 1 |  | 0,0000 | 1,16 | 2 | 769,39141 | 0,12 | 36,87 |
| **7109** | M5W9G4 | S-formylglutathione hydrolase | Uncharacterized protein OS=Prunus persica GN=PRUPE_ppa009542mg PE=4 SV=1 - [M5W9G4_PRUPE] | 99% to GI:645278527 Prunus mume, E value 0,00 | [Prunus mume](http://www.ncbi.nlm.nih.gov/Taxonomy/Browser/wwwtax.cgi?id=102107) | Chloroplast | 11.06-Disease/Defense/Detoxification | 44,26 | 25,09 | 2 | 7 | 7 | 22 | 287 | 32,2 | 7,08 | High | VASSEGVALIAPDTSPR | 4 |  | 0,0000 | 3,87 | 2 | 1669,87688 | 0,75 | 43,74 |
|  |  |  |  |  |  |  |  |  |  |  |  |  |  |  |  |  | High | mETKPSEISSSK | 3 | N-Term(Acetyl) | 0,0000 | 3,20 | 2 | 1365,65764 | 0,70 | 38,64 |
|  |  |  |  |  |  |  |  |  |  |  |  |  |  |  |  |  | High | AFSNYLGGNK | 2 |  | 0,0000 | 2,39 | 2 | 1070,52641 | -0,15 | 41,38 |
|  |  |  |  |  |  |  |  |  |  |  |  |  |  |  |  |  | High | LLNENFPQLDTSR | 1 |  | 0,0000 | 2,22 | 2 | 1546,78740 | 0,87 | 45,24 |
|  |  |  |  |  |  |  |  |  |  |  |  |  |  |  |  |  | High | mYDYVVK | 3 | M1(Oxidation) | 0,0000 | 2,07 | 2 | 933,43907 | 0,45 | 39,90 |
|  |  |  |  |  |  |  |  |  |  |  |  |  |  |  |  |  | Medium | MYDYVVK | 3 |  | 0,0000 | 1,92 | 2 | 917,44353 | -0,23 | 40,89 |
|  |  |  |  |  |  |  |  |  |  |  |  |  |  |  |  |  | Medium | mFGGYNK | 1 | M1(Oxidation) | 0,0000 | 1,84 | 2 | 832,36577 | -0,08 | 35,61 |
|  |  |  |  |  |  |  |  |  |  |  |  |  |  |  |  |  | Medium | MFGGYNK | 2 |  | 0,0000 | 1,76 | 2 | 816,37078 | -0,18 | 38,54 |
|  |  |  |  |  |  |  |  |  |  |  |  |  |  |  |  |  | Medium | VPLLLR | 3 |  | 0,0000 | 1,26 | 2 | 710,49242 | 0,08 | 44,04 |
| **7113** | M5Y9C1 | Malate dehydrogenase | Malate dehydrogenase OS=Prunus persica GN=PRUPE_ppa008435mg PE=3 SV=1 - [M5Y9C1_PRUPE] |  | Prunus persica | Cytoplasm | 02.10-Energy/TCA pathway | 46,57 | 40,36 | 3 | 15 | 15 | 29 | 332 | 35,4 | 6,79 | High | LDATADELSEEK | 3 |  | 0,0000 | 3,55 | 2 | 1320,61724 | 0,48 | 40,06 |
|  |  |  |  |  |  |  |  |  |  |  |  |  |  |  |  |  | High | VLVVANPANTNALILK | 4 |  | 0,0000 | 3,31 | 2 | 1649,99700 | 1,24 | 47,06 |
|  |  |  |  |  |  |  |  |  |  |  |  |  |  |  |  |  | High | VLVTGAAGQIGYALVPMIAR | 2 |  | 0,0000 | 3,13 | 2 | 2000,13970 | 1,73 | 50,58 |
|  |  |  |  |  |  |  |  |  |  |  |  |  |  |  |  |  | High | LNVQVSDVK | 2 |  | 0,0000 | 3,02 | 2 | 1001,56212 | -0,51 | 38,75 |
|  |  |  |  |  |  |  |  |  |  |  |  |  |  |  |  |  | High | DVMSKNVSIYK | 1 |  | 0,0000 | 2,64 | 2 | 1283,66789 | 1,12 | 39,08 |
|  |  |  |  |  |  |  |  |  |  |  |  |  |  |  |  |  | High | KLDATADELSEEK | 1 |  | 0,0000 | 2,55 | 2 | 1448,71330 | 1,20 | 37,65 |
|  |  |  |  |  |  |  |  |  |  |  |  |  |  |  |  |  | High | mELVDAAFPLLK | 2 | M1(Oxidation) | 0,0000 | 2,52 | 2 | 1362,73369 | -0,11 | 49,32 |
|  |  |  |  |  |  |  |  |  |  |  |  |  |  |  |  |  | High | MELVDAAFPLLK | 2 |  | 0,0000 | 2,50 | 2 | 1346,73967 | 0,56 | 50,78 |
|  |  |  |  |  |  |  |  |  |  |  |  |  |  |  |  |  | High | IVQGLSIDEFSR | 2 |  | 0,0000 | 2,30 | 2 | 1363,72246 | 0,58 | 46,53 |
|  |  |  |  |  |  |  |  |  |  |  |  |  |  |  |  |  | Medium | SQASALEKHAAANcK | 2 | C14(Carbamidomethyl) | 0,0000 | 2,09 | 3 | 1585,77536 | 0,11 | 33,88 |
|  |  |  |  |  |  |  |  |  |  |  |  |  |  |  |  |  | Medium | KKLDATADELSEEK | 1 |  | 0,0000 | 1,69 | 3 | 1576,80686 | 0,20 | 35,42 |
|  |  |  |  |  |  |  |  |  |  |  |  |  |  |  |  |  | Medium | NITcLTR | 2 | C4(Carbamidomethyl) | 0,0000 | 1,51 | 2 | 877,45592 | -0,14 | 37,40 |
|  |  |  |  |  |  |  |  |  |  |  |  |  |  |  |  |  | Medium | EFAPSIPEK | 2 |  | 0,0000 | 1,48 | 2 | 1017,52513 | -0,06 | 41,41 |
|  |  |  |  |  |  |  |  |  |  |  |  |  |  |  |  |  | Medium | KDVMSKNVSIYK | 1 |  | 0,0593 | 1,27 | 3 | 1411,76053 | -0,63 | 35,95 |
|  |  |  |  |  |  |  |  |  |  |  |  |  |  |  |  |  | Medium | ALAYScLS | 1 | C6(Carbamidomethyl) | 0,0000 | 1,06 | 2 | 884,41820 | -0,06 | 44,57 |
|  |  |  |  |  |  |  |  |  |  |  |  |  |  |  |  |  | Medium | NVSIYK | 1 |  | 0,0000 | 1,02 | 2 | 723,40386 | 0,38 | 36,67 |
| **7202** | M5X598 | cinnamoyl-CoA reductase | Uncharacterized protein OS=Prunus persica GN=PRUPE_ppa006945mg PE=4 SV=1 - [M5X598_PRUPE] | 99% to GI:645245246 Prunus mume, E value 0 | Prunus mume | Cytoplasm | 20.1-Secondary metabolism/Phenylpropanoids/Phenolics | 37,53 | 26,48 | 4 | 11 | 11 | 23 | 389 | 43,0 | 7,46 | High | AVAEQAAWEEAK | 2 |  | 0,0000 | 3,46 | 2 | 1302,63323 | 0,53 | 41,95 |
|  |  |  |  |  |  |  |  |  |  |  |  |  |  |  |  |  | High | LQDLGLEFTPVK | 2 |  | 0,0000 | 3,10 | 2 | 1359,75286 | 0,69 | 49,85 |
|  |  |  |  |  |  |  |  |  |  |  |  |  |  |  |  |  | High | ADLLDYESLK | 2 |  | 0,0000 | 3,09 | 2 | 1166,59416 | 0,15 | 48,72 |
|  |  |  |  |  |  |  |  |  |  |  |  |  |  |  |  |  | High | NVIVAAAEAK | 3 |  | 0,0000 | 2,53 | 2 | 985,56719 | -0,55 | 40,35 |
|  |  |  |  |  |  |  |  |  |  |  |  |  |  |  |  |  | High | GDVVEILAK | 2 |  | 0,0000 | 2,46 | 2 | 943,54546 | -0,50 | 46,55 |
|  |  |  |  |  |  |  |  |  |  |  |  |  |  |  |  |  | High | KADLLDYESLK | 3 |  | 0,0000 | 2,29 | 2 | 1294,68926 | 0,23 | 44,17 |
|  |  |  |  |  |  |  |  |  |  |  |  |  |  |  |  |  | High | FFPEYPIPTK | 2 |  | 0,0000 | 2,03 | 2 | 1238,64580 | 0,14 | 48,79 |
|  |  |  |  |  |  |  |  |  |  |  |  |  |  |  |  |  | Medium | NWYcYGK | 3 | C4(Carbamidomethyl) | 0,0000 | 1,98 | 2 | 990,41368 | -0,14 | 43,46 |
|  |  |  |  |  |  |  |  |  |  |  |  |  |  |  |  |  | Medium | GHLAVPKPQEDSIK | 1 |  | 0,0000 | 1,44 | 3 | 1518,82715 | -0,26 | 38,26 |
|  |  |  |  |  |  |  |  |  |  |  |  |  |  |  |  |  | Medium | YLcAESVLHR | 1 | C3(Carbamidomethyl) | 0,0000 | 1,26 | 2 | 1247,62065 | 0,40 | 42,11 |
|  |  |  |  |  |  |  |  |  |  |  |  |  |  |  |  |  | Medium | QcLYDTVK | 2 | C2(Carbamidomethyl) | 0,0000 | 1,15 | 2 | 1026,49224 | -0,24 | 41,32 |
| **7212** | M5W2H9 | Fructose-bisphosphate aldolase | Fructose-bisphosphate aldolase OS=Prunus persica GN=PRUPE_ppa007696mg PE=3 SV=1 - [M5W2H9_PRUPE] |  | Prunus persica | Cytoplasm | 02.01-Energy/Glycolysis | 70,16 | 38,55 | 3 | 15 | 17 | 44 | 358 | 38,4 | 7,36 | High | KASPEVIAEYTVR | 1 |  | 0,0000 | 3,52 | 3 | 1462,79010 | 0,02 | 40,85 |
|  |  |  |  |  |  |  |  |  |  |  |  |  |  |  |  |  | High | FASINVENVESNR | 2 |  | 0,0000 | 3,41 | 2 | 1478,72502 | 1,06 | 43,17 |
|  |  |  |  |  |  |  |  |  |  |  |  |  |  |  |  |  | High | ANSEATLGTYK | 2 |  | 0,0000 | 3,40 | 2 | 1154,56914 | 0,27 | 37,72 |
|  |  |  |  |  |  |  |  |  |  |  |  |  |  |  |  |  | High | GILAADESTGTIGK | 2 |  | 0,0000 | 3,24 | 2 | 1332,70134 | 0,55 | 42,67 |
|  |  |  |  |  |  |  |  |  |  |  |  |  |  |  |  |  | High | GILAADESTGTIGKR | 3 |  | 0,0000 | 3,17 | 2 | 1488,80303 | 0,87 | 39,55 |
|  |  |  |  |  |  |  |  |  |  |  |  |  |  |  |  |  | High | ASPEVIAEYTVR | 2 |  | 0,0000 | 2,96 | 2 | 1334,69573 | 0,46 | 44,95 |
|  |  |  |  |  |  |  |  |  |  |  |  |  |  |  |  |  | High | AWAGKEENIGK | 4 |  | 0,0000 | 2,85 | 2 | 1202,61675 | 0,22 | 36,93 |
|  |  |  |  |  |  |  |  |  |  |  |  |  |  |  |  |  | High | IGPNEPSQLSINENAnGLAR | 4 | N16(Deamidated) | 0,0000 | 2,80 | 2 | 2095,04277 | 0,59 | 44,08 |
|  |  |  |  |  |  |  |  |  |  |  |  |  |  |  |  |  | High | AKANSEATLGTYK | 4 |  | 0,0000 | 2,46 | 2 | 1353,70134 | 0,31 | 35,53 |
|  |  |  |  |  |  |  |  |  |  |  |  |  |  |  |  |  | High | AQAALLTR | 2 |  | 0,0000 | 2,39 | 2 | 843,50475 | 0,03 | 39,41 |
|  |  |  |  |  |  |  |  |  |  |  |  |  |  |  |  |  | High | TASGKPFVDVLK | 1 |  | 0,0000 | 2,38 | 2 | 1261,71501 | -0,09 | 41,51 |
|  |  |  |  |  |  |  |  |  |  |  |  |  |  |  |  |  | High | LGEGAAESLHVK | 2 |  | 0,0000 | 2,36 | 2 | 1210,64263 | -0,05 | 38,68 |
|  |  |  |  |  |  |  |  |  |  |  |  |  |  |  |  |  | Medium | AKANSEATLGTYKGDAK | 1 |  | 0,0000 | 2,30 | 3 | 1724,88169 | 0,15 | 34,77 |
|  |  |  |  |  |  |  |  |  |  |  |  |  |  |  |  |  | High | ANSEATLGTYKGDAK | 4 |  | 0,0000 | 2,14 | 2 | 1525,75029 | 0,62 | 36,95 |
|  |  |  |  |  |  |  |  |  |  |  |  |  |  |  |  |  | Medium | IGPNEPSQLSINENANGLAR | 1 |  | 0,0000 | 1,74 | 2 | 2094,06059 | 1,47 | 43,87 |
|  |  |  |  |  |  |  |  |  |  |  |  |  |  |  |  |  | Medium | EGGVLPGIK | 5 |  | 0,0000 | 1,66 | 2 | 869,50890 | -0,29 | 44,24 |
|  |  |  |  |  |  |  |  |  |  |  |  |  |  |  |  |  | Medium | VLAAcYK | 3 | C5(Carbamidomethyl) | 0,0000 | 1,57 | 2 | 824,43346 | -0,07 | 37,91 |
|  |  |  |  |  |  |  |  |  |  |  |  |  |  |  |  |  | Medium | FASINVENVESNRR | 1 |  | 0,0000 | 1,51 | 3 | 1634,82401 | -0,35 | 40,68 |
| **7301** | M5X0L9 | Phosphoglycerate kinase | Phosphoglycerate kinase OS=Prunus persica GN=PRUPE_ppa005669mg PE=3 SV=1 - [M5X0L9_PRUPE] |  | Prunus persica | Chloroplast | 02.01-Energy/Glycolysis | 92,79 | 43,21 | 2 | 17 | 17 | 39 | 449 | 47,5 | 7,03 | High | GVTTIIGGGDSVAAVEK | 4 |  | 0,0000 | 4,85 | 2 | 1573,84490 | 1,03 | 44,79 |
|  |  |  |  |  |  |  |  |  |  |  |  |  |  |  |  |  | High | ELDYLVGAVGNPK | 3 |  | 0,0000 | 3,61 | 2 | 1374,72661 | 0,14 | 46,03 |
|  |  |  |  |  |  |  |  |  |  |  |  |  |  |  |  |  | High | FLKPSVAGFLMQK | 2 |  | 0,0000 | 3,52 | 3 | 1465,82306 | -0,40 | 46,17 |
|  |  |  |  |  |  |  |  |  |  |  |  |  |  |  |  |  | High | FYKEEEKNDPEFSK | 3 |  | 0,0000 | 3,27 | 3 | 1789,82755 | -0,25 | 36,09 |
|  |  |  |  |  |  |  |  |  |  |  |  |  |  |  |  |  | High | VDLNVPLDDNSKITDDTR | 2 |  | 0,0000 | 3,03 | 3 | 2030,00443 | 0,33 | 43,64 |
|  |  |  |  |  |  |  |  |  |  |  |  |  |  |  |  |  | High | IANDcIGEEVEK | 2 | C5(Carbamidomethyl) | 0,0000 | 3,03 | 2 | 1376,63713 | 0,61 | 39,21 |
|  |  |  |  |  |  |  |  |  |  |  |  |  |  |  |  |  | High | LVAQLPDGGVLLLENVR | 1 |  | 0,0000 | 3,02 | 2 | 1806,05010 | 0,91 | 51,02 |
|  |  |  |  |  |  |  |  |  |  |  |  |  |  |  |  |  | High | KLAELSDK | 3 |  | 0,0000 | 2,86 | 2 | 903,51396 | -0,73 | 35,87 |
|  |  |  |  |  |  |  |  |  |  |  |  |  |  |  |  |  | High | RPFAAIVGGSK | 4 |  | 0,0000 | 2,73 | 3 | 1102,63657 | -0,23 | 39,01 |
|  |  |  |  |  |  |  |  |  |  |  |  |  |  |  |  |  | High | FAAGTEAIAK | 3 |  | 0,0000 | 2,59 | 2 | 978,52556 | 0,03 | 38,35 |
|  |  |  |  |  |  |  |  |  |  |  |  |  |  |  |  |  | High | VDLNVPLDDNSK | 2 |  | 0,0000 | 2,54 | 2 | 1328,66960 | 0,23 | 45,10 |
|  |  |  |  |  |  |  |  |  |  |  |  |  |  |  |  |  | High | LAELSDK | 2 |  | 0,0000 | 2,33 | 2 | 775,41979 | 0,17 | 37,82 |
|  |  |  |  |  |  |  |  |  |  |  |  |  |  |  |  |  | High | VVPASAIPDGWMGLDIGPDSIK | 1 |  | 0,0000 | 2,32 | 2 | 2238,15117 | 1,59 | 51,93 |
|  |  |  |  |  |  |  |  |  |  |  |  |  |  |  |  |  | High | YSLKPLVPR | 3 |  | 0,0000 | 2,14 | 2 | 1072,65129 | -0,08 | 40,75 |
|  |  |  |  |  |  |  |  |  |  |  |  |  |  |  |  |  | Medium | AAVPTIK | 2 |  | 0,0000 | 1,50 | 2 | 699,43987 | -0,18 | 37,59 |
|  |  |  |  |  |  |  |  |  |  |  |  |  |  |  |  |  | Medium | GVSILLPTDVVIADK | 1 |  | 0,0000 | 1,25 | 2 | 1539,90068 | 0,88 | 50,57 |
|  |  |  |  |  |  |  |  |  |  |  |  |  |  |  |  |  | Medium | YLQGHGAK | 1 |  | 0,0000 | 1,03 | 2 | 873,45732 | -0,49 | 32,10 |
| **7302** | Q9MBD7 | NAD-dependent sorbitol dehydrogenase | NAD-dependent sorbitol dehydrogenase OS=Prunus persica GN=PRUPE_ppa007458mg PE=2 SV=1 - [Q9MBD7_PRUPE] |  | Prunus persica | Cytoplasm | 01.05-Metabolism/Sugars and polysaccharides | 42,97 | 33,79 | 4 | 12 | 12 | 24 | 367 | 39,1 | 6,90 | High | VSTNPQDLEDEVSK | 2 |  | 0,0000 | 3,08 | 2 | 1560,74077 | 1,24 | 44,11 |
|  |  |  |  |  |  |  |  |  |  |  |  |  |  |  |  |  | High | IVIVDVDDER | 3 |  | 0,0000 | 3,07 | 2 | 1172,61589 | 0,07 | 45,45 |
|  |  |  |  |  |  |  |  |  |  |  |  |  |  |  |  |  | High | LPTVGPNDVR | 2 |  | 0,0000 | 2,81 | 2 | 1067,58440 | -0,04 | 42,36 |
|  |  |  |  |  |  |  |  |  |  |  |  |  |  |  |  |  | High | VALEPGIScWR | 2 | C9(Carbamidomethyl) | 0,0000 | 2,63 | 2 | 1287,65154 | 0,04 | 47,40 |
|  |  |  |  |  |  |  |  |  |  |  |  |  |  |  |  |  | High | TMSTALSATRPGGK | 2 |  | 0,0000 | 2,56 | 2 | 1377,71526 | -0,21 | 37,82 |
|  |  |  |  |  |  |  |  |  |  |  |  |  |  |  |  |  | High | EIEEAFETSAR | 3 |  | 0,0000 | 2,32 | 2 | 1281,59612 | 0,25 | 42,47 |
|  |  |  |  |  |  |  |  |  |  |  |  |  |  |  |  |  | High | YNLcPDmK | 1 | C4(Carbamidomethyl); M7(Oxidation) | 0,0000 | 2,18 | 2 | 1056,44829 | -0,60 | 39,59 |
|  |  |  |  |  |  |  |  |  |  |  |  |  |  |  |  |  | Medium | AVGIcGSDVHYLK | 1 | C5(Carbamidomethyl) | 0,0000 | 2,12 | 3 | 1418,70920 | -0,36 | 41,85 |
|  |  |  |  |  |  |  |  |  |  |  |  |  |  |  |  |  | High | SLGADDAVK | 2 |  | 0,0000 | 2,01 | 2 | 875,44689 | -0,06 | 37,23 |
|  |  |  |  |  |  |  |  |  |  |  |  |  |  |  |  |  | Medium | YNLcPDMK | 1 | C4(Carbamidomethyl) | 0,0000 | 2,00 | 2 | 1040,45391 | -0,10 | 42,07 |
|  |  |  |  |  |  |  |  |  |  |  |  |  |  |  |  |  | Medium | FGFSQK | 2 |  | 0,0000 | 1,35 | 2 | 713,36186 | 0,19 | 41,32 |
|  |  |  |  |  |  |  |  |  |  |  |  |  |  |  |  |  | Medium | GGNAIKVmFnL | 1 | M8(Oxidation); N10(Deamidated) | 0,0000 | 1,30 | 2 | 1180,60320 | 0,05 | 47,22 |
|  |  |  |  |  |  |  |  |  |  |  |  |  |  |  |  |  | Medium | NLVPGDR | 2 |  | 0,0000 | 1,15 | 2 | 770,41551 | -0,07 | 39,17 |
| **7308** | M5WD75 | Serine/threonine-protein kinase | Uncharacterized protein OS=Prunus persica GN=PRUPE_ppa007019mg PE=4 SV=1 - [M5WD75_PRUPE] | 99% to GI:645268898 Prunus mume, E value 0 | Prunus mume | Nucleus | 10.04.04-Signal transduction/Kinases | 49,03 | 25,45 | 8 | 12 | 12 | 28 | 385 | 43,0 | 7,36 | High | TEAEISSLR | 2 |  | 0,0000 | 2,77 | 2 | 1005,52068 | -0,48 | 39,72 |
|  |  |  |  |  |  |  |  |  |  |  |  |  |  |  |  |  | High | SLDEQLER | 2 |  | 0,0000 | 2,71 | 2 | 989,48961 | -0,25 | 39,60 |
|  |  |  |  |  |  |  |  |  |  |  |  |  |  |  |  |  | High | GmYDGQDVAVK | 3 | M2(Oxidation) | 0,0000 | 2,63 | 2 | 1198,54106 | 0,11 | 40,76 |
|  |  |  |  |  |  |  |  |  |  |  |  |  |  |  |  |  | High | GMYDGQDVAVK | 2 |  | 0,0000 | 2,55 | 2 | 1182,54631 | 0,25 | 41,34 |
|  |  |  |  |  |  |  |  |  |  |  |  |  |  |  |  |  | High | IADFGVAR | 3 |  | 0,0000 | 2,27 | 2 | 848,46214 | -0,46 | 42,66 |
|  |  |  |  |  |  |  |  |  |  |  |  |  |  |  |  |  | High | TENMLLDK | 2 |  | 0,0000 | 2,23 | 2 | 963,48131 | -0,31 | 42,34 |
|  |  |  |  |  |  |  |  |  |  |  |  |  |  |  |  |  | High | GLSYLHSQK | 3 |  | 0,0000 | 2,16 | 2 | 1032,54741 | 0,12 | 36,84 |
|  |  |  |  |  |  |  |  |  |  |  |  |  |  |  |  |  | High | ccPSSLANVMK | 1 | C1(Carbamidomethyl); C2(Carbamidomethyl) | 0,0000 | 2,09 | 2 | 1266,56450 | 0,40 | 42,29 |
|  |  |  |  |  |  |  |  |  |  |  |  |  |  |  |  |  | High | cWDANPDK | 1 | C1(Carbamidomethyl) | 0,0000 | 2,08 | 2 | 1005,40935 | -0,16 | 38,18 |
|  |  |  |  |  |  |  |  |  |  |  |  |  |  |  |  |  | High | ADQIDLK | 2 |  | 0,0000 | 2,03 | 2 | 802,43065 | 0,12 | 39,90 |
|  |  |  |  |  |  |  |  |  |  |  |  |  |  |  |  |  | Medium | TENmLLDK | 1 | M4(Oxidation) | 0,0000 | 1,92 | 2 | 979,47612 | -0,41 | 38,64 |
|  |  |  |  |  |  |  |  |  |  |  |  |  |  |  |  |  | Medium | QDWEIDPAK | 2 |  | 0,0000 | 1,82 | 2 | 1101,52129 | 0,11 | 44,67 |
|  |  |  |  |  |  |  |  |  |  |  |  |  |  |  |  |  | Medium | QNLRPEIPR | 2 |  | 0,0000 | 1,49 | 3 | 1122,63800 | 0,12 | 38,51 |
|  |  |  |  |  |  |  |  |  |  |  |  |  |  |  |  |  | Medium | ccPSSLANVmK | 1 | C1(Carbamidomethyl); C2(Carbamidomethyl); M10(Oxidation) | 0,0000 | 1,47 | 2 | 1282,55864 | -0,21 | 38,95 |
|  |  |  |  |  |  |  |  |  |  |  |  |  |  |  |  |  | Medium | RcWDANPDK | 1 | C2(Carbamidomethyl) | 0,0000 | 1,42 | 2 | 1161,51128 | 0,56 | 35,79 |
| **7511** | M5X1V1 | inosine-5'-monophosphate dehydrogenase | Inosine-5'-monophosphate dehydrogenase OS=Prunus persica GN=PRUPE_ppa004597mg PE=3 SV=1 - [M5X1V1_PRUPE] |  | Prunus persica | Chloroplast | 01.03-Metabolism/Nucleotides | 10,41 | 7,19 | 3 | 4 | 4 | 7 | 501 | 53,7 | 6,86 | High | DDEVVDVVAK | 2 |  | 0,0000 | 2,64 | 2 | 1088,54668 | -0,35 | 43,94 |
|  |  |  |  |  |  |  |  |  |  |  |  |  |  |  |  |  | Medium | IAQGVVGAVADK | 1 |  | 0,0000 | 1,85 | 2 | 1127,64189 | -0,07 | 40,35 |
|  |  |  |  |  |  |  |  |  |  |  |  |  |  |  |  |  | Medium | LLGYVAGR | 2 |  | 0,0000 | 1,73 | 2 | 848,49870 | -0,23 | 42,47 |
|  |  |  |  |  |  |  |  |  |  |  |  |  |  |  |  |  | Medium | LEGLVK | 2 |  | 0,0000 | 1,09 | 2 | 658,41332 | -0,19 | 39,59 |
| **7609** | M5X7M3 | dihydropyrimidinase | Uncharacterized protein OS=Prunus persica GN=PRUPE_ppa003789mg PE=4 SV=1 - [M5X7M3_PRUPE] | 95% to GI:645241843 Prunus mume, E value 0 | Prunus mume | Chloroplast | 01.01-Metabolism/Amino Acid | 26,79 | 15,88 | 3 | 10 | 10 | 19 | 548 | 59,5 | 6,54 | High | VGDDVTVLDATGK | 3 |  | 0,0000 | 4,32 | 2 | 1289,65862 | 0,16 | 43,64 |
|  |  |  |  |  |  |  |  |  |  |  |  |  |  |  |  |  | High | IPNGVNGIEER | 1 |  | 0,0000 | 2,92 | 2 | 1197,62187 | -0,35 | 39,89 |
|  |  |  |  |  |  |  |  |  |  |  |  |  |  |  |  |  | High | IPNGVnGIEER | 2 | N6(Deamidated) | 0,0000 | 2,90 | 2 | 1198,60771 | 1,17 | 40,24 |
|  |  |  |  |  |  |  |  |  |  |  |  |  |  |  |  |  | Medium | ALGIDDFR | 1 |  | 0,0000 | 1,99 | 2 | 906,46745 | -0,61 | 45,16 |
|  |  |  |  |  |  |  |  |  |  |  |  |  |  |  |  |  | Medium | EmEIMVNEK | 1 | M2(Oxidation) | 0,0000 | 1,78 | 2 | 1138,51323 | 1,13 | 38,76 |
|  |  |  |  |  |  |  |  |  |  |  |  |  |  |  |  |  | Medium | YVMSPPIR | 3 |  | 0,0000 | 1,77 | 2 | 962,51262 | -0,23 | 41,48 |
|  |  |  |  |  |  |  |  |  |  |  |  |  |  |  |  |  | Medium | FFMAYK | 2 |  | 0,0000 | 1,72 | 2 | 806,39043 | -0,20 | 44,48 |
|  |  |  |  |  |  |  |  |  |  |  |  |  |  |  |  |  | Medium | SDTNVFEGWR | 1 |  | 0,0000 | 1,66 | 2 | 1210,54912 | 0,28 | 46,24 |
|  |  |  |  |  |  |  |  |  |  |  |  |  |  |  |  |  | Medium | VVWLNDELK | 1 |  | 0,0000 | 1,66 | 2 | 1115,60893 | -0,59 | 46,29 |
|  |  |  |  |  |  |  |  |  |  |  |  |  |  |  |  |  | Medium | WDEVVSK | 2 |  | 0,0000 | 1,52 | 2 | 862,43041 | -0,17 | 39,02 |
|  |  |  |  |  |  |  |  |  |  |  |  |  |  |  |  |  | Medium | YVmSPPIR | 1 | M3(Oxidation) | 0,1040 | 1,12 | 2 | 978,50749 | -0,27 | 39,78 |
|  |  |  |  |  |  |  |  |  |  |  |  |  |  |  |  |  | Medium | YLSSLK | 1 |  | 0,0000 | 0,99 | 2 | 710,40843 | 0,14 | 38,02 |
| **7702** | M5WGX8 | peptidylprolyl isomerase domain and WD repeat-containing protein | Uncharacterized protein OS=Prunus persica GN=PRUPE_ppa003495mg PE=4 SV=1 - [M5WGX8_PRUPE] | 99% to GI:645217828 Prunus mume, E value 0 | Prunus mume | Chloroplast | 06.01-Protein destination and storage/Folding and stability | 12,47 | 13,51 | 3 | 8 | 8 | 11 | 570 | 64,3 | 7,25 | High | GMDVVQAIEK | 1 |  | 0,0000 | 2,52 | 2 | 1089,56072 | -0,21 | 43,91 |
|  |  |  |  |  |  |  |  |  |  |  |  |  |  |  |  |  | High | AGTNEPIISR | 2 |  | 0,0000 | 2,09 | 2 | 1057,56352 | -0,17 | 37,84 |
|  |  |  |  |  |  |  |  |  |  |  |  |  |  |  |  |  | Medium | KIPAAAANVNESK | 1 |  | 0,0000 | 2,03 | 3 | 1312,72281 | 0,61 | 35,17 |
|  |  |  |  |  |  |  |  |  |  |  |  |  |  |  |  |  | Medium | TTVSSIEVSPDGK | 2 |  | 0,0000 | 1,89 | 2 | 1319,66838 | -0,44 | 40,04 |
|  |  |  |  |  |  |  |  |  |  |  |  |  |  |  |  |  | Medium | IALYQGDR | 1 |  | 0,0000 | 1,62 | 2 | 935,49407 | -0,51 | 39,80 |
|  |  |  |  |  |  |  |  |  |  |  |  |  |  |  |  |  | Medium | QFSITSPDR | 1 |  | 0,0000 | 1,26 | 2 | 1050,52214 | 0,62 | 41,29 |
|  |  |  |  |  |  |  |  |  |  |  |  |  |  |  |  |  | Medium | LAISDR | 1 |  | 0,0000 | 1,15 | 2 | 674,38329 | 0,13 | 36,98 |
|  |  |  |  |  |  |  |  |  |  |  |  |  |  |  |  |  | Medium | ILNVTVPK | 2 |  | 0,0000 | 1,01 | 2 | 883,56096 | -0,24 | 43,22 |
| **7704** | M5WFB9 | 5-methyltetrahydropteroyltriglutamate-homocysteine methyltransferase | Uncharacterized protein OS=Prunus persica GN=PRUPE_ppa001783mg PE=3 SV=1 - [M5WFB9_PRUPE] | 98% to GI:645263470 Prunus mume, E value 0 | Prunus persica | Chloroplast | 01.01-Metabolism/Amino Acid | 15,52 | 11,50 | 1 | 11 | 11 | 14 | 765 | 84,6 | 6,55 | High | IPSTEEIADR | 1 |  | 0,0000 | 2,99 | 2 | 1130,56914 | 0,25 | 40,42 |
|  |  |  |  |  |  |  |  |  |  |  |  |  |  |  |  |  | High | GNASVPAMEMTK | 2 |  | 0,0000 | 2,45 | 2 | 1235,57573 | -0,18 | 42,47 |
|  |  |  |  |  |  |  |  |  |  |  |  |  |  |  |  |  | High | YLFAGLVDGR | 1 |  | 0,0000 | 2,39 | 2 | 1110,59490 | 0,56 | 49,92 |
|  |  |  |  |  |  |  |  |  |  |  |  |  |  |  |  |  | High | IVEVNALAK | 2 |  | 0,0000 | 2,06 | 2 | 956,57726 | -0,32 | 43,05 |
|  |  |  |  |  |  |  |  |  |  |  |  |  |  |  |  |  | Medium | GVTAYGFDLVR | 1 |  | 0,0000 | 1,94 | 2 | 1197,62663 | 0,28 | 48,55 |
|  |  |  |  |  |  |  |  |  |  |  |  |  |  |  |  |  | Medium | ISEEEYVK | 1 |  | 0,0000 | 1,82 | 2 | 996,48815 | -0,31 | 39,46 |
|  |  |  |  |  |  |  |  |  |  |  |  |  |  |  |  |  | Medium | KISEEEYVK | 1 |  | 0,0000 | 1,46 | 2 | 1124,58330 | -0,11 | 37,02 |
|  |  |  |  |  |  |  |  |  |  |  |  |  |  |  |  |  | Medium | EEISNVVK | 1 |  | 0,0000 | 1,43 | 2 | 917,49321 | -0,73 | 39,50 |
|  |  |  |  |  |  |  |  |  |  |  |  |  |  |  |  |  | Medium | LLSVFR | 1 |  | 0,0000 | 1,39 | 2 | 734,45592 | -0,07 | 46,00 |
|  |  |  |  |  |  |  |  |  |  |  |  |  |  |  |  |  | Medium | EVISELK | 1 |  | 0,0000 | 1,37 | 2 | 817,46623 | -0,45 | 41,11 |
|  |  |  |  |  |  |  |  |  |  |  |  |  |  |  |  |  | Medium | TLDLIK | 2 |  | 0,0000 | 1,31 | 2 | 702,43993 | 0,39 | 45,04 |
| **7707** | M5XQJ0 | Phosphoenolpyruvate carboxykinase [ATP] | Uncharacterized protein OS=Prunus persica GN=PRUPE_ppa002490mg PE=3 SV=1 - [M5XQJ0_PRUPE] | 99% to GI:645224569 Prunus mume, E value 0 | Prunus mume | Cytoplasm | 02.02-Energy/Gluconeogenesis | 78,14 | 27,89 | 2 | 15 | 16 | 54 | 667 | 74,0 | 7,23 | High | AAEnGGFSFTR | 3 | N4(Deamidated) | 0,0000 | 3,47 | 2 | 1157,52434 | 1,82 | 44,14 |
|  |  |  |  |  |  |  |  |  |  |  |  |  |  |  |  |  | High | TIDELHSLQK | 2 |  | 0,0000 | 3,39 | 2 | 1183,63164 | -0,11 | 39,83 |
|  |  |  |  |  |  |  |  |  |  |  |  |  |  |  |  |  | High | SSQPTTPInGK | 4 | N9(Deamidated) | 0,0000 | 3,28 | 2 | 1130,56938 | 0,48 | 37,13 |
|  |  |  |  |  |  |  |  |  |  |  |  |  |  |  |  |  | High | EMVILGTQYAGEmK | 2 | M13(Oxidation) | 0,0000 | 3,20 | 2 | 1585,76091 | 0,50 | 46,35 |
|  |  |  |  |  |  |  |  |  |  |  |  |  |  |  |  |  | High | AAENGGFSFTR | 1 |  | 0,0000 | 3,20 | 2 | 1156,53838 | 0,14 | 43,09 |
|  |  |  |  |  |  |  |  |  |  |  |  |  |  |  |  |  | High | EMVILGTQYAGEMK | 2 |  | 0,0000 | 3,04 | 2 | 1569,76641 | 0,76 | 48,57 |
|  |  |  |  |  |  |  |  |  |  |  |  |  |  |  |  |  | High | AVDYLNSLDK | 4 |  | 0,0000 | 3,01 | 2 | 1137,57878 | 0,10 | 46,09 |
|  |  |  |  |  |  |  |  |  |  |  |  |  |  |  |  |  | High | DEETENELWWGK | 2 |  | 0,0000 | 2,83 | 2 | 1535,66557 | 0,41 | 50,75 |
|  |  |  |  |  |  |  |  |  |  |  |  |  |  |  |  |  | High | QGSFAAISEEDR | 5 |  | 0,0000 | 2,77 | 2 | 1309,60234 | 0,30 | 43,83 |
|  |  |  |  |  |  |  |  |  |  |  |  |  |  |  |  |  | High | IIDAIHSGR | 5 |  | 0,0000 | 2,48 | 2 | 981,54723 | -0,43 | 38,54 |
|  |  |  |  |  |  |  |  |  |  |  |  |  |  |  |  |  | High | LLNASYTK | 2 |  | 0,0000 | 2,39 | 2 | 909,50365 | -0,42 | 40,03 |
|  |  |  |  |  |  |  |  |  |  |  |  |  |  |  |  |  | High | YAAMLSEK | 2 |  | 0,0000 | 2,33 | 2 | 912,44902 | -0,60 | 40,23 |
|  |  |  |  |  |  |  |  |  |  |  |  |  |  |  |  |  | High | AAYPIEYIPNAK | 3 |  | 0,0000 | 2,31 | 2 | 1349,71062 | 0,44 | 46,79 |
|  |  |  |  |  |  |  |  |  |  |  |  |  |  |  |  |  | High | YAAmLSEK | 3 | M4(Oxidation) | 0,0000 | 2,16 | 2 | 928,44438 | -0,11 | 37,39 |
|  |  |  |  |  |  |  |  |  |  |  |  |  |  |  |  |  | Medium | IQTHPEATSEVcHDDSGTPVK | 3 | C12(Carbamidomethyl) | 0,0000 | 2,05 | 4 | 2308,05092 | -0,08 | 38,16 |
|  |  |  |  |  |  |  |  |  |  |  |  |  |  |  |  |  | Medium | AVDYLnSLDK | 1 | N6(Deamidated) | 0,0000 | 1,84 | 2 | 1138,56291 | 0,20 | 44,61 |
|  |  |  |  |  |  |  |  |  |  |  |  |  |  |  |  |  | Medium | IPcVGPHPK | 3 | C3(Carbamidomethyl) | 0,0000 | 1,64 | 3 | 1004,53443 | -0,22 | 38,91 |
|  |  |  |  |  |  |  |  |  |  |  |  |  |  |  |  |  | Medium | DEETEnELWWGK | 1 | N6(Deamidated) | 0,0000 | 1,60 | 2 | 1536,64336 | -3,65 | 51,51 |
|  |  |  |  |  |  |  |  |  |  |  |  |  |  |  |  |  | Medium | EVDYSEKSVTENTR | 1 |  | 0,0000 | 1,55 | 3 | 1656,77085 | -0,21 | 38,21 |
|  |  |  |  |  |  |  |  |  |  |  |  |  |  |  |  |  | Medium | LTEEILAAGPNF | 2 |  | 0,0000 | 1,42 | 2 | 1274,66313 | 0,29 | 52,75 |
|  |  |  |  |  |  |  |  |  |  |  |  |  |  |  |  |  | Medium | QILSLHSGcNmGK | 1 | C9(Carbamidomethyl); M11(Oxidation) | 0,0000 | 1,30 | 3 | 1460,69794 | -0,39 | 38,75 |
|  |  |  |  |  |  |  |  |  |  |  |  |  |  |  |  |  | Medium | QILSLHSGcNMGK | 2 | C9(Carbamidomethyl) | 0,0000 | 1,20 | 3 | 1444,70285 | -0,51 | 41,11 |
| **7709** | M5XQJ0 | Phosphoenolpyruvate carboxykinase [ATP] | Uncharacterized protein OS=Prunus persica GN=PRUPE_ppa002490mg PE=3 SV=1 - [M5XQJ0_PRUPE] | 99% to GI:645224569 Prunus mume, E value 0 | Prunus persica | Cytoplasm | 02.02-Energy/Gluconeogenesis | 137,58 | 40,93 | 3 | 23 | 23 | 82 | 667 | 74,0 | 7,23 | High | GSFITATGALATLSGAK | 3 |  | 0,0000 | 4,01 | 2 | 1565,85478 | 0,86 | 50,58 |
|  |  |  |  |  |  |  |  |  |  |  |  |  |  |  |  |  | High | EMVILGTQYAGEMK | 3 |  | 0,0000 | 3,82 | 2 | 1569,76677 | 0,99 | 48,56 |
|  |  |  |  |  |  |  |  |  |  |  |  |  |  |  |  |  | High | DGDVALFFGLSGTGK | 2 |  | 0,0000 | 3,52 | 2 | 1483,74394 | 0,76 | 53,37 |
|  |  |  |  |  |  |  |  |  |  |  |  |  |  |  |  |  | High | IQTHPEATSEVcHDDSGTPVK | 3 | C12(Carbamidomethyl) | 0,0000 | 3,45 | 3 | 2308,05210 | 0,43 | 45,93 |
|  |  |  |  |  |  |  |  |  |  |  |  |  |  |  |  |  | High | EMVILGTQYAGEmK | 4 | M13(Oxidation) | 0,0000 | 3,07 | 2 | 1585,76140 | 0,80 | 47,20 |
|  |  |  |  |  |  |  |  |  |  |  |  |  |  |  |  |  | High | AVDYLnSLDK | 1 | N6(Deamidated) | 0,0000 | 3,00 | 2 | 1138,56401 | 1,16 | 44,84 |
|  |  |  |  |  |  |  |  |  |  |  |  |  |  |  |  |  | High | DEETENELWWGK | 2 |  | 0,0000 | 2,87 | 2 | 1535,66631 | 0,88 | 50,15 |
|  |  |  |  |  |  |  |  |  |  |  |  |  |  |  |  |  | High | YAAMLSEK | 4 |  | 0,0000 | 2,70 | 2 | 912,44908 | -0,53 | 40,15 |
|  |  |  |  |  |  |  |  |  |  |  |  |  |  |  |  |  | High | AVDYLNSLDK | 5 |  | 0,0000 | 2,53 | 2 | 1137,57866 | -0,01 | 45,30 |
|  |  |  |  |  |  |  |  |  |  |  |  |  |  |  |  |  | High | DKEPDIWNAIK | 1 |  | 0,0000 | 2,46 | 2 | 1328,68437 | -0,14 | 48,04 |
|  |  |  |  |  |  |  |  |  |  |  |  |  |  |  |  |  | High | AAYPIEYIPNAK | 4 |  | 0,0000 | 2,44 | 2 | 1349,71062 | 0,44 | 47,98 |
|  |  |  |  |  |  |  |  |  |  |  |  |  |  |  |  |  | High | QGSFAAISEEDR | 5 |  | 0,0000 | 2,40 | 2 | 1309,60234 | 0,30 | 41,74 |
|  |  |  |  |  |  |  |  |  |  |  |  |  |  |  |  |  | High | SSQPTTPInGK | 6 | N9(Deamidated) | 0,0000 | 2,38 | 2 | 1130,56828 | -0,49 | 37,99 |
|  |  |  |  |  |  |  |  |  |  |  |  |  |  |  |  |  | High | TIDELHSLQK | 2 |  | 0,0000 | 2,37 | 2 | 1183,63164 | -0,11 | 45,76 |
|  |  |  |  |  |  |  |  |  |  |  |  |  |  |  |  |  | High | LLNASYTK | 4 |  | 0,0000 | 2,36 | 2 | 909,50383 | -0,22 | 37,97 |
|  |  |  |  |  |  |  |  |  |  |  |  |  |  |  |  |  | High | QQLQSISASLASLTR | 2 |  | 0,0000 | 2,34 | 2 | 1602,88103 | 0,00 | 48,88 |
|  |  |  |  |  |  |  |  |  |  |  |  |  |  |  |  |  | High | YAAmLSEK | 5 | M4(Oxidation) | 0,0000 | 2,23 | 2 | 928,44408 | -0,44 | 36,64 |
|  |  |  |  |  |  |  |  |  |  |  |  |  |  |  |  |  | Medium | NVILLAcDAFGVLPPVSK | 2 | C7(Carbamidomethyl) | 0,0000 | 1,90 | 2 | 1913,05974 | 1,65 | 52,36 |
|  |  |  |  |  |  |  |  |  |  |  |  |  |  |  |  |  | Medium | DTLLQLGGLFK | 2 |  | 0,0000 | 1,88 | 2 | 1204,69390 | 0,19 | 52,81 |
|  |  |  |  |  |  |  |  |  |  |  |  |  |  |  |  |  | Medium | LTEEILAAGPNF | 3 |  | 0,0000 | 1,69 | 2 | 1274,66313 | 0,29 | 52,86 |
|  |  |  |  |  |  |  |  |  |  |  |  |  |  |  |  |  | Medium | GSPNIEMDEHTFLVNR | 1 |  | 0,0000 | 1,61 | 3 | 1858,87644 | 0,61 | 47,39 |
|  |  |  |  |  |  |  |  |  |  |  |  |  |  |  |  |  | Medium | IPcVGPHPK | 5 | C3(Carbamidomethyl) | 0,0000 | 1,58 | 3 | 1004,53452 | -0,13 | 36,66 |
|  |  |  |  |  |  |  |  |  |  |  |  |  |  |  |  |  | Medium | cIDLSK | 3 | C1(Carbamidomethyl) | 0,0000 | 1,57 | 2 | 735,37047 | -0,16 | 39,38 |
|  |  |  |  |  |  |  |  |  |  |  |  |  |  |  |  |  | Medium | IIDAIHSGR | 3 |  | 0,0000 | 1,44 | 3 | 981,54792 | 0,27 | 36,48 |
|  |  |  |  |  |  |  |  |  |  |  |  |  |  |  |  |  | Medium | EVDYSEK | 2 |  | 0,0000 | 1,27 | 2 | 869,38860 | -0,15 | 35,80 |
|  |  |  |  |  |  |  |  |  |  |  |  |  |  |  |  |  | Medium | TTLSTDHNR | 4 |  | 0,0000 | 1,22 | 3 | 1044,50718 | 0,27 | 42,63 |
|  |  |  |  |  |  |  |  |  |  |  |  |  |  |  |  |  | Medium | SSQPTTPINGK | 1 |  | 0,0000 | 0,92 | 2 | 1129,58476 | -0,05 | 35,45 |
| **7716** | M5X5I9 | acetyl-coenzyme A carboxylase carboxyl transferase subunit alpha | Uncharacterized protein OS=Prunus persica GN=PRUPE_ppa001950mg PE=3 SV=1 - [M5X5I9_PRUPE] | 99% to GI:645253901 Prunus mume, E value 0 | Prunus persica | Chloroplast | 01.06-Metabolism/Lipid and sterol | 18,85 | 11,80 | 2 | 9 | 9 | 13 | 737 | 82,6 | 7,69 | High | VINEAGISGR | 2 |  | 0,0000 | 3,22 | 2 | 1015,55284 | -0,29 | 38,51 |
|  |  |  |  |  |  |  |  |  |  |  |  |  |  |  |  |  | High | GPSDPITTQAIEK | 1 |  | 0,0000 | 2,74 | 2 | 1356,70098 | 0,28 | 40,59 |
|  |  |  |  |  |  |  |  |  |  |  |  |  |  |  |  |  | High | NVVTAPPELK | 1 |  | 0,0000 | 2,12 | 2 | 1067,60942 | -0,16 | 40,77 |
|  |  |  |  |  |  |  |  |  |  |  |  |  |  |  |  |  | High | SANLEVVGVTK | 2 |  | 0,0000 | 2,07 | 2 | 1116,62602 | 0,05 | 41,20 |
|  |  |  |  |  |  |  |  |  |  |  |  |  |  |  |  |  | Medium | LNLVSR | 2 |  | 0,0000 | 1,86 | 2 | 701,43035 | -0,19 | 38,97 |
|  |  |  |  |  |  |  |  |  |  |  |  |  |  |  |  |  | Medium | ELMEEVEAVK | 1 |  | 0,0000 | 1,58 | 2 | 1176,58098 | -0,65 | 43,80 |
|  |  |  |  |  |  |  |  |  |  |  |  |  |  |  |  |  | Medium | SIGGFQEGIPVEPK | 1 |  | 0,0000 | 1,32 | 2 | 1457,76360 | 0,03 | 45,12 |
|  |  |  |  |  |  |  |  |  |  |  |  |  |  |  |  |  | Medium | TMFGLK | 2 |  | 0,0000 | 1,20 | 2 | 696,37480 | -0,21 | 43,13 |
|  |  |  |  |  |  |  |  |  |  |  |  |  |  |  |  |  | Medium | HLTPIQR | 1 |  | 0,0000 | 1,14 | 2 | 864,50493 | -0,13 | 34,99 |
| **7727** | M5VGU2 | cullin | Uncharacterized protein OS=Prunus persica GN=PRUPE_ppa001948mg PE=3 SV=1 - [M5VGU2_PRUPE] | 99% to GI:645258714 Prunus mume, E value 0 | Prunus mume | Cytoplasm | 06.07-Protein destination and storage/Modification | 43,84 | 17,21 | 4 | 14 | 14 | 27 | 738 | 85,8 | 6,90 | High | LSDEAIEETLEK | 2 |  | 0,0000 | 3,62 | 2 | 1376,68022 | 0,74 | 45,69 |
|  |  |  |  |  |  |  |  |  |  |  |  |  |  |  |  |  | High | YAIDAAIVR | 3 |  | 0,0000 | 3,08 | 2 | 991,55675 | -0,41 | 45,10 |
|  |  |  |  |  |  |  |  |  |  |  |  |  |  |  |  |  | High | DDKVEDLSR | 3 |  | 0,0000 | 2,65 | 3 | 1076,52170 | -0,19 | 37,24 |
|  |  |  |  |  |  |  |  |  |  |  |  |  |  |  |  |  | High | GLEPVSSVFK | 2 |  | 0,0000 | 2,29 | 2 | 1062,58269 | -0,33 | 45,80 |
|  |  |  |  |  |  |  |  |  |  |  |  |  |  |  |  |  | High | IEDLITR | 1 |  | 0,0000 | 2,23 | 2 | 859,48833 | -0,08 | 42,25 |
|  |  |  |  |  |  |  |  |  |  |  |  |  |  |  |  |  | High | EAFEVFcNK | 2 | C7(Carbamidomethyl) | 0,0000 | 2,16 | 2 | 1143,51372 | -0,22 | 46,13 |
|  |  |  |  |  |  |  |  |  |  |  |  |  |  |  |  |  | Medium | LLHSLScAK | 2 | C7(Carbamidomethyl) | 0,0000 | 2,12 | 3 | 1028,55585 | 0,08 | 36,59 |
|  |  |  |  |  |  |  |  |  |  |  |  |  |  |  |  |  | Medium | DKENPNMFK | 2 |  | 0,0000 | 2,11 | 3 | 1122,52466 | -0,20 | 37,25 |
|  |  |  |  |  |  |  |  |  |  |  |  |  |  |  |  |  | Medium | TISPTDSFEFNSK | 2 |  | 0,0000 | 1,98 | 2 | 1472,69145 | 0,70 | 46,14 |
|  |  |  |  |  |  |  |  |  |  |  |  |  |  |  |  |  | Medium | VAVIGLIDK | 1 |  | 0,0000 | 1,83 | 2 | 927,58702 | -0,41 | 46,41 |
|  |  |  |  |  |  |  |  |  |  |  |  |  |  |  |  |  | Medium | VIEDVDKDR | 4 |  | 0,0000 | 1,65 | 2 | 1088,55803 | -0,24 | 35,61 |
|  |  |  |  |  |  |  |  |  |  |  |  |  |  |  |  |  | Medium | cVEVFK | 1 | C1(Carbamidomethyl) | 0,0000 | 1,50 | 2 | 781,39122 | -0,13 | 40,14 |
|  |  |  |  |  |  |  |  |  |  |  |  |  |  |  |  |  | Medium | ILIKEPNTK | 1 |  | 0,0000 | 1,15 | 2 | 1055,64726 | 1,22 | 37,89 |
|  |  |  |  |  |  |  |  |  |  |  |  |  |  |  |  |  | Medium | MFKPDIK | 1 |  | 0,1261 | 1,04 | 2 | 878,47978 | -0,81 | 39,20 |
| **7812** | M5WU88 | Sucrose synthase | Sucrose synthase OS=Prunus persica GN=PRUPE_ppa017606mg PE=3 SV=1 - [M5WU88_PRUPE] |  | Prunus persica | Chloroplast | 01.05-Metabolism/Sugars and polysaccharides | 11,08 | 7,08 | 2 | 6 | 7 | 12 | 833 | 94,7 | 6,99 | High | cNQELEPInGTK | 2 | C1(Carbamidomethyl); N9(Deamidated) | 0,0000 | 2,02 | 2 | 1403,64873 | 1,10 | 40,30 |
|  |  |  |  |  |  |  |  |  |  |  |  |  |  |  |  |  | Medium | TDATYWDR | 1 |  | 0,0000 | 1,91 | 2 | 1027,44780 | -0,17 | 40,80 |
|  |  |  |  |  |  |  |  |  |  |  |  |  |  |  |  |  | Medium | YSNILR | 1 |  | 0,0000 | 1,88 | 2 | 765,42522 | -0,21 | 39,04 |
|  |  |  |  |  |  |  |  |  |  |  |  |  |  |  |  |  | Medium | cNQELEPINGTK | 1 | C1(Carbamidomethyl) | 0,0000 | 1,75 | 2 | 1402,66399 | 0,59 | 39,23 |
|  |  |  |  |  |  |  |  |  |  |  |  |  |  |  |  |  | Medium | WIAAQTDR | 2 |  | 0,0000 | 1,69 | 2 | 960,48955 | -0,26 | 38,59 |
|  |  |  |  |  |  |  |  |  |  |  |  |  |  |  |  |  | Medium | VVSGINVFDPK | 2 |  | 0,0000 | 1,54 | 2 | 1174,64763 | 0,78 | 45,39 |
|  |  |  |  |  |  |  |  |  |  |  |  |  |  |  |  |  | Medium | IADFFEK | 2 |  | 0,0000 | 0,91 | 2 | 869,43974 | -0,74 | 45,67 |
| **8004** | M5XA69 | Universal stress protein | Uncharacterized protein OS=Prunus persica GN=PRUPE_ppb010253mg PE=4 SV=1 - [M5XA69_PRUPE] | 98% to GI:645276352 Prunus mume, E value 2e-122 | Prunus mume | Cytoplasm | 11.05-Disease/Defense/Stress responses | 45,51 | 49,71 | 1 | 7 | 10 | 30 | 175 | 18,7 | 7,77 | High | AILGSVSNYcVQNAK | 3 | C10(Carbamidomethyl) | 0,0000 | 4,23 | 2 | 1623,81731 | 0,82 | 44,64 |
|  |  |  |  |  |  |  |  |  |  |  |  |  |  |  |  |  | High | VVTEIGDPK | 10 |  | 0,0000 | 2,82 | 2 | 957,52489 | -0,32 | 38,71 |
|  |  |  |  |  |  |  |  |  |  |  |  |  |  |  |  |  | High | cPVLVVK | 4 | C1(Carbamidomethyl) | 0,0000 | 2,55 | 2 | 814,48534 | -0,28 | 39,59 |
|  |  |  |  |  |  |  |  |  |  |  |  |  |  |  |  |  | High | TAIcDAVEK | 3 | C4(Carbamidomethyl) | 0,0000 | 2,07 | 2 | 1006,48693 | -0,50 | 38,72 |
|  |  |  |  |  |  |  |  |  |  |  |  |  |  |  |  |  | Medium | FAVALLEK | 2 |  | 0,0000 | 1,75 | 2 | 890,53417 | -0,52 | 45,95 |
|  |  |  |  |  |  |  |  |  |  |  |  |  |  |  |  |  | Medium | KFAVALLEK | 2 |  | 0,0000 | 1,33 | 2 | 1018,62932 | -0,28 | 42,58 |
|  |  |  |  |  |  |  |  |  |  |  |  |  |  |  |  |  | Medium | cPVLVVKK | 1 | C1(Carbamidomethyl) | 0,0917 | 1,09 | 2 | 942,58068 | 0,15 | 36,01 |
| **8005** | M5WHT9 | acyl-coenzyme A thioesterase | Uncharacterized protein OS=Prunus persica GN=PRUPE_ppa012630mg PE=4 SV=1 - [M5WHT9_PRUPE] | 97% to GI:645238521 Prunus mume, E value 8e-106 | Prunus persica | Cytoplasm | 20.1-Secondary metabolism/Phenylpropanoids/Phenolics | 13,42 | 38,13 | 5 | 7 | 7 | 18 | 160 | 17,1 | 8,09 | High | AVGVASVELR | 2 |  | 0,0000 | 3,10 | 2 | 1000,57805 | -0,58 | 42,65 |
|  |  |  |  |  |  |  |  |  |  |  |  |  |  |  |  |  | High | EGNEETSMESVK | 3 |  | 0,0000 | 2,39 | 2 | 1339,56902 | 0,56 | 37,83 |
|  |  |  |  |  |  |  |  |  |  |  |  |  |  |  |  |  | Medium | YLALASK | 3 |  | 0,0000 | 1,94 | 2 | 765,45067 | 0,17 | 40,39 |
|  |  |  |  |  |  |  |  |  |  |  |  |  |  |  |  |  | Medium | EGEMAISMDGLPDK | 1 |  | 0,0000 | 1,91 | 2 | 1492,66667 | 0,51 | 45,88 |
|  |  |  |  |  |  |  |  |  |  |  |  |  |  |  |  |  | Medium | GIQVDLLEPGR | 1 |  | 0,0000 | 1,44 | 2 | 1196,66374 | 0,26 | 46,81 |
|  |  |  |  |  |  |  |  |  |  |  |  |  |  |  |  |  | Medium | EGNEETSmESVK | 2 | M8(Oxidation) | 0,0000 | 1,12 | 2 | 1355,56316 | -0,02 | 37,22 |
|  |  |  |  |  |  |  |  |  |  |  |  |  |  |  |  |  | Medium | IVcSFK | 3 | C3(Carbamidomethyl) | 0,0000 | 1,11 | 2 | 753,39641 | 0,01 | 38,39 |
|  |  |  |  |  |  |  |  |  |  |  |  |  |  |  |  |  | Medium | EGNEETSMESVKK | 1 |  | 0,0000 | 1,11 | 2 | 1467,66277 | -0,32 | 34,78 |
|  |  |  |  |  |  |  |  |  |  |  |  |  |  |  |  |  | Medium | EGEmAISMDGLPDK | 2 | M4(Oxidation) | 0,0000 | 1,02 | 2 | 1508,66301 | 1,45 | 43,67 |
| **8006** | M5W5R5 | CBS domain-containing protein | Uncharacterized protein OS=Prunus persica GN=PRUPE_ppa011584mg PE=4 SV=1 - [M5W5R5_PRUPE] | 94% to GI:657985083 Malus domestica, E value 2e-139 | Prunus mume | Mitochondrion | 11.05-Disease/Defense/Stress responses | 41,78 | 34,15 | 2 | 8 | 8 | 27 | 205 | 22,7 | 9,17 | High | VGDIMTEENK | 3 |  | 0,0000 | 3,33 | 2 | 1135,52971 | -0,28 | 39,51 |
|  |  |  |  |  |  |  |  |  |  |  |  |  |  |  |  |  | High | AMQLMTDNR | 2 |  | 0,0000 | 2,60 | 2 | 1079,49748 | 0,18 | 40,02 |
|  |  |  |  |  |  |  |  |  |  |  |  |  |  |  |  |  | High | AmQLMTDNR | 7 | M2(Oxidation) | 0,0000 | 2,59 | 2 | 1095,49101 | -1,09 | 36,70 |
|  |  |  |  |  |  |  |  |  |  |  |  |  |  |  |  |  | High | IEEHGFESTR | 5 |  | 0,0000 | 2,55 | 3 | 1204,55912 | -0,19 | 36,53 |
|  |  |  |  |  |  |  |  |  |  |  |  |  |  |  |  |  | High | SIAGIITER | 3 |  | 0,0000 | 2,50 | 2 | 959,55193 | -0,15 | 44,47 |
|  |  |  |  |  |  |  |  |  |  |  |  |  |  |  |  |  | High | LITVTPDTK | 3 |  | 0,0000 | 2,04 | 2 | 987,57182 | -0,31 | 40,67 |
|  |  |  |  |  |  |  |  |  |  |  |  |  |  |  |  |  | Medium | IADILK | 1 |  | 0,0000 | 1,79 | 2 | 672,42931 | 0,32 | 41,90 |
|  |  |  |  |  |  |  |  |  |  |  |  |  |  |  |  |  | Medium | LNAYIQGGY | 2 |  | 0,0000 | 1,77 | 2 | 998,49352 | -0,68 | 46,60 |
|  |  |  |  |  |  |  |  |  |  |  |  |  |  |  |  |  | Medium | HIPVIDDR | 1 |  | 0,0000 | 1,17 | 2 | 964,52074 | -0,38 | 40,20 |
| **8122** | M5XQ59 | GAPDH | Uncharacterized protein OS=Prunus persica GN=PRUPE_ppa008227mg PE=3 SV=1 - [M5XQ59_PRUPE] | 99% to GI:645230667 Prunus mume, E value 0 | Prunus persica | Cytosol | 02.01-Energy/Glycolysis | 64,43 | 25,29 | 4 | 9 | 10 | 46 | 340 | 37,0 | 7,52 | High | AASFNIIPSSTGAAK | 3 |  | 0,0000 | 3,38 | 2 | 1434,75969 | 0,63 | 45,10 |
|  |  |  |  |  |  |  |  |  |  |  |  |  |  |  |  |  | High | DAPMFVVGVNEK | 5 |  | 0,0000 | 3,08 | 2 | 1305,65154 | 0,54 | 48,66 |
|  |  |  |  |  |  |  |  |  |  |  |  |  |  |  |  |  | High | AGIALnDNFVK | 2 | N6(Deamidated) | 0,0000 | 3,00 | 2 | 1162,61162 | 1,11 | 45,94 |
|  |  |  |  |  |  |  |  |  |  |  |  |  |  |  |  |  | High | AGIALNDNFVK | 2 |  | 0,0000 | 2,78 | 2 | 1161,62627 | -0,04 | 45,01 |
|  |  |  |  |  |  |  |  |  |  |  |  |  |  |  |  |  | High | KVIISAPSK | 6 |  | 0,0000 | 2,63 | 2 | 942,59770 | -0,62 | 37,52 |
|  |  |  |  |  |  |  |  |  |  |  |  |  |  |  |  |  | High | YDTVHGPWK | 2 |  | 0,0000 | 2,41 | 2 | 1102,53118 | -0,43 | 39,01 |
|  |  |  |  |  |  |  |  |  |  |  |  |  |  |  |  |  | High | IGInGFGR | 3 | N4(Deamidated) | 0,0000 | 2,34 | 2 | 834,44786 | 1,18 | 43,75 |
|  |  |  |  |  |  |  |  |  |  |  |  |  |  |  |  |  | High | DAPmFVVGVNEK | 4 | M4(Oxidation) | 0,0000 | 2,25 | 2 | 1321,64617 | 0,32 | 45,37 |
|  |  |  |  |  |  |  |  |  |  |  |  |  |  |  |  |  | High | IGINGFGR | 1 |  | 0,0000 | 2,09 | 2 | 833,46074 | -2,54 | 42,63 |
|  |  |  |  |  |  |  |  |  |  |  |  |  |  |  |  |  | Medium | SSIFDAK | 4 |  | 0,0000 | 1,71 | 2 | 767,39336 | -0,10 | 43,29 |
|  |  |  |  |  |  |  |  |  |  |  |  |  |  |  |  |  | Medium | LTGmAFR | 4 | M4(Oxidation) | 0,0000 | 1,64 | 2 | 811,41295 | -0,23 | 42,12 |
|  |  |  |  |  |  |  |  |  |  |  |  |  |  |  |  |  | Medium | LTGMAFR | 5 |  | 0,0000 | 1,62 | 2 | 795,41826 | 0,05 | 41,95 |
|  |  |  |  |  |  |  |  |  |  |  |  |  |  |  |  |  | Medium | VIISAPSK | 3 |  | 0,0000 | 1,51 | 2 | 814,50353 | 0,25 | 40,07 |
| **8127** | M5WBP7 | Alpha/beta-Hydrolases superfamily protein | Uncharacterized protein OS=Prunus persica GN=PRUPE_ppa008197mg PE=4 SV=1 - [M5WBP7_PRUPE] | 96% to GI:590586968 Theobroma cacao, E value 0 | Prunus persica | Chloroplast | 06.13-Protein destination and storage/Proteolysis | 30,44 | 22,29 | 1 | 7 | 8 | 15 | 341 | 38,7 | 7,14 | High | AGYGESDPYPSR | 3 |  | 0,0000 | 3,65 | 2 | 1298,56548 | 0,51 | 37,88 |
|  |  |  |  |  |  |  |  |  |  |  |  |  |  |  |  |  | High | SEAFDIQELADK | 1 |  | 0,0000 | 3,30 | 2 | 1365,65361 | 0,21 | 46,73 |
|  |  |  |  |  |  |  |  |  |  |  |  |  |  |  |  |  | High | LSEAPSVGQEK | 2 |  | 0,0000 | 3,20 | 2 | 1144,58489 | 0,34 | 37,48 |
|  |  |  |  |  |  |  |  |  |  |  |  |  |  |  |  |  | High | IcGSPGGPPVTSPR | 3 | C2(Carbamidomethyl) | 0,0000 | 3,19 | 2 | 1381,69036 | 0,74 | 38,95 |
|  |  |  |  |  |  |  |  |  |  |  |  |  |  |  |  |  | High | DILAGYAK | 3 |  | 0,0000 | 2,01 | 2 | 850,46703 | 0,11 | 42,93 |
|  |  |  |  |  |  |  |  |  |  |  |  |  |  |  |  |  | Medium | TDLDMLKK | 1 |  | 0,0000 | 1,50 | 2 | 963,51842 | 0,43 | 39,26 |
|  |  |  |  |  |  |  |  |  |  |  |  |  |  |  |  |  | Medium | IIVVHGFDSSK | 1 |  | 0,0000 | 1,21 | 3 | 1201,65827 | 0,55 | 40,58 |
| **8128** | M5XCW1 | voltage-gated potassium channel subunit beta | Uncharacterized protein OS=Prunus persica GN=PRUPE_ppa008405mg PE=4 SV=1 - [M5XCW1_PRUPE] | 99% to GI:645234796 Prunus mume, E value 0 | Prunus persica | Chloroplast | 07.01-Transporters/Ions | 20,37 | 16,82 | 1 | 6 | 6 | 14 | 333 | 37,0 | 7,39 | High | AEEIMGQAIR | 3 |  | 0,0000 | 3,12 | 2 | 1117,56706 | -0,02 | 42,68 |
|  |  |  |  |  |  |  |  |  |  |  |  |  |  |  |  |  | High | AEEImGQAIR | 2 | M5(Oxidation) | 0,0000 | 3,05 | 2 | 1133,56182 | -0,17 | 38,65 |
|  |  |  |  |  |  |  |  |  |  |  |  |  |  |  |  |  | High | AMNYVIDK | 2 |  | 0,0000 | 2,40 | 2 | 953,47612 | 0,00 | 41,36 |
|  |  |  |  |  |  |  |  |  |  |  |  |  |  |  |  |  | Medium | FALDNYK | 2 |  | 0,0000 | 1,59 | 2 | 870,43602 | 0,46 | 42,03 |
|  |  |  |  |  |  |  |  |  |  |  |  |  |  |  |  |  | Medium | SLLQTcR | 2 | C6(Carbamidomethyl) | 0,0000 | 1,54 | 2 | 877,45610 | 0,06 | 39,24 |
|  |  |  |  |  |  |  |  |  |  |  |  |  |  |  |  |  | Medium | ESQIQENMK | 1 |  | 0,0000 | 1,51 | 2 | 1106,51457 | -0,12 | 36,58 |
|  |  |  |  |  |  |  |  |  |  |  |  |  |  |  |  |  | Medium | AmNYVIDK | 1 | M2(Oxidation) | 0,0136 | 1,45 | 2 | 969,47106 | 0,02 | 39,54 |
|  |  |  |  |  |  |  |  |  |  |  |  |  |  |  |  |  | Medium | AVDVIPLLTPAVMEK | 1 |  | 0,0000 | 1,32 | 2 | 1595,90935 | 0,98 | 51,51 |
| **8131** | M5XWJ9 | mitochondrial outer membrane protein porin | Uncharacterized protein OS=Prunus persica GN=PRUPE_ppa008111mg PE=4 SV=1 - [M5XWJ9_PRUPE] | 99% to GI:645229903 Prunus mume, E value 0 | Prunus persica | Mitochondrion | 07.01-Transporters/Ions | 15,46 | 15,12 | 2 | 6 | 6 | 8 | 344 | 37,8 | 8,75 | High | GDLYLGDVSTQLK | 2 |  | 0,0000 | 3,05 | 2 | 1408,73332 | 1,02 | 47,31 |
|  |  |  |  |  |  |  |  |  |  |  |  |  |  |  |  |  | High | TTITIDEPAPGLK | 2 |  | 0,0000 | 2,92 | 2 | 1355,74260 | 0,63 | 43,33 |
|  |  |  |  |  |  |  |  |  |  |  |  |  |  |  |  |  | Medium | KGDLYLGDVSTQLK | 1 |  | 0,0000 | 2,09 | 3 | 1536,82651 | -0,23 | 43,52 |
|  |  |  |  |  |  |  |  |  |  |  |  |  |  |  |  |  | Medium | AIFSFIVPDQR | 1 |  | 0,0000 | 1,81 | 2 | 1292,69939 | -0,33 | 50,28 |
|  |  |  |  |  |  |  |  |  |  |  |  |  |  |  |  |  | Medium | ARDLLYK | 1 |  | 0,0000 | 1,39 | 2 | 878,50841 | -1,20 | 36,96 |
|  |  |  |  |  |  |  |  |  |  |  |  |  |  |  |  |  | Medium | NITTDVK | 1 |  | 0,0108 | 0,92 | 2 | 790,43047 | -0,09 | 36,05 |
| **8305** | M5WGA9 | Protein DJ-1 homolog | Uncharacterized protein OS=Prunus persica GN=PRUPE_ppa005820mg PE=4 SV=1 - [M5WGA9_PRUPE] | 97% to GI:645240260 Prunus mume, E value 0 | Prunus persica | Chloroplast | 11.05-Disease/Defense/Stress responses | 109,23 | 24,43 | 2 | 13 | 13 | 51 | 442 | 47,2 | 8,68 | High | AGADVTVASVEK | 4 |  | 0,0000 | 3,87 | 2 | 1146,60015 | -0,01 | 38,69 |
|  |  |  |  |  |  |  |  |  |  |  |  |  |  |  |  |  | High | GPGTSMEFALGIVEK | 2 |  | 0,0000 | 3,67 | 2 | 1535,77910 | 1,04 | 49,86 |
|  |  |  |  |  |  |  |  |  |  |  |  |  |  |  |  |  | High | NSEVLESLVK | 3 |  | 0,0000 | 3,14 | 2 | 1117,61016 | 0,16 | 45,53 |
|  |  |  |  |  |  |  |  |  |  |  |  |  |  |  |  |  | High | ADEVSGPLVMR | 7 |  | 0,0000 | 3,11 | 2 | 1173,59307 | -0,21 | 45,10 |
|  |  |  |  |  |  |  |  |  |  |  |  |  |  |  |  |  | High | RAGADVTVASVEK | 4 |  | 0,0000 | 3,09 | 3 | 1302,70081 | -0,36 | 37,46 |
|  |  |  |  |  |  |  |  |  |  |  |  |  |  |  |  |  | High | LEADVLLDEAAK | 2 |  | 0,0000 | 2,82 | 2 | 1286,68450 | 0,46 | 47,63 |
|  |  |  |  |  |  |  |  |  |  |  |  |  |  |  |  |  | High | EKADEVSGPLVmR | 2 | M12(Oxidation) | 0,0000 | 2,81 | 3 | 1446,72553 | -0,19 | 38,10 |
|  |  |  |  |  |  |  |  |  |  |  |  |  |  |  |  |  | High | KATAFPAMcDK | 3 | C9(Carbamidomethyl) | 0,0000 | 2,80 | 3 | 1239,58554 | -0,46 | 37,96 |
|  |  |  |  |  |  |  |  |  |  |  |  |  |  |  |  |  | High | EKADEVSGPLVMR | 3 |  | 0,0000 | 2,68 | 2 | 1430,73198 | 0,77 | 41,25 |
|  |  |  |  |  |  |  |  |  |  |  |  |  |  |  |  |  | High | LSDKSEIENR | 3 |  | 0,0000 | 2,52 | 3 | 1190,60108 | -0,11 | 34,99 |
|  |  |  |  |  |  |  |  |  |  |  |  |  |  |  |  |  | High | VVVDGNLITSR | 1 |  | 0,0000 | 2,51 | 2 | 1172,66374 | 0,28 | 42,31 |
|  |  |  |  |  |  |  |  |  |  |  |  |  |  |  |  |  | High | nSEVLESLVK | 1 | N1(Deamidated) | 0,0000 | 2,36 | 2 | 1118,59404 | 0,04 | 49,39 |
|  |  |  |  |  |  |  |  |  |  |  |  |  |  |  |  |  | High | VMLFVHP | 6 |  | 0,0000 | 2,29 | 2 | 842,45946 | 0,13 | 47,74 |
|  |  |  |  |  |  |  |  |  |  |  |  |  |  |  |  |  | High | GPGTSmEFALGIVEK | 1 | M6(Oxidation) | 0,0000 | 2,02 | 2 | 1551,77324 | 0,53 | 46,29 |
|  |  |  |  |  |  |  |  |  |  |  |  |  |  |  |  |  | Medium | ATAFPAMcDK | 3 | C8(Carbamidomethyl) | 0,0000 | 1,96 | 2 | 1111,49126 | 0,10 | 41,91 |
|  |  |  |  |  |  |  |  |  |  |  |  |  |  |  |  |  | Medium | LVNLLK | 2 |  | 0,0000 | 1,74 | 2 | 699,47655 | 0,25 | 42,52 |
|  |  |  |  |  |  |  |  |  |  |  |  |  |  |  |  |  | Medium | VmLFVHP | 2 | M2(Oxidation) | 0,0000 | 1,61 | 2 | 858,45415 | -0,14 | 46,08 |
|  |  |  |  |  |  |  |  |  |  |  |  |  |  |  |  |  | Medium | ATAFPAmcDK | 2 | M7(Oxidation); C8(Carbamidomethyl) | 0,0000 | 1,21 | 2 | 1127,48650 | 0,39 | 40,81 |
| **8306** | M1QDN1 | Phosphoserine aminotransferase | Phosphoserine aminotransferase OS=Prunus persica PE=2 SV=1 - [M1QDN1_PRUPE] |  | Prunus persica | Chloroplast | 01.01-Metabolism/Amino Acid | 202,48 | 34,42 | 3 | 17 | 17 | 113 | 430 | 47,8 | 8,40 | High | KADLLYNAIDESK | 3 |  | 0,0000 | 5,07 | 2 | 1479,77007 | 0,71 | 43,78 |
|  |  |  |  |  |  |  |  |  |  |  |  |  |  |  |  |  | High | VDYVVTGSWGDK | 3 |  | 0,0000 | 3,92 | 2 | 1325,63823 | 0,73 | 46,45 |
|  |  |  |  |  |  |  |  |  |  |  |  |  |  |  |  |  | High | ADLLYNAIDESK | 3 |  | 0,0000 | 3,70 | 2 | 1351,67461 | 0,42 | 48,01 |
|  |  |  |  |  |  |  |  |  |  |  |  |  |  |  |  |  | High | ASIYNAMPLAGVEK | 8 |  | 0,0000 | 3,68 | 2 | 1463,75762 | 0,86 | 49,22 |
|  |  |  |  |  |  |  |  |  |  |  |  |  |  |  |  |  | High | IPAFEELEQSPDAK | 8 |  | 0,0000 | 3,60 | 2 | 1573,77617 | 1,05 | 49,65 |
|  |  |  |  |  |  |  |  |  |  |  |  |  |  |  |  |  | High | FGIIYAGAQK | 4 |  | 0,0000 | 3,30 | 2 | 1067,58830 | -0,14 | 45,67 |
|  |  |  |  |  |  |  |  |  |  |  |  |  |  |  |  |  | High | ASIYNAmPLAGVEK | 4 | M7(Oxidation) | 0,0000 | 3,25 | 2 | 1479,75102 | -0,16 | 47,90 |
|  |  |  |  |  |  |  |  |  |  |  |  |  |  |  |  |  | High | NVGPSGVTVVIIR | 2 |  | 0,0000 | 3,20 | 2 | 1310,78008 | 0,72 | 47,56 |
|  |  |  |  |  |  |  |  |  |  |  |  |  |  |  |  |  | High | ASIYnAMPLAGVEK | 1 | N5(Deamidated) | 0,0000 | 3,02 | 2 | 1464,74004 | -0,22 | 47,06 |
|  |  |  |  |  |  |  |  |  |  |  |  |  |  |  |  |  | High | SLmNVPFTLEK | 5 | M3(Oxidation) | 0,0000 | 3,00 | 2 | 1294,67107 | -0,11 | 49,84 |
|  |  |  |  |  |  |  |  |  |  |  |  |  |  |  |  |  | High | AQSELYNWR | 10 |  | 0,0000 | 2,83 | 2 | 1166,55925 | 0,27 | 44,70 |
|  |  |  |  |  |  |  |  |  |  |  |  |  |  |  |  |  | High | GKEFLSIIQK | 2 |  | 0,0000 | 2,83 | 3 | 1162,68326 | 0,15 | 45,00 |
|  |  |  |  |  |  |  |  |  |  |  |  |  |  |  |  |  | High | SLMNVPFTLEK | 5 |  | 0,0000 | 2,80 | 2 | 1278,67656 | 0,20 | 50,31 |
|  |  |  |  |  |  |  |  |  |  |  |  |  |  |  |  |  | High | GSGMSVMEMSHR | 5 |  | 0,0000 | 2,48 | 2 | 1308,55034 | 0,70 | 39,94 |
|  |  |  |  |  |  |  |  |  |  |  |  |  |  |  |  |  | High | SELEAEFVK | 8 |  | 0,0000 | 2,25 | 2 | 1051,53093 | 0,25 | 46,04 |
|  |  |  |  |  |  |  |  |  |  |  |  |  |  |  |  |  | High | GSGmSVMEMSHR | 14 | M4(Oxidation) | 0,0000 | 2,01 | 2 | 1324,54460 | 0,20 | 38,65 |
|  |  |  |  |  |  |  |  |  |  |  |  |  |  |  |  |  | Medium | VIWSGKSEK | 1 |  | 0,0000 | 1,87 | 3 | 1033,56800 | 0,27 | 36,88 |
|  |  |  |  |  |  |  |  |  |  |  |  |  |  |  |  |  | Medium | EFLSIIQK | 3 |  | 0,0000 | 1,77 | 2 | 977,56639 | -0,26 | 48,69 |
|  |  |  |  |  |  |  |  |  |  |  |  |  |  |  |  |  | Medium | LVDFMK | 8 |  | 0,0000 | 1,70 | 2 | 752,40141 | 0,32 | 45,58 |
|  |  |  |  |  |  |  |  |  |  |  |  |  |  |  |  |  | Medium | AEADLR | 1 |  | 0,0000 | 1,48 | 2 | 674,34679 | -0,05 | 35,70 |
|  |  |  |  |  |  |  |  |  |  |  |  |  |  |  |  |  | Medium | LVDFmK | 10 | M5(Oxidation) | 0,0000 | 1,46 | 2 | 768,39574 | -0,45 | 47,07 |
|  |  |  |  |  |  |  |  |  |  |  |  |  |  |  |  |  | Medium | VIWSGK | 5 |  | 0,0000 | 1,27 | 2 | 689,39824 | 0,17 | 40,18 |
| **8307** | M5WSM4 | formate dehydrogenase | Uncharacterized protein OS=Prunus persica GN=PRUPE_ppa006791mg PE=3 SV=1 - [M5WSM4_PRUPE] | 98% to GI:645249147 Prunus mume, E value 0 | Prunus mume | Mitochondrion | 11.06-Disease/Defense/Detoxification | 72,20 | 36,88 | 4 | 14 | 14 | 36 | 385 | 42,2 | 7,61 | High | TLASSGSSASSTTFTR | 3 |  | 0,0000 | 4,34 | 2 | 1560,75127 | 0,77 | 39,19 |
|  |  |  |  |  |  |  |  |  |  |  |  |  |  |  |  |  | High | IDPELEQQIGAK | 3 |  | 0,0000 | 3,35 | 2 | 1340,70635 | 0,49 | 42,86 |
|  |  |  |  |  |  |  |  |  |  |  |  |  |  |  |  |  | High | FEEDLDAMLPK | 2 |  | 0,0000 | 3,06 | 2 | 1307,61943 | 0,44 | 49,04 |
|  |  |  |  |  |  |  |  |  |  |  |  |  |  |  |  |  | High | FEEDLDAmLPK | 1 | M8(Oxidation) | 0,0000 | 2,96 | 2 | 1323,61443 | 0,49 | 44,50 |
|  |  |  |  |  |  |  |  |  |  |  |  |  |  |  |  |  | High | GEDFPAQNYIVK | 3 |  | 0,0000 | 2,95 | 2 | 1380,68010 | 0,46 | 45,01 |
|  |  |  |  |  |  |  |  |  |  |  |  |  |  |  |  |  | High | GVLIVnNAR | 2 | N6(Deamidated) | 0,0000 | 2,81 | 2 | 956,55229 | -0,12 | 41,59 |
|  |  |  |  |  |  |  |  |  |  |  |  |  |  |  |  |  | High | KGVLIVNNAR | 3 |  | 0,0000 | 2,58 | 2 | 1083,66301 | -0,32 | 36,51 |
|  |  |  |  |  |  |  |  |  |  |  |  |  |  |  |  |  | High | cDVIVINTPLTEK | 2 | C1(Carbamidomethyl) | 0,0000 | 2,56 | 2 | 1501,79497 | 1,23 | 46,63 |
|  |  |  |  |  |  |  |  |  |  |  |  |  |  |  |  |  | High | GVLIVNNAR | 3 |  | 0,0000 | 2,49 | 2 | 955,56804 | -0,37 | 40,55 |
|  |  |  |  |  |  |  |  |  |  |  |  |  |  |  |  |  | Medium | YIVTDDKDGPDcELDK | 2 | C12(Carbamidomethyl) | 0,0000 | 2,26 | 3 | 1882,83872 | 0,60 | 39,66 |
|  |  |  |  |  |  |  |  |  |  |  |  |  |  |  |  |  | High | GVIASAVR | 3 |  | 0,0000 | 2,17 | 2 | 772,46764 | 0,03 | 38,30 |
|  |  |  |  |  |  |  |  |  |  |  |  |  |  |  |  |  | Medium | HIQDLHVLISTPFHPAYVTAER | 1 |  | 0,0000 | 2,11 | 4 | 2544,33315 | -1,06 | 45,33 |
|  |  |  |  |  |  |  |  |  |  |  |  |  |  |  |  |  | High | IVGVFYK | 2 |  | 0,0000 | 2,08 | 2 | 825,48705 | 0,14 | 43,62 |
|  |  |  |  |  |  |  |  |  |  |  |  |  |  |  |  |  | Medium | ILILVR | 3 |  | 0,0000 | 1,96 | 2 | 726,52367 | 0,01 | 45,57 |
|  |  |  |  |  |  |  |  |  |  |  |  |  |  |  |  |  | Medium | DGPDcELDK | 1 | C5(Carbamidomethyl) | 0,0000 | 1,84 | 2 | 1048,42534 | 0,11 | 37,22 |
|  |  |  |  |  |  |  |  |  |  |  |  |  |  |  |  |  | Medium | DWLESQGHK | 2 |  | 0,0000 | 1,84 | 2 | 1099,51677 | 0,03 | 39,89 |
| **8310** | B2BMP9 | Endo-1,4-beta-mannosidase | Endo-1,4-beta-mannosidase protein 1 OS=Prunus persica PE=2 SV=1 - [B2BMP9_PRUPE] |  | Prunus persica | Chloroplast | 06.07-Protein destination and storage/Modification | 15,56 | 7,89 | 2 | 4 | 4 | 9 | 431 | 48,7 | 8,60 | High | ILSAFQEATK | 2 |  | 0,0000 | 2,63 | 2 | 1107,60430 | -0,19 | 44,32 |
|  |  |  |  |  |  |  |  |  |  |  |  |  |  |  |  |  | High | TNSLTGVAYK | 2 |  | 0,0000 | 2,60 | 2 | 1053,55718 | -0,34 | 39,95 |
|  |  |  |  |  |  |  |  |  |  |  |  |  |  |  |  |  | High | HGLSIAR | 3 |  | 0,0000 | 2,08 | 2 | 753,43651 | -0,16 | 36,85 |
|  |  |  |  |  |  |  |  |  |  |  |  |  |  |  |  |  | Medium | QYVDWAR | 2 |  | 0,0000 | 1,49 | 2 | 937,45201 | -0,70 | 43,65 |
|  | M5VW13 | glycerate dehydrogenase | Uncharacterized protein OS=Prunus persica GN=PRUPE_ppa006993mg PE=3 SV=1 - [M5VW13_PRUPE] | 99% to GI:645260882 Prunus mume, E value 0 | Prunus mume | Chloroplast | 02.30-Energy/Photosynthesis | 13,34 | 23,06 | 2 | 9 | 9 | 14 | 386 | 42,2 | 7,80 | High | GQTVGVIGAGR | 2 |  | 0,0000 | 2,49 | 2 | 1014,56883 | -0,29 | 40,54 |
|  |  |  |  |  |  |  |  |  |  |  |  |  |  |  |  |  | High | IVEADEFMR | 1 |  | 0,0000 | 2,48 | 2 | 1109,52959 | -0,05 | 44,82 |
|  |  |  |  |  |  |  |  |  |  |  |  |  |  |  |  |  | High | ANGEQPVTWK | 1 |  | 0,0000 | 2,41 | 2 | 1129,56365 | -0,05 | 39,89 |
|  |  |  |  |  |  |  |  |  |  |  |  |  |  |  |  |  | Medium | AASMEEVLR | 1 |  | 0,0000 | 1,87 | 2 | 1005,50298 | -0,44 | 42,65 |
|  |  |  |  |  |  |  |  |  |  |  |  |  |  |  |  |  | Medium | ALGLPVSR | 2 |  | 0,0000 | 1,81 | 2 | 812,49901 | 0,12 | 44,06 |
|  |  |  |  |  |  |  |  |  |  |  |  |  |  |  |  |  | Medium | NAIVVPHIASASK | 1 |  | 0,0000 | 1,42 | 2 | 1306,74907 | 0,96 | 41,59 |
|  |  |  |  |  |  |  |  |  |  |  |  |  |  |  |  |  | Medium | TTYHLVNK | 2 |  | 0,0000 | 1,23 | 2 | 975,52483 | -1,02 | 37,22 |
|  |  |  |  |  |  |  |  |  |  |  |  |  |  |  |  |  | Medium | NAPPPAASPSIVNAK | 2 |  | 0,0000 | 1,09 | 2 | 1433,77532 | 0,37 | 39,78 |
|  |  |  |  |  |  |  |  |  |  |  |  |  |  |  |  |  | Medium | QNPMFR | 2 |  | 0,0000 | 1,09 | 2 | 792,38194 | -0,27 | 40,26 |
| **8311** | M5WG66 | src substrate protein | Uncharacterized protein OS=Prunus persica GN=PRUPE_ppa008017mg PE=4 SV=1 - [M5WG66_PRUPE] | 99% to GI:645237812 Prunus mume, E value 0 | Prunus persica | Chloroplast | 09.04-Cell structure/Cytoskeleton | 26,03 | 29,89 | 3 | 10 | 11 | 18 | 348 | 38,8 | 7,52 | High | MRQEAETQAVEVSR | 2 |  | 0,0000 | 3,25 | 3 | 1633,79569 | -0,39 | 37,04 |
|  |  |  |  |  |  |  |  |  |  |  |  |  |  |  |  |  | High | AMIAGAPLEDAR | 2 |  | 0,0000 | 2,82 | 2 | 1214,61968 | -0,16 | 43,53 |
|  |  |  |  |  |  |  |  |  |  |  |  |  |  |  |  |  | High | QEAETQAVEVSR | 2 |  | 0,0000 | 2,68 | 2 | 1346,65569 | 0,72 | 37,78 |
|  |  |  |  |  |  |  |  |  |  |  |  |  |  |  |  |  | High | AmIAGAPLEDAR | 1 | M2(Oxidation) | 0,0000 | 2,35 | 2 | 1230,61492 | 0,11 | 40,32 |
|  |  |  |  |  |  |  |  |  |  |  |  |  |  |  |  |  | Medium | KESAPPVIPQENSSEK | 1 |  | 0,0000 | 2,33 | 3 | 1739,88022 | -0,51 | 37,13 |
|  |  |  |  |  |  |  |  |  |  |  |  |  |  |  |  |  | High | AAEVFTAIGYK | 1 |  | 0,0000 | 2,33 | 2 | 1169,62065 | 0,43 | 45,11 |
|  |  |  |  |  |  |  |  |  |  |  |  |  |  |  |  |  | High | LVALVEGEK | 1 |  | 0,0000 | 2,05 | 2 | 957,56133 | -0,27 | 41,02 |
|  |  |  |  |  |  |  |  |  |  |  |  |  |  |  |  |  | Medium | mRQEAETQAVEVSR | 1 | M1(Oxidation) | 0,0000 | 1,73 | 3 | 1649,79251 | 0,77 | 35,77 |
|  |  |  |  |  |  |  |  |  |  |  |  |  |  |  |  |  | Medium | YGAENTNDKILAK | 1 |  | 0,0000 | 1,65 | 3 | 1436,73850 | 0,32 | 36,73 |
|  |  |  |  |  |  |  |  |  |  |  |  |  |  |  |  |  | Medium | ESAPPVIPQENSSEK | 2 |  | 0,0000 | 1,58 | 2 | 1611,78752 | 0,86 | 40,44 |
|  |  |  |  |  |  |  |  |  |  |  |  |  |  |  |  |  | Medium | mDALRK | 2 | N-Term(Acetyl) | 0,0000 | 1,57 | 2 | 775,41319 | 0,08 | 41,03 |
|  |  |  |  |  |  |  |  |  |  |  |  |  |  |  |  |  | Medium | VSPSGWSEGEcK | 1 | C11(Carbamidomethyl) | 0,0000 | 1,30 | 2 | 1322,56841 | 0,15 | 39,40 |
| **8401** | M5WZU8 | V-type proton ATPase subunit H | Uncharacterized protein OS=Prunus persica GN=PRUPE_ppa005592mg PE=4 SV=1 - [M5WZU8_PRUPE] | 96% to GI:658016951 Malus domestica, E value 0 | Malus domestica | Chloroplast | 07.22-Transporters/Transport ATPases | 14,20 | 19,69 | 4 | 10 | 10 | 14 | 452 | 51,3 | 7,42 | High | SSFVQLDGVK | 2 |  | 0,0000 | 2,32 | 2 | 1079,57268 | -0,48 | 43,54 |
|  |  |  |  |  |  |  |  |  |  |  |  |  |  |  |  |  | High | mDHAELTTEQVLK | 1 | N-Term(Acetyl) | 0,0000 | 2,22 | 2 | 1556,76470 | 1,37 | 45,82 |
|  |  |  |  |  |  |  |  |  |  |  |  |  |  |  |  |  | High | NALLcIQR | 2 | C5(Carbamidomethyl) | 0,0000 | 2,11 | 2 | 987,53942 | -1,05 | 42,21 |
|  |  |  |  |  |  |  |  |  |  |  |  |  |  |  |  |  | Medium | LFHDGTIVDK | 1 |  | 0,0000 | 2,11 | 3 | 1144,59903 | -0,63 | 39,07 |
|  |  |  |  |  |  |  |  |  |  |  |  |  |  |  |  |  | Medium | IIVTDLK | 2 |  | 0,0000 | 1,60 | 2 | 801,50792 | -0,19 | 42,25 |
|  |  |  |  |  |  |  |  |  |  |  |  |  |  |  |  |  | Medium | VVVLTLR | 2 |  | 0,0000 | 1,23 | 2 | 799,54009 | 0,06 | 44,54 |
|  |  |  |  |  |  |  |  |  |  |  |  |  |  |  |  |  | Medium | GNWFIQEK | 1 |  | 0,0167 | 1,18 | 2 | 1021,50994 | -0,26 | 44,48 |
|  |  |  |  |  |  |  |  |  |  |  |  |  |  |  |  |  | Medium | LMNHENAEVTK | 1 |  | 0,0000 | 1,02 | 2 | 1285,62212 | 1,20 | 34,29 |
|  |  |  |  |  |  |  |  |  |  |  |  |  |  |  |  |  | Medium | LFLGAK | 1 |  | 0,0211 | 0,93 | 2 | 648,40801 | 0,07 | 42,06 |
|  |  |  |  |  |  |  |  |  |  |  |  |  |  |  |  |  | Medium | ITTIDDVLK | 1 |  | 0,0000 | 0,91 | 2 | 1017,58275 | 0,05 | 45,46 |
| **8405** | M5X0L8 | 3-oxoacyl-[acyl-carrier-protein] synthase | Uncharacterized protein OS=Prunus persica GN=PRUPE_ppa005228mg PE=3 SV=1 - [M5X0L8_PRUPE] | 99% to GI:645237961 Prunus mume, E value 0 | Prunus mume | Chloroplast | 01.06-Metabolism/Lipid and sterol | 52,23 | 11,04 | 1 | 8 | 8 | 22 | 471 | 50,0 | 8,31 | High | ALEDADLGGDKR | 5 |  | 0,0000 | 3,81 | 2 | 1259,62322 | 0,41 | 37,47 |
|  |  |  |  |  |  |  |  |  |  |  |  |  |  |  |  |  | High | RLDDcLR | 3 | C5(Carbamidomethyl) | 0,0000 | 3,43 | 2 | 947,47228 | -0,53 | 36,63 |
|  |  |  |  |  |  |  |  |  |  |  |  |  |  |  |  |  | High | ADGLGVSTcIER | 3 | C9(Carbamidomethyl) | 0,0000 | 3,28 | 2 | 1277,61626 | 0,60 | 41,86 |
|  |  |  |  |  |  |  |  |  |  |  |  |  |  |  |  |  | High | ALEDADLGGDK | 2 |  | 0,0000 | 3,27 | 2 | 1103,52165 | 0,07 | 39,87 |
|  |  |  |  |  |  |  |  |  |  |  |  |  |  |  |  |  | High | LLAGESGVGPIDR | 4 |  | 0,0000 | 2,93 | 2 | 1283,69524 | -0,18 | 43,44 |
|  |  |  |  |  |  |  |  |  |  |  |  |  |  |  |  |  | Medium | KALEDADLGGDK | 1 |  | 0,0000 | 2,42 | 3 | 1231,61597 | -0,47 | 36,03 |
|  |  |  |  |  |  |  |  |  |  |  |  |  |  |  |  |  | High | LDDcLR | 2 | C4(Carbamidomethyl) | 0,0000 | 2,17 | 2 | 791,37139 | -0,34 | 38,08 |
|  |  |  |  |  |  |  |  |  |  |  |  |  |  |  |  |  | Medium | YSIVAGK | 2 |  | 0,0000 | 1,91 | 2 | 737,41936 | 0,16 | 38,68 |
| **8510** | M5VLY6 | Adenylyl cyclase-associated protein | Adenylyl cyclase-associated protein OS=Prunus persica GN=PRUPE_ppa005136mg PE=3 SV=1 - [M5VLY6_PRUPE] |  | Prunus persica | Chloroplast | 09.04-Cell structure/Cytoskeleton | 122,40 | 34,32 | 1 | 19 | 19 | 72 | 475 | 51,0 | 7,65 | High | VLQQAFAVQK | 7 |  | 0,0000 | 3,51 | 2 | 1131,65239 | 0,24 | 41,61 |
|  |  |  |  |  |  |  |  |  |  |  |  |  |  |  |  |  | High | VNNITIDK | 4 |  | 0,0000 | 3,30 | 2 | 916,50945 | -0,45 | 38,49 |
|  |  |  |  |  |  |  |  |  |  |  |  |  |  |  |  |  | High | DSVVQIQGK | 5 |  | 0,0000 | 3,04 | 2 | 973,53105 | -0,29 | 39,49 |
|  |  |  |  |  |  |  |  |  |  |  |  |  |  |  |  |  | High | DALGASITTAK | 4 |  | 0,0000 | 2,88 | 2 | 1047,56743 | -0,66 | 41,59 |
|  |  |  |  |  |  |  |  |  |  |  |  |  |  |  |  |  | High | FETTPVSHSAGG | 3 |  | 0,0000 | 2,68 | 2 | 1189,54802 | -0,35 | 38,06 |
|  |  |  |  |  |  |  |  |  |  |  |  |  |  |  |  |  | High | DLVISDcDSK | 5 | C7(Carbamidomethyl) | 0,0000 | 2,36 | 2 | 1151,52483 | -0,09 | 44,11 |
|  |  |  |  |  |  |  |  |  |  |  |  |  |  |  |  |  | High | IGGPVLDVTK | 10 |  | 0,0000 | 2,33 | 2 | 998,58812 | -0,01 | 46,17 |
|  |  |  |  |  |  |  |  |  |  |  |  |  |  |  |  |  | High | KDLVISDcDSK | 4 | C8(Carbamidomethyl) | 0,0000 | 2,31 | 2 | 1279,61907 | -0,66 | 36,56 |
|  |  |  |  |  |  |  |  |  |  |  |  |  |  |  |  |  | High | DLLIQVK | 2 |  | 0,0000 | 2,20 | 2 | 828,51903 | 0,07 | 46,41 |
|  |  |  |  |  |  |  |  |  |  |  |  |  |  |  |  |  | High | LELQMGR | 2 |  | 0,0000 | 2,14 | 2 | 846,45000 | -0,30 | 41,60 |
|  |  |  |  |  |  |  |  |  |  |  |  |  |  |  |  |  | High | KWAVENQIGK | 2 |  | 0,0000 | 2,05 | 2 | 1172,64238 | 0,08 | 38,76 |
|  |  |  |  |  |  |  |  |  |  |  |  |  |  |  |  |  | Medium | LELQmGR | 5 | M5(Oxidation) | 0,0000 | 1,78 | 2 | 862,44493 | -0,27 | 37,29 |
|  |  |  |  |  |  |  |  |  |  |  |  |  |  |  |  |  | Medium | SDFFNHLK | 2 |  | 0,0000 | 1,74 | 2 | 1007,49425 | -0,29 | 42,07 |
|  |  |  |  |  |  |  |  |  |  |  |  |  |  |  |  |  | Medium | QSVYIFGcK | 4 | C8(Carbamidomethyl) | 0,0000 | 1,69 | 2 | 1101,53996 | 0,17 | 45,85 |
|  |  |  |  |  |  |  |  |  |  |  |  |  |  |  |  |  | Medium | DPNHLEWAK | 1 |  | 0,0000 | 1,66 | 2 | 1109,53703 | -0,40 | 39,43 |
|  |  |  |  |  |  |  |  |  |  |  |  |  |  |  |  |  | Medium | SKDPNHLEWAK | 1 |  | 0,0000 | 1,56 | 3 | 1324,66394 | -0,41 | 36,34 |
|  |  |  |  |  |  |  |  |  |  |  |  |  |  |  |  |  | Medium | ADRTGIVGTQEK | 1 |  | 0,0000 | 1,47 | 3 | 1274,67185 | 1,47 | 34,70 |
|  |  |  |  |  |  |  |  |  |  |  |  |  |  |  |  |  | Medium | ELYLPGLR | 3 |  | 0,0000 | 1,44 | 2 | 960,55089 | -0,46 | 47,94 |
|  |  |  |  |  |  |  |  |  |  |  |  |  |  |  |  |  | Medium | ILVEYK | 6 |  | 0,0000 | 1,27 | 2 | 764,45531 | 0,02 | 43,94 |
|  |  |  |  |  |  |  |  |  |  |  |  |  |  |  |  |  | Medium | SFYPLGPVWSSTGK | 1 |  | 0,0000 | 1,21 | 2 | 1525,76970 | 0,71 | 49,11 |
| **8511** | M5W5K0 | Pyruvate kinase | Pyruvate kinase OS=Prunus persica GN=PRUPE_ppa004703mg PE=3 SV=1 - [M5W5K0_PRUPE] |  | Prunus mume | Chloroplast | 02.01-Energy/Glycolysis | 20,79 | 18,38 | 1 | 9 | 10 | 20 | 495 | 53,5 | 7,90 | High | IVcTLGPASR | 1 | C3(Carbamidomethyl) | 0,0000 | 2,63 | 2 | 1073,57659 | -0,61 |  |
|  |  |  |  |  |  |  |  |  |  |  |  |  |  |  |  |  | High | cENSAVLGER | 2 | C1(Carbamidomethyl) | 0,0000 | 2,52 | 2 | 1134,52031 | -0,48 |  |
|  |  |  |  |  |  |  |  |  |  |  |  |  |  |  |  |  | High | NGDAVVALHR | 3 |  | 0,0000 | 2,27 | 2 | 1051,56438 | 0,01 |  |
|  |  |  |  |  |  |  |  |  |  |  |  |  |  |  |  |  | High | GSDLVEVR | 2 |  | 0,0000 | 2,25 | 2 | 874,46263 | -0,33 |  |
|  |  |  |  |  |  |  |  |  |  |  |  |  |  |  |  |  | High | nGDAVVALHR | 3 | N1(Deamidated) | 0,0000 | 2,23 | 2 | 1052,54802 | -0,35 |  |
|  |  |  |  |  |  |  |  |  |  |  |  |  |  |  |  |  | Medium | GDETTIcMSYK | 1 | C7(Carbamidomethyl) | 0,0000 | 1,77 | 2 | 1304,54741 | -1,80 |  |
|  |  |  |  |  |  |  |  |  |  |  |  |  |  |  |  |  | Medium | GLVPVLSAGSSR | 2 |  | 0,0000 | 1,62 | 2 | 1142,65288 | 0,02 |  |
|  |  |  |  |  |  |  |  |  |  |  |  |  |  |  |  |  | Medium | GDLGMEIPIEK | 1 |  | 0,0000 | 1,58 | 2 | 1201,61272 | -0,55 |  |
|  |  |  |  |  |  |  |  |  |  |  |  |  |  |  |  |  | Medium | IFLAQK | 2 |  | 0,0000 | 1,55 | 2 | 719,44518 | 0,15 |  |
|  |  |  |  |  |  |  |  |  |  |  |  |  |  |  |  |  | Medium | GDETTIcmSYK | 1 | C7(Carbamidomethyl); M8(Oxidation) | 0,0000 | 1,44 | 2 | 1320,54521 | 0,40 |  |
|  |  |  |  |  |  |  |  |  |  |  |  |  |  |  |  |  | Medium | WGVPNK | 1 |  | 0,0274 | 1,42 | 2 | 700,37779 | 0,10 |  |
|  |  |  |  |  |  |  |  |  |  |  |  |  |  |  |  |  | Medium | NILLMSK | 1 |  | 0,0303 | 1,28 | 2 | 818,48046 | -0,03 |  |
| **8520** | M5VLY6 | Adenylyl cyclase-associated protein | Adenylyl cyclase-associated protein OS=Prunus persica GN=PRUPE_ppa005136mg PE=3 SV=1 - [M5VLY6_PRUPE] |  | Prunus persica | Chloroplast | 09.04-Cell structure/Cytoskeleton | 54,58 | 19,58 | 1 | 11 | 11 | 27 | 475 | 51,0 | 7,65 | High | DSVVQIQGK | 3 |  | 0,0000 | 3,14 | 2 | 973,53105 | -0,29 | 39,46 |
|  |  |  |  |  |  |  |  |  |  |  |  |  |  |  |  |  | High | VLQQAFAVQK | 3 |  | 0,0000 | 3,01 | 2 | 1131,65227 | 0,13 | 41,50 |
|  |  |  |  |  |  |  |  |  |  |  |  |  |  |  |  |  | High | IGGPVLDVTK | 3 |  | 0,0000 | 3,00 | 2 | 998,58794 | -0,19 | 44,06 |
|  |  |  |  |  |  |  |  |  |  |  |  |  |  |  |  |  | High | VNNITIDK | 2 |  | 0,0000 | 2,91 | 2 | 916,50932 | -0,59 | 37,63 |
|  |  |  |  |  |  |  |  |  |  |  |  |  |  |  |  |  | High | VNnITIDK | 2 | N3(Deamidated) | 0,0000 | 2,87 | 2 | 917,49382 | -0,06 | 39,37 |
|  |  |  |  |  |  |  |  |  |  |  |  |  |  |  |  |  | High | DALGASITTAK | 3 |  | 0,0000 | 2,54 | 2 | 1047,56780 | -0,31 | 42,66 |
|  |  |  |  |  |  |  |  |  |  |  |  |  |  |  |  |  | High | DLVISDcDSK | 3 | C7(Carbamidomethyl) | 0,0000 | 2,45 | 2 | 1151,52507 | 0,12 | 42,07 |
|  |  |  |  |  |  |  |  |  |  |  |  |  |  |  |  |  | High | FETTPVSHSAGG | 2 |  | 0,0000 | 2,39 | 2 | 1189,54827 | -0,15 | 37,96 |
|  |  |  |  |  |  |  |  |  |  |  |  |  |  |  |  |  | Medium | ILVEYK | 2 |  | 0,0000 | 1,68 | 2 | 764,45537 | 0,10 | 40,68 |
|  |  |  |  |  |  |  |  |  |  |  |  |  |  |  |  |  | Medium | LELQmGR | 1 | M5(Oxidation) | 0,0000 | 1,43 | 2 | 862,44481 | -0,41 | 37,24 |
|  |  |  |  |  |  |  |  |  |  |  |  |  |  |  |  |  | Medium | QSVYIFGcK | 2 | C8(Carbamidomethyl) | 0,0000 | 1,42 | 2 | 1101,53935 | -0,38 | 44,04 |
|  |  |  |  |  |  |  |  |  |  |  |  |  |  |  |  |  | Medium | KDLVISDcDSK | 1 | C8(Carbamidomethyl) | 0,0000 | 1,40 | 2 | 1279,62114 | 0,97 | 36,98 |
| **8521** | M5WPC0 | Cannabidiolic acid synthase | Uncharacterized protein OS=Prunus persica GN=PRUPE_ppa003798mg PE=4 SV=1 - [M5WPC0_PRUPE] | 97% to GI:645238389 Prunus mume, E value 0 | Prunus persica | Plasma membrane | 20.99-Secondary metabolism/Others | 22,01 | 13,14 | 3 | 7 | 9 | 27 | 548 | 61,4 | 8,60 | High | YGLGADNVIDAR | 3 |  | 0,0000 | 3,92 | 2 | 1263,63347 | 0,49 | 44,49 |
|  |  |  |  |  |  |  |  |  |  |  |  |  |  |  |  |  | Medium | NGILFK | 3 |  | 0,0000 | 1,98 | 2 | 691,41393 | 0,23 | 42,92 |
|  |  |  |  |  |  |  |  |  |  |  |  |  |  |  |  |  | Medium | nGILFK | 5 | N1(Deamidated) | 0,0000 | 1,93 | 2 | 692,39787 | 0,13 | 47,00 |
|  |  |  |  |  |  |  |  |  |  |  |  |  |  |  |  |  | Medium | DcTETSWLK | 3 | C2(Carbamidomethyl) | 0,0000 | 1,90 | 2 | 1139,50408 | 0,25 | 45,15 |
|  |  |  |  |  |  |  |  |  |  |  |  |  |  |  |  |  | Medium | IIDVSGR | 3 |  | 0,0000 | 1,83 | 2 | 759,43590 | -0,09 | 39,03 |
|  |  |  |  |  |  |  |  |  |  |  |  |  |  |  |  |  | Medium | DLDLGINKK | 3 |  | 0,0000 | 1,60 | 2 | 1015,57817 | -0,12 | 40,49 |
|  |  |  |  |  |  |  |  |  |  |  |  |  |  |  |  |  | Medium | FPELGLTK | 2 |  | 0,0000 | 1,58 | 2 | 904,51354 | -0,39 | 45,83 |
|  |  |  |  |  |  |  |  |  |  |  |  |  |  |  |  |  | Medium | LLDVVK | 1 |  | 0,0000 | 1,36 | 2 | 686,44481 | 0,10 | 42,64 |
|  |  |  |  |  |  |  |  |  |  |  |  |  |  |  |  |  | Medium | QAYVNYR | 2 |  | 0,0000 | 1,30 | 2 | 913,45232 | -0,36 | 38,31 |
|  |  |  |  |  |  |  |  |  |  |  |  |  |  |  |  |  | Medium | VDPDNFFR | 2 |  | 0,0000 | 0,94 | 2 | 1009,47386 | 0,04 | 46,02 |
| **8524** | M5VKY8 | Serine hydroxymethyltransferase | Serine hydroxymethyltransferase OS=Prunus persica GN=PRUPE_ppa004090mg PE=3 SV=1 - [M5VKY8_PRUPE] |  | Prunus persica | Chloroplast | 01.01-Metabolism/Amino acid | 16,41 | 13,58 | 2 | 9 | 10 | 16 | 530 | 57,7 | 8,34 | High | LIIAGASAYPR | 1 |  | 0,0000 | 2,40 | 2 | 1131,65288 | 0,67 | 41,99 |
|  |  |  |  |  |  |  |  |  |  |  |  |  |  |  |  |  | High | GGMIFFK | 2 |  | 0,0000 | 2,19 | 2 | 799,41734 | 0,23 | 46,76 |
|  |  |  |  |  |  |  |  |  |  |  |  |  |  |  |  |  | High | YAQSPEFK | 2 |  | 0,0000 | 2,03 | 2 | 969,46794 | 0,30 | 36,83 |
|  |  |  |  |  |  |  |  |  |  |  |  |  |  |  |  |  | Medium | LTELGYK | 2 |  | 0,0000 | 1,99 | 2 | 823,45574 | -0,35 | 38,43 |
|  |  |  |  |  |  |  |  |  |  |  |  |  |  |  |  |  | Medium | SAVVPGGIR | 2 |  | 0,0000 | 1,68 | 2 | 855,50450 | -0,27 | 38,11 |
|  |  |  |  |  |  |  |  |  |  |  |  |  |  |  |  |  | Medium | YSEGLPGK | 1 |  | 0,0000 | 1,55 | 2 | 850,43053 | -0,01 | 36,65 |
|  |  |  |  |  |  |  |  |  |  |  |  |  |  |  |  |  | Medium | IGSPAMTTR | 2 |  | 0,0000 | 1,53 | 2 | 933,48192 | -0,39 | 36,18 |
|  |  |  |  |  |  |  |  |  |  |  |  |  |  |  |  |  | Medium | LQDFMR | 1 |  | 0,0779 | 1,42 | 2 | 809,39745 | -0,04 | 40,55 |
|  |  |  |  |  |  |  |  |  |  |  |  |  |  |  |  |  | Medium | DFDYPR | 2 |  | 0,0000 | 1,28 | 2 | 812,35753 | 0,20 | 40,99 |
| **8528** | M5WY75 | Catalase | Catalase OS=Prunus persica GN=PRUPE_ppa004763mg PE=3 SV=1 - [M5WY75_PRUPE] |  | Prunus persica | Peroxisome | 01.01-Metabolism/Amino acid | 88,18 | 29,47 | 3 | 8 | 17 | 57 | 492 | 57,0 | 7,43 | High | WVDALSDPR | 4 |  | 0,0000 | 3,28 | 2 | 1058,52641 | -0,17 | 42,37 |
|  |  |  |  |  |  |  |  |  |  |  |  |  |  |  |  |  | High | DEEVNYFPSR | 3 |  | 0,0000 | 2,93 | 2 | 1255,55950 | 0,39 | 43,46 |
|  |  |  |  |  |  |  |  |  |  |  |  |  |  |  |  |  | High | cVIEKENNFK | 3 | C1(Carbamidomethyl) | 0,0000 | 2,80 | 2 | 1280,63066 | 0,20 | 35,42 |
|  |  |  |  |  |  |  |  |  |  |  |  |  |  |  |  |  | High | APGVQTPVIVR | 6 |  | 0,0000 | 2,77 | 2 | 1136,67888 | 0,18 | 42,56 |
|  |  |  |  |  |  |  |  |  |  |  |  |  |  |  |  |  | High | IFAYSDTQR | 4 |  | 0,0000 | 2,73 | 2 | 1100,53728 | 0,13 | 39,26 |
|  |  |  |  |  |  |  |  |  |  |  |  |  |  |  |  |  | High | DAMKFPDAIR | 5 |  | 0,0000 | 2,67 | 2 | 1163,58769 | -0,12 | 42,88 |
|  |  |  |  |  |  |  |  |  |  |  |  |  |  |  |  |  | High | SHIQETWR | 4 |  | 0,0000 | 2,60 | 2 | 1056,52190 | -0,24 | 36,33 |
|  |  |  |  |  |  |  |  |  |  |  |  |  |  |  |  |  | High | cLLEDEAIK | 3 | C1(Carbamidomethyl) | 0,0000 | 2,56 | 2 | 1090,54448 | -0,43 | 42,66 |
|  |  |  |  |  |  |  |  |  |  |  |  |  |  |  |  |  | High | FSTVIHER | 5 |  | 0,0000 | 2,55 | 2 | 988,52086 | -0,23 | 36,97 |
|  |  |  |  |  |  |  |  |  |  |  |  |  |  |  |  |  | High | LGPNYLQLPVNAPK | 2 |  | 0,0000 | 2,45 | 2 | 1523,85832 | 0,15 | 47,00 |
|  |  |  |  |  |  |  |  |  |  |  |  |  |  |  |  |  | High | GPVLLEDYHLVEK | 1 |  | 0,0000 | 2,23 | 2 | 1511,81279 | 1,53 | 44,16 |
|  |  |  |  |  |  |  |  |  |  |  |  |  |  |  |  |  | High | LNVRPSI | 4 |  | 0,0000 | 2,21 | 2 | 798,48320 | -0,06 | 40,76 |
|  |  |  |  |  |  |  |  |  |  |  |  |  |  |  |  |  | High | DAmKFPDAIR | 4 | M3(Oxidation) | 0,0000 | 2,13 | 2 | 1179,58293 | 0,16 | 40,07 |
|  |  |  |  |  |  |  |  |  |  |  |  |  |  |  |  |  | Medium | LATFDRER | 3 |  | 0,0000 | 1,84 | 2 | 1007,52696 | 0,04 | 35,21 |
|  |  |  |  |  |  |  |  |  |  |  |  |  |  |  |  |  | Medium | SWAPDRQER | 1 |  | 0,1844 | 1,46 | 3 | 1144,54950 | 0,05 | 34,56 |
|  |  |  |  |  |  |  |  |  |  |  |  |  |  |  |  |  | Medium | FPDAIR | 1 |  | 0,0809 | 1,25 | 2 | 718,38841 | 0,17 | 40,79 |
|  |  |  |  |  |  |  |  |  |  |  |  |  |  |  |  |  | Medium | GSPETLRDPR | 2 |  | 0,0000 | 1,23 | 3 | 1127,58030 | -0,11 | 35,35 |
|  |  |  |  |  |  |  |  |  |  |  |  |  |  |  |  |  | Medium | LATFDR | 2 |  | 0,0000 | 1,08 | 2 | 722,38317 | -0,05 | 37,32 |
| **8529** | M5XWQ3 | NADH dehydrogenase [ubiquinone] flavoprotein | Uncharacterized protein OS=Prunus persica GN=PRUPE_ppa004904mg PE=4 SV=1 - [M5XWQ3_PRUPE] | 99% to GI:645230713 Prunus mume, E value 0 | Prunus persica | Mitochondrion | 02.20-Energy/Electron-transport | 17,94 | 8,64 | 1 | 6 | 6 | 14 | 486 | 53,2 | 7,93 | High | ASAAYIYIR | 2 |  | 0,0000 | 2,71 | 2 | 1027,55718 | 0,04 | 41,42 |
|  |  |  |  |  |  |  |  |  |  |  |  |  |  |  |  |  | High | STDIVDAIAR | 2 |  | 0,0000 | 2,53 | 2 | 1060,56304 | -0,32 | 43,16 |
|  |  |  |  |  |  |  |  |  |  |  |  |  |  |  |  |  | Medium | KEAYEAGLLGK | 1 |  | 0,0000 | 2,00 | 2 | 1178,64177 | 0,13 | 38,38 |
|  |  |  |  |  |  |  |  |  |  |  |  |  |  |  |  |  | Medium | EAYEAGLLGK | 3 |  | 0,0000 | 1,82 | 2 | 1050,54643 | -0,21 | 40,97 |
|  |  |  |  |  |  |  |  |  |  |  |  |  |  |  |  |  | Medium | LSYFYK | 2 |  | 0,0000 | 1,38 | 2 | 820,42406 | 0,11 | 40,92 |
|  |  |  |  |  |  |  |  |  |  |  |  |  |  |  |  |  | Medium | WSFmPK | 2 | M4(Oxidation) | 0,0000 | 1,12 | 2 | 811,38066 | -0,13 | 40,87 |
|  |  |  |  |  |  |  |  |  |  |  |  |  |  |  |  |  | Medium | WSFMPK | 2 |  | 0,0000 | 1,11 | 2 | 795,38561 | -0,31 | 44,01 |
| **8604** | M5X2C2 | Malic enzyme | Malic enzyme OS=Prunus persica GN=PRUPE_ppa003102mg PE=3 SV=1 - [M5X2C2_PRUPE] |  | Prunus persica | Mitochondrion | 02.10-Energy/TCA pathway | 21,58 | 16,72 | 1 | 12 | 12 | 21 | 604 | 66,6 | 7,80 | High | VISFEQQYAR | 2 |  | 0,0000 | 3,12 | 2 | 1240,63237 | 0,22 | 40,12 |
|  |  |  |  |  |  |  |  |  |  |  |  |  |  |  |  |  | High | GQPEGVVALAK | 1 |  | 0,0000 | 2,59 | 2 | 1068,60491 | 0,05 | 38,45 |
|  |  |  |  |  |  |  |  |  |  |  |  |  |  |  |  |  | High | DTGFPLTER | 1 |  | 0,0000 | 2,38 | 2 | 1035,51055 | -0,05 | 41,97 |
|  |  |  |  |  |  |  |  |  |  |  |  |  |  |  |  |  | High | WAFETLQR | 2 |  | 0,0000 | 2,25 | 2 | 1050,53655 | -0,19 | 43,37 |
|  |  |  |  |  |  |  |  |  |  |  |  |  |  |  |  |  | High | EETLEYVTR | 1 |  | 0,0000 | 2,14 | 2 | 1139,55803 | 0,09 | 39,22 |
|  |  |  |  |  |  |  |  |  |  |  |  |  |  |  |  |  | High | NETLYYR | 1 |  | 0,0000 | 2,09 | 2 | 958,46263 | -0,26 | 37,01 |
|  |  |  |  |  |  |  |  |  |  |  |  |  |  |  |  |  | Medium | AIVQFEDFQMK | 1 |  | 0,0000 | 1,93 | 2 | 1355,66814 | 1,23 | 45,35 |
|  |  |  |  |  |  |  |  |  |  |  |  |  |  |  |  |  | Medium | NLDPMAAPFAK | 1 |  | 0,0825 | 1,78 | 2 | 1174,59221 | -0,32 | 43,30 |
|  |  |  |  |  |  |  |  |  |  |  |  |  |  |  |  |  | Medium | LYLGLR | 5 |  | 0,0000 | 1,33 | 2 | 734,45561 | -0,47 | 44,17 |
|  |  |  |  |  |  |  |  |  |  |  |  |  |  |  |  |  | Medium | GLLPPR | 2 |  | 0,0000 | 1,03 | 2 | 652,41442 | 0,47 | 38,38 |
|  |  |  |  |  |  |  |  |  |  |  |  |  |  |  |  |  | Medium | FMESYR | 2 |  | 0,0000 | 1,03 | 2 | 832,36577 | -0,07 | 36,82 |
|  |  |  |  |  |  |  |  |  |  |  |  |  |  |  |  |  | Medium | ELMNMSK | 1 |  | 0,0098 | 1,01 | 2 | 852,39537 | -0,08 | 36,83 |
|  |  |  |  |  |  |  |  |  |  |  |  |  |  |  |  |  | Medium | FmESYR | 1 | M2(Oxidation) | 0,1415 | 0,91 | 2 | 848,36052 | -0,27 | 34,85 |
| **8611** | B6ZL96 | L-galactono-1,4-lactone dehydrogenase | L-galactono-1,4-lactone dehydrogenase (Fragment) OS=Prunus persica GN=GLDH PE=2 SV=1 - [B6ZL96_PRUPE] |  | Prunus persica | Mitochondrion | 20.99-Secondary metabolism/Others | 7,46 | 11,71 | 4 | 6 | 7 | 10 | 589 | 66,6 | 8,38 | High | DLEYIEDVK | 1 |  | 0,0000 | 2,31 | 2 | 1123,55181 | 0,01 | 45,87 |
|  |  |  |  |  |  |  |  |  |  |  |  |  |  |  |  |  | Medium | cSVVPVK | 1 | C1(Carbamidomethyl) | 0,0000 | 1,79 | 2 | 788,43370 | 0,22 | 36,24 |
|  |  |  |  |  |  |  |  |  |  |  |  |  |  |  |  |  | Medium | LPPMDEQVISMK | 1 |  | 0,0000 | 1,69 | 2 | 1387,69780 | 1,25 | 45,92 |
|  |  |  |  |  |  |  |  |  |  |  |  |  |  |  |  |  | Medium | NEVPAPAPIEQR | 2 |  | 0,0000 | 1,67 | 2 | 1320,69084 | 0,10 | 38,86 |
|  |  |  |  |  |  |  |  |  |  |  |  |  |  |  |  |  | Medium | YSSDEAIQHVR | 3 |  | 0,0000 | 1,46 | 3 | 1304,62278 | -0,17 | 37,09 |
|  |  |  |  |  |  |  |  |  |  |  |  |  |  |  |  |  | Medium | EELASLQAR | 1 |  | 0,0000 | 1,00 | 2 | 1016,53777 | 0,61 | 39,25 |
| **8615** | M5VMH1 | Histidine-tRNA ligase | Uncharacterized protein OS=Prunus persica GN=PRUPE_ppa003021mg PE=3 SV=1 - [M5VMH1_PRUPE] | 98% to GI:645264598 Prunus mume, E value 0 | Prunus mume | Chloroplast | 05.07-Protein synthesis/Translation control | 5,29 | 3,60 | 1 | 2 | 2 | 4 | 611 | 68,5 | 5,69 | Medium | GqNQTTRATK | 2 | Q2(Deamidated) | 0,0000 | 1,79 | 2 | 1105,56316 | 3,16 | 38,35 |
|  |  |  |  |  |  |  |  |  |  |  |  |  |  |  |  |  | Medium | DPEFDSLLNRVK | 2 |  | 0,0000 | 1,72 | 2 | 1432,74492 | 1,24 | 41,86 |
| **8702** | M5VWS4 | Small nuclear ribonucleoprotein 70 kDa | Uncharacterized protein OS=Prunus persica GN=PRUPE_ppa004309mg PE=4 SV=1 - [M5VWS4_PRUPE] |  | Prunus mume | Mitochondrion | 04.22-Transcription/mRNA processing | 5,89 | 6,38 | 1 | 3 | 3 | 3 | 517 | 62,0 | 8,73 | High | LGGGLGTTR | 1 |  | 0,0000 | 2,07 | 2 | 831,46727 | -1,29 | 36,30 |
|  |  |  |  |  |  |  |  |  |  |  |  |  |  |  |  |  | High | LNYESTESR | 1 |  | 0,0000 | 2,01 | 2 | 1098,50591 | -0,29 | 36,02 |
|  |  |  |  |  |  |  |  |  |  |  |  |  |  |  |  |  | Medium | FAEPGDPEYTAPVQK | 1 |  | 0,0000 | 1,81 | 2 | 1648,78642 | 0,62 | 41,01 |
| **8707** | M5W944 | Ras-group-related LRR protein | Uncharacterized protein OS=Prunus persica GN=PRUPE_ppa003554mg PE=4 SV=1 - [M5W944_PRUPE] |  | Prunus persica | Chloroplast | 08.99-Intracellular traffic/Others | 5,80 | 5,12 | 1 | 3 | 3 | 5 | 566 | 62,5 | 7,06 | High | VEENPLEVPPR | 3 |  | 0,0000 | 2,03 | 2 | 1278,66899 | 0,06 | 40,97 |
|  |  |  |  |  |  |  |  |  |  |  |  |  |  |  |  |  | Medium | EVKAQPAKQK | 1 |  | 0,0000 | 1,22 | 2 | 1126,64678 | -9,91 | 49,56 |
|  |  |  |  |  |  |  |  |  |  |  |  |  |  |  |  |  | Medium | ALPEAVGK | 1 |  | 0,0000 | 1,12 | 2 | 784,45653 | 0,19 | 36,88 |
| **8711** | M5XQJ0 | Phosphoenolpyruvate carboxykinase [ATP] | Uncharacterized protein OS=Prunus persica GN=PRUPE_ppa002490mg PE=3 SV=1 - [M5XQJ0_PRUPE] | 99% to GI:645224569 Prunus mume, E value 0 | Prunus mume | Cytoplasm | 02.02-Energy/Gluconeogenesis | 15,58 | 14,84 | 1 | 7 | 9 | 11 | 667 | 74,0 | 7,23 | High | AAEnGGFSFTR | 1 | N4(Deamidated) | 0,0000 | 3,09 | 2 | 1157,52251 | 0,23 | 40,90 |
|  |  |  |  |  |  |  |  |  |  |  |  |  |  |  |  |  | High | IIDAIHSGR | 2 |  | 0,0000 | 2,29 | 2 | 981,54729 | -0,37 | 34,33 |
|  |  |  |  |  |  |  |  |  |  |  |  |  |  |  |  |  | High | YAAMLSEK | 1 |  | 0,0000 | 2,27 | 2 | 912,44969 | 0,13 | 37,10 |
|  |  |  |  |  |  |  |  |  |  |  |  |  |  |  |  |  | High | DEETENELWWGK | 1 |  | 0,0000 | 2,16 | 2 | 1535,66594 | 0,64 | 46,68 |
|  |  |  |  |  |  |  |  |  |  |  |  |  |  |  |  |  | Medium | LLNASYTK | 1 |  | 0,0000 | 2,00 | 2 | 909,50377 | -0,29 | 36,01 |
|  |  |  |  |  |  |  |  |  |  |  |  |  |  |  |  |  | Medium | SSQPTTPInGK | 2 | N9(Deamidated) | 0,0000 | 1,89 | 2 | 1130,56902 | 0,16 | 33,99 |
|  |  |  |  |  |  |  |  |  |  |  |  |  |  |  |  |  | Medium | IQTHPEATSEVcHDDSGTPVK | 1 | C12(Carbamidomethyl) | 0,0000 | 1,84 | 4 | 2308,04873 | -1,03 | 34,04 |
| **9012** | M5W0H8 | CBS domain-containing protein | Uncharacterized protein OS=Prunus persica GN=PRUPE_ppa012574mg PE=4 SV=1 - [M5W0H8_PRUPE] | 97% to GI:645221749 Prunus mume, E value 1e-107 | Prunus mume | Chloroplast | 11.05-Disease/Defense/Stress responses | 102,93 | 66,87 | 1 | 10 | 10 | 43 | 163 | 18,1 | 8,29 | High | LVSYAPEVTAYVENGK | 7 |  | 0,0000 | 4,67 | 2 | 1739,88652 | 0,82 | 46,83 |
|  |  |  |  |  |  |  |  |  |  |  |  |  |  |  |  |  | High | ADELYHGDGIcQEK | 2 | C11(Carbamidomethyl) | 0,0000 | 4,13 | 2 | 1634,71343 | 1,13 | 38,28 |
|  |  |  |  |  |  |  |  |  |  |  |  |  |  |  |  |  | High | TFPVSAFENEEAANK | 11 |  | 0,0000 | 4,08 | 2 | 1653,77739 | 1,10 | 44,98 |
|  |  |  |  |  |  |  |  |  |  |  |  |  |  |  |  |  | High | TFPVSAFENEEAANKDVK | 3 |  | 0,0000 | 3,96 | 2 | 1995,96965 | 1,87 | 41,93 |
|  |  |  |  |  |  |  |  |  |  |  |  |  |  |  |  |  | High | SKADELYHGDGIcQEK | 3 | C13(Carbamidomethyl) | 0,0000 | 3,14 | 3 | 1849,83945 | 0,47 | 37,01 |
|  |  |  |  |  |  |  |  |  |  |  |  |  |  |  |  |  | High | LLTEISLPNGLLPLK | 2 |  | 0,0000 | 3,10 | 2 | 1620,99553 | 1,22 | 50,88 |
|  |  |  |  |  |  |  |  |  |  |  |  |  |  |  |  |  | High | DmEEcGYVK | 2 | M2(Oxidation); C5(Carbamidomethyl) | 0,0000 | 2,43 | 2 | 1146,44426 | 0,02 | 35,97 |
|  |  |  |  |  |  |  |  |  |  |  |  |  |  |  |  |  | High | DMEEcGYVK | 3 | C5(Carbamidomethyl) | 0,0000 | 2,39 | 2 | 1130,44988 | 0,49 | 40,00 |
|  |  |  |  |  |  |  |  |  |  |  |  |  |  |  |  |  | High | ELLVWVSLSDIYVDDPPTGK | 1 |  | 0,0000 | 2,16 | 2 | 2246,16411 | 2,19 | 53,53 |
|  |  |  |  |  |  |  |  |  |  |  |  |  |  |  |  |  | Medium | TPSGLFR | 4 |  | 0,0000 | 1,58 | 2 | 777,42571 | 0,39 | 40,56 |
|  |  |  |  |  |  |  |  |  |  |  |  |  |  |  |  |  | Medium | ETGFVWLK | 5 |  | 0,0000 | 1,31 | 2 | 979,52446 | -0,34 | 46,44 |
| **9013** | M5W6L5 | Peptidyl-prolyl cis-trans isomerase | Peptidyl-prolyl cis-trans isomerase OS=Prunus persica GN=PRUPE_ppa012332mg PE=3 SV=1 - [M5W6L5_PRUPE] |  | Prunus persica | Cytoplasm | 06.01-Protein destination and storage/Folding and stability | 22,53 | 39,88 | 1 | 6 | 7 | 13 | 173 | 18,2 | 8,47 | High | IVMELFADTTPR | 2 |  | 0,0000 | 2,95 | 2 | 1392,71965 | 0,29 | 47,97 |
|  |  |  |  |  |  |  |  |  |  |  |  |  |  |  |  |  | High | VFFDMTIGGQPAGR | 2 |  | 0,0000 | 2,47 | 2 | 1495,73760 | 0,87 | 48,53 |
|  |  |  |  |  |  |  |  |  |  |  |  |  |  |  |  |  | High | IVmELFADTTPR | 1 | M3(Oxidation) | 0,0000 | 2,37 | 2 | 1408,71623 | 1,47 | 44,70 |
|  |  |  |  |  |  |  |  |  |  |  |  |  |  |  |  |  | High | TSRPVVVADcGQLS | 3 | C10(Carbamidomethyl) | 0,0000 | 2,19 | 2 | 1488,74870 | 0,76 | 41,60 |
|  |  |  |  |  |  |  |  |  |  |  |  |  |  |  |  |  | Medium | FADENFIKK | 1 |  | 0,0000 | 1,98 | 2 | 1111,57781 | -0,44 | 38,46 |
|  |  |  |  |  |  |  |  |  |  |  |  |  |  |  |  |  | Medium | VFFDmTIGGQPAGR | 1 | M5(Oxidation) | 0,0000 | 1,97 | 2 | 1511,73003 | -0,79 | 44,92 |
|  |  |  |  |  |  |  |  |  |  |  |  |  |  |  |  |  | Medium | FADENFIK | 1 |  | 0,0000 | 1,37 | 2 | 983,48296 | -0,37 | 42,75 |
|  |  |  |  |  |  |  |  |  |  |  |  |  |  |  |  |  | Medium | TEWLDGK | 1 |  | 0,0000 | 1,37 | 2 | 848,41448 | -0,50 | 40,72 |
| **9103** | M5X697 | thaumatin | Uncharacterized protein OS=Prunus persica GN=PRUPE_ppa010473mg PE=4 SV=1 - [M5X697_PRUPE] | 100% to GI:595941380 Prunus persica, E value 6e-179 | Prunus persica | Extracellular | 11.02-Disease/defense/Defense-related | 51,23 | 31,05 | 6 | 6 | 7 | 31 | 248 | 26,0 | 8,06 | High | YccTPPNDKPETcPPTDYSK | 2 | C2(Carbamidomethyl); C3(Carbamidomethyl); C13(Carbamidomethyl) | 0,0000 | 3,90 | 3 | 2430,00803 | 1,36 | 37,66 |
|  |  |  |  |  |  |  |  |  |  |  |  |  |  |  |  |  | High | SAcLAFNQPK | 8 | C3(Carbamidomethyl) | 0,0000 | 2,97 | 2 | 1135,55705 | 0,48 | 42,02 |
|  |  |  |  |  |  |  |  |  |  |  |  |  |  |  |  |  | High | SVDAPSPWSGR | 12 |  | 0,0000 | 2,69 | 2 | 1158,55425 | 0,32 | 41,35 |
|  |  |  |  |  |  |  |  |  |  |  |  |  |  |  |  |  | High | SACLAFNQPK | 1 |  | 0,0000 | 2,14 | 2 | 1078,53325 | -1,66 | 44,07 |
|  |  |  |  |  |  |  |  |  |  |  |  |  |  |  |  |  | High | GSDGSVIAcK | 1 | C9(Carbamidomethyl) | 0,0000 | 2,03 | 2 | 993,46691 | -0,12 | 35,36 |
|  |  |  |  |  |  |  |  |  |  |  |  |  |  |  |  |  | Medium | ASTcPADINK | 1 | C4(Carbamidomethyl) | 0,0000 | 1,23 | 2 | 1076,50408 | -0,06 | 34,57 |
|  |  |  |  |  |  |  |  |  |  |  |  |  |  |  |  |  | Medium | ASTcPADInK | 1 | C4(Carbamidomethyl); N9(Deamidated) | 0,0000 | 1,23 | 2 | 1077,48784 | -0,29 | 35,70 |
|  |  |  |  |  |  |  |  |  |  |  |  |  |  |  |  |  | Medium | VcPAPLQVK | 4 | C2(Carbamidomethyl) | 0,0000 | 1,03 | 2 | 1011,56548 | -0,14 | 40,50 |
| **9108** | M5W030 | Peroxidase | Uncharacterized protein OS=Prunus persica GN=PRUPE_ppa008489mg PE=3 SV=1 - [M5W030_PRUPE] | 97% to GI:645219989 Prunus mume, E value 0 | Prunus mume | Cell wall | 11.06-Disease/Defense/Detoxification | 14,46 | 17,93 | 3 | 5 | 5 | 5 | 329 | 35,6 | 7,96 | High | NVDPDIAINmDPNTPR | 1 | M10(Oxidation) | 0,0000 | 3,87 | 2 | 1797,84599 | 1,28 | 40,08 |
|  |  |  |  |  |  |  |  |  |  |  |  |  |  |  |  |  | High | VScADILALATR | 1 | C3(Carbamidomethyl) | 0,0000 | 3,18 | 2 | 1289,68901 | 0,58 | 46,10 |
|  |  |  |  |  |  |  |  |  |  |  |  |  |  |  |  |  | High | FQQTFVTVPATIR | 1 |  | 0,0000 | 2,72 | 2 | 1507,82695 | 0,10 | 43,73 |
|  |  |  |  |  |  |  |  |  |  |  |  |  |  |  |  |  | High | AAVDAVPQcK | 1 | C9(Carbamidomethyl) | 0,0000 | 2,51 | 2 | 1058,52983 | -0,13 | 35,11 |
|  |  |  |  |  |  |  |  |  |  |  |  |  |  |  |  |  | High | TFDNVYFK | 1 |  | 0,0000 | 2,18 | 2 | 1033,49895 | 0,00 | 41,87 |
| **9109** | M5VZV0 | ATP synthase 24 kDa | Uncharacterized protein OS=Prunus persica GN=PRUPE_ppa010675mg PE=4 SV=1 - [M5VZV0_PRUPE] | 99% to GI:645218784 Prunus mume, E value 2e-167 | Prunus mume | Mitochondrion | 01.01-Metabolism/Amino acid | 102,20 | 45,83 | 1 | 14 | 14 | 71 | 240 | 27,8 | 9,11 | High | ITIDPEDPAAVSQYAK | 1 |  | 0,0000 | 3,22 | 2 | 1717,86528 | 0,52 | 43,14 |
|  |  |  |  |  |  |  |  |  |  |  |  |  |  |  |  |  | High | DALEAMETQK | 14 |  | 0,0000 | 3,09 | 2 | 1135,53020 | 0,15 | 40,91 |
|  |  |  |  |  |  |  |  |  |  |  |  |  |  |  |  |  | High | ADLFSESQR | 17 |  | 0,0000 | 2,76 | 2 | 1052,50212 | 1,29 | 39,50 |
|  |  |  |  |  |  |  |  |  |  |  |  |  |  |  |  |  | High | GMALLQAEFEK | 1 |  | 0,0000 | 2,54 | 2 | 1236,62993 | 0,46 | 44,86 |
|  |  |  |  |  |  |  |  |  |  |  |  |  |  |  |  |  | High | DEEMVDVK | 1 |  | 0,0000 | 2,50 | 2 | 964,42919 | -0,07 | 37,87 |
|  |  |  |  |  |  |  |  |  |  |  |  |  |  |  |  |  | High | YEEQLELK | 1 |  | 0,0000 | 2,38 | 2 | 1051,53069 | 0,03 | 38,94 |
|  |  |  |  |  |  |  |  |  |  |  |  |  |  |  |  |  | Medium | EEFKDEEMVDVK | 1 |  | 0,0000 | 2,27 | 3 | 1497,67719 | -0,44 | 38,57 |
|  |  |  |  |  |  |  |  |  |  |  |  |  |  |  |  |  | High | DALEAmETQK | 3 | M6(Oxidation) | 0,0000 | 2,24 | 2 | 1151,52373 | -1,06 | 40,38 |
|  |  |  |  |  |  |  |  |  |  |  |  |  |  |  |  |  | High | AQLEELK | 8 |  | 0,0000 | 2,07 | 2 | 830,46184 | -0,01 | 36,36 |
|  |  |  |  |  |  |  |  |  |  |  |  |  |  |  |  |  | Medium | TqDIPDAR | 1 | Q2(Deamidated) | 0,0000 | 1,85 | 2 | 916,43718 | 0,09 | 35,30 |
|  |  |  |  |  |  |  |  |  |  |  |  |  |  |  |  |  | Medium | TQDIPDAR | 1 |  | 0,0000 | 1,60 | 2 | 915,45281 | -0,30 | 34,35 |
|  |  |  |  |  |  |  |  |  |  |  |  |  |  |  |  |  | Medium | NIFLDVK | 8 |  | 0,0000 | 1,56 | 2 | 848,48802 | 0,42 | 50,19 |
|  |  |  |  |  |  |  |  |  |  |  |  |  |  |  |  |  | Medium | GDEMLK | 1 |  | 0,0000 | 1,55 | 2 | 692,32805 | -0,51 | 34,33 |
|  |  |  |  |  |  |  |  |  |  |  |  |  |  |  |  |  | Medium | IQHTIQSR | 1 |  | 0,0000 | 1,47 | 3 | 982,54328 | 0,40 | 30,49 |
|  |  |  |  |  |  |  |  |  |  |  |  |  |  |  |  |  | Medium | TYLLTLK | 11 |  | 0,0000 | 1,12 | 2 | 851,52324 | -0,54 | 51,49 |
|  |  |  |  |  |  |  |  |  |  |  |  |  |  |  |  |  | Medium | AQLEELKK | 1 |  | 0,0000 | 1,06 | 2 | 958,55718 | 0,37 | 33,41 |
| **9206** | M5XCT3 | 3-hydroxyisobutyryl-CoA hydrolase | Uncharacterized protein OS=Prunus persica GN=PRUPE_ppa007004mg PE=4 SV=1 - [M5XCT3_PRUPE] | 96% to GI:645254927 Prunus mume, E value 0 | Prunus mume | Chloroplast | 01.01-Metabolism/Amino Acid | 60,36 | 22,02 | 6 | 10 | 12 | 40 | 386 | 42,9 | 7,97 | High | AGDEWLASTIR | 1 |  | 0,0000 | 3,39 | 2 | 1218,61162 | 0,19 | 45,18 |
|  |  |  |  |  |  |  |  |  |  |  |  |  |  |  |  |  | High | LDGDEELKLPQR | 4 |  | 0,0000 | 2,85 | 3 | 1412,73740 | -0,47 | 41,18 |
|  |  |  |  |  |  |  |  |  |  |  |  |  |  |  |  |  | High | VQAMDEcLVR | 3 | C7(Carbamidomethyl) | 0,0000 | 2,85 | 2 | 1220,57634 | 0,05 | 41,43 |
|  |  |  |  |  |  |  |  |  |  |  |  |  |  |  |  |  | High | VQAmDEcLVR | 5 | M4(Oxidation); C7(Carbamidomethyl) | 0,0000 | 2,82 | 2 | 1236,57121 | 0,02 | 40,78 |
|  |  |  |  |  |  |  |  |  |  |  |  |  |  |  |  |  | High | AILWDKDK | 4 |  | 0,0000 | 2,59 | 2 | 988,54607 | -0,20 | 39,15 |
|  |  |  |  |  |  |  |  |  |  |  |  |  |  |  |  |  | High | YGENFAQK | 2 |  | 0,0000 | 2,53 | 2 | 956,44713 | -0,13 | 36,45 |
|  |  |  |  |  |  |  |  |  |  |  |  |  |  |  |  |  | High | LDGDEELK | 2 |  | 0,0000 | 2,35 | 2 | 918,44115 | -0,41 | 36,73 |
|  |  |  |  |  |  |  |  |  |  |  |  |  |  |  |  |  | High | HFLEGNLK | 2 |  | 0,0000 | 2,28 | 2 | 957,51488 | -0,42 | 38,94 |
|  |  |  |  |  |  |  |  |  |  |  |  |  |  |  |  |  | Medium | TLTLNRPR | 6 |  | 0,0000 | 1,99 | 2 | 970,57897 | -0,32 | 36,35 |
|  |  |  |  |  |  |  |  |  |  |  |  |  |  |  |  |  | Medium | VFANAKL | 1 |  | 0,0000 | 1,82 | 2 | 762,45091 | 0,03 | 40,05 |
|  |  |  |  |  |  |  |  |  |  |  |  |  |  |  |  |  | Medium | LSLLEK | 6 |  | 0,0000 | 1,66 | 2 | 702,43987 | 0,31 | 41,93 |
|  |  |  |  |  |  |  |  |  |  |  |  |  |  |  |  |  | Medium | AILWDK | 2 |  | 0,0000 | 1,48 | 2 | 745,42443 | 0,11 | 42,25 |
| **9211** | M5Y500 | 3-ketoacyl-CoA thiolase | Uncharacterized protein OS=Prunus persica GN=PRUPE_ppa005421mg PE=3 SV=1 - [M5Y500_PRUPE] | 92% to GI:645236120 Prunus mume, E value 0 | Prunus mume | peroxisome | 01.06-Metabolism/Lipid and sterol | 40,79 | 25,41 | 3 | 10 | 10 | 19 | 429 | 45,0 | 8,09 | High | DGSTTAGNSSQVSDGAGAVLLMK | 2 |  | 0,0000 | 4,27 | 2 | 2166,03765 | 1,49 | 46,89 |
|  |  |  |  |  |  |  |  |  |  |  |  |  |  |  |  |  | High | DGSTTAGNSSQVSDGAGAVLLmK | 3 | M22(Oxidation) | 0,0000 | 3,53 | 2 | 2182,03349 | 1,91 | 43,40 |
|  |  |  |  |  |  |  |  |  |  |  |  |  |  |  |  |  | High | DTHADDLLAPVLK | 2 |  | 0,0000 | 3,14 | 2 | 1407,74809 | 0,14 | 45,90 |
|  |  |  |  |  |  |  |  |  |  |  |  |  |  |  |  |  | High | GDSTDELcNAR | 2 | C8(Carbamidomethyl) | 0,0000 | 2,75 | 2 | 1237,51177 | 0,28 | 37,00 |
|  |  |  |  |  |  |  |  |  |  |  |  |  |  |  |  |  | High | AVATLLHEMK | 1 |  | 0,0000 | 2,69 | 2 | 1112,61406 | 0,68 | 39,93 |
|  |  |  |  |  |  |  |  |  |  |  |  |  |  |  |  |  | High | SGEEKPVTISVDDGIR | 1 |  | 0,0000 | 2,58 | 3 | 1701,86551 | 0,03 | 40,30 |
|  |  |  |  |  |  |  |  |  |  |  |  |  |  |  |  |  | High | LGLDPEK | 2 |  | 0,0000 | 2,12 | 2 | 771,42473 | -0,01 | 39,58 |
|  |  |  |  |  |  |  |  |  |  |  |  |  |  |  |  |  | High | FKDEIIPVATK | 2 |  | 0,0000 | 2,11 | 2 | 1260,71953 | -0,28 | 40,60 |
|  |  |  |  |  |  |  |  |  |  |  |  |  |  |  |  |  | Medium | NTTLADLAK | 1 |  | 0,0000 | 1,99 | 2 | 946,51995 | -0,51 | 40,80 |
|  |  |  |  |  |  |  |  |  |  |  |  |  |  |  |  |  | Medium | DEIIPVATK | 1 |  | 0,0000 | 1,93 | 2 | 985,55614 | -0,36 | 42,88 |
|  |  |  |  |  |  |  |  |  |  |  |  |  |  |  |  |  | Medium | GLPILGVFR | 1 |  | 0,1318 | 1,12 | 2 | 971,60362 | -0,10 | 49,89 |
| **9213** | M5X171 | Aspartic protease in guard cell | Uncharacterized protein OS=Prunus persica GN=PRUPE_ppa005966mg PE=4 SV=1 - [M5X171_PRUPE] | 98% to GI:645251460 Prunus mume, E value 0 | Prunus mume | Nucleus | 01.01-Metabolism/Amino acid | 6,95 | 7,82 | 1 | 4 | 4 | 4 | 435 | 46,9 | 9,11 | High | LVEPAYVAVR | 1 |  | 0,0000 | 2,69 | 2 | 1116,64153 | 0,27 | 40,29 |
|  |  |  |  |  |  |  |  |  |  |  |  |  |  |  |  |  | High | LGPVGQPIR | 1 |  | 0,0000 | 2,13 | 2 | 936,56236 | -0,23 | 37,81 |
|  |  |  |  |  |  |  |  |  |  |  |  |  |  |  |  |  | High | VLIDVPNSR | 1 |  | 0,0000 | 2,12 | 2 | 1012,57842 | -0,20 | 39,90 |
|  |  |  |  |  |  |  |  |  |  |  |  |  |  |  |  |  | Medium | YTPLLK | 1 |  | 0,0000 | 0,97 | 2 | 734,44493 | 0,29 | 38,24 |
| **9214** | M5XYB0 | Aspartate aminotransferase | Aspartate aminotransferase OS=Prunus persica GN=PRUPE_ppa005369mg PE=3 SV=1 - [M5XYB0_PRUPE] |  | Prunus persica | Cytoplasm | 01.01-Metabolism/Amino acid | 34,61 | 20,04 | 1 | 11 | 12 | 27 | 464 | 50,8 | 8,84 | High | LNLGVGAYR | 8 |  | 0,0000 | 2,97 | 2 | 962,54155 | -0,29 | 41,67 |
|  |  |  |  |  |  |  |  |  |  |  |  |  |  |  |  |  | High | AENLmIER | 1 | M5(Oxidation) | 0,0000 | 2,74 | 2 | 991,48705 | -0,73 | 36,59 |
|  |  |  |  |  |  |  |  |  |  |  |  |  |  |  |  |  | High | AEMEMMAGR | 1 |  | 0,0000 | 2,71 | 2 | 1025,42119 | -0,18 | 38,06 |
|  |  |  |  |  |  |  |  |  |  |  |  |  |  |  |  |  | High | LAAALIER | 1 |  | 0,0000 | 2,44 | 2 | 856,52507 | -0,07 | 39,52 |
|  |  |  |  |  |  |  |  |  |  |  |  |  |  |  |  |  | High | IADVIQEK | 2 |  | 0,0000 | 2,41 | 2 | 915,51445 | -0,19 | 37,74 |
|  |  |  |  |  |  |  |  |  |  |  |  |  |  |  |  |  | High | AENLMIER | 1 |  | 0,0000 | 2,40 | 2 | 975,49578 | 3,00 | 38,80 |
|  |  |  |  |  |  |  |  |  |  |  |  |  |  |  |  |  | High | NLGLYAER | 4 |  | 0,0000 | 2,33 | 2 | 935,49443 | -0,12 | 39,09 |
|  |  |  |  |  |  |  |  |  |  |  |  |  |  |  |  |  | High | KAENLMIER | 2 |  | 0,0000 | 2,14 | 2 | 1103,58757 | -0,23 | 35,48 |
|  |  |  |  |  |  |  |  |  |  |  |  |  |  |  |  |  | Medium | TEELQPYVLDVVK | 1 |  | 0,0000 | 1,85 | 2 | 1532,82244 | 1,12 | 45,92 |
|  |  |  |  |  |  |  |  |  |  |  |  |  |  |  |  |  | Medium | LYDNLTAK | 2 |  | 0,0000 | 1,78 | 2 | 937,49877 | -0,20 | 38,03 |
|  |  |  |  |  |  |  |  |  |  |  |  |  |  |  |  |  | Medium | VPWSEYR | 1 |  | 0,0000 | 1,58 | 2 | 936,45702 | -0,43 | 39,62 |
|  |  |  |  |  |  |  |  |  |  |  |  |  |  |  |  |  | Medium | DWSFILK | 1 |  | 0,0000 | 1,43 | 2 | 908,48747 | -0,22 | 49,07 |
|  |  |  |  |  |  |  |  |  |  |  |  |  |  |  |  |  | Medium | NIFNDSR | 1 |  | 0,0000 | 1,38 | 2 | 865,41600 | -0,33 | 38,16 |
|  |  |  |  |  |  |  |  |  |  |  |  |  |  |  |  |  | Medium | KAENLmIER | 1 | M6(Oxidation) | 0,0579 | 1,14 | 2 | 1119,58159 | -1,02 | 33,77 |
| **9216** | M1PYG9 | Aspartate aminotransferase | Aspartate aminotransferase OS=Prunus persica GN=PRUPE_ppa005315mg PE=2 SV=1 - [M1PYG9_PRUPE] |  | Prunus mume | Cytoplasm | 01.01-Metabolism/Amino acid | 78,30 | 19,70 | 3 | 10 | 10 | 36 | 467 | 50,8 | 8,57 | High | LILGADSPPIQENR | 7 |  | 0,0000 | 3,12 | 2 | 1522,82451 | 1,35 | 45,99 |
|  |  |  |  |  |  |  |  |  |  |  |  |  |  |  |  |  | High | VGALSIVcK | 4 | C8(Carbamidomethyl) | 0,0000 | 3,03 | 2 | 946,53868 | -0,40 | 41,10 |
|  |  |  |  |  |  |  |  |  |  |  |  |  |  |  |  |  | High | LNLGVGAYR | 4 |  | 0,0000 | 2,90 | 2 | 962,54149 | -0,35 | 42,91 |
|  |  |  |  |  |  |  |  |  |  |  |  |  |  |  |  |  | High | ISMAGLSSR | 2 |  | 0,0000 | 2,86 | 2 | 921,48204 | -0,26 | 41,09 |
|  |  |  |  |  |  |  |  |  |  |  |  |  |  |  |  |  | High | VGGEFLAK | 4 |  | 0,0000 | 2,60 | 2 | 820,45696 | 0,70 | 40,09 |
|  |  |  |  |  |  |  |  |  |  |  |  |  |  |  |  |  | High | NMGLYGER | 2 |  | 0,0000 | 2,50 | 2 | 939,43541 | 0,10 | 38,69 |
|  |  |  |  |  |  |  |  |  |  |  |  |  |  |  |  |  | High | ISmAGLSSR | 4 | M3(Oxidation) | 0,0000 | 2,36 | 2 | 937,47740 | 0,22 | 36,26 |
|  |  |  |  |  |  |  |  |  |  |  |  |  |  |  |  |  | High | EYHIYMTSDGR | 2 |  | 0,0000 | 2,10 | 2 | 1371,60149 | 1,22 | 39,20 |
|  |  |  |  |  |  |  |  |  |  |  |  |  |  |  |  |  | Medium | HQLFESLR | 1 |  | 0,0000 | 1,91 | 2 | 1029,54692 | -0,70 | 39,87 |
|  |  |  |  |  |  |  |  |  |  |  |  |  |  |  |  |  | Medium | NmGLYGER | 1 | M2(Oxidation) | 0,0000 | 1,87 | 2 | 955,42998 | -0,27 | 39,01 |
|  |  |  |  |  |  |  |  |  |  |  |  |  |  |  |  |  | Medium | EYHIYmTSDGR | 2 | M6(Oxidation) | 0,0000 | 1,80 | 2 | 1387,59502 | 0,21 | 36,87 |
|  |  |  |  |  |  |  |  |  |  |  |  |  |  |  |  |  | Medium | YYDPATR | 2 |  | 0,0000 | 1,76 | 2 | 885,41020 | 0,09 | 37,15 |
|  |  |  |  |  |  |  |  |  |  |  |  |  |  |  |  |  | Medium | AEQLLVNDR | 1 |  | 0,0000 | 1,50 | 2 | 1057,56352 | -0,17 | 39,78 |
| **9217** | M5WTY8 | sulfite oxidase | Uncharacterized protein OS=Prunus persica GN=PRUPE_ppa006835mg PE=4 SV=1 - [M5WTY8_PRUPE] | 99% to GI:645250254 Prunus mume, E value 0 | Prunus mume | Chloroplast | 01.02-Metabolism/Nitrogen and sulphur | 14,78 | 19,34 | 1 | 7 | 7 | 7 | 393 | 43,4 | 8,27 | High | SALVSSYVTPVDFFYK | 1 |  | 0,0000 | 2,90 | 2 | 1822,93010 | 2,12 | 49,50 |
|  |  |  |  |  |  |  |  |  |  |  |  |  |  |  |  |  | High | AVDSAANVQPEK | 1 |  | 0,0000 | 2,88 | 2 | 1228,61687 | 0,00 | 33,80 |
|  |  |  |  |  |  |  |  |  |  |  |  |  |  |  |  |  | High | VEDIWNLR | 1 |  | 0,0000 | 2,84 | 2 | 1044,54692 | -0,38 | 44,56 |
|  |  |  |  |  |  |  |  |  |  |  |  |  |  |  |  |  | High | LADVLELVGIPK | 1 |  | 0,0000 | 2,52 | 2 | 1266,76775 | 0,72 | 49,40 |
|  |  |  |  |  |  |  |  |  |  |  |  |  |  |  |  |  | Medium | HVEFVSVDK | 1 |  | 0,0000 | 1,82 | 2 | 1059,54729 | 0,29 | 36,73 |
|  |  |  |  |  |  |  |  |  |  |  |  |  |  |  |  |  | Medium | VIVPGVIGAR | 1 |  | 0,0000 | 1,82 | 2 | 980,62480 | -0,40 | 41,77 |
|  |  |  |  |  |  |  |  |  |  |  |  |  |  |  |  |  | Medium | HPSLQINAK | 1 |  | 0,0000 | 1,12 | 2 | 1007,56291 | -0,37 | 34,33 |
| **9308** | M5W5P0 | Elongation factor 1-alpha | Elongation factor 1-alpha OS=Prunus persica GN=PRUPE_ppa005717mg PE=3 SV=1 - [M5W5P0_PRUPE] |  | Prunus persica | Cytoplasm | 05.04-Protein synthesis/Translation factors | 46,75 | 17,23 | 4 | 3 | 9 | 22 | 447 | 49,4 | 9,07 | High | YYcTVIDAPGHR | 4 | C3(Carbamidomethyl) | 0,0000 | 3,51 | 2 | 1451,67436 | 0,50 | 38,57 |
|  |  |  |  |  |  |  |  |  |  |  |  |  |  |  |  |  | High | IGGIGTVPVGR | 1 |  | 0,0000 | 2,86 | 2 | 1025,61016 | -0,11 | 41,52 |
|  |  |  |  |  |  |  |  |  |  |  |  |  |  |  |  |  | High | STTTGHLIYK | 5 |  | 0,0000 | 2,80 | 2 | 1120,59953 | -0,17 | 36,12 |
|  |  |  |  |  |  |  |  |  |  |  |  |  |  |  |  |  | High | LPLQDVYK | 3 |  | 0,0000 | 2,47 | 2 | 975,55058 | -0,42 | 42,83 |
|  |  |  |  |  |  |  |  |  |  |  |  |  |  |  |  |  | High | ARYDEIVK | 2 |  | 0,0000 | 2,46 | 2 | 993,53600 | -0,42 | 36,64 |
|  |  |  |  |  |  |  |  |  |  |  |  |  |  |  |  |  | High | STNLDWYK | 2 |  | 0,0000 | 2,22 | 2 | 1026,48918 | 0,07 | 43,36 |
|  |  |  |  |  |  |  |  |  |  |  |  |  |  |  |  |  | Medium | YDEIVK | 2 |  | 0,0000 | 1,85 | 2 | 766,39800 | -0,23 | 37,80 |
|  |  |  |  |  |  |  |  |  |  |  |  |  |  |  |  |  | Medium | EVSSYLK | 2 |  | 0,0000 | 1,63 | 2 | 825,43535 | 0,08 | 37,95 |
| **9701** | M5WQA3 | dynamin-related protein | Uncharacterized protein OS=Prunus persica GN=PRUPE_ppa003029mg PE=3 SV=1 - [M5WQA3_PRUPE] | 99% to GI:645267575 Prunus mume, E value 0 | Prunus mume | Mitochondrion | 03.22-Cell growth/division/Cell cycle | 29,16 | 18,20 | 2 | 11 | 12 | 17 | 610 | 68,1 | 7,97 | High | VEVTNAASDSLER | 2 |  | 0,0000 | 3,05 | 2 | 1390,68181 | 0,63 | 40,00 |
|  |  |  |  |  |  |  |  |  |  |  |  |  |  |  |  |  | High | GTDAAEILEGK | 1 |  | 0,0000 | 2,69 | 2 | 1103,55803 | 0,06 | 42,56 |
|  |  |  |  |  |  |  |  |  |  |  |  |  |  |  |  |  | High | LIESSVITIR | 1 |  | 0,0000 | 2,38 | 2 | 1130,67803 | 0,02 | 43,80 |
|  |  |  |  |  |  |  |  |  |  |  |  |  |  |  |  |  | High | SIVYcQVR | 2 | C5(Carbamidomethyl) | 0,0000 | 2,20 | 2 | 1024,52458 | 0,13 | 38,26 |
|  |  |  |  |  |  |  |  |  |  |  |  |  |  |  |  |  | High | SVNETPELK | 2 |  | 0,0000 | 2,15 | 2 | 1016,52574 | -0,17 | 37,74 |
|  |  |  |  |  |  |  |  |  |  |  |  |  |  |  |  |  | High | HLETVIK | 2 |  | 0,0000 | 2,04 | 2 | 839,49846 | -0,12 | 36,36 |
|  |  |  |  |  |  |  |  |  |  |  |  |  |  |  |  |  | High | YNDSYLR | 1 |  | 0,0000 | 2,00 | 2 | 930,43120 | -0,41 | 38,19 |
|  |  |  |  |  |  |  |  |  |  |  |  |  |  |  |  |  | Medium | EYFSTTPEYK | 1 |  | 0,0000 | 1,78 | 2 | 1264,57414 | 0,72 | 40,72 |
|  |  |  |  |  |  |  |  |  |  |  |  |  |  |  |  |  | Medium | QLSMENIR | 2 |  | 0,0000 | 1,62 | 2 | 990,50377 | 0,03 | 40,21 |
|  |  |  |  |  |  |  |  |  |  |  |  |  |  |  |  |  | Medium | IPGIQSLISK | 1 |  | 0,0000 | 1,50 | 2 | 1055,64653 | 0,53 | 45,93 |
|  |  |  |  |  |  |  |  |  |  |  |  |  |  |  |  |  | Medium | IDLMDK | 1 |  | 0,0199 | 1,48 | 2 | 734,37541 | 0,09 | 40,46 |
|  |  |  |  |  |  |  |  |  |  |  |  |  |  |  |  |  | Medium | LGKPISADAGGK | 1 |  | 0,0000 | 0,99 | 2 | 1113,62627 | -0,05 | 33,77 |
| **9801** | M5WT60 | Monocopper oxidase | Uncharacterized protein OS=Prunus persica GN=PRUPE_ppa003224mg PE=4 SV=1 - [M5WT60_PRUPE] | 99% to GI:645266448 Prunus mume, E value 0 | Prunus mume | Cell wall | 03.01-Cell growth/division/Cell growth | 27,77 | 7,45 | 1 | 4 | 4 | 16 | 591 | 65,8 | 8,28 | High | ASGGFGGFIINNR | 2 |  | 0,0000 | 3,26 | 2 | 1309,66570 | 0,66 | 46,49 |
|  |  |  |  |  |  |  |  |  |  |  |  |  |  |  |  |  | High | NKPPVVInGR | 6 | N8(Deamidated) | 0,0000 | 3,14 | 3 | 1094,63230 | 0,52 | 36,13 |
|  |  |  |  |  |  |  |  |  |  |  |  |  |  |  |  |  | High | AAGPLPEAPNDEFDK | 2 |  | 0,0000 | 2,91 | 2 | 1570,73943 | 0,61 | 42,28 |
|  |  |  |  |  |  |  |  |  |  |  |  |  |  |  |  |  | High | NKPPVVINGR | 4 |  | 0,0000 | 2,84 | 3 | 1093,64774 | 0,03 | 35,73 |
|  |  |  |  |  |  |  |  |  |  |  |  |  |  |  |  |  | Medium | WDGIAR | 2 |  | 0,0000 | 1,81 | 2 | 717,36821 | 0,45 | 39,87 |
| **9802** | M5VVS9 | Eukaryotic translation initiation factor | Uncharacterized protein OS=Prunus persica GN=PRUPE_ppa001554mg PE=4 SV=1 - [M5VVS9_PRUPE] | 99% to GI:645275864 Prunus mume, E value 0 | Prunus mume | Chloroplast | 05.04-Protein synthesis/Translation factors | 19,58 | 19,05 | 2 | 12 | 14 | 15 | 803 | 87,9 | 8,00 | High | QMTSPEQEMER | 1 |  | 0,0000 | 2,51 | 2 | 1365,57842 | 0,74 | 36,73 |
|  |  |  |  |  |  |  |  |  |  |  |  |  |  |  |  |  | High | FPSDEPDGK | 2 |  | 0,0000 | 2,36 | 2 | 991,43694 | 0,17 | 34,49 |
|  |  |  |  |  |  |  |  |  |  |  |  |  |  |  |  |  | High | QQEANQPQFAR | 1 |  | 0,0000 | 2,28 | 2 | 1316,63420 | -0,03 | 34,67 |
|  |  |  |  |  |  |  |  |  |  |  |  |  |  |  |  |  | High | ELTTNPQLAPR | 1 |  | 0,0000 | 2,28 | 2 | 1239,66973 | 0,40 | 37,84 |
|  |  |  |  |  |  |  |  |  |  |  |  |  |  |  |  |  | High | INDLYFSR | 1 |  | 0,0000 | 2,15 | 2 | 1027,52068 | -0,07 | 41,27 |
|  |  |  |  |  |  |  |  |  |  |  |  |  |  |  |  |  | High | APSNFGEVIGK | 1 |  | 0,0000 | 2,13 | 2 | 1118,58415 | 0,04 | 39,98 |
|  |  |  |  |  |  |  |  |  |  |  |  |  |  |  |  |  | High | NNVTGAQGNTSPGGFPNNR | 1 |  | 0,0000 | 2,10 | 2 | 1901,88786 | 1,53 | 36,56 |
|  |  |  |  |  |  |  |  |  |  |  |  |  |  |  |  |  | Medium | SPAYHPEVVK | 1 |  | 0,0000 | 1,93 | 3 | 1126,58933 | 0,14 | 33,72 |
|  |  |  |  |  |  |  |  |  |  |  |  |  |  |  |  |  | Medium | TISEIHTEAEK | 1 |  | 0,0000 | 1,87 | 2 | 1257,63249 | 0,26 | 33,29 |
|  |  |  |  |  |  |  |  |  |  |  |  |  |  |  |  |  | Medium | THNSSNQGGGPAPALTK | 1 |  | 0,0000 | 1,85 | 3 | 1636,80741 | 2,18 | 32,41 |
|  |  |  |  |  |  |  |  |  |  |  |  |  |  |  |  |  | Medium | AEVPWSAR | 1 |  | 0,0000 | 1,48 | 2 | 915,46807 | -0,30 | 39,32 |
|  |  |  |  |  |  |  |  |  |  |  |  |  |  |  |  |  | Medium | VFTASDIR | 1 |  | 0,0000 | 1,35 | 2 | 908,48351 | -0,16 | 38,39 |

^a^*Spot* *N°*, protein spot number of the reference gel maps presented in Figure S1;

*^b^Accession number*, UNIPROT accession number;

^c^*Suggested name*; Suggested protein name according to database identification or manual blast against current databases;

^d^*Matching criteria,* adopted criteria for the valuation of similarity for proteins identified after manual blast against current databases. Blank cells correspond to proteins readily identified without further blast;

*^e^Organism,* organism in which the protein has been matched to by BLAST search;

^f^*Subellular localization,* Sub-cellular localization of proteins was assigned based on database searches;

^g^*Functional category,* proteins ontologically classified into functional categories proposed by Bevan et al., 1998;

**Table S2.** Quantitative data for peach protein spot volumes on 2DE-gels. Protein extracts have been analyzed by two-dimensional electrophoresis and 2D-gels were submitted to image analysis as described in Experimental procedures.

| **Spot N°, spot label on the reference 2DE-map presented in Figure S1;**  **RGC Mean, average of normalized spot volumes of peach cultivar "Royal Glory" Control fruits as obtained from densitometric analysis of individual spots from proteins in 2D gels stained with silver nitrate from three different gels and independent extractions**  **RGC Coef. Var %, Coefficient of variation of normalized spot volumes of peach cultivar "Royal Glory" Control fruits as obtained from densitometric analysis of individual spots from proteins in 2D gels stained with silver nitrate from three different gels and independent extractions**  **RGL Mean, average of normalized spot volumes of peach cultivar "Royal Glory" fruits inoculated with *Monilinia laxa*, as obtained from densitometric analysis of individual spots from proteins in 2D gels stained with silver nitrate from three different gels and independent extractions**  **RGL Coef. Var %, Coefficient of variation of normalized spot volumes of peach cultivar "Royal Glory" fruits inoculated with *M. laxa* as obtained from densitometric analysis of individual spots from proteins in 2D gels stained with silver nitrate from three different gels and independent extractions**  **Ratio RGL Mean/RGC Mean, The RGL (Mean) spot volume divided by the RGC (Mean) spot volume**  **P value RGL/RGC, p value between normalized spot volumes of individual spots from three different gels and independent extractions of peach cultivar "Royal Glory" Control fruits and normalized spot volumes of individual spots from three different gels and independent extractions of peach cultivar "Royal Glory" fruits inoculated with *M. laxa*, as obtained from densitometric analysis of individual spots from proteins in 2D gels stained with silver nitrate from three different gels and independent extractions**  **RGF Mean, average of normalized spot volumes of peach cultivar "Royal Glory" fruits inoculated with *M. fructicola* as obtained from densitometric analysis of individual spots from proteins in 2D gels stained with silver nitrate from three different gels and independent extractions**  **RGF Coef. Var %, Coefficient of variation of normalized spot volumes of peach cultivar "Royal Glory" fruits inoculated with *M. fructicola* fruits as obtained from densitometric analysis of individual spots from proteins in 2D gels stained with silver nitrate from three different gels and independent extractions**  **Ratio RGF Mean/RGC Mean, The RGF (Mean) spot volume divided by the RGC (Mean) spot volume**  **P value RGF/RGC, p value between normalized spot volumes of individual spots from three different gels and independent extractions of peach cultivar "Royal Glory" Control fruits and normalized spot volumes of individual spots from three different gels and independent extractions of peach cultivar "Royal Glory" fruits inoculated with *M. fructicola* as obtained from densitometric analysis of individual spots from proteins in 2D gels stained with silver nitrate from three different gels and independent extractions**  **Ratio RGL Mean/RGF Mean, The RGL (Mean) spot volume divided by the RGF (Mean) spot volume**  **P value RGL/RGF, p value between normalized spot volumes of individual spots from three different gels and independent extractions of peach cultivar "Royal Glory" fruits inoculated with *M. fructicola* and normalized spot volumes of individual spots from three different gels and independent extractions of peach cultivar "Royal Glory" fruits inoculated with *M. laxa* fruits as obtained from densitometric analysis of individual spots from proteins in 2D gels stained with silver nitrate from three different gels and independent extractions**  **RLC Mean, average of normalized spot volumes of peach cultivar "Rich Lady" Control fruits as obtained from densitometric analysis of individual spots from proteins in 2D gels stained with silver nitrate from three different gels and independent extractions**  **RLC Coef. Var %, Coefficient of variation of normalized spot volumes of peach cultivar "Rich Lady" Control fruits as obtained from densitometric analysis of individual spots from proteins in 2D gels stained with silver nitrate from three different gels and independent extractions**  **Ratio RLC Mean/RGC Mean, The RLC (Mean) spot volume divided by the RGC (Mean) spot volume**  **P value RLC/RGC, p value between normalized spot volumes of individual spots from three different gels and independent extractions of peach cultivar "Royal Glory" Control fruits and normalized spot volumes of individual spots from three different gels and independent extractions of peach cultivar "Rich Lady" Control fruits as obtained from densitometric analysis of individual spots from proteins in 2D gels stained with silver nitrate from three different gels and independent extractions**  **RLL Mean, average of normalized spot volumes of peach cultivar "Rich Lady" fruits inoculated with *M. laxa* fruits as obtained from densitometric analysis of individual spots from proteins in 2D gels stained with silver nitrate from three different gels and independent extractions**  **RLL Coef. Var %, Coefficient of variation of normalized spot volumes of peach cultivar "Rich Lady" fruits inoculated with *M. laxa* fruits as obtained from densitometric analysis of individual spots from proteins in 2D gels stained with silver nitrate from three different gels and independent extractions**  **Ratio RLL Mean/RLC Mean, The RLL (Mean) spot volume divided by the RLC (Mean) spot volume**  **P value RLL/RLC, p value between normalized spot volumes of individual spots from three different gels and independent extractions of peach cultivar "Rich Lady" Control fruits and normalized spot volumes of individual spots from three different gels and independent extractions of peach cultivar "Rich Lady" fruits inoculated with *M. laxa* fruits as obtained from densitometric analysis of individual spots from proteins in 2D gels stained with silver nitrate from three different gels and independent extractions**  **Ratio RLL Mean/RGL Mean, The RLL (Mean) spot volume divided by the RGL (Mean) spot volume**  **P value RLL/RGL, p value between normalized spot volumes of individual spots from three different gels and independent extractions of peach cultivar "Royal Glory" fruits inoculated with *M. laxa* fruits and normalized spot volumes of individual spots from three different gels and independent extractions of peach cultivar "Rich Lady" fruits inoculated with *M. laxa* fruits as obtained from densitometric analysis of individual spots from proteins in 2D gels stained with silver nitrate from three different gels and independent extractions**  **RLF Mean, average of normalized spot volumes of peach cultivar "Rich Lady" fruits inoculated with *M. fructicola* fruits fruits as obtained from densitometric analysis of individual spots from proteins in 2D gels stained with silver nitrate from three different gels and independent extractions**  **RLF Coef. Var %, Coefficient of variation of normalized spot volumes of peach cultivar "Rich Lady" fruits inoculated with *M. fructicola* fruits as obtained from densitometric analysis of individual spots from proteins in 2D gels stained with silver nitrate from three different gels and independent extractions**  **Ratio RLF Mean/RLC Mean, The RLF (Mean) spot volume divided by the RLC (Mean) spot volume**  **P value RLF/RLC, p value between normalized spot volumes of individual spots from three different gels and independent extractions of peach cultivar "Rich Lady" Control fruits and normalized spot volumes of individual spots from three different gels and independent extractions of peach cultivar "Rich Lady" fruits inoculated with *M. fructicola* fruits as obtained from densitometric analysis of individual spots from proteins in 2D gels stained with silver nitrate from three different gels and independent extractions**  **Ratio RLF Mean/RGF Mean, The RLF (Mean) spot volume divided by the RGF (Mean) spot volume**  **P value RLF/RGF, p value between normalized spot volumes of individual spots from three different gels and independent extractions of peach cultivar "Royal Glory" fruits inoculated with *M. fructicola* and normalized spot volumes of individual spots from three different gels and independent extractions of peach cultivar "Rich Lady" fruits inoculated with *M. fructicola*, as obtained from densitometric analysis of individual spots from proteins in 2D gels stained with silver nitrate from three different gels and independent extractions**  **Ratio RLL Mean/RLF Mean, The RLL (Mean) spot volume divided by the RLF (Mean) spot volume**  **P value RLL/RLF, p value between normalized spot volumes of individual spots from three different gels and independent extractions of peach cultivar "Rich Lady" fruits inoculated with *M. fructicola* and normalized spot volumes of individual spots from three different gels and independent extractions of peach cultivar "Rich Lady" fruits inoculated with *M. laxa*, as obtained from densitometric analysis of individual spots from proteins in 2D gels stained with silver nitrate from three different gels and independent extractions** | | | | | | | | | | | | | | | | | | | | | | | | | | | | | | |
| --- | --- | --- | --- | --- | --- | --- | --- | --- | --- | --- | --- | --- | --- | --- | --- | --- | --- | --- | --- | --- | --- | --- | --- | --- | --- | --- | --- | --- | --- | --- |
| **Spot N^o^** | **RGC Mean** | **RGC Coef. Var %** | **RGL Mean** | **RGL Coef. Var %** | **Ratio RGL Mean/RGC Mean** | **P value RGL/RGC** | **RGF Mean** | **RGF Coef. Var %** | **Ratio RGF Mean/RGC Mean** | **P value RGF/RGC** | **Ratio RGL Mean/RGF Mean** | **P value RGL/RGF** | **RLC Mean** | **RLC Coef. Var %** | **Ratio RLC Mean/RGC Mean** | **P value RLC/RGC** | **RLL Mean** | **RLL Coef. Var %** | **Ratio RLL Mean/RLC Mean** | **P value RLL/RLC** | **Ratio RLL Mean/RGL Mean** | **P value RLL/RGL** | **RLF Mean** | **RLF Coef. Var %** | **Ratio RLF Mean/RLC Mean** | **P value RLF/RLC** | **Ratio RLF Mean/RGF Mean** | **P value RLF/RGF** | **Ratio RLL Mean/RLF Mean** | **P value RLL/RLF** |
| **13** | 3165,2 | 30,1% | 2786,6 | 15,0% | 0,88 | 0,352 | 2478,7 | 50,0% | 0,78 | 0,119 | 1,12 | 0,322 | 1471,8 | 17,5% | 0,46 | 0,005 | 1063,1 | 49,5% | 0,72 | 0,451 | 0,38 | 0,000 | 1668,1 | 15,4% | 1,13 | 0,734 | 0,67 | 0,059 | 0,64 | 0,178 |
| **19** | 1790,3 | 38,8% | 1437,3 | 47,1% | 0,80 | 0,386 | 3315,4 | 1,8% | 1,85 | 0,001 | 0,43 | 0,000 | 4073,8 | 51,2% | 2,28 | 0,000 | 3188 | 19,7% | 0,78 | 0,104 | 2,22 | 0,000 | 3390,3 | 35,5% | 0,83 | 0,238 | 1,02 | 0,86 | 0,94 | 0,651 |
| **20** | 394,4 | 11,5% | 667,5 | 33,1% | 1,69 | 0,502 | 1115,4 | 75,8% | 2,83 | 0,101 | 0,60 | 0,151 | 569,4 | 32,8% | 1,44 | 0,765 | 1488,8 | 25,5% | 2,61 | 0,092 | 2,23 | 0,017 | 1535,6 | 15,7% | 2,70 | 0,097 | 1,38 | 0,325 | 0,97 | 0,917 |
| **407** | 2971,3 | 29,5% | 1959,2 | 12,7% | 0,66 | 0,014 | 1369,7 | 34,1% | 0,46 | 0,000 | 1,43 | 0,060 | 2568,8 | 52,5% | 0,86 | 0,493 | 1081,2 | 9,2% | 0,42 | 0,007 | 0,55 | 0,011 | 932,3 | 84,3% | 0,36 | 0,005 | 0,68 | 0,305 | 1,16 | 0,739 |
| **812** | 820,5 | 51,7% | 1358,4 | 17,8% | 1,66 | 0,187 | 1264,8 | 29,6% | 1,54 | 0,311 | 1,07 | 0,763 | 867,8 | 37,9% | 1,06 | 0,936 | 1422,7 | 38,5% | 1,64 | 0,307 | 1,05 | 0,850 | 2057,4 | 22,7% | 2,37 | 0,042 | 1,63 | 0,065 | 0,69 | 0,158 |
| **1013** | 406,9 | 55,4% | 771,6 | 16,6% | 1,90 | 0,370 | 1353,8 | 56,3% | 3,33 | 0,032 | 0,57 | 0,063 | 1628,7 | 42,5% | 4,00 | 0,039 | 1439,1 | 23,9% | 0,88 | 0,727 | 1,87 | 0,051 | 1349 | 56,1% | 0,83 | 0,628 | 1 | 0,991 | 1,07 | 0,840 |
| **1109** | 1537,7 | 133,2% | 884,1 | 23,7% | 0,57 | 0,110 | 548,9 | 30,0% | 0,36 | 0,025 | 1,61 | 0,282 | 440,1 | 126,1% | 0,29 | 0,063 | 382,4 | 42,4% | 0,87 | 0,915 | 0,43 | 0,141 | 578,6 | 48,5% | 1,31 | 0,811 | 1,05 | 0,944 | 0,66 | 0,661 |
| **1119** | 4389,9 | 30,6% | 3986,7 | 15,5% | 0,91 | 0,322 | 4107,4 | 19,8% | 0,94 | 0,519 | 0,97 | 0,697 | 8230,5 | 28,4% | 1,87 | 0,000 | 3452,6 | 9,8% | 0,42 | 0,000 | 0,87 | 0,118 | 2881,1 | 23,1% | 0,35 | 0,000 | 0,7 | 0,005 | 1,20 | 0,203 |
| **1314** | 476,4 | 75,1% | 608,6 | 46,2% | 1,28 | 0,745 | 438,5 | 16,2% | 0,92 | 0,931 | 1,39 | 0,584 | 1185,8 | 19,3% | 2,49 | 0,228 | 1120,3 | 23,2% | 0,94 | 0,904 | 1,84 | 0,134 | 1012,6 | 48,1% | 0,85 | 0,764 | 2,31 | 0,179 | 1,11 | 0,810 |
| **1319** | 350,1 | 36,4% | 897,9 | 35,4% | 2,56 | 0,180 | 699 | 27,1% | 2,00 | 0,425 | 1,28 | 0,522 | 1656,2 | 15,4% | 4,73 | 0,028 | 3303,5 | 5,9% | 1,99 | 0,003 | 3,68 | 0,000 | 2627 | 24,2% | 1,59 | 0,095 | 3,76 | 0 | 1,26 | 0,132 |
| **1321** | 459,7 | 25,5% | 614,6 | 19,0% | 1,34 | 0,703 | 760,6 | 26,4% | 1,65 | 0,492 | 0,81 | 0,638 | 829 | 28,2% | 1,80 | 0,529 | 1887,9 | 26,6% | 2,28 | 0,053 | 3,07 | 0,000 | 1046,3 | 6,6% | 1,26 | 0,707 | 1,38 | 0,503 | 1,80 | 0,062 |
| **1628** | 357,2 | 23,2% | 519,3 | 46,6% | 1,45 | 0,690 | 550,3 | 28,5% | 1,54 | 0,659 | 0,94 | 0,921 | 951,4 | 38,4% | 2,66 | 0,312 | 1452,6 | 19,1% | 1,53 | 0,356 | 2,80 | 0,007 | 1050,5 | 18,3% | 1,10 | 0,864 | 1,91 | 0,241 | 1,38 | 0,369 |
| **1705** | 313,6 | 25,3% | 670,6 | 36,1% | 2,14 | 0,381 | 715,8 | 32,1% | 2,28 | 0,358 | 0,94 | 0,884 | 414,3 | 37,2% | 1,32 | 0,864 | 285,4 | 37,9% | 0,69 | 0,812 | 0,43 | 0,258 | 406,2 | 31,6% | 0,98 | 0,989 | 0,57 | 0,468 | 0,70 | 0,787 |
| **1820** | 245,9 | 30,0% | 363 | 32,2% | 1,48 | 0,773 | 418,4 | 11,0% | 1,70 | 0,693 | 0,87 | 0,859 | 537,1 | 31,4% | 2,18 | 0,620 | 792,8 | 23,5% | 1,48 | 0,637 | 2,18 | 0,207 | 729,4 | 12,0% | 1,36 | 0,739 | 1,74 | 0,466 | 1,09 | 0,887 |
| **1821** | 84,7 | 26,3% | 498,9 | 1,7% | 5,89 | 0,309 | 362 | 46,4% | 4,27 | 0,526 | 1,38 | 0,659 | 392 | 15,6% | 4,63 | 0,600 | 572,3 | 25,2% | 1,46 | 0,739 | 1,15 | 0,829 | 409,6 | 10,5% | 1,04 | 0,976 | 1,13 | 0,911 | 1,40 | 0,716 |
| **2007** | 1179,2 | 2,7% | 1009,9 | 49,3% | 0,86 | 0,677 | 1349,2 | 22,3% | 1,14 | 0,697 | 0,75 | 0,276 | 1983 | 34,2% | 1,68 | 0,172 | 3588,5 | 17,4% | 1,81 | 0,004 | 3,55 | 0,000 | 4008,5 | 46,4% | 2,02 | 0,001 | 2,97 | 0 | 0,90 | 0,349 |
| **2104** | 2573,1 | 36,1% | 1001,4 | 69,9% | 0,39 | 0,000 | 1120,4 | 43,3% | 0,44 | 0,001 | 0,89 | 0,701 | 634,6 | 91,3% | 0,25 | 0,001 | 1535,1 | 107,2% | 2,42 | 0,099 | 1,53 | 0,118 | 1141,4 | 38,0% | 1,80 | 0,381 | 1,02 | 0,961 | 1,34 | 0,379 |
| **2311** | 648,3 | 27,1% | 527,8 | 55,6% | 0,81 | 0,767 | 570 | 7,1% | 0,88 | 0,858 | 0,93 | 0,892 | 3202,8 | 36,6% | 4,94 | 0,000 | 3327,1 | 24,9% | 1,04 | 0,819 | 6,30 | 0,000 | 2861,8 | 25,0% | 0,89 | 0,555 | 5,02 | 0 | 1,16 | 0,299 |
| **2312** | 645,1 | 28,5% | 798 | 20,3% | 1,24 | 0,707 | 442,3 | 13,4% | 0,69 | 0,643 | 1,80 | 0,253 | 2556,9 | 28,5% | 3,96 | 0,001 | 2737,2 | 14,1% | 1,07 | 0,739 | 3,43 | 0,000 | 2509,7 | 2,0% | 0,98 | 0,935 | 5,67 | 0 | 1,09 | 0,611 |
| **2319** | 834,6 | 35,2% | 1197,7 | 14,6% | 1,44 | 0,372 | 1159,1 | 15,1% | 1,39 | 0,458 | 1,03 | 0,901 | 794,7 | 43,2% | 0,95 | 0,946 | 1960,9 | 8,4% | 2,47 | 0,033 | 1,64 | 0,026 | 1755,1 | 12,3% | 2,21 | 0,099 | 1,51 | 0,163 | 1,12 | 0,645 |
| **2609** | 300,5 | 61,0% | 560,8 | 25,9% | 1,87 | 0,522 | 375,1 | 3,7% | 1,25 | 0,864 | 1,50 | 0,550 | 352,4 | 48,5% | 1,17 | 0,929 | 209 | 42,3% | 0,59 | 0,791 | 0,37 | 0,301 | 401,3 | 36,5% | 1,14 | 0,932 | 1,07 | 0,951 | 0,52 | 0,667 |
| **2617** | 448,6 | 31,1% | 450,9 | 26,5% | 1,01 | 0,995 | 346,7 | 29,1% | 0,77 | 0,816 | 1,30 | 0,737 | 446,9 | 35,4% | 1,00 | 0,998 | 244,6 | 8,3% | 0,55 | 0,709 | 0,54 | 0,543 | 543,8 | 24,4% | 1,22 | 0,867 | 1,57 | 0,643 | 0,45 | 0,504 |
| **2701** | 177,2 | 47,1% | 501,5 | 26,9% | 2,83 | 0,425 | 543,5 | 24,9% | 3,07 | 0,403 | 0,92 | 0,892 | 332,6 | 25,9% | 1,88 | 0,791 | 431,2 | 42,6% | 1,30 | 0,856 | 0,86 | 0,836 | 568,7 | 64,1% | 1,71 | 0,683 | 1,05 | 0,953 | 0,76 | 0,758 |
| **2703** | 424,4 | 43,0% | 1020,8 | 28,8% | 2,41 | 0,144 | 951,1 | 28,0% | 2,24 | 0,230 | 1,07 | 0,822 | 751,8 | 5,8% | 1,77 | 0,577 | 961,1 | 19,3% | 1,28 | 0,699 | 0,94 | 0,860 | 1172,1 | 42,0% | 1,56 | 0,467 | 1,23 | 0,604 | 0,82 | 0,637 |
| **2704** | 840,3 | 13,7% | 1249,7 | 15,6% | 1,49 | 0,315 | 1193,1 | 14,6% | 1,42 | 0,420 | 1,05 | 0,855 | 716,8 | 34,1% | 0,85 | 0,833 | 576,6 | 33,8% | 0,80 | 0,796 | 0,46 | 0,049 | 837,1 | 42,1% | 1,17 | 0,835 | 0,7 | 0,404 | 0,69 | 0,560 |
| **3205** | 1816,1 | 32,5% | 870,7 | 50,0% | 0,48 | 0,000 | 1173,1 | 54,5% | 0,65 | 0,017 | 0,74 | 0,242 | 1564,1 | 20,8% | 0,86 | 0,561 | 1295,4 | 30,0% | 0,83 | 0,574 | 1,49 | 0,161 | 772,5 | 42,1% | 0,49 | 0,096 | 0,66 | 0,222 | 1,68 | 0,151 |
| **3308** | 1302,8 | 29,7% | 591,4 | 13,3% | 0,45 | 0,002 | 712,9 | 48,2% | 0,55 | 0,028 | 0,83 | 0,637 | 994,9 | 44,3% | 0,76 | 0,477 | 901 | 15,0% | 0,91 | 0,844 | 1,52 | 0,305 | 1071,3 | 31,7% | 1,08 | 0,871 | 1,5 | 0,274 | 0,84 | 0,638 |
| **3311** | 782,3 | 27,6% | 1773,5 | 17,4% | 2,27 | 0,000 | 1477,5 | 47,0% | 1,89 | 0,010 | 1,20 | 0,252 | 1097 | 71,6% | 1,40 | 0,467 | 2560 | 22,3% | 2,33 | 0,003 | 1,44 | 0,010 | 1287,3 | 25,2% | 1,17 | 0,687 | 0,87 | 0,561 | 1,99 | 0,001 |
| **3314** | 342,1 | 78,1% | 406,7 | 92,4% | 1,19 | 0,773 | 209 | 36,5% | 0,61 | 0,617 | 1,95 | 0,443 | 2512 | 14,9% | 7,34 | 0,000 | 2145 | 22,5% | 0,85 | 0,443 | 5,27 | 0,000 | 1666,3 | 22,5% | 0,66 | 0,076 | 7,97 | 0 | 1,29 | 0,188 |
| **3316** | 233,6 | 31,7% | 449,6 | 40,2% | 1,92 | 0,335 | 326,9 | 27,0% | 1,40 | 0,726 | 1,38 | 0,634 | 4043,4 | 36,3% | 17,31 | 0,000 | 3057,9 | 8,5% | 0,76 | 0,041 | 6,80 | 0,000 | 3346,9 | 18,3% | 0,83 | 0,142 | 10,24 | 0 | 0,91 | 0,426 |
| **3518** | 742 | 20,8% | 751,4 | 32,9% | 1,01 | 0,966 | 521,5 | 31,8% | 0,70 | 0,408 | 1,44 | 0,373 | 349,9 | 12,7% | 0,47 | 0,366 | 309,5 | 47,9% | 0,88 | 0,933 | 0,41 | 0,145 | 326,7 | 1,5% | 0,93 | 0,961 | 0,63 | 0,551 | 0,95 | 0,962 |
| **3616** | 337,6 | 33,2% | 644 | 40,6% | 1,91 | 0,173 | 559,3 | 15,4% | 1,66 | 0,406 | 1,15 | 0,742 | 1154,5 | 6,4% | 3,42 | 0,061 | 1048,8 | 31,5% | 0,91 | 0,825 | 1,63 | 0,181 | 858 | 21,6% | 0,74 | 0,530 | 1,53 | 0,362 | 1,22 | 0,599 |
| **4208** | 1098,2 | 36,3% | 422,4 | 58,5% | 0,38 | 0,003 | 614,5 | 52,6% | 0,56 | 0,071 | 0,69 | 0,456 | 5784,8 | 39,2% | 5,27 | 0,000 | 5818,7 | 18,3% | 1,01 | 0,944 | 13,78 | 0,000 | 4580,8 | 16,5% | 0,79 | 0,012 | 7,45 | 0 | 1,27 | 0,001 |
| **4209** | 205,2 | 44,1% | 236,5 | 71,5% | 1,15 | 0,889 | 300,6 | 58,7% | 1,46 | 0,720 | 0,79 | 0,000 | 5067,1 | 29,6% | 24,69 | 0,000 | 4083,7 | 24,2% | 0,81 | 0,041 | 17,27 | 0,000 | 2001 | 47,4% | 0,39 | 0,000 | 6,66 | 0 | 2,04 | 0,000 |
| **4211** | 320,3 | 39,0% | 181,4 | 91,8% | 0,57 | 0,535 | 276,4 | 65,6% | 0,86 | 0,869 | 0,66 | 0,712 | 884,7 | 46,0% | 2,76 | 0,194 | 1743,9 | 32,0% | 1,97 | 0,074 | 9,61 | 0,000 | 1147,4 | 50,2% | 1,30 | 0,578 | 4,15 | 0,009 | 1,52 | 0,102 |
| **4214** | 192,3 | 47,1% | 243,7 | 100,1% | 1,27 | 0,818 | 359,9 | 79,0% | 1,87 | 0,529 | 0,68 | 0,652 | 433,4 | 18,6% | 2,25 | 0,578 | 862,4 | 30,3% | 1,99 | 0,370 | 3,54 | 0,042 | 631,4 | 42,4% | 1,46 | 0,675 | 1,75 | 0,406 | 1,37 | 0,524 |
| **4215** | 295,9 | 37,2% | 560,3 | 44,3% | 1,89 | 0,239 | 485,4 | 38,4% | 1,64 | 0,477 | 1,15 | 0,771 | 611,6 | 54,5% | 2,07 | 0,466 | 2540,9 | 29,7% | 4,15 | 0,000 | 4,53 | 0,000 | 881,9 | 114,5% | 1,44 | 0,567 | 1,82 | 0,226 | 2,88 | 0,000 |
| **4313** | 2169 | 5,4% | 919,8 | 36,3% | 0,42 | 0,000 | 1306,4 | 53,2% | 0,60 | 0,002 | 0,70 | 0,135 | 2026,2 | 29,0% | 0,93 | 0,741 | 1824,3 | 12,8% | 0,90 | 0,673 | 1,98 | 0,003 | 1733,6 | 9,5% | 0,86 | 0,536 | 1,33 | 0,193 | 1,05 | 0,802 |
| **4421** | 312,9 | 43,7% | 453,7 | 5,8% | 1,45 | 0,529 | 321,8 | 29,3% | 1,03 | 0,973 | 1,41 | 0,609 | 301,1 | 24,4% | 0,96 | 0,978 | 896,2 | 12,6% | 2,98 | 0,214 | 1,98 | 0,144 | 1019,3 | 20,4% | 3,39 | 0,131 | 3,17 | 0,035 | 0,88 | 0,734 |
| **4711** | 137,6 | 49,1% | 232,3 | 34,2% | 1,69 | 0,672 | 179,9 | 48,9% | 1,31 | 0,874 | 1,29 | 0,839 | 167,5 | 115,3% | 1,22 | 0,945 | 36,2 | 59,9% | 0,22 | 0,783 | 0,16 | 0,516 | 66,7 | 70,9% | 0,40 | 0,831 | 0,37 | 0,729 | 0,54 | 0,933 |
| **4726** | 413 | 24,8% | 684,6 | 28,6% | 1,66 | 0,226 | 559,2 | 30,3% | 1,35 | 0,583 | 1,22 | 0,626 | 460,1 | 33,9% | 1,11 | 0,913 | 316,8 | 25,9% | 0,69 | 0,764 | 0,46 | 0,224 | 362,8 | 57,8% | 0,79 | 0,837 | 0,65 | 0,548 | 0,87 | 0,899 |
| **4805** | 159,1 | 49,5% | 159,2 | 1,5% | 1,00 | 1,000 | 169,4 | 6,0% | 1,06 | 0,969 | 0,94 | 0,968 | 369,2 | 27,2% | 2,32 | 0,628 | 402,4 | 39,3% | 1,09 | 0,945 | 2,53 | 0,420 | 371,6 | 39,5% | 1,01 | 0,996 | 2,19 | 0,536 | 1,08 | 0,932 |
| **4828** | 373,5 | 23,6% | 990,3 | 43,1% | 2,65 | 0,007 | 897,8 | 27,9% | 2,40 | 0,051 | 1,10 | 0,719 | 569,6 | 21,5% | 1,53 | 0,651 | 1181,7 | 23,2% | 2,07 | 0,202 | 1,19 | 0,526 | 758,4 | 20,5% | 1,33 | 0,689 | 0,84 | 0,67 | 1,56 | 0,244 |
| **4829** | 384,4 | 40,0% | 1252 | 36,0% | 3,26 | 0,000 | 1228,5 | 51,2% | 3,20 | 0,000 | 1,02 | 0,927 | 767,8 | 31,4% | 2,00 | 0,376 | 1586,7 | 47,5% | 2,07 | 0,089 | 1,27 | 0,268 | 1543,7 | 33,3% | 2,01 | 0,103 | 1,26 | 0,336 | 1,03 | 0,905 |
| **5008** | 3,3 | 88,8% | 1602,3 | 25,9% | 485,55 | 0,000 | 1686,8 | 52,5% | 511,15 | 0,000 | 0,95 | 0,743 | 1171,3 | 56,8% | 354,94 | 0,008 | 1941,8 | 26,8% | 1,66 | 0,109 | 1,21 | 0,261 | 3305,2 | 16,3% | 2,82 | 0,000 | 1,96 | 0 | 0,59 | 0,000 |
| **5414** | 1256,9 | 26,3% | 630,2 | 32,4% | 0,50 | 0,006 | 464,1 | 30,9% | 0,37 | 0,004 | 1,36 | 0,519 | 1379,5 | 7,3% | 1,10 | 0,777 | 984,1 | 10,4% | 0,71 | 0,408 | 1,56 | 0,242 | 1028,1 | 29,1% | 0,75 | 0,458 | 2,22 | 0,086 | 0,96 | 0,903 |
| **5509** | 944,1 | 25,9% | 379,1 | 32,2% | 0,40 | 0,013 | 609,8 | 26,9% | 0,65 | 0,211 | 0,62 | 0,371 | 459,9 | 36,4% | 0,49 | 0,264 | 464,4 | 29,9% | 1,01 | 0,993 | 1,23 | 0,777 | 467,7 | 23,1% | 1,02 | 0,987 | 0,77 | 0,664 | 0,99 | 0,993 |
| **5615** | 749,1 | 126,4% | 205,4 | 61,1% | 0,27 | 0,017 | 180,3 | 40,8% | 0,24 | 0,035 | 1,14 | 0,922 | 507,5 | 32,6% | 0,68 | 0,577 | 442,5 | 43,1% | 0,87 | 0,892 | 2,15 | 0,432 | 432,7 | 15,0% | 0,85 | 0,874 | 2,4 | 0,44 | 1,02 | 0,979 |
| **5703** | 472,1 | 24,6% | 728,9 | 37,3% | 1,54 | 0,252 | 933,9 | 30,1% | 1,98 | 0,085 | 0,78 | 0,427 | 202,8 | 45,2% | 0,43 | 0,534 | 186,3 | 69,8% | 0,92 | 0,972 | 0,26 | 0,074 | 338,1 | 101,0% | 1,67 | 0,775 | 0,36 | 0,07 | 0,55 | 0,675 |
| **5705** | 444,2 | 23,5% | 315,3 | 14,7% | 0,71 | 0,565 | 282,9 | 24,2% | 0,64 | 0,545 | 1,11 | 0,900 | 106,1 | 88,9% | 0,24 | 0,435 | 89,2 | 33,1% | 0,84 | 0,972 | 0,28 | 0,454 | 84,5 | 93,3% | 0,80 | 0,964 | 0,3 | 0,544 | 1,06 | 0,990 |
| **5707** | 257,4 | 66,1% | 148,5 | 28,7% | 0,58 | 0,572 | 175,4 | 33,1% | 0,68 | 0,757 | 0,85 | 0,909 | 119,5 | 99,4% | 0,46 | 0,660 | 25,8 | 76,1% | 0,22 | 0,765 | 0,17 | 0,526 | 27,8 | 73,8% | 0,23 | 0,786 | 0,16 | 0,616 | 0,93 | 0,994 |
| **5712** | 4,2 | 60,2% | 4,9 | 131,4% | 1,17 | 0,997 | 2,9 | 79,6% | 0,69 | 0,996 | 1,69 | 0,993 | 200,2 | 98,3% | 47,67 | 0,532 | 67,3 | 33,9% | 0,34 | 0,672 | 13,73 | 0,747 | 113,8 | 11,1% | 0,57 | 0,798 | 39,24 | 0,707 | 0,59 | 0,859 |
| **6013** | 480,8 | 11,4% | 358,8 | 61,3% | 0,75 | 0,527 | 478,8 | 63,2% | 1,00 | 0,994 | 0,75 | 0,611 | 162,4 | 18,5% | 0,34 | 0,311 | 165 | 26,2% | 1,02 | 0,993 | 0,46 | 0,317 | 155,5 | 15,0% | 0,96 | 0,984 | 0,32 | 0,273 | 1,06 | 0,971 |
| **6101** | 1971 | 32,3% | 406,4 | 25,3% | 0,21 | 0,000 | 600,6 | 55,8% | 0,30 | 0,000 | 0,68 | 0,411 | 592,7 | 46,6% | 0,30 | 0,000 | 334,8 | 57,1% | 0,56 | 0,412 | 0,82 | 0,711 | 324,4 | 82,4% | 0,55 | 0,429 | 0,54 | 0,349 | 1,03 | 0,968 |
| **6206** | 2375,5 | 31,9% | 3561,9 | 12,7% | 1,50 | 0,000 | 3073,5 | 34,9% | 1,29 | 0,010 | 1,16 | 0,040 | 2671,8 | 27,1% | 1,12 | 0,345 | 5358,8 | 17,9% | 2,01 | 0,000 | 1,50 | 0,000 | 4333,9 | 35,9% | 1,62 | 0,000 | 1,41 | 0 | 1,24 | 0,000 |
| **6220** | 758,3 | 42,7% | 1755,1 | 8,3% | 2,31 | 0,000 | 2085 | 52,9% | 2,75 | 0,000 | 0,84 | 0,164 | 3094,9 | 58,9% | 4,08 | 0,000 | 835 | 30,2% | 0,27 | 0,000 | 0,48 | 0,000 | 1112,1 | 7,3% | 0,36 | 0,000 | 0,53 | 0,001 | 0,75 | 0,291 |
| **6413** | 1087,3 | 30,2% | 440 | 13,9% | 0,40 | 0,001 | 422,8 | 31,5% | 0,39 | 0,014 | 1,04 | 0,942 | 466,3 | 13,1% | 0,43 | 0,050 | 202,1 | 33,2% | 0,43 | 0,400 | 0,46 | 0,220 | 459,3 | 30,5% | 0,98 | 0,983 | 1,09 | 0,901 | 0,44 | 0,327 |
| **6419** | 2018,5 | 30,3% | 933,1 | 31,8% | 0,46 | 0,000 | 1021,4 | 43,2% | 0,51 | 0,000 | 0,91 | 0,708 | 793,5 | 34,8% | 0,39 | 0,000 | 277 | 12,8% | 0,35 | 0,102 | 0,30 | 0,001 | 291 | 36,0% | 0,37 | 0,140 | 0,28 | 0,015 | 0,95 | 0,957 |
| **6708** | 363,7 | 26,7% | 308,1 | 37,3% | 0,85 | 0,773 | 206,5 | 34,3% | 0,57 | 0,554 | 1,49 | 0,666 | 101,2 | 61,3% | 0,28 | 0,403 | 32,3 | 38,1% | 0,32 | 0,826 | 0,10 | 0,156 | 69,7 | 72,0% | 0,69 | 0,926 | 0,34 | 0,642 | 0,46 | 0,886 |
| **6709** | 414,7 | 17,4% | 127,2 | 22,5% | 0,31 | 0,138 | 107,5 | 40,9% | 0,26 | 0,248 | 1,18 | 0,934 | 79 | 62,8% | 0,19 | 0,285 | 3,6 | 22,5% | 0,05 | 0,810 | 0,03 | 0,523 | 3 | 20,6% | 0,04 | 0,822 | 0,03 | 0,723 | 1,20 | 0,998 |
| **7004** | 438,2 | 30,9% | 765,8 | 19,5% | 1,75 | 0,091 | 1326,9 | 25,2% | 3,03 | 0,001 | 0,58 | 0,019 | 2813,6 | 19,9% | 6,42 | 0,000 | 2294,2 | 28,5% | 0,82 | 0,100 | 3,00 | 0,000 | 2601,9 | 12,0% | 0,92 | 0,532 | 1,96 | 0 | 0,88 | 0,241 |
| **7106** | 589,5 | 18,4% | 581,3 | 33,8% | 0,99 | 0,966 | 974,6 | 7,3% | 1,65 | 0,148 | 0,60 | 0,097 | 1922,8 | 23,2% | 3,26 | 0,000 | 1229,7 | 18,1% | 0,64 | 0,029 | 2,12 | 0,001 | 1316,3 | 20,5% | 0,68 | 0,075 | 1,35 | 0,247 | 0,93 | 0,741 |
| **7109** | 965,4 | 9,3% | 435,3 | 17,6% | 0,45 | 0,007 | 765,3 | 22,7% | 0,79 | 0,451 | 0,57 | 0,163 | 609,5 | 32,3% | 0,63 | 0,258 | 227,8 | 9,2% | 0,37 | 0,225 | 0,52 | 0,284 | 305,2 | 44,8% | 0,50 | 0,370 | 0,4 | 0,12 | 0,75 | 0,767 |
| **7113** | 2068 | 3,4% | 1890,8 | 13,4% | 0,91 | 0,358 | 1536,6 | 7,7% | 0,74 | 0,047 | 1,23 | 0,135 | 1035,6 | 32,0% | 0,50 | 0,001 | 885,7 | 25,4% | 0,86 | 0,633 | 0,47 | 0,000 | 783,9 | 4,0% | 0,76 | 0,458 | 0,51 | 0,012 | 1,13 | 0,697 |
| **7202** | 525 | 28,6% | 799,4 | 19,1% | 1,52 | 0,156 | 689,8 | 33,7% | 1,31 | 0,534 | 1,16 | 0,642 | 1097,3 | 22,3% | 2,09 | 0,070 | 829,9 | 41,9% | 0,76 | 0,395 | 1,04 | 0,875 | 579,7 | 67,3% | 0,53 | 0,128 | 0,84 | 0,708 | 1,43 | 0,340 |
| **7212** | 748,4 | 32,4% | 1040,3 | 44,1% | 1,39 | 0,132 | 1360,2 | 8,3% | 1,82 | 0,023 | 0,76 | 0,177 | 2014,6 | 32,9% | 2,69 | 0,000 | 1421,7 | 22,2% | 0,71 | 0,061 | 1,37 | 0,051 | 934,9 | 25,8% | 0,46 | 0,002 | 0,69 | 0,151 | 1,52 | 0,065 |
| **7301** | 171,5 | 40,4% | 583,8 | 6,8% | 3,40 | 0,034 | 691,2 | 44,0% | 4,03 | 0,052 | 0,84 | 0,649 | 1223,3 | 13,2% | 7,13 | 0,001 | 1380,9 | 13,7% | 1,13 | 0,616 | 2,37 | 0,000 | 1502,6 | 19,4% | 1,23 | 0,410 | 2,17 | 0,007 | 0,92 | 0,642 |
| **7302** | 406,5 | 55,6% | 1076,7 | 23,1% | 2,65 | 0,001 | 1334,4 | 21,8% | 3,28 | 0,001 | 0,81 | 0,276 | 1159,2 | 18,6% | 2,85 | 0,018 | 1583,4 | 20,9% | 1,37 | 0,178 | 1,47 | 0,010 | 1134,5 | 32,3% | 0,98 | 0,942 | 0,85 | 0,497 | 1,40 | 0,088 |
| **7308** | 674,6 | 20,0% | 279,5 | 22,1% | 0,41 | 0,042 | 424 | 35,1% | 0,63 | 0,346 | 0,66 | 0,540 | 219,1 | 41,7% | 0,32 | 0,148 | 168,5 | 50,4% | 0,77 | 0,872 | 0,60 | 0,566 | 127,8 | 19,5% | 0,58 | 0,787 | 0,3 | 0,315 | 1,32 | 0,876 |
| **7511** | 174 | 38,8% | 56,6 | 121,7% | 0,33 | 0,543 | 63,6 | 18,0% | 0,37 | 0,677 | 0,89 | 0,976 | 46,6 | 77,7% | 0,27 | 0,684 | 9,8 | 62,8% | 0,21 | 0,907 | 0,17 | 0,809 | 7,9 | 47,3% | 0,17 | 0,909 | 0,12 | 0,85 | 1,24 | 0,994 |
| **7609** | 220 | 34,0% | 123,8 | 44,2% | 0,56 | 0,618 | 98,8 | 20,3% | 0,45 | 0,648 | 1,25 | 0,916 | 77,5 | 51,5% | 0,35 | 0,650 | 19,6 | 14,5% | 0,25 | 0,854 | 0,16 | 0,590 | 32,7 | 19,6% | 0,42 | 0,895 | 0,33 | 0,822 | 0,60 | 0,960 |
| **7702** | 1619,9 | 18,9% | 921,7 | 0,7% | 0,57 | 0,000 | 835,8 | 33,3% | 0,52 | 0,004 | 1,10 | 0,715 | 515,9 | 51,5% | 0,32 | 0,001 | 69,2 | 81,3% | 0,13 | 0,157 | 0,08 | 0,000 | 68,4 | 80,7% | 0,13 | 0,188 | 0,08 | 0,01 | 1,01 | 0,998 |
| **7704** | 318,9 | 28,9% | 266 | 16,6% | 0,83 | 0,784 | 293 | 26,7% | 0,92 | 0,922 | 0,91 | 0,909 | 85 | 46,9% | 0,27 | 0,456 | 31,4 | 61,4% | 0,37 | 0,864 | 0,12 | 0,226 | 58,2 | 69,9% | 0,68 | 0,937 | 0,2 | 0,426 | 0,54 | 0,918 |
| **7707** | 581,2 | 15,8% | 233,5 | 20,1% | 0,40 | 0,073 | 190,8 | 22,2% | 0,33 | 0,143 | 1,22 | 0,856 | 175,2 | 55,8% | 0,30 | 0,197 | 55,6 | 69,9% | 0,32 | 0,703 | 0,24 | 0,358 | 103,1 | 26,2% | 0,59 | 0,831 | 0,54 | 0,766 | 0,54 | 0,856 |
| **7709** | 543,4 | 7,6% | 164,2 | 8,4% | 0,30 | 0,051 | 138,3 | 26,7% | 0,25 | 0,129 | 1,19 | 0,913 | 175,4 | 36,4% | 0,32 | 0,242 | 172,9 | 63,9% | 0,99 | 0,994 | 1,05 | 0,964 | 146,2 | 10,8% | 0,83 | 0,931 | 1,06 | 0,979 | 1,18 | 0,919 |
| **7716** | 553,8 | 14,9% | 219,8 | 27,7% | 0,40 | 0,106 | 236,6 | 42,8% | 0,43 | 0,094 | 0,93 | 0,940 | 157,4 | 29,2% | 0,28 | 0,019 | 59 | 22,9% | 0,37 | 0,600 | 0,27 | 0,471 | 80,7 | 52,3% | 0,51 | 0,592 | 0,34 | 0,353 | 0,73 | 0,898 |
| **7727** | 177,8 | 41,4% | 79 | 46,8% | 0,44 | 0,631 | 70 | 46,5% | 0,39 | 0,567 | 1,13 | 0,967 | 58,2 | 28,2% | 0,33 | 0,474 | 29,1 | 14,2% | 0,50 | 0,877 | 0,37 | 0,823 | 38,3 | 23,7% | 0,66 | 0,889 | 0,55 | 0,85 | 0,76 | 0,956 |
| **7812** | 464,4 | 11,0% | 457,3 | 30,2% | 0,98 | 0,972 | 757,8 | 28,2% | 1,63 | 0,121 | 0,60 | 0,177 | 69,1 | 61,0% | 0,15 | 0,019 | 165,6 | 92,5% | 2,40 | 0,607 | 0,36 | 0,192 | 94,3 | 71,6% | 1,36 | 0,860 | 0,12 | 0 | 1,76 | 0,672 |
| **8004** | 1575 | 32,0% | 1072,3 | 72,6% | 0,68 | 0,016 | 747,4 | 74,1% | 0,47 | 0,000 | 1,43 | 0,145 | 604 | 14,8% | 0,38 | 0,000 | 527,7 | 23,9% | 0,87 | 0,684 | 0,49 | 0,016 | 428,8 | 9,1% | 0,71 | 0,222 | 0,57 | 0,059 | 1,23 | 0,558 |
| **8005** | 719,8 | 56,1% | 979,2 | 64,5% | 1,36 | 0,208 | 1487,3 | 27,4% | 2,07 | 0,000 | 0,66 | 0,024 | 493 | 91,7% | 0,68 | 0,176 | 710,1 | 42,8% | 1,44 | 0,249 | 0,73 | 0,228 | 430,3 | 35,7% | 0,87 | 0,661 | 0,29 | 0 | 1,65 | 0,099 |
| **8006** | 784,1 | 24,7% | 963,1 | 65,0% | 1,23 | 0,384 | 1015,1 | 68,7% | 1,29 | 0,221 | 0,95 | 0,814 | 1168,9 | 24,5% | 1,49 | 0,023 | 536,6 | 21,4% | 0,46 | 0,001 | 0,56 | 0,058 | 583,6 | 7,0% | 0,50 | 0,000 | 0,57 | 0,011 | 0,92 | 0,780 |
| **8122** | 1295,4 | 29,5% | 1672,9 | 14,2% | 1,29 | 0,068 | 1192,3 | 23,6% | 0,92 | 0,584 | 1,40 | 0,032 | 494,6 | 15,3% | 0,38 | 0,000 | 1300,7 | 28,7% | 2,63 | 0,000 | 0,78 | 0,097 | 875,4 | 39,5% | 1,77 | 0,009 | 0,73 | 0,061 | 1,49 | 0,013 |
| **8127** | 290,3 | 42,2% | 486,6 | 50,2% | 1,68 | 0,340 | 572,5 | 13,8% | 1,97 | 0,135 | 0,85 | 0,698 | 737,1 | 19,1% | 2,54 | 0,008 | 1241,1 | 14,9% | 1,68 | 0,008 | 2,55 | 0,001 | 974,4 | 16,0% | 1,32 | 0,099 | 1,7 | 0,018 | 1,27 | 0,116 |
| **8128** | 192,9 | 45,2% | 310,5 | 57,2% | 1,61 | 0,567 | 467,2 | 63,4% | 2,42 | 0,147 | 0,66 | 0,480 | 527,2 | 18,2% | 2,73 | 0,047 | 621,6 | 19,2% | 1,18 | 0,615 | 2,00 | 0,164 | 485,2 | 24,4% | 0,92 | 0,769 | 1,04 | 0,914 | 1,28 | 0,419 |
| **8131** | 15,5 | 114,2% | 116,4 | 48,2% | 7,51 | 0,623 | 286,8 | 51,6% | 18,50 | 0,151 | 0,41 | 0,443 | 204,1 | 39,9% | 13,17 | 0,259 | 465,7 | 14,5% | 2,28 | 0,165 | 4,00 | 0,119 | 351,1 | 4,2% | 1,72 | 0,305 | 1,22 | 0,701 | 1,33 | 0,497 |
| **8305** | 1139,5 | 14,6% | 587,9 | 32,1% | 0,52 | 0,008 | 791,7 | 7,5% | 0,69 | 0,067 | 0,74 | 0,359 | 559,4 | 50,1% | 0,49 | 0,001 | 455,9 | 24,4% | 0,81 | 0,581 | 0,78 | 0,553 | 411,4 | 6,4% | 0,74 | 0,302 | 0,52 | 0,025 | 1,11 | 0,792 |
| **8306** | 3398,5 | 8,4% | 2535,7 | 16,8% | 0,75 | 0,000 | 2726,7 | 15,7% | 0,80 | 0,001 | 0,93 | 0,390 | 1479,3 | 32,4% | 0,44 | 0,000 | 1492,9 | 42,0% | 1,01 | 0,942 | 0,59 | 0,000 | 1785,6 | 24,1% | 1,21 | 0,034 | 0,65 | 0 | 0,84 | 0,085 |
| **8307** | 222,2 | 17,3% | 567,8 | 32,4% | 2,56 | 0,094 | 761,6 | 14,6% | 3,43 | 0,005 | 0,75 | 0,383 | 990,4 | 30,0% | 4,46 | 0,000 | 995,1 | 48,6% | 1,00 | 0,980 | 1,75 | 0,057 | 1259,9 | 17,9% | 1,27 | 0,062 | 1,65 | 0,004 | 0,79 | 0,118 |
| **8310** | 950,1 | 13,1% | 413,8 | 17,1% | 0,44 | 0,010 | 432,5 | 9,2% | 0,46 | 0,007 | 0,96 | 0,933 | 136,5 | 78,5% | 0,14 | 0,000 | 115,5 | 73,6% | 0,85 | 0,911 | 0,28 | 0,182 | 130,4 | 24,7% | 0,96 | 0,966 | 0,3 | 0,073 | 0,89 | 0,930 |
| **8311** | 354 | 14,1% | 295,7 | 26,5% | 0,84 | 0,776 | 434,6 | 9,2% | 1,23 | 0,668 | 0,68 | 0,531 | 208,8 | 52,7% | 0,59 | 0,385 | 175,3 | 79,3% | 0,84 | 0,858 | 0,59 | 0,589 | 94,3 | 45,8% | 0,45 | 0,424 | 0,22 | 0,044 | 1,86 | 0,631 |
| **8401** | 1087,7 | 10,2% | 840,9 | 30,0% | 0,77 | 0,231 | 949,8 | 21,7% | 0,87 | 0,464 | 0,89 | 0,623 | 451,2 | 45,6% | 0,41 | 0,000 | 365,9 | 40,3% | 0,81 | 0,650 | 0,44 | 0,035 | 434,2 | 22,0% | 0,96 | 0,905 | 0,46 | 0,003 | 0,84 | 0,685 |
| **8405** | 810,7 | 3,6% | 354,4 | 14,0% | 0,44 | 0,028 | 369,2 | 6,6% | 0,46 | 0,021 | 0,96 | 0,946 | 223,1 | 64,1% | 0,28 | 0,001 | 146,5 | 50,7% | 0,66 | 0,683 | 0,41 | 0,351 | 99,2 | 9,3% | 0,44 | 0,387 | 0,27 | 0,109 | 1,48 | 0,779 |
| **8510** | 1312,6 | 3,5% | 888 | 2,3% | 0,68 | 0,041 | 829 | 23,5% | 0,63 | 0,011 | 1,07 | 0,790 | 1929,7 | 19,7% | 1,47 | 0,000 | 1746,6 | 19,6% | 0,91 | 0,330 | 1,97 | 0,000 | 1830,7 | 7,0% | 0,95 | 0,489 | 2,21 | 0 | 0,95 | 0,618 |
| **8511** | 1348,3 | 30,2% | 898,3 | 21,9% | 0,67 | 0,030 | 824,1 | 28,0% | 0,61 | 0,006 | 1,09 | 0,738 | 10,6 | 41,8% | 0,01 | 0,000 | 4 | 81,8% | 0,38 | 0,972 | 0,00 | 0,000 | 34,6 | 71,0% | 3,26 | 0,867 | 0,04 | 0 | 0,12 | 0,856 |
| **8520** | 695,3 | 34,9% | 493,5 | 34,9% | 0,71 | 0,327 | 276,6 | 38,9% | 0,40 | 0,028 | 1,78 | 0,329 | 6,1 | 62,0% | 0,01 | 0,000 | 6,1 | 106,8% | 1,00 | 1,000 | 0,01 | 0,030 | 10,5 | 29,4% | 1,72 | 0,975 | 0,04 | 0,114 | 0,58 | 0,979 |
| **8521** | 334,3 | 3,5% | 132,9 | 85,0% | 0,40 | 0,328 | 56,2 | 71,3% | 0,17 | 0,141 | 2,36 | 0,730 | 1,7 | 72,1% | 0,01 | 0,048 | 1,9 | 71,0% | 1,12 | 0,999 | 0,01 | 0,556 | 0,5 | 57,9% | 0,29 | 0,993 | 0,01 | 0,739 | 3,80 | 0,994 |
| **8524** | 361,2 | 17,4% | 226,1 | 14,9% | 0,63 | 0,511 | 290 | 22,9% | 0,80 | 0,705 | 0,78 | 0,773 | 51,7 | 51,3% | 0,14 | 0,066 | 62,8 | 111,3% | 1,21 | 0,953 | 0,28 | 0,464 | 28,8 | 54,0% | 0,56 | 0,872 | 0,1 | 0,121 | 2,18 | 0,840 |
| **8528** | 325,8 | 13,2% | 362,9 | 37,8% | 1,11 | 0,857 | 356,6 | 11,3% | 1,09 | 0,870 | 1,02 | 0,977 | 784,3 | 11,5% | 2,41 | 0,007 | 768,3 | 69,6% | 0,98 | 0,932 | 2,12 | 0,071 | 786,7 | 18,2% | 1,00 | 0,987 | 2,21 | 0,011 | 0,98 | 0,913 |
| **8529** | 536 | 30,8% | 516,7 | 51,9% | 0,96 | 0,925 | 435,3 | 27,1% | 0,81 | 0,592 | 1,19 | 0,713 | 285,2 | 64,0% | 0,53 | 0,134 | 652,6 | 62,7% | 2,29 | 0,052 | 1,26 | 0,542 | 829,7 | 20,9% | 2,91 | 0,000 | 1,91 | 0,02 | 0,79 | 0,294 |
| **8604** | 492,9 | 25,5% | 279,1 | 41,0% | 0,57 | 0,299 | 263,1 | 16,0% | 0,53 | 0,223 | 1,06 | 0,943 | 101,6 | 73,0% | 0,21 | 0,021 | 71,1 | 77,9% | 0,70 | 0,871 | 0,25 | 0,351 | 77 | 19,1% | 0,76 | 0,863 | 0,29 | 0,268 | 0,92 | 0,972 |
| **8611** | 61,3 | 23,1% | 1207,4 | 50,3% | 19,70 | 0,010 | 927,6 | 47,4% | 15,13 | 0,020 | 1,30 | 0,571 | 120,5 | 98,6% | 1,97 | 0,864 | 257,7 | 69,9% | 2,14 | 0,720 | 0,21 | 0,044 | 1718,1 | 54,3% | 14,26 | 0,002 | 1,85 | 0,12 | 0,15 | 0,003 |
| **8615** | 258 | 16,1% | 139,1 | 67,6% | 0,54 | 0,785 | 37,9 | 63,7% | 0,15 | 0,548 | 3,67 | 0,838 | 767,2 | 84,2% | 2,97 | 0,142 | 2,7 | 109,5% | 0,00 | 0,048 | 0,02 | 0,769 | 12,9 | 79,1% | 0,02 | 0,126 | 0,34 | 0,96 | 0,21 | 0,983 |
| **8702** | 782 | 7,8% | 200,8 | 6,4% | 0,26 | 0,184 | 140,9 | 38,4% | 0,18 | 0,083 | 1,43 | 0,903 | 299,4 | 36,9% | 0,38 | 0,164 | 188,6 | 50,7% | 0,63 | 0,772 | 0,94 | 0,979 | 170,5 | 19,1% | 0,57 | 0,792 | 1,21 | 0,953 | 1,11 | 0,970 |
| **8707** | 1070,8 | 15,1% | 374,3 | 37,9% | 0,35 | 0,112 | 675,1 | 27,9% | 0,63 | 0,281 | 0,55 | 0,543 | 254,6 | 91,0% | 0,24 | 0,020 | 275 | 65,8% | 1,08 | 0,957 | 0,73 | 0,831 | 392,2 | 11,0% | 1,54 | 0,779 | 0,58 | 0,575 | 0,70 | 0,806 |
| **8711** | 137,4 | 32,2% | 153,3 | 29,1% | 1,12 | 0,971 | 124,7 | 29,5% | 0,91 | 0,972 | 1,23 | 0,954 | 4,1 | 54,7% | 0,03 | 0,699 | 3,6 | 12,6% | 0,88 | 0,999 | 0,02 | 0,748 | 2,4 | 34,3% | 0,59 | 0,047 | 0,02 | 0,809 | 1,50 | 0,998 |
| **9012** | 3507,9 | 20,8% | 3457 | 29,7% | 0,99 | 0,907 | 3586,4 | 28,9% | 1,02 | 0,830 | 0,96 | 0,793 | 6545,5 | 6,7% | 1,87 | 0,000 | 2218,7 | 28,5% | 0,34 | 0,000 | 0,64 | 0,009 | 2607,1 | 25,4% | 0,40 | 0,000 | 0,73 | 0,055 | 0,85 | 0,416 |
| **9013** | 1454,1 | 15,1% | 1673,5 | 53,0% | 1,15 | 0,614 | 2157,2 | 29,3% | 1,48 | 0,057 | 0,78 | 0,328 | 2047,4 | 29,3% | 1,41 | 0,088 | 717,3 | 39,3% | 0,35 | 0,001 | 0,43 | 0,042 | 912,1 | 17,2% | 0,45 | 0,023 | 0,42 | 0,015 | 0,79 | 0,683 |
| **9103** | 639,8 | 34,7% | 3304,7 | 74,9% | 5,17 | 0,000 | 3232,8 | 42,6% | 5,05 | 0,000 | 1,02 | 0,884 | 1437,3 | 54,8% | 2,25 | 0,023 | 3794,4 | 44,9% | 2,64 | 0,000 | 1,15 | 0,294 | 5956 | 46,9% | 4,14 | 0,000 | 1,84 | 0 | 0,64 | 0,000 |
| **9108** | 3072,9 | 13,1% | 2031 | 12,0% | 0,66 | 0,019 | 2168,4 | 30,1% | 0,71 | 0,015 | 0,94 | 0,781 | 7,9 | 83,4% | 0,00 | 0,000 | 1,4 | 152,9% | 0,18 | 0,986 | 0,00 | 0,000 | 20,9 | 97,3% | 2,65 | 0,979 | 0,01 | 0 | 0,07 | 0,967 |
| **9109** | 436,8 | 72,4% | 460,7 | 35,1% | 1,05 | 0,956 | 1032,1 | 18,8% | 2,36 | 0,107 | 0,45 | 0,249 | 146,7 | 18,1% | 0,34 | 0,401 | 244,1 | 18,1% | 1,66 | 0,799 | 0,53 | 0,642 | 373,1 | 82,9% | 2,54 | 0,644 | 0,36 | 0,194 | 0,65 | 0,787 |
| **9206** | 1857,9 | 23,9% | 604,7 | 47,1% | 0,33 | 0,005 | 663 | 37,4% | 0,36 | 0,002 | 0,91 | 0,906 | 1540,9 | 24,3% | 0,83 | 0,359 | 699,4 | 26,2% | 0,45 | 0,030 | 1,16 | 0,839 | 1104 | 4,3% | 0,72 | 0,373 | 1,67 | 0,383 | 0,63 | 0,397 |
| **9211** | 1216,3 | 12,0% | 834 | 3,1% | 0,69 | 0,381 | 894,9 | 15,2% | 0,74 | 0,381 | 0,93 | 0,902 | 574,7 | 32,4% | 0,47 | 0,066 | 476,9 | 19,0% | 0,83 | 0,798 | 0,57 | 0,444 | 576,6 | 23,0% | 1,00 | 0,997 | 0,64 | 0,529 | 0,83 | 0,835 |
| **9213** | 1243,1 | 20,4% | 835,2 | 13,7% | 0,67 | 0,350 | 814,4 | 2,9% | 0,66 | 0,243 | 1,03 | 0,966 | 609 | 38,2% | 0,49 | 0,069 | 525,4 | 27,6% | 0,86 | 0,827 | 0,63 | 0,506 | 561,3 | 28,1% | 0,92 | 0,922 | 0,69 | 0,616 | 0,94 | 0,940 |
| **9214** | 1331,9 | 20,8% | 724,1 | 3,0% | 0,54 | 0,165 | 687,5 | 14,5% | 0,52 | 0,081 | 1,05 | 0,941 | 504,5 | 5,0% | 0,38 | 0,018 | 361,6 | 31,1% | 0,72 | 0,709 | 0,50 | 0,437 | 392,1 | 16,8% | 0,78 | 0,819 | 0,57 | 0,559 | 0,92 | 0,949 |
| **9216** | 2054,6 | 13,4% | 1421,5 | 11,3% | 0,69 | 0,148 | 1875,4 | 23,4% | 0,91 | 0,000 | 0,76 | 0,359 | 985,2 | 31,9% | 0,48 | 0,003 | 730,5 | 21,6% | 0,74 | 0,505 | 0,51 | 0,140 | 763,7 | 12,2% | 0,78 | 0,651 | 0,41 | 0,03 | 0,96 | 0,944 |
| **9217** | 1419,3 | 10,6% | 650 | 38,3% | 0,46 | 0,080 | 802,7 | 30,1% | 0,57 | 0,095 | 0,81 | 0,757 | 703 | 86,0% | 0,50 | 0,040 | 400,2 | 85,0% | 0,57 | 0,429 | 0,62 | 0,592 | 527,6 | 48,4% | 0,75 | 0,720 | 0,66 | 0,586 | 0,76 | 0,789 |
| **9308** | 2360,6 | 23,1% | 782,5 | 27,9% | 0,33 | 0,000 | 1270,8 | 18,1% | 0,54 | 0,004 | 0,62 | 0,324 | 1237,4 | 52,9% | 0,52 | 0,002 | 884,8 | 56,3% | 0,72 | 0,357 | 1,13 | 0,826 | 1196,4 | 8,8% | 0,97 | 0,933 | 0,94 | 0,883 | 0,74 | 0,514 |
| **9701** | 1620,1 | 32,2% | 346,5 | 38,7% | 0,21 | 0,004 | 173,5 | 25,0% | 0,11 | 0,000 | 2,00 | 0,726 | 1933,1 | 71,7% | 1,19 | 0,365 | 316,7 | 70,8% | 0,16 | 0,000 | 0,91 | 0,949 | 459,9 | 96,4% | 0,24 | 0,003 | 2,65 | 0,571 | 0,69 | 0,764 |
| **9801** | 2486,4 | 25,7% | 3285,9 | 11,7% | 1,32 | 0,069 | 3187 | 32,1% | 1,28 | 0,058 | 1,03 | 0,841 | 40,4 | 59,1% | 0,02 | 0,000 | 110,7 | 93,3% | 2,74 | 0,854 | 0,03 | 0,000 | 97,8 | 85,8% | 2,42 | 0,907 | 0,03 | 0 | 1,13 | 0,978 |
| **9802** | 378,8 | 28,3% | 595,2 | 42,5% | 1,57 | 0,619 | 701,2 | 44,6% | 1,85 | 0,379 | 0,85 | 0,830 | 13,6 | 77,4% | 0,04 | 0,291 | 29,7 | 61,0% | 2,18 | 0,967 | 0,05 | 0,226 | 19,8 | 84,3% | 1,46 | 0,990 | 0,03 | 0,179 | 1,50 | 0,983 |

**Figure S1.** Reference map for peach fruit proteins. The specific proteins spots labelled with the arrows correspond to proteins listed in the Supplementary Data Table S2.

**Figure S2.** Full-length gels of peach fruit proteins of ‘Rich Lady’ control fruits (RLC), ‘Rich Lady’ fruits inoculated with *M. laxa* (RL1L) and *M. fructicola* (RL101F), ‘Royal Glory’ control fruits (RGC), ‘Royal Glory’ fruits inoculated with *M. laxa* (RGL) and *M. fructicola* (RGF) of which zoomed in views of selected areas are presented in Figure 2.
